# Phenyl-Substituted Cibalackrot Derivatives: Synthesis, Structure, and Solution Photophysics

**DOI:** 10.1021/acs.joc.2c02706

**Published:** 2023-05-23

**Authors:** Jiří Kaleta, Miroslav Dudič, Lucie Ludvíková, Alan Liška, Alexandr Zaykov, Igor Rončević, Milan Mašát, Lucie Bednárová, Paul I. Dron, Simon J. Teat, Josef Michl

**Affiliations:** † Institute of Organic Chemistry and Biochemistry of the Czech Academy of Sciences, Flemingovo nám. 2, 16610 Prague, Czech Republic; ‡ J. Heyrovsky Institute of Physical Chemistry, Academy of Sciences of the Czech Republic, Dolejškova 3, 182 23 Prague 8, Czech Republic; § Advanced Light Source, Lawrence Berkeley National Laboratory, Berkeley, California 94720-1460, United States; ∥ Department of Chemistry, University of Colorado, Boulder, Colorado 80309-0215, United States; # University of Chemistry and Technology, Technicka 5, 16000 Prague 6, Czech Republic

## Abstract

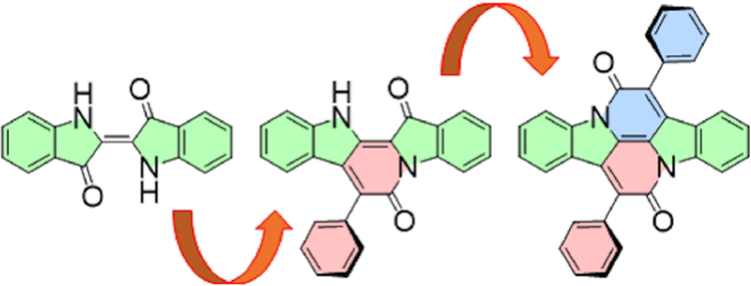

Three symmetrically and three unsymmetrically substituted cibalackrot (7,14-diphenyldiindolo[3,2,1-*de*:3′,2′,1′-*ij*][1,5]naphthyridine-6,13-dione, **1**) dyes carrying two derivatized phenyl rings have been synthesized as candidates for molecular electronics and especially for singlet fission, a process of interest for solar energy conversion. Solution measurements provided singlet and triplet excitation energies and fluorescence yields and lifetimes; conformational properties were analyzed computationally. The molecular properties are close to ideal for singlet fission. However, crystal structures, obtained by single-crystal X-ray diffraction (XRD), are rather similar to those of the polymorphs of solid **1**, in which the formation of a charge-separated state followed by intersystem crossing, complemented with excimer formation, outcompetes singlet fission. Results of calculations by the approximate SIMPLE method suggest which ones among the solid derivatives are the best candidates for singlet fission, but it appears difficult to change the crystal packing in a desirable direction. We also describe the preparation of three specifically deuteriated versions of **1**, expected to help sort out the mechanism of fast intersystem crossing in its charge-separated state.

## Introduction

The sturdy indigoid industrial dye known as cibalackrot (7,14-diphenyldiindolo[3,2,1-*de*:3′,2′,1′-*ij*][1,5]naphthyridine-6,13-dione, **1**)^1,2^ and its analogues containing thienyl instead of phenyl groups^3,4^ have been of potential interest as organic electronic,^5,6^ photonic,^7^ and singlet fission^3,8−12^ materials. Of particular appeal to us is singlet fission,^13^ a process in which a singlet exciton splits into two triplet excitons, promising to overcome the Shockley–Queisser limit^14^ of about 1/3 to single-junction solar cell efficiency.^15^ Although it has already been demonstrated in the laboratory that singlet fission cells are capable of yielding more than one electron–hole pair per absorbed photon,^16−23^ the experiments relied on materials that are probably not sufficiently sturdy to be used in practice.

The two main criteria for a new singlet fission solid are (i) a sturdy molecular chromophore with no fast intramolecular decay channels and excitation energies of ∼1.2 eV for T_1_ and ∼2.2–2.4 eV for S_1_, and (ii) a packing in solid that introduces no fast intermolecular decay channels, preserves the ratio of S_1_ to T_1_ excitation energies, and provides a large matrix element for singlet fission. The light fastness of **1**, its basic solution photophysics,^24^ and its redox properties^5^ appeared promising, but a detailed study^8^ showed that in both known crystal modifications and the amorphous phase, intermolecular interactions stabilize S_1_ more than T_1_ and make singlet fission so endothermic that it is outcompeted by the formation of an excimer and a charge-separated state, which act as traps. Calculations for **1**(8) predicted other possible packing motifs in which this problem would be avoided, and we wondered whether substituents could force **1** to pack better. One could also use substitution to increase the already nearly ideal 2.25/1.3 ratio of molecular S_1_ to T_1_ excitation energies^3,9,10^ by reducing the latter to 1.2 eV, and this route might be worth pursuing in the future.

**Chart 1:**
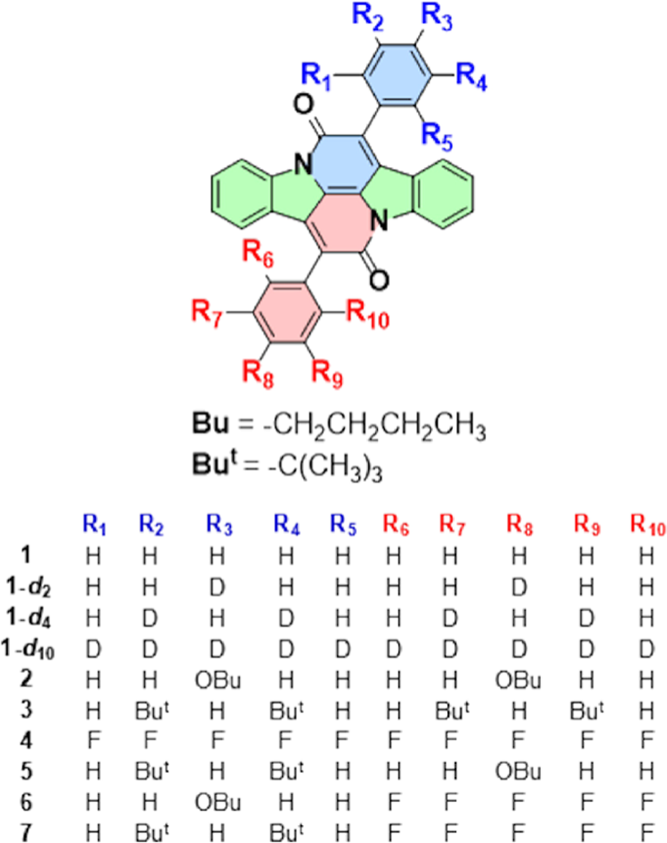
Cibalackrot and its Derivatives

We prepared six derivatives of **1** substituted on phenyl groups, including the previously inaccessible unsymmetrical ones, in which different substituents are present on the two phenyls (Chart 1). In preparation for an investigation of their solid-state photophysics, we report their solution properties: absorption, excitation, and emission spectra, including quantum yields and lifetimes, triplet–triplet absorption spectra, and triplet excitation energies from bracketing sensitization. We include the mostly already reported^8^ properties of **1**. We have obtained the X-ray diffraction (XRD) structures of five of the new derivatives, with attention paid to their con and dis conformers, which differ in the relative orientation of the two aryl groups. We use the crystal packing structures to estimate anticipated relative singlet fission rate constants. Information on the required redox properties and radical ion absorption spectra is being published separately.

We examine how easily the packing can be changed by the addition of bulky *t*-butyl groups, a butoxy chain, or an introduction of an opportunity for a different π-stacking (perfluorinated/ordinary ring). These substituents cause a very small (≤600 cm^–1^) perturbation of molecular S_1_ and T_1_ excitation energies.

## Results

### Synthesis

**Figure 1:**
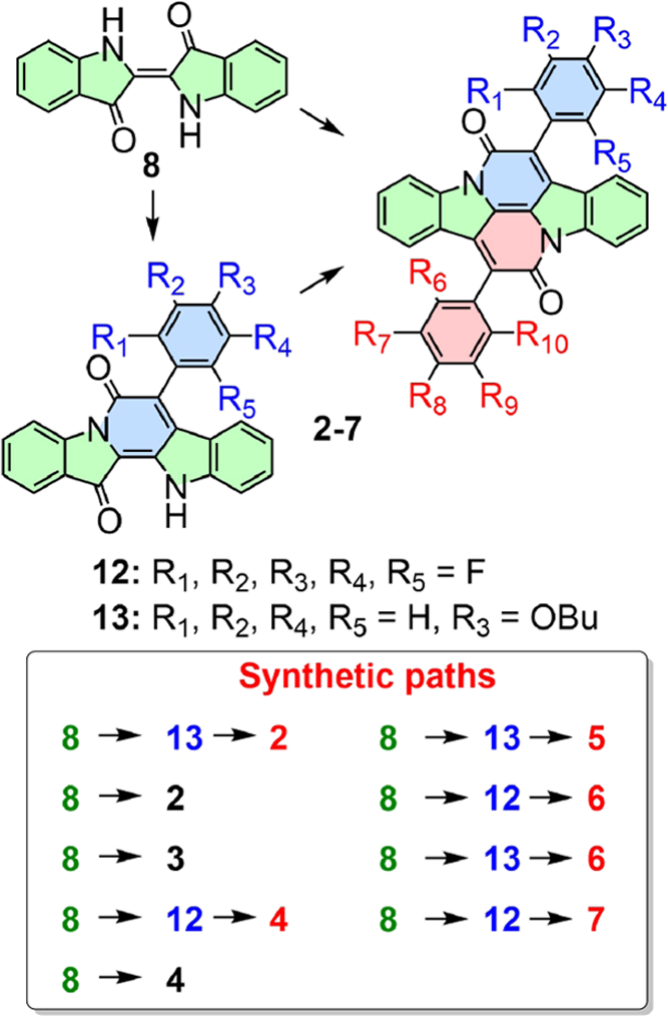
Pathways to cibalackrots.

We report syntheses of six new derivatives **2**–**7** (Chart 1). The symmetrical species **2–4**, carrying two identical aryl substituents on the central indigoid moiety, were obtained by the standard synthetic approach to **1**, reflux of indigo (**8**) with an excess of an arylacetyl chloride in a high boiling solvent.^25^ A discovery of conditions under which indigo condenses with only one equivalent of an arylacetyl chloride to yield a “half-cibalackrot” that precipitates and does not react further allowed us to perform the reaction in two separate steps with two different arylacetyl chlorides, thus gaining access to the unsymmetrical species **5**–**7** (Figure 1). Even though the solvent, reaction time, and isolation procedure were optimized in each reaction, the syntheses proceeded in poor yields, both because of low conversion and demanding purification. The quality of the freshly distilled arylacetyl chlorides obtained from arylacetic acids in boiling thionyl chloride was particularly important. The yields of **2**, best obtained with an excess of 4-butoxyphenylacetyl chloride **9** in boiling 1,1,2,2-tetrachloroethane, and **3**, best obtained with 3,5-di-*tert*-butylphenylacetyl chloride **10** in boiling xylene, were below 10%. The best route to **4** is the reaction of **8** with neat pentafluorophenylacetyl chloride **11** at 155 °C for 24 h, which produces a precipitate of **12**. Dilution with 1,1,2,2-tetrachloroethane and reflux for two more days afforded **4** in 19% yield.

In the syntheses of the asymmetric cibalackrots **5**–**7**, it is crucial to first form the less soluble half-cibalackrot and to allow it to precipitate from the reaction mixture, suppressing the formation of the undesired symmetrical cibalackrot. The unsymmetrical **5** was prepared from **13**, obtained in 38% yield by reaction of **8** with excess **9**, by condensation with **10** in boiling xylene in 54% yield. Both possible approaches were used for **6**. A reaction of one equivalent of **8** with 2 equiv of **11** in boiling 1,1,2,2-tetrachloroethane provided **12** (9%), which was then refluxed with **9** in xylene, yielding 33% of **6**. When the reaction was performed in the reverse order, the yield of **6** was increased to 65%. The reaction of **12** with **10** in refluxing 1,1,2,2-tetrachloroethane afforded **7** in 13% yield.

The specifically deuteriated compounds **1**-*d*_2_, **1**-*d*_4_, and **1**-*d*_10_ (Figure 1) were prepared by refluxing **8** in a high boiling solvent with deuteriated acyl chlorides **22**, **23**, or **24**, obtained from their acids using thionyl chloride.^26^ Synthesis of the phenylacetic acids started with the conversion of *p*-bromotoluene (**14**) and 3,5-dibromotoluene (**15**) into Grignard reagents and treating them with D_2_O to obtain toluene-4-*d* (**16**)^27^ and toluene-3,5-*d*_2_ (**17**).^28^ A radical bromination of these toluenes and commercial toluene-*d*_8_ (**18**), followed by reaction with NaCN^29^ and hydrolysis^24^ yielded (phenyl-4-*d*)acetic (**19**),^30^ (phenyl-3,5-*d*_2_)acetic (**20**),^28^ and (phenyl-*d*_5_)acetic (**21**)^24^ acids.

The approximate room-temperature solubilities of these cibalackrots in dichloromethane are **1**, **2**: ∼10^–5^ M; **4**, **5**, **6**: ∼10^–4^ M; and **3**, **7**: ≥10^–3^ M.

### Structure

#### Calculated for **1**–**7** in Solution

**Figure 2:**
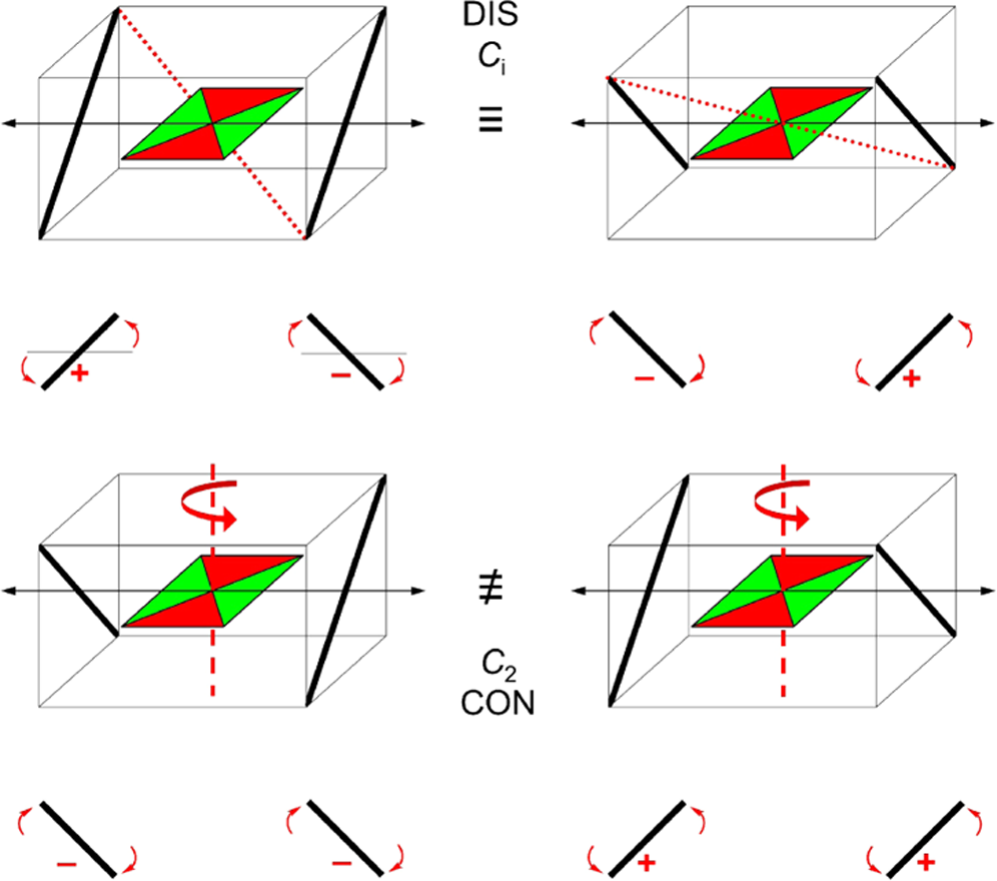
Schematic representation of cibalackrot (**1**) conformers. Top, the centrosymmetric disrotatory conformer (*C_i_*, meso, achiral), with the inversion symmetry exemplified by a dotted red line; a 180° rotation of the molecule about a vertical axis converts one into another view of the same conformer. Bottom, the enantiomeric (mirror image) pair of distinct conrotatory conformers (*C*_2_, chiral), with the twofold symmetry axis in each indicated by a dashed red line and a curved arrow. The centrosymmetric indigoid core is symbolized by a horizontal rectangle, with the amide region in red and the substituent carrying region in green, and the two aryl substituent planes are symbolized by fat bars. Underneath, views of the aryls from the center of the molecule in the direction of black double-headed arrows. The sense of rotation from the horizontal plane is clockwise (−) or counterclockwise (+) viewed from the center of the molecule.

There is nothing unusual about the bond lengths and angles in the central indigoid part of the molecules observed in crystals or calculated for the electronic ground state of an isolated molecule by density functional theory (DFT), which are in good agreement. The important variable structural characteristics are the calculated angles of rotation of the two aryl substituents. They were reported earlier^8^ for parent **1** and are now also available for its derivatives **2**–**7** (Table 1). According to expectations and DFT calculations, in general, these molecules have two pairs of enantiomeric conformations (Figure 2). The pairs differ by the clockwise or counterclockwise sense of rotation of the two aryl substituents that allows the conformer to reach its equilibrium geometry starting from a hypothetical all-planar species of *C*_2*h*_ symmetry. When viewed from the center of the molecule, as was done in the earlier work on **1**,^8^ the sense can be the same (the con conformer) or opposite (the dis conformer). When the substituents on the two aryls are different (**5**–**7**), symmetry is absent, and the mixture of four distinct conformers consists of two pairs of enantiomers (point group *C*_1_). When the two aryls are equal (**1**–**4**) or if only the parent skeleton is considered, the situation simplifies. The con conformer has a twofold axis of symmetry, belongs to the *C*_2_ point group, and is chiral (the enantiomers in this pair differ in the sense of aryl rotation), whereas the dis conformer is centrosymmetric, belongs to the *C*_*i*_ point group, and is achiral (the two members of the pair are identical; this is the meso form).

**Table 1:** Observed and M06HF-GD3/cc-pVDZ Calculated Aryl Rotation Angles in **1**–**7** (deg)^a^

		S_0_^b^				T_1_^c^	
		(R_1_–R_5_)		(R_6_–R_10_)		(R_1_–R_5_)	(R_6_–R_10_)
		calcd^d^	obsd	calcd^d^	obsd	calcd	calcd
**1**	con	–53 (41)	–49^e^	–53 (41)	–52^e^	–43	–43
	dis	–53 (−42)	–53^f^	53 (42)	53^f^	–45	45
**2**	con	–50		–50		–40	–40
	dis	–51	–42	51	42	–42	42
**3**	con	–52		–50		–44	–42
	dis	–51	–77	52	77	–43	46
**4**	con	–57 (−53)		–57 (−53)		–53	–53
	dis	–56 (−53)	–59,^g^ −55^h^	56 (53)	59,^g^ 55^h^	–53	53
**5**	con	–52		–51		–44	–40
	dis	52	53	–51	–52	46	–42
**6**	con	–51	58	–56	63	–40	–53
	dis	50		–56		40	–54
**7**	con	–51		–56		–42	–53
	dis	–51	–46	57	54	–42	54

a Dihedral rotation angle of aryls carrying substituents R_1_–R_5_ or R_6_–R_10_, turned counterclockwise (+) or clockwise (−) when viewed from the center, starting with a planar molecule. One plane is defined by the ortho and para carbon atoms of the aryl, and the other is defined by analogous carbon atoms of the pyridone ring in the central indigoid part to which the aryl is attached, two adjacent to the ipso carbon and one para to it. Only results for one member of an enantiomeric pair are given. Data for **1** in ref (8).

b Ground state.

c First triplet state.

d Results for the first excited singlet S_1_ are in parentheses.

e Source document 1a.res.

f Source document 1b.cif.

g Source document 4a.cif.

h Source document 4b.cif.

The interconversion of the con and dis conformers can be accomplished by rotation of one of the aryl substituents into its other possible orientation, by motion either through a planar or an orthogonal orientation with respect to the central indigoid system. The former costs energy because it introduces steric interaction between hydrogens or fluorines in ortho positions of the aryl and the carbonyl oxygen and peri hydrogen on the indigoid core, and the latter costs energy because it removes conjugation between the aryl and the core. These activation energies were calculated for **1** and **4** as representatives for the rotation of phenyl and pentafluorophenyl (Table 2).

**Table 2:** B3LYP-D3/cc-pVTZ Calculated Relative Energies of Conformers of **1** and **4** in S_0_ and S_1_ States (kcal/mol)^a^

			S_0_		S_1_	
cmpd.	conf.	sym.	*E*_rel_	α/deg	*E*_rel_	α/deg
**1**	con	*C*_2_	0	48	0	41
	dis	*C*_*i*_	0.2	49	0.5	42
	ortho	*C*_2*h*_	2.0	90	4.0	90
	planar	*C*_2*h*_	8.2	0	6.0	0
**4**	con	*C*_2_	0	59	0	53
	dis	*C*_*i*_	0.0	58	0.1	53
	ortho	*C*_2*h*_	1.3	90	2.6	90
	planar	*C*_2*h*_	25.4	0	72.7	0

a Con and dis geometries are fully relaxed. Orthogonal and planar geometries are fully relaxed, except that the aryl rotation angle α was constrained to 90 and 0°, respectively.

We have examined the ^1^H, ^13^C, and ^19^F NMR spectra of **1** and **4** down to −90 °C (DCM-*d*_*2*_) but did not detect any indication of dynamic effects. The signals of the two nuclei attached to the ortho positions, and the two attached to the meta positions, remained averaged by fast conformer interconversion. This agrees with the results shown in Table 2, which predict a very fast interconversion via the orthogonal geometry.

#### Observed for **1–7** in Crystals

Like **1** itself, its derivatives tend to precipitate from solution as fine powders, and obtaining single crystals suitable for X-ray diffraction analysis is difficult. We have succeeded in solving five of the six structures, **2**–**4**, **6**, and **7**, and details are provided in the Supporting Information. None of the results are unusual. The primary items of interest are the twist angles of the aryl substituents (Table 1), which can be expected to affect the energies of electronic transitions, but do so only to a negligible degree.

### Solution Spectroscopy and Photophysics

**Figure 3:**
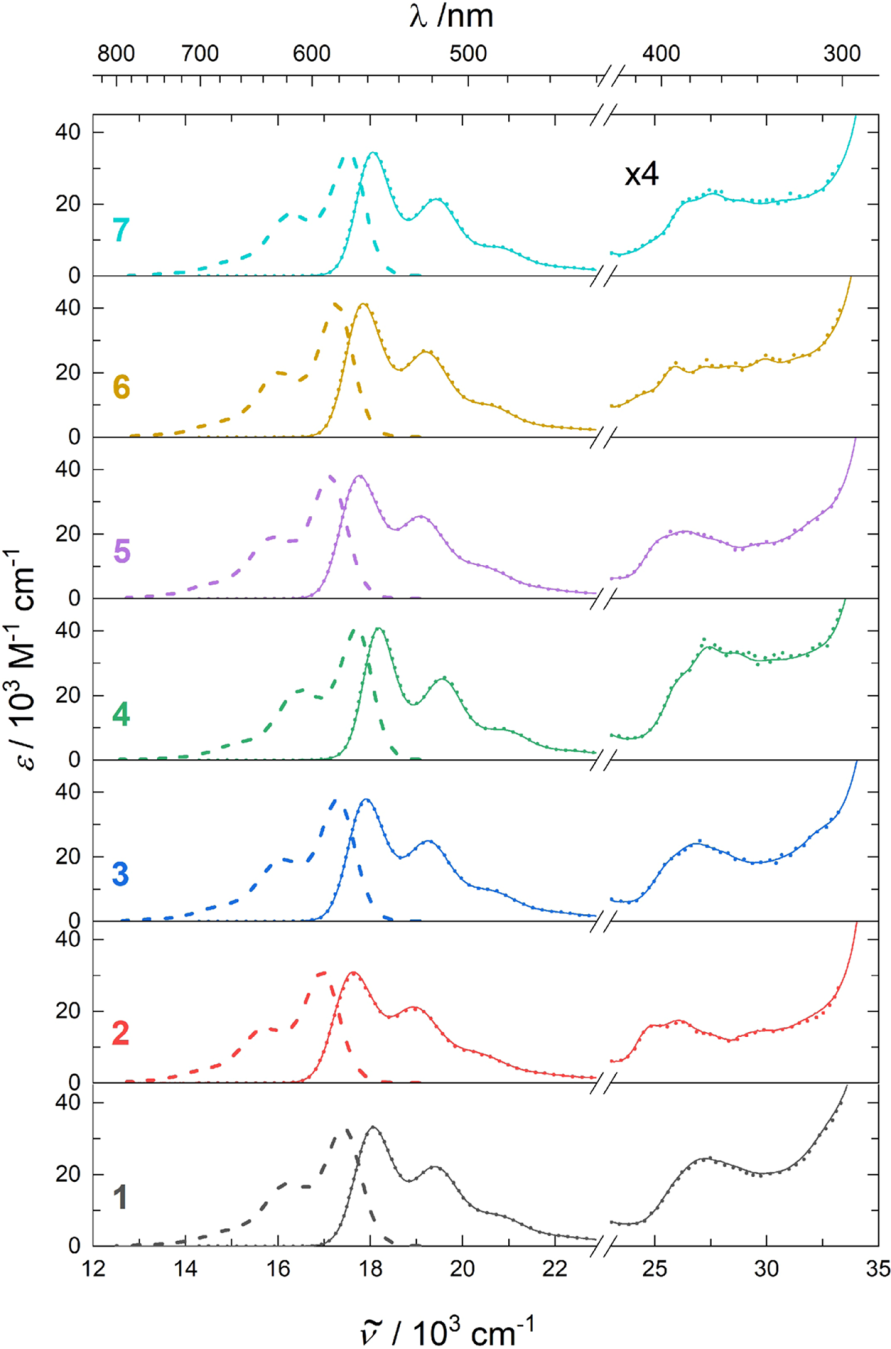
Steady-state absorption (full), fluorescence emission (dashed), and fluorescence excitation (dotted) spectra of **1**–**7** in toluene. The 34,000–23,000 cm^–1^ region is magnified 4 times.

**Figure 4:**
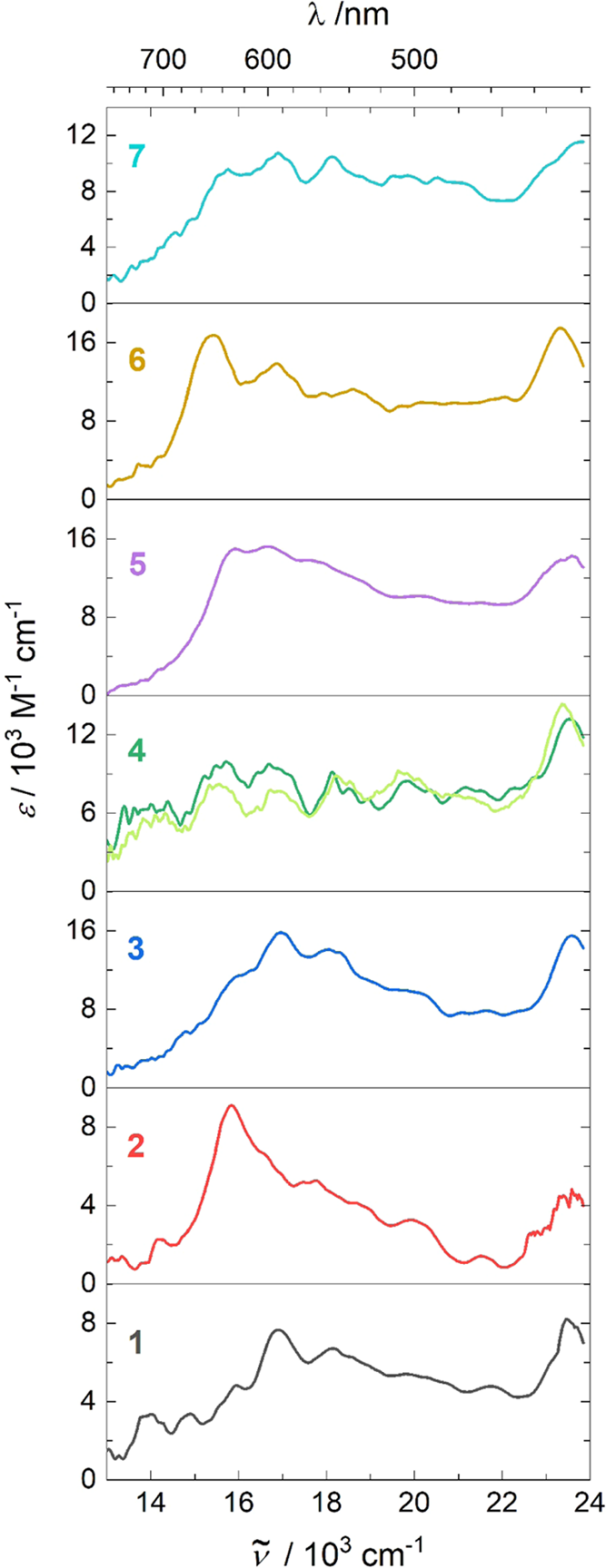
Triplet state absorption spectra of **1–7** in toluene derived from TA difference spectra of triplet states obtained from global analysis of TAS sensitization experiments by addition of ground state absorption spectra. The spectrum of 4 is noisy and was measured independently twice.

Absorption and fluorescence spectra in toluene (Figure 3) and sensitized triplet–triplet absorption spectra (Figure 4) are compared with the results of DFT calculations in Tables 3 and 4, respectively. Triplet excitation energies were obtained by the bracketing procedure. The Supporting Information lists results for singlet transitions in a better solvent, dichloromethane (Table S1), and provides details of the experimental procedures.

**Table 3:** Observed (obsd, in Toluene) and Calculated (con, dis) Properties of Singlet States of **1**–**7** (Energies in 10^3^ cm^–1^)

	con	dis	obsd	con	dis	obsd	con	dis	obsd	con	dis	obsd
property	**1**			**2**			**3**			**4**		
S_0_ → S_1_ (*f*)^a^	19.6 (0.57)	19.7 (0.56)	18.1 (0.22)	18.6 (0.77)	18.7 (0.75)	17.6 (0.22)	19.3 (0.68)	19.3 (0.67)	17.9 (0.25)	19.7 (0.52)	19.7 (0.52)	18.2 (0.24)
S_0_ → S_2_ (*f*)^a^	24.0 (0)	24.0 (0)		22.6 (0)	22.5 (0)		23.8 (0)	23.8 (0)		23.2 (0)	23.2 (0)	
S_0_ → S_3_ (*f*)^a^	27.0 (0.16)	27.0 (0.16)	27.2 (0.08)	24.7 (0.06)	24.6 (0.06)	26.0 (0.05)	25.7 (0.01)	25.7 (0.01)		27.0 (0.19)	27.0 (0.20)	27.4 (0.11)
S_0_ → S_4_ (*f*)^a^	28.3 (0)	28.3 (0)		26.0 (0)	26.0 (0)	29.6 (0.02)	26.1 (0.01)	26.1 (0.02)	26.8 (0.08)	27.8 (0)	27.8 (0)	
S_1_ → S_0_			17.4			17.0			17.3			17.7
S_0_ ave^b^			17.7			17.3			17.6			18.0
Φ_F_^c^			0.77			0.77			0.82			0.59
τ_F_/ns^d^			6.9			5.8			6.7			5.8
property	**5**			**6**			**7**					
S_0_ → S_1_ (*f*)^a^	18.9 (0.71)	19.0 (0.71)	17.8 (0.25)	18.9 (0.64)	18.9 (0.64)	17.9 (0.26)	19.4 (0.61)	19.4 (0.61)	18.1 (0.21)			
S_0_ → S_2_ (*f*)^a^	23.1 (0.01)	23.1 (0.01)		22.7 (0.02)	22.7 (0.02)	25.9 (0.04)	23.4 (0)	23.4 (0)				
S_0_ → S_3_ (*f*)^a^	25.2 (0.05)	25.2 (0.04)	26.4 (0.06)	24.7 (0.03)	24.7 (0.04)	27.2 (0.04)	25.1 (0)	25.1 (0)				
S_0_ → S_4_ (*f*)^a^	25.9 (0)	25.9 (0)	29.7 (0.02)	28.3 (0.04)	28.3 (0.05)	29.9 (0.03)	25.8 (0.09)	25.8 (0.09)	27.6 (0.07)			
S_1_ → S_0_			17.1			17.3			17.5			
S_0_ ave^b^			17.4			17.5			17.8			
Φ_F_^c^			0.81			0.80			0.75			
τ_F_/ns^d^			6.4			6.9			7.0			

a Oscillator strength, ; *n* = 1.49693.

b Obsd. crossing of normalized absorption and emission spectra.

c Fluorescence quantum yield. Standard: Rhodamine 6G in ethanol. Error, 0.05.

d Fluorescence lifetime. Error, ±0.1 ns.

**Table 4:** Observed (obsd, in Toluene) and Calculated (con, dis) Triplet Energies of **1**–**7** (Energies in 10^3^ cm^–1^)

	con	dis	obsd	con	dis	obsd	con	dis	obsd	con	dis	obsd
property	1			2			3			4		
T_1_ → T_2_ (*f*)^a^	9.6(0.05)	9.6(0.05)		9.6(0.16)	9.5(0.15)		9.7(0.08)	9.7(0.07)		8.8(0.03)	8.8(0.03)	
T_1_ → T_3_ (*f*)^a^	12.8(0)	12.8(0)		12.4(0)	12.3(0)		12.7(0)	12.7(0)		12.1(0)	12.1(0)	
T_1_ → T_4_ (*f*)^a^	15.2 (0.09)	15.2 (0.09)	15.9 (w)	14.4 (0.21)	14.3 (0.20)	15.8 (s)	14.3 (0)	14.3 (0)		14.6 (0.11)	14.6 (0.11)	15.6 (m)
T_1_ → T_5_ (*f*)^a^	16.8 (0)	16.7 (0.01)		16.7 (0.32)	16.5 (0.28)		14.7 (0)	14.6 (0)		16.0 (0)	16.0 (0)	
T_1_ → T_6_ (*f*)^a^	17.2 (0)	17.1 (0)		17.0 (0)	17.1 (0)		15.1 (0.11)	15.0 (0.11)	16.0 (m)	16.1 (0)	16.0 (0)	
T_1_ → T_7_ (*f*)^a^	17.7 (0.37)	17.6 (0.35)	16.9 (s)	18.3 (0)	17.9 (0.03)		17.2 (0.40)	17.1 (0.38)	16.9 (s)	16.4 (0)	16.4 (0)	16.7 (m)
T_1_ → T_8_ (*f*)^a^	18.2 (0)	18.2 (0)	18.2 (m)	18.4 (0.01)	18.4 (0)		18.2 (0)	18.3 (0)	18.2 (s)	17.9 (0)	17.9 (0.01)	
T_1_ → T_9_ (*f*)^a^	19.6 (0)	19.5 (0)		18.9 (0.03)	19.0 (0.03)	18.9 (w)	18.6 (0)	18.6 (0)		18.0 (0.27)	18.0 (0.27)	18.1 (m)
T_1_ → T_10_ (*f*)^a^	19.7 (0)	19.6 (0)		19.2 (0)	19.1 (0)		18.9 (0)	18.9 (0.01)		18.5 (0)	18.4 (0)	
T_1_ → T_11_ (*f*)^a^	19.7 (0.01)	19.9 (0.02)	20.0 (w)	20.0 (0)	20.1 (0)	19.9 (w)	19.1 (0)	19.1 (0)		19.0 (0)	19.1 (0.05)	
T_1_ → T_12_ (*f*)^a^	20.3 (0.01)	20.1 (0)		20.9 (0)	20.9 (0)		19.7 (0)	19.6 (0)		19.2 (0.06)	19.2 (0)	19.8 (m)
T_1_ → T_13_ (*f*)^a^	20.4 (0)	20.4 (0)		21.0 (0)	21.0 (0)		20.3 (0)	20.3 (0)	20.2 (w)	20.5 (0)	20.5 (0)	
T_1_ → T_14_ (*f*)^a^	21.2 (0)	21.3 (0)	21.7 (w)	21.2 (0)	21.1 (0)		21.1 (0)	21.2 (0)		21.3 (0)	21.3 (0)	
T_1_ → T_15_ (*f*)^a^	23.6 (0.26)	23.6 (0.25)	23.5 (s)	23.3 (0.18)	23.3 (0.20)	23.6 (m)	23.6 (0.28)	23.6 (0.27)	23.6 (s)	23.2 (0.20)	23.2 (0.20)	23.5 (s)
T_1_ → T_16_ (*f*)^a^	24.6 (0.06)	24.7 (0.06)		23.6 (0.11)	23.6 (0.09)		24.3 (0.04)	24.4 (0.04)		24.7 (0.10)	24.7 (0.10)	
S_0_ → T_1_^b^	9.9	9.9	10.2^d^	9.3	9.5	10.8	9.7	9.8	10.8	10.6	10.6	10.8
τ_T_/μs^c^			55^d^			93			83			103
property	**5**			**6**			**7**					
T_1_ → T_2_ (*f*)^a^	9.6(0.11)	9.6(0.11)		9.2(0.10)	9.2(0.09)		9.3(0.05)	9.3(0.05)				
T_1_ → T_3_ (*f*)^a^	12.5(0.01)	12.4(0.01)		12.0(0.04)	12.0(0.04)		12.3(0.01)	12.3(0.01)				
T_1_ → T_4_ (*f*)^a^	14.6 (0.05)	14.6 (0.04)		14.6 (0.11)	14.9 (0.12)	15.4 (s)	13.4 (0)	13.4 (0)				
T_1_ → T_5_ (*f*)^a^	15.0 (0.10)	14.8 (0.11)	15.9 (s)	16.2 (0.09)	16.2 (0.09)		15.1 (0.10)	15.1 (0.10)	15.7 (s)			
T_1_ → T_6_ (*f*)^a^	16.7 (0.25)	16.5 (0.23)	16.6 (s)	17.0 (0.05)	17.0 (0.04)	16.9 (m)	17.0 (0.19)	17.0 (0.18)	16.9 (s)			
T_1_ → T_7_ (*f*)^a^	17.6 (0.10)	17.7 (0.11)	17.6 (s)	17.8 (0.06)	17.8 (0.06)	17.9 (m)	17.5 (0.01)	17.5 (0)				
T_1_ → T_8_ (*f*)^a^	18.3 (0.03)	18.0 (0.01)		18.0 (0.06)	18.0 (0.06)		17.6 (0)	17.6 (0.01)				
T_1_ → T_9_ (*f*)^a^	18.9 (0)	18.9 (0)		18.1 (0.07)	18.0 (0.07)	18.6 (m)	18.0 (0.14)	18.0 (0.15)	18.1 (s)			
T_1_ → T_10_ (*f*)^a^	19.2 (0)	19.2 (0)		19.5 (0)	19.4 (0)		18.3 (0.01)	18.3 (0)				
T_1_ → T_11_ (*f*)^a^	19.6 (0)	19.6 (0)		19.9 (0)	19.9 (0)		19.4 (0)	19.4 (0)				
T_1_ → T_12_ (*f*)^a^	20.2 (0)	20.2 (0)	20.1 (w)	20.2 (0)	20.2 (0)		20.3 (0.01)	20.2 (0.01)	20.0 (m)			
T_1_ → T_13_ (*f*)^a^	20.8 (0)	20.7 (0)		20.7 (0.01)	20.7 (0.01)		20.6 (0.01)	20.5 (0.01)				
T_1_ → T_14_ (*f*)^a^	21.0 (0)	21.1 (0)		21.2 (0.02)	21.1 (0.01)		21.2 (0.01)	21.2 (0)				
T_1_ → T_15_ (*f*)^a^	23.5 (0.24)	23.5 (0.25)	23.6 (s)	23.1 (0.21)	23.2 (0.21)	23.3 (s)	23.2 (0.22)	23.2 (0.22)	23.7 (s)			
T_1_ → T_16_ (*f*)^a^	23.9 (0.06)	23.9 (0.04)		23.5 (0.06)	23.5 (0.05)		24.2 (0.05)	24.2 (0.05)				
S_0_ → T_1_^b^	9.5	9.7	10.8	9.9	9.9	10.8	10.1	10.1	10.8			
τ_T_/μs^c^			50			74			72			

a Oscillator strength, ; *n* = 1.49693; w = weak, m = middle, s = strong.

b Obsd. values: from sensitization, error ± 0.35 × 10^–3^ cm^–1^.

c Error ± 5 μs.

d ref (8).

### Crystal Packing

**Figure 5:**
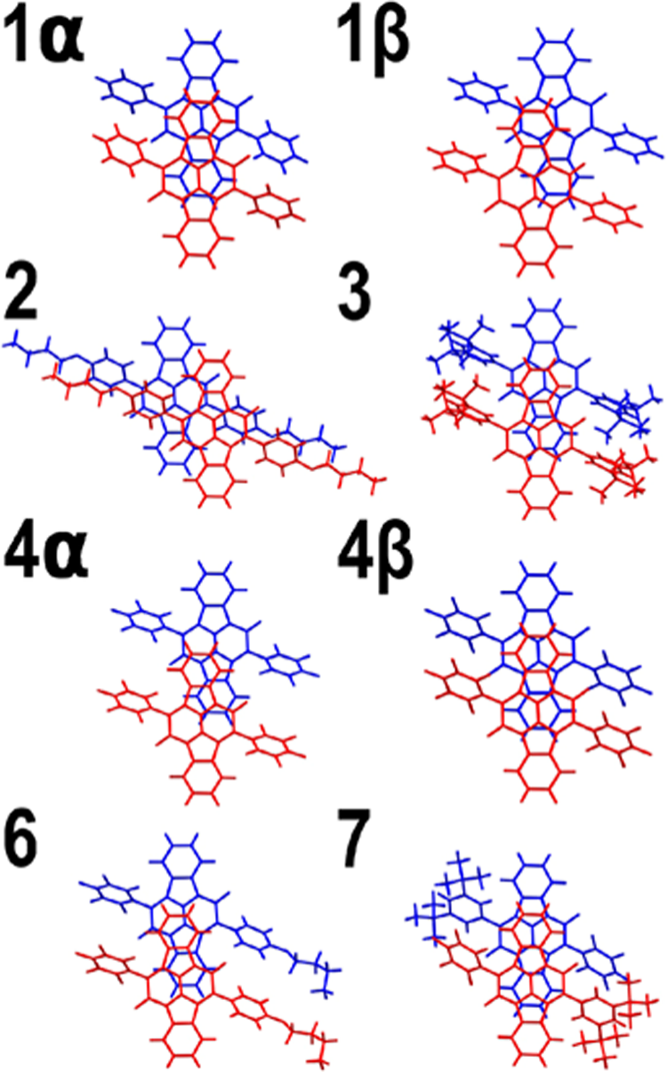
Most strongly interacting molecular pairs excised from single crystals.

The crystal structures are shown in Figures S3–S8, and the most strongly interacting molecular pairs excised out of them are collected in Figure 5. The latter were used to obtain a preliminary estimate of the suitability of the derivatives of **1** for singlet fission, using the program SIMPLE^31^ to calculate the rate constant *k*_SF_ for singlet fission for the solids **2**–**4**, **6**, and **7**. The calculated rates are not absolute but only relative to the singlet fission rate constant in a standard. We have used two such standards. One is the rate constant *k*_max_ calculated for the best pair geometry of **1**. The other is the largest rate constant *k*_0_ predicted previously^8^ by calculations for all pair geometries that were actually found in one of the two known^8^ polymorphs of **1**, *P*2_1_/*n*, referred to as **1α**, and *P*2_1_/*c*, called **1β**. The results of the computations are collected in Table 5.

**Table 5:** SIMPLE Results for Molecular Pairs Excised from Single-Crystal Structures (cf. Figure 5)^a^

no.	|T*|^2^^b^	|T**|^2^^c^	Δ*E*_DS_^d^	Δ*E* (S*)^e^	Δ*E* (S**)^f^	Δ*E*_BB_^g^	*k*_SF_/*k*_0_^h^	*k*_SF_/*k*_max_^i^
**3**	102	0	297	148	–149	11	4.25	0.11
**6**	30	2	253	123	–130	8	2.79	0.072
**1β**^j^	25	0	294	149	–145	3	1.00	0.026
**4α**	5	0	195	102	–93	3	0.80	0.021
**4β**	22	0	312	159	–153	6	0.68	0.016
**1α**^k^	14	0	349	175	–174	3	0.23	0.006
**7**	7	0	392	196	–196	2	0.06	0.001
**2**	1	0	270	123	–147	18	0.05	0.001

a All energies are in meV. 6-311G basis set, reorganization energy set at λ = 220 (ref (8)) energy of the charge-separated state chosen higher than that of the locally excited state by Δ*E*_CT_ = 1000.

b Squares of the electronic coupling elements for SF from the lower excitonic state S*.

c Squares of the electronic coupling elements for SF from the upper excitonic state S**.

d Absolute value of the energy difference between S* and S** (Davydov splitting).

e SF energy balance from the S* state, *E*(T_1_T_1_) – *E*(S*).

f SF energy balance from the S** state.

g Biexciton binding energy.

h Relative SF rate constant; *k*_0_ = 2.2 × 10^9^ s^–1^ is the largest value found for the best pair geometry actually present in **1β**.

i Relative SF rate constant; *k*_max_ = 8.4 × 10^10^ s^–1^ is the value SIMPLE found (ref (8)) for the best possible pair geometry of **1**.

j *P*2_1_/*c* form of **1** (**1β**).

k *P*2_1_/*n* form of **1** (**1α**).

## Discussion

The presently reported synthesis, crystal structure determination, and photophysical characterization of molecular photophysical properties represent necessary first steps in the planned examination of the suitability of solid thin layers of these cibalackrot derivatives for SF and further structural optimization directed toward the production of a practically useful material.

The roughly 10% yields in the syntheses of the new cibalackrots **2**–**7** are disappointing but provide enough material for measurements on the solids. If one or more of them are found to have promise in this regard, it will be necessary to develop better procedures. Possible routes forward would be the testing of a wider variety of solvents, search for catalysts, and introduction of solubilizing substituents. Such an effort and scale-up would be premature at this time because it is likely that further structural modification will be needed before a truly useful compound is found.

The agreement between calculated and observed molecular structures is good, except for the degree of rotation of the plane of the aryl substituent out of the plane of the indigoid core (Table 1). Instead of the calculated angle of ∼60° for the aryls difluorinated in the ortho positions and of ∼50° for the others, the equilibrium rotation angles of the aryl groups range from 40 to 80° apparently randomly, undoubtedly due to poorly understood packing forces.

The solution absorption spectra of the six substituted derivatives are all very similar to that of **1**, whose electronic excitations have been discussed in detail elsewhere.^8^ The visible region is dominated by an intense highest occupied molecular orbital (HOMO) to lowest unoccupied molecular orbital (LUMO) singlet excitation near 18,000 cm^–1^, which is slightly red shifted in **2** (Figure 3). This shift is reproduced by the TD-DFT calculations, which generally account for the spectra well. Perturbations in spectral energies, intensities, and S_1_ band shape introduced by the substituents used are minute, comparable to those introduced by a change in the solvent. Somewhat larger substitution effects are observed at higher energies, where contributions from several electronic transitions overlap.

The long fluorescence lifetime of about 6–7 ns and its high quantum yield, ∼80%, are good signs for singlet fission since they suggest that competing intramolecular deactivation processes are slow. Only **4** shows a yield reduced to 60%, presumably due to intersystem crossing, promoted by both the abundance of somewhat heavier atoms and the larger twist angle of its two C_6_F_5_ substituents.

The T_1_ state of **1** is also due to HOMO–LUMO excitation from the S_0_ state. All of the T_1_ excitation energies are equal within experimental uncertainty and at about 10,000 cm^–1^ are a little over half the S_1_ excitation energies. Slight endoergicity would actually be favorable for the efficiency of a singlet fission solar cell operating at room temperature. However, this promising picture is likely to change upon going to the solid state, as is already known for parent **1**.^8^ The triplet–triplet excitation spectra, important for the detection of singlet fission in solids, show a little more variation. The main peak is at 15,000–17,000 cm^–1^, unfortunately quite close to strong ground state absorption, which appears as a bleach in transient spectra. The overlap of the two reduces the accuracy with which the triplet–triplet absorption can be measured, and if it persists in the solid state, it will also make it harder to detect and study singlet fission.

In the solid, the relative singlet fission rate constants *k*_SF_ of the seven compounds (Table 5) should be determined primarily by the size of the squared singlet fission electronic matrix elements |T*|^2^ and |T**|^2^ of the states that result from Davydov splitting and by the increase Δ*E*(S*) in the endothermicity that results from this splitting. Calculations using the SIMPLE approximation^29^ predict that in these compounds, the stabilized lower exciton state S* couples much more strongly than the energetically higher state S**, |T*|^2^ ≫ |T**|^2^. Therefore, the magnitude of *k*_SF_ should be chiefly governed by the ratio of |T*|^2^ to Δ*E*(S*). The most promising structure **3** has roughly four times larger *k*_SF_ than the previously calculated **1β**. This value of *k*_SF_ is also only roughly nine times smaller than that of the previously calculated and published optimal structure of **1**. The large value is due to the roughly four times larger calculated squared SF matrix element |T*|^2^, while Δ*E*(S*) remains virtually the same as in **1β**. This shows a straightforward example of a stronger coupling leading to a larger predicted rate constant.

More interestingly, the structures of **4α** and **4β** provide an example of the importance of Davydov splitting. While the |T*|^2^ value in **4α** is 5 times smaller than in **1β** and is the lowest among the calculated molecular pairs, the predicted *k*_SF_ constant in **4α** is of comparable magnitude to **1β** due to a much smaller Δ*E*(S*) in **4α**. On the other hand, in **4β**, |T*|^2^ is almost the same as in **1β**, but Δ*E*(S*) is slightly larger than in **1β**. As a result, *k*_SF_ is considerably smaller in **4β** than in **1β**; moreover, it is even smaller than in **4α** despite having a much larger |T*|^2^. Finally, **7** suffers from both small |T*|^2^ and high Δ*E*(S*). In all cases, the calculated biexciton binding energy *E*_BB_ is very small and almost constant.

Figure 5 shows how far the crystal structures of **1**–**7** are from the optimal structures predicted for **1** and provides a qualitative rationale for the relatively small variation of Δ*E*(S*) within the group of the seven cibalackrots. A large component of this quantity is the Davydov splitting Δ*E*_DS_, which can be approximated by the dipole–dipole interaction of the transition moments of the S_0_ → S_1_ transitions on the two molecules. In both forms of crystalline **1**, the molecules in the most strongly interacting pair are stacked in parallel planes. Perfect eclipsing would provide the strongest dipolar splitting but is not observed. Instead, the two molecules are slip-stacked. We originally expected that bulky substituents on the phenyl groups might introduce a twisted arrangement. In fact, they do not. The structures of the dominant pairs are all alike, with the long axes of the two molecules parallel, and the substituents merely cause a variation in the degree and direction of the slip. Increasing slip along the long axis reduces the Davydov splitting as it gradually converts an H-type interaction to a J-type interaction, but the variation is not sufficient to have a significant effect. In **6** and **7**, one could have expected a stacking of the electron-poor perfluorinated phenyl substituent with its electron-rich hydrogenated counterpart, and this is indeed observed in **7**. However, the molecules still manage to keep their long axes parallel and avoid twisted packing that would affect the Davydov splitting fundamentally.

What ultimately matters for solar cell energy efficiency is not the absolute value of *k*_SF_ but its value relative to the rate constants of competing processes, which determines the biexciton quantum yield and the ability of the biexciton to dissociate into two independent triplets. In parent **1**, the most serious competing processes were the formation of excimers and charge-separated states, which were too stable to proceed to singlet fission and served as excitation traps. We are not aware of a simple theory that would provide estimates of the rates of these competitors as a function of crystal structure, but it is perhaps reasonable to assume that the more exoergic these processes are, the faster they will occur. Their exoergicity can be expected to correlate with the magnitude of Δ*E*(S*), providing us with an additional reason to look for crystal packing that yields small Δ*E*(S*).

It also needs to be recognized that the present discussion only deals with the first half of the singlet fission process, in which a singlet exciton is converted into a biexciton, in which two molecular triplets are bound to form an overall singlet. The second step, a dissociation of the biexciton into two freely diffusing triplets, proceeds through intermediates that offer additional opportunities for radiationless return to the ground state that lower the triplet quantum yields.

We must admit an initial defeat in our attempts to guess what kind of structural modification will lead to the desired crystal packing resembling the optimal structures previously calculated for **1**.^8^ We clearly have to turn to computer programs developed for crystal engineering and prediction of crystal structures from molecular structures, still a very imperfect art.

## Summary

Six new derivatives of cibalackrot (**1**) carrying substituents on its phenyl rings have been prepared, including three of a previously unavailable unsymmetrical structure. The crystal packing of five of them has been determined by single-crystal X-ray diffraction and used to predict the relative values of the rate constants for the conversion of singlet excitons into biexcitons, the first step of singlet fission. They are quite similar to the crystal packing in **1**, indicating that this will not be easily changed in a direction more favorable for singlet fission. The solution photophysical properties of all seven compounds are reported: singlet and triplet absorption spectra, fluorescence and its quantum yield and lifetime, and triplet excitation energies.

## Experimental Section

### Crystal Growth

Crystals of **3**, **4β**, **6**, and **7** were grown by slow diffusion of pentane (**3**, **4β**) or hexane (**6**, **7**) into a solution in DCM (**3**, 1 mg/1 mL), toluene (**6**, 1 mg/2 mL), or benzene (**4β**, saturated; **7**, 1 mg/2 mL) at room temperature. Crystals of **2** (1 mg) were grown by slow evaporation from (CHCl_3_–CCl_4_ 4:1; 3 mL). Crystals of **4α** were obtained by sublimation under 15 psi of argon in a 40 cm long glass tube (5 mm i.d.) whose sample containing end was inserted 30 cm deep into an oven kept for 2 h at 450 °C. Crystals grew in the room-temperature tube end. Single crystals for **2–7** were selected, mounted on MiTeGen loops with Paratone oil, and placed in an Oxford Cryosystems Cryostream 800 plus at *T* = 100 K at the Advanced Light Source. Data were collected for **2**, **4α**, **5**, **6**, and **7** on beamline 12.2.1 with λ = 0.7288 Å using a Bruker D8 diffractometer with a Bruker PHOTONII CPAD detector. Data for **3** and **4β** were collected on beamline 11.3.1 with λ = 0.7749 and 0.8856 Å, respectively, using a Bruker D8 diffractometer with a Bruker PHOTON100 CMOS detector and a Bruker PHOTONII CPAD detector, respectively. Data reduction was performed and corrected for Lorentz and polarization effects using SAINT^32^ v8.40a and was corrected for absorption effects and other effects using SADABS v2016/2^33^ for **2**, **4α**, **4β**, **6**, and **7** and TWINABS 2012/1^34^ for **3** and **5**. Structure solutions were performed by SHELXT^35^ using the dual space method and were refined by least-square refinement against F^2^ by SHELXL^36^

### Photophysics

Toluene for gas chromatography–electron capture detector (ECD) and flame ionization detector (FID) SupraSol and dichloromethane for spectroscopy Uvasol (Aldrich) were used without further purification. Ultraviolet–visible (UV–vis) spectra were measured with CARY 5000 (Agilent), and fluorescence spectra with FP-6600 (Jasco). Fluorescence quantum yields were measured in DCM solutions in 1 cm cells at an absorbance of 1.66 × 10^–2^ at the excitation wavelength (530 nm), using sulforhodamin B in ethanol as a reference (Φ_F_ = 66%).^37^ The standard correction^38,39^ was used for the different indices of refraction. Fluorescence lifetimes were measured with a Becker & Hickl SPC-130-EMN photon-counting PC card with a time resolution of 3 ps/channel and excitation at 450 nm (Coherent MIRA-HP laser, repetition rate 76 MHz, cut to ∼3.5 MHz). Emission was detected (Hamamatsu R3809U-50 microchannel plate photomultiplier) at 564 (**1**), 570 nm (**2**, **3**, **4**, **5**, **7**), and 574 nm (**6**).

Transient absorption experiments were carried out using a commercially available apparatus EOS Fire transient absorption spectrometer (Ultrafast systems, Sarasota, FL) combined with a pump laser NT242 (Ekspla, Vilnius, Lithuania), with an OPO-based system at 1 kHz repetition rate, tunable from 210 to 2600 nm. The pulse duration was 5 ns, and the energy of the pulse was 0.3–0.4 μJ at the sample position. The probe light source was a subnanosecond pulsed photonic crystal fiber-based supercontinuum laser with a spectral range of 350–950 nm. The spectrometer used a linear array detector with a CMOS sensor (1024 pixels). The sample in freeze–pump–thaw degassing cuvette with a 2 mm path length was randomly moved at 1 mm/s speed through the measurement. The stability of the sample was verified by recording steady-state absorption spectra before and after each measurement.

### Calculations

Optimized geometries were obtained at the M06HF-GD3/CC-PVDZ level (CPCM, CH_2_Cl_2_), and subsequent TD B3LYP-GD3/CC-PVTZ-TD (CPCM, CH_2_Cl_2_) calculations were done for 20 lowest states. Aryl twist angles were also calculated at the CAM-B3LYP-D3 level, and the results were identical within 3°.

Calculations of activation energies for conformer interconversion (Table 2) were done with the Gaussian 16 (rev. A.03) code.^40^ S_0_ and S_1_ minima were optimized without constraints. Barriers for the interconversion between the *C*_2_ and *C*_*i*_ minima were estimated by constraining the angle of rotation of a single aryl group to 90 and 0° with respect to the indigoid plane and performing a relaxed optimization. All calculations used the (TD) B3LYP-D3/CC-PVTZ level of theory,^41−43^ while S_1_ optimizations were performed for 6 electronic states.

The computational prediction of *k*_SF_ of **1α**, **1β**, and **2**–**7** was made using the program SIMPLE (ver. 3.0),^8^ using the 6-311G basis set and assuming the energy gap between the locally excited state and charge transfer state Δ*E*_CT_ to be 1 eV. The expansion coefficients of natural atomic orbitals in terms of contracted Gaussians were obtained via Weinhold′s NBO analysis (ver. 7.0)^44^ and an SCF wavefunction provided by Gaussian 16 (rev. C.01).^45^ The biexciton formation reorganization energy λ that enters the Marcus equation was calculated using the modified^46^ Nelsen^47^ four-point formula, λ = *E*(S_1_) + *E*(S_0_) – 2*E*(T_1_), where *E*(X) is the energy of the T_1_ state at the equilibrium geometry of state X. The energies and optimized geometries were obtained using Gaussian 09 (rev. D.01) at the B3LYP/6-311G level. The reorganization energy λ is 0.22 eV.

### Synthesis

#### Procedures

Reactions were carried out under a nitrogen atmosphere with anhydrous solvents freshly distilled under anhydrous conditions unless otherwise noted. Reagents were used as supplied unless otherwise stated. Standard Schlenk and vacuum line techniques were employed for all manipulations of air or moisture-sensitive compounds. Yields refer to isolated, chromatographically, and spectroscopically homogenous materials unless otherwise stated.

Analytical thin-layer chromatography (TLC) was performed using precoated TLC aluminum sheets (Silica gel 60 F254). TLC spots were visualized using 254 nm light. Column chromatography was performed using silica gel (high purity grade, pore size 60 Å, 70–230 mesh). Melting points are reported uncorrected. Infrared spectra (IR) were recorded in KBr pellets. UV–vis spectra were recorded in dichloromethane from 200 to 800 nm in 1 cm cells. Chemical shifts in ^1^H, ^2^H, and ^13^C spectra are reported in ppm. Proton and carbon spectra were recorded in CD_2_Cl_2_, CDCl_3_, Cl_2_CDCDCl_2_, or DMSO-*d*_6_, and ^2^H spectra were recorded in 1,1,2,2-tetrachloroethane. Chemical shift values for protons are referenced to the residual proton resonance of CD_2_Cl_2_ (5.32 ppm), CDCl_3_ (7.26 ppm), Cl_2_CDCDCl_2_ (6.00 ppm), and DMSO-*d*_6_ (2.50 ppm). Chemical shift values for deuterons are referenced to Cl_2_CDCDCl_2_. Chemical shift values for carbons are referenced to the carbon resonance of CD_2_Cl_2_ (54.0 ppm), CDCl_3_ (77.2 ppm), Cl_2_CDCDCl_2_ (74.2 ppm), and DMSO-*d*_*6*_ (39.5 ppm). Splitting patterns are assigned as follows: s, singlet; d, doublet; t, triplet; m, multiplet; and br, broad signal. Structural assignments were made with additional information from gCOSY, gHSQC, and gHMBC measurements. High-resolution mass spectra (HRMS) were obtained using electrospray ionization (ESI) and atmospheric pressure chemical ionization (APCI) with a mass analyzer combining a linear ion trap and an Orbitrap.

##### 4-Butoxyphenylacetyl Chloride (**9**)^48^

A mixture of 4-butoxyphenylacetic acid (2.0 g, 9.60 mmol) and thionyl chloride (4.9 mL, 67 mmol) was refluxed (oil bath) for 2 h (CaCl_2_ tube). Excess thionyl chloride was removed under reduced pressure, and the crude acyl chloride was purified by kugelrohr distillation (170 °C, 0.5 Torr). The pure product was obtained as a colorless viscous liquid (1.85 g, 85%). ^1^H NMR (400 MHz, CDCl_3_): δ 7.17 (m, 2H, ArH), 6.89 (m, 2H, ArH), 4.07 (s, 2H, ArCH_2_CO), 3.96 (t, *J* = 6.5 Hz, 2H, OCH_2_CH_2_CH_2_CH_3_), 1.77 (m, 2H, OCH_2_CH_2_CH_2_CH_3_), 1.49 (m, 2H, OCH_2_CH_2_CH_2_CH_3_), 0.90 (t, *J* = 11.0 Hz, 3H, OCH_2_CH_2_CH_2_CH_3_).

##### 3,5-Di-*tert*-butylphenylacetyl Chloride (**10**)^49^

A mixture of 3,5-di-*tert*-butylphenylacetic acid (2.0 g, 8.1 mmol) and thionyl chloride (2.9 mL, 40.0 mmol) was refluxed (oil bath) for 2 h (CaCl_2_ tube). Excess thionyl chloride was removed under reduced pressure, and the crude acyl chloride was purified by Kugelrohr distillation (160 °C, 0.5 Torr). The pure product was obtained as a pale yellow viscous liquid (1.7 g, 79%). ^1^H NMR (400 MHz, CDCl_3_): δ 7.39 (t, *J* = 2.0 Hz, 1H, ArH), 7.09 (d, *J* = 1.8 Hz, 2H, ArH), 4.13 (brs, 2H, −CH_2_−), 1.32 (s, 18H, *^t^*Bu).

##### Pentafluorophenylacetyl Chloride (**11**)^50^

A mixture of pentafluorophenylacetic acid (2.0 g, 8.9 mmol) and thionyl chloride (3.85 mL, 53.0 mmol) was refluxed (oil bath) for 2 h (CaCl_2_ tube). Excess thionyl chloride was removed under reduced pressure. The resulting slightly pink product was sufficiently pure for further reaction (2.0 g, 91%). ^1^H NMR (400 MHz, CDCl_3_): δ 4.26 (s, 2H, −CH_2_−). ^19^F NMR (376 MHz, CDCl_3_) δ −141.43 (m, 2F), −152.40 (t, *J* = 21.0 Hz, 1F), −161.05 to −160.84 (m, 2F).

##### 7-(Pentafluorophenyl)-6*H*-pyrido[1,2-*a*:3,4-*b*′]diindole-6,13(12*H*)-dione (**12**)

***Caution: 1,1,2,2-tetrachloroethane is a very toxic solvent. Work with this chemical must be carried out under very strictly observed safety conditions.*** A solution of **11** (5.1 g, 21.0 mmol) was added to a suspension of indigo (**8**, 2.5 g, 9.5 mmol) in anhydrous 1,1,2,2-tetrachloroethane (20 mL). The mixture was brought to reflux and left to stir in a nitrogen atmosphere for 41 h. The solvent was removed under reduced pressure, and the residue was purified by column chromatography on silica gel using CH_2_Cl_2_ and CH_2_Cl_2_—ethyl acetate 100:1, 50:1, 20:1, and 10:1 (v/v) as eluents. The almost pure cibalackrot was triturated with ethanol (3 × 40 mL) and pentane (1 × 40 mL). The product was obtained as a purple powder (385 mg, 9%). Mp: >300 °C. ^1^H NMR (400 MHz, DMSO-*d*_6_): δ 11.93 (br s, 1H, NH), 8.57 (td, *J*_1_ = 8.1 Hz, *J*_2_ = 0.8 Hz, 1H, ArH), 7.86 (m, 1H, ArH), 7.76 (m, 1H, ArH), 7.56 (m, 1H, ArH), 7.44 (m, 2H, ArH), 7.26 (d, *J*_1_ = 8.0 Hz, 1H, ArH), 7.02 (m, 1H, ArH). ^19^F NMR (376 MHz, DMSO-*d*_6_): δ −138.77 (m, 2F), −152.12 (m, 1F), −161.47 (m, 2F). ^13^C{^1^H}NMR 126 MHz, DMSO-*d*_6_: δ 180.8, 154.7, 148.3, 146.3, 144.1 (m, *J*_CF_ = 246.3 Hz), 141.6, 141.5 (m, *J*_CF_ = 253.7 Hz), 137.7 (m, *J*_CF_ = 249.3 Hz), 136.0, 133.2, 127.5, 126.7, 124.6, 124.1, 124.0, 121.6, 118.1, 117.6, 116.5, 116.0, 108.2. IR (KBr): 3347, 1696, 1645, 1631, 1615, 1593, 1521, 1493, 1459, 1303, 1179, 1143, 1020, 988, 973, 750, 687 cm^–1^. UV–vis (CH_2_Cl_2_) λ_max_ (ε): 228 (3.5 × 10^4^), 277 (3.9 × 10^4^), 318 (1.7 × 10^4^), 517 (1.2 × 10^4^), 547 (1.6 × 10^4^) nm (M^–1^ cm^–1^). MS (ESI) [M + H]^+^*m*/*z* 453.1. HRMS (ESI) *m*/*z*: [M + H]^+^ calcd for C_24_H_10_O_2_N_2_F_5_ 453.0657; Found 453.0651. Calcd for C_24_H_9_F_5_N_2_O_2_: C, 63.73; H, 2.01; N, 6.19. Found: C, 64.01; H, 2.34; N, 6.10.

##### 7-(4-Butoxyphenyl)-6*H*-pyrido[1,2-*a*:3,4-*b*′]diindole-6,13(12*H*)-dione (**13**)

A solution of freshly distilled 4-butoxylphenylacetyl chloride (**9**, 1.8 g, 7.9 mmol) in an anhydrous isomer mixture of xylenes (5 mL) was added dropwise to the refluxing solution/suspension of indigo (**8**, 393 mg, 1.5 mmol) in the anhydrous isomer mixture of xylenes (20 mL) over a period of 40 min. The dark blue/purple reaction mixture was refluxed (oil bath temperature 170 °C) for an additional 4 h and then stirred for 18 h at 140 °C. The reaction mixture turned red during this time. The heating was stopped, and volatiles were removed under reduced pressure. The dark red honey-like residue was carefully triturated with hexane (8 × 5 mL) and then purified by column chromatography on silica gel (hexane/THF, 2:1, then pure THF). Compound **13** was obtained as a purple crystalline solid (248 mg; 38%). Mp: 337–343 °C. ^1^H NMR (400 MHz, THF-*d*_8_): δ 10.92 (brs, 1H, NH), 8.74–8.76 (m, 1H, ArH), 7.79–7.81 (m, 1H, ArH), 7.64–7.67 (m, 1H, ArH), 7.55–7.56 (m, 2H, ArH), 7.33–7.42 (m, 3H, ArH), 7.30–7.31 (m, 1H, ArH), 7.10–7.12 (m, 2H, ArH), 6.87–6.90 (m, 1H, ArH), 4.12 (t, *J* = 6.4 Hz, 2H, OCH_2_CH_2_CH_2_CH_3_), 1.85–1.89 (m, 2H, OCH_2_CH_2_CH_2_CH_3_), 1.58–1.63 (m, 2H, OCH_2_CH_2_CH_2_CH_3_) 1.06 (t, *J* = 7.4 Hz, 3H, OCH_2_CH_2_CH_2_CH_3_). ^13^C{^1^H}NMR (100 MHz, THF-*d*_8_): δ 180.0, 160.0, 156.9, 147.6, 146.8, 137.2, 134.8, 134.7, 131.4, 131.0, 129.6, 125.8, 125.5, 124.5, 123.0, 120.6, 120.4, 117.9, 114.8, 113.9, 111.5, 67.3, 31.4, 19.3, 13.3. IR (KBr): 3201, 3127, 2960, 2933, 2872, 1699, 1642, 1625, 1614, 1605, 1590, 1510, 1489, 1473, 1457, 1410, 1397, 1344, 1330, 1305, 1293, 1246, 1229, 1178, 1159, 1126, 1104, 1087, 1034, 1007, 974, 863, 840, 816, 783, 759, 751, 707, 688, 654, 635, 623, 559, 540, 531 cm^–1^. UV–vis (CH_2_Cl_2_) λ_max_ (ε): 229 (4.0 × 10^4^), 276 (3.6 × 10^4^), 314 (1.5 × 10^4^), 516 (1.5 × 10^4^), 545 (1.9 × 10^4^) nm (M^–1^ cm^–1^). MS, *m*/*z* (%): 435.2 (100, M + H). HRMS (ESI) *m*/*z*: [M + H]^+^ calcd for C_28_H_23_N_2_O_3_ 435.1703; found 435.1704. Anal. calcd for C_28_H_22_N_2_O_3_: C, 77.40; H, 5.10; N, 6.45. Found: C, 77.23; H, 5.13; N, 6.48.

##### 7,14-Bis(4-butoxyphenyl)diindolo[3,2,1-de:3′,2′,1′-ij][1,5]naphthyridine-6,13-dione (**2**; Method A)

Indigo (**8**, 190 mg, 0.7 mmol) was suspended in an anhydrous isomer mixture of xylenes (120 mL). Freshly prepared 4-butoxyphenylacetyl chloride (**9**, 1.85 g, 8.2 mmol) was added to the reaction mixture as a solution in the anhydrous isomer mixture of xylenes (5 mL), and the resulting mixture was brought to reflux (oil bath) and left to stir in a nitrogen atmosphere for 72 h. The reaction mixture was left to cool to rt and evaporated to dryness. The residue was triturated with hexane (2 × 40 mL) and then with ethyl acetate (2 × 40 mL). The product was identified by the ^1^H NMR spectrum as a mixture of the desired product **2** and 7-(4-butoxyphenyl)-6*H*-pyrido[1,2-a:3,4-b′]diindole-6,13(12*H*)-dione **13** in a 1:4 ratio (70 mg). The mixture (70 mg) was suspended in anhydrous 1,1,2,2-tetrachloroethane (50 mL). Freshly prepared 4-butoxyphenylacetyl chloride (**9**, 0.75 g, 3.3 mmol) was added to the reaction mixture as a solution in anhydrous 1,1,2,2-tetrachloroethane (5 mL), and the resulting mixture was brought to reflux (oil bath) and left to stir in a nitrogen atmosphere for 110 h. The solvent was removed under reduced pressure, and the residue was triturated with ethyl acetate (2 × 40 mL). The desired product was obtained as a purple powder (25 mg, 6%).

##### 7,14-Bis(4-butoxyphenyl)diindolo[3,2,1-de:3′,2′,1′-ij][1,5]naphthyridine-6,13-dione (**2**; Method B)

***Caution: 1,1,2,2-tetrachloroethane is a very toxic solvent. Work with this chemical must be carried out under very strictly observed safety conditions**.* Indigo (**8**, 242 mg, 0.9 mmol) was suspended in anhydrous 1,1,2,2-tetrachloroethane (40 mL). Freshly prepared 4-butoxyphenylacetyl chloride **9** (2.5 g; 11 mmol) was added as a solution in anhydrous 1,1,2,2-tetrachloroethane (10 mL), and the resulting mixture was brought to reflux (oil bath) and left to stir in a nitrogen atmosphere for 94 h. The solvent was removed under reduced pressure, and the residue was triturated with ethyl acetate (3 × 35 mL). The crude product was purified by column chromatography on silica gel using CH_2_Cl_2_ and CH_2_Cl_2_–ethyl acetate 100:1 (v/v) as eluents. The product was triturated with ethyl acetate (20 mL). The product was obtained as a purple powder (57 mg, 10%). Mp: >300 °C. ^1^H NMR (400 MHz, Cl_2_CDCDCl_2_): δ 8.49 (d, *J* = 8.1 Hz, 2H, ArH), 7.69 (m, 2H, ArH), 7.69 (m, 4H, *J*_AX_ = *J*_A′X′_ = 8.3 Hz, *J*_AA′_ = 2.5 Hz, *J*_AX′_ = *J*_A′X_ = 0.4 Hz, ArH), 7.59 (m, 2H, ArH), 7.26 (m, 2H, ArH), 7.13 (m, 4H, *J*_XA_ = *J*_X′A′_ = 8.3 Hz, *J*_XX′_ = 2.5 Hz, *J*_XA′_ = *J*_X′A_ = 0.4 Hz, ArH), 4.11 (t, *J* = 6.5 Hz, 4H, OCH_2_CH_2_CH_2_CH_3_), 1.57 (m, 4H, OCH_2_CH_2_CH_2_CH_3_), 1.49 (m, 4H, OCH_2_CH_2_CH_2_CH_3_), 1.04 (t, *J* = 7.4 Hz, 6H, OCH_2_CH_2_CH_2_CH_3_). ^13^C{^1^H}NMR (126 MHz, Cl_2_CDCDCl_2_) δ 160.2, 159.9, 144.7, 132.3, 132.0, 131.6, 131.0, 126.4, 126.1, 125.7, 125.7, 122.2, 117.8, 114.9, 68.3, 31.6, 19.6, 14.3. IR (KBr): 3071, 3037, 2951, 2936, 2870, 1632, 1600, 1573, 1508, 1249, 1072, 986, 793, 796, 762 cm^–1^. UV–vis (CH_2_Cl_2_) λ_max_ (ε): 279 (4.7 × 10^4^), 382 (4.4 × 10^3^), 483 (1.2 × 10^4^), 518 (2.6 × 10^4^), 552 (3.5 × 10^4^) nm (M^–1^ cm^–1^). MS (APCI) [M + H]^+^ m/z 607.3. HRMS (APCI) *m*/*z*: [M + H]^+^ calcd for C_40_H_35_O_4_N_2_ 607.2591; found 607.2590. Anal. for 4 × C_40_H_34_N_2_O_4_: 1 × CH_2_Cl_2_: calcd C, 76.99; H, 5.54; N, 4.46. Found: C, 76.81; H, 5.63; N, 4.23.

##### 1,8-Bis(3,5-di-*tert*-butylphenyl)diindolo[3,2,1-de:3′,2′,1′-ij][1,5]naphthyridine-2,9-dione (**3**)

The solution of freshly distilled 3,5-di-*tert*-butylphenylacetyl chloride **10** (1.75 g, 6.6 mmol) in an anhydrous isomer mixture of xylenes (5 mL) was added dropwise to the refluxing solution/suspension of indigo (**8**, 393 mg, 1.5 mmol) in the anhydrous isomer mixture of xylenes (25 mL) over a period of 30 min. The dark blue/purple reaction mixture was refluxed at the temperature of the oil bath (170 °C) for an additional 6 h and then stirred for 16 h at 140 °C. The reaction mixture turned red during this time. The heating was stopped, and volatiles were removed under reduced pressure. The dark red honey-like residue was carefully triturated with hexane (5 × 5 mL) and then purified by column chromatography on silica gel (hexane—CH_2_Cl_2_, 1:4). Cibalackrot derivative **3** was obtained as a deep red crystalline material (101 mg, 10%). Mp: >320 °C (dec.). ^1^H NMR (500 MHz, CDCl_3_): δ 8.53–8.54 (m, 2H, ArH), 7.59–7.60 (m, 2H, ArH), 7.57–7.58 (m, 6H, ArH), 7.52–7.55 (m, 2H, ArH), 7.17–7.20 (m, 2H, ArH), 1.42 (s, 36H, *^t^*Bu). ^13^C{^1^H}NMR (100 MHz, CDCl_3_): δ 159.6, 150.6, 132.6, 144.7, 132.2, 131.9, 131.8, 126.0, 125.6, 125.5, 124.6, 123.1, 122.1, 117.7, 35.1, 31.5. IR (KBr): 3066, 2960, 2903, 2866, 1639, 1605, 1594, 1576, 1482, 1461, 1442, 1410, 1392, 1384, 1363, 1312, 1271, 1237, 1203, 1163, 1079, 1015, 908, 901, 894, 880, 875, 839, 805, 780, 765, 757, 751, 745, 733, 708, 656 cm^–1^. UV–vis (CH_2_Cl_2_) λ_max_ (ε): 277 (5.1 × 10^4^), 369 (5.9 × 10^3^), 476 (1.1 × 10^4^), 508 (2.6 × 10^4^), 543 (3.6 × 10^4^) nm (M^–1^ cm^–1^). UV–vis (CH_2_Cl_2_) λ_max_ (ε): 277 (5.1 × 10^4^); 369 (5.9 × 10^3^); 476 (1.1 × 10^4^); 508 (2.6 × 10^4^); 543 (3.6 × 10^4^) nm (M^–1^ cm^–1^). MS, *m*/*z* (%): 687.4 (100, M + H). HRMS (APCI) *m*/*z*: [M + H]^+^ calcd for C_48_H_51_N_2_O_2_ 687.3945; found 687.3944. Anal. calcd for C_48_H_50_N_2_O_2_ + CDCl_3_ in 1:1 ratio: C, 72.90; H, 6.49; N, 3.47. Found: C, 72.85; H, 6.18; N, 3.73.

##### 7,14-Bis(pentafluorophenyl)diindolo[3,2,1-de:3′,2′,1′-ij][1,5]naphthyridine-6,13-dione (**4**; Method A)

A solution of pentafluorophenylacetyl chloride (**11**, 2.0 g, 8.2 mmol) in an anhydrous isomer mixture of xylenes (20 mL) was added to indigo (**8**, 211 mg, 0.8 mmol), and the resulting mixture was refluxed (oil bath) in a nitrogen atmosphere for 22 h. The solvents were removed under reduced pressure, and the residue was triturated with hexane (4 × 25 mL). The isolated solid was purified by column chromatography on silica gel using CH_2_Cl_2_ and CH_2_Cl_2_—ethyl acetate 100:1 and 50:1 (v/v) as eluents to yield 7-(pentafluorophenyl)-6*H*-pyrido[1,2-a:3,4-b′]diindole-6,13(12*H*)-dione (**12**), identified by ^1^H NMR. Freshly prepared pentafluorophenylacetyl chloride (**11**, 1.4 g, 5.7 mmol) was dissolved in the anhydrous isomer mixture of xylenes (3 mL), and the resulting solution was added to 7-(pentafluorophenyl)-6*H*-pyrido[1,2-a:3,4-b′]diindole-6,13(12*H*)-dione (**12**). The reaction mixture was refluxed (oil bath) in a nitrogen atmosphere for 72 h. The solvents were removed under reduced pressure, and the resulting mixture was purified by column chromatography on silica gel using CH_2_Cl_2_ and CH_2_Cl_2_–ethyl acetate 100:1 (v/v) as eluents. The product was obtained as a purple powder (46 mg, 9%).

##### 7,14-Bis(pentafluorophenyl)diindolo[3,2,1-de:3′,2′,1′-ij][1,5]naphthyridine-6,13-dione (**4**; Method B)

***Caution: 1,1,2,2-tetrachloroethane is a very toxic solvent. Work with this chemical must be carried out under very strictly observed safety conditions.*** Indigo (**8**, 475 mg; 1.8 mmol) was suspended in excess pentafluorophenylacetyl chloride (**11**, 5.0 g; 20.0 mmol). The reaction mixture was heated to 155°C (oil bath) and stirred in a nitrogen atmosphere for 22 h. Anhydrous 1,1,2,2-tetrachloroethane (5 mL) was added and the resulting mixture was refluxed (oil bath) in a nitrogen atmosphere for 48 h. The solvent was removed under reduced pressure, and the resulting oil was purified by column chromatography on silica gel using CH_2_Cl_2_–hexane 3:1, 5:1, 8:1, and 1:0 (v/v) as eluents. The product was triturated with ethanol (20 mL) and hexane (20 mL). The product was obtained as a purple powder (220 mg, 19%). Mp: >300 °C. ^1^H NMR (400 MHz, Cl_2_CDCDCl_2_): δ 8.44 (m, 2H, ArH), 7.69 (m, 2H, ArH), 7.38 (m, 4H, ArH). ^19^F NMR (376 MHz, Cl_2_CDCDCl_2_): δ −136.36 (m, 4F), −151.13 (t, *J* = 21 Hz, 2F), −160.47 (m, 4F). ^13^C{^1^H}NMR (126 MHz, Cl_2_CDCDCl_2_) δ 157.1, 145.1, 145.2 (d, *J*_CF_ = 250.2 Hz), 142.4 (m, *J*_CF_ = 253.4 Hz), 138.3 (dt, ^*1*^*J*_CF_ = 249.1 Hz, ^*2*^*J*_CF_ = 15.1 Hz), 136.6, 134.1, 127.5, 125.7, 124.3, 123.1, 118.3, 116.5, 108.4. IR (KBr): 1643, 1608, 1578, 1556, 1482; 1520, 1496, 990 cm^–1^. UV–vis (CH_2_Cl_2_) λ_max_ (ε): 274 (4.3 × 10^4^), 362 (7.5 × 10^3^), 372 (9.1 × 10^3^), 500 (2.3 × 10^4^), 538 (3.5 × 10^4^) nm (M^–1^ cm^–1^). MS (ESI) [M + H]^+^*m*/*z* 643.2, [M + Na]^+^*m*/*z* 665.2. HRMS (ESI) *m*/*z*: [M + H]^+^ calcd for C_32_H_9_O_2_N_2_F_10_ 643.0499; found 643.0505. Anal. for 3 × C_32_H_8_F_10_N_2_O_2_: 2 × H_2_O: calcd C, 58.73; H, 1.44; N, 4.28. Found: C, 58.51; H, 1.57; N, 4.33.

##### 8-(4-Butoxyphenyl)-1-(3,5-di-*tert*-butylphenyl)diindolo[3,2,1-de:3′,2′,1′-ij][1,5]naphthyridine-2,9-dione (**5**)

***Caution: 1,1,2,2-tetrachloroethane is a very toxic solvent. Work with this chemical must be carried out under very strictly observed safety conditions.*** Compound **13** (200 mg, 4.6 mmol) was suspended in freshly distilled 3,5-di-*tert*-butylphenylacetyl chloride (**10**, 4.7 g, 18.0 mmol), and the purple reaction mixture was stirred for 16 h at 160 °C (heated in an oil bath). Subsequently, anhydrous 1,1,2,2-tetrachloroethane (5 mL) was added at the same temperature. All solids dissolved, and the deep red reaction mixture was stirred for an additional 8 h at 180 °C. The heating was stopped, and the reaction mixture was allowed to cool to room temperature. 1,1,2,2-Tetrachloroethane was then distilled off under reduced pressure, and the remaining volatiles were removed in a Kugelrohr distillation apparatus (190 °C, 0.6 Torr). The dark red honey-like residue was carefully triturated with hexane (3 × 5 mL) and then purified by column chromatography on silica gel (CH_2_Cl_2_). Compound **5** was obtained as a deep red crystalline solid (160 mg, 54%). Mp: 326–330 °C. ^1^H NMR (500 MHz, CDCl_3_): δ 8.52–8.56 (m, 1H, ArH), 8.48–8.52 (m, 1H, ArH), 7.65–7.72 (m, 1H, ArH), 7.65–7.72 (m, 2H, *J*_AX_ = *J*_A′X′_*=* 8.2 Hz, *J*_AA′_*=* 2.7 Hz, *J*_AX′_ = *J*_A′X_*=* 0.4 Hz, ArH), 7.51–7.61 (m, 6H, ArH), 7.15–7.2 (m, 2H, ArH), 7.08–7.10 (m, 2H, *J*_XA_ = *J*_X′A′_*=* 8.2 Hz, *J*_XX′_*=* 2.7 Hz, *J*_XA′_ = *J*_X′A_*=* 0.4 Hz, ArH), 4.09 (t, *J* = 6.5 Hz, 2H, OCH_2_CH_2_CH_2_CH_3_), 1.81–1.89 (m, 2H, OCH_2_CH_2_CH_2_CH_3_), 1.53–1.59 (m, 2H, OCH_2_CH_2_CH_2_CH_3_), 1.03 (t, *J* = 7.4 Hz, 3H, OCH_2_CH_2_CH_2_CH_3_). ^13^C{^1^H}NMR (100 MHz, CDCl_3_): δ 159.9, 159.7, 159.6, 150.5, 144.6, 132.5, 132.2, 131.84, 131.80, 131.6, 131.3, 130.8, 126.0, 125.9, 125.7, 125.53, 125.50, 125.3, 124.6, 123.1, 122.1, 121.9, 117.64, 117.57, 114.4, 67.8, 35.1, 31.5, 31.3, 19.3, 13.9. IR (KBr): 3071, 2959, 2870, 1634, 1603, 1572, 1540, 1511, 1481, 1442, 1410, 1394, 1382, 1362, 1313, 1298, 1271, 1246, 1780, 1164, 1121, 1076, 1017, 979, 896, 885, 837, 807, 781, 774, 758, 711, 663, 635, 629, 594 cm^–1^. UV–vis (CH_2_Cl_2_) λ_max_ (ε): 278 (5.1 × 10^4^), 376 (5.3 × 10^3^), 383 (1.3 × 10^4^), 513 (2.8 × 10^4^), 549 (3.8 × 10^4^) nm (M^–1^ cm^–1^). MS, *m*/*z* (%): 647.3 (100, M + H). HRMS (APCI) *m*/*z*: [M + H]^+^ calcd for C_44_H_43_N_2_O_3_ 647.3268; found 647.3269. Anal. calcd for C_44_H_42_N_2_O_3_: C, 81.70; H, 6.55; N, 4.33. Found: C, 81.55; H, 6.54; N, 4.17.

##### 7-(4-Butoxyphenyl)-14-(pentafluorophenyl)diindolo[3,2,1-de:3′,2′,1′-ij][1,5]naphthyridine-6,13-dione (**6**; Method A)

A solution of 4-butoxyphenylacetyl chloride (**9**, 0.367 mg, 1.5 mmol) in an anhydrous isomer mixture of xylenes (10 mL) was added to 7-(pentafluorophenyl)-12,13-dihydro-6H-pyrido[1,2-a:3,4-b′]diindol-6-one (**12**, 109 mg, 0.2 mmol) and refluxed (oil bath) in a nitrogen atmosphere for 20 h. The solvents were removed under reduced pressure, and the residue was triturated with hexane (2 × 20 mL). The resulting solid was purified by column chromatography on silica gel using dichloromethane as an eluent. The pure product was obtained after subsequent trituration in ethanol (2 × 20 mL) as a purple powder (50 mg, 33%).

##### 7-(4-Butoxyphenyl)-14-(pentafluorophenyl)diindolo[3,2,1-de:3′,2′,1′-ij][1,5]naphthyridine-6,13-dione (**6**; Method B)

7-(4-Butoxyphenyl)-12,13-dihydro-6*H*-pyrido[1,2-a:3,4-b′]diindol-6-one (**13**, 186 mg, 0.4 mmol) was suspended in an anhydrous isomer mixture of xylenes (5 mL). Freshly prepared pentafluorophenylacetyl chloride (**11**, 825 mg; 3.4 mmol) was added to the reaction mixture as a solution in the anhydrous isomer mixture of xylenes (10 mL), and the resulting mixture was refluxed (oil bath) in a nitrogen atmosphere for 21 h. The solvents were removed under reduced pressure, and the solid was triturated with hexane (3 × 40 mL). The resulting solid was purified by column chromatography on silica gel using CH_2_Cl_2_ as an eluent. The pure product was a purple powder (175 mg, 65%). Mp: >300 °C. ^1^H NMR (600 MHz, Cl_2_CDCDCl_2_): δ 8.47 (m, 2H, ArH), 7.71 (m, 1H, ArH), 7.69 (m, 2H, *J*_AX_ = *J*_A′X′_*=* 8.4 Hz, *J*_AA′_*=* 2.5 Hz, *J*_AX′_ = *J*_A′X_*=* 0.5 Hz, ArH), 7.66 (m, 1H, ArH), 7.60 (m, 1H, ArH), 7.35 (m, 2H, ArH), 7.29 (m, 1H, ArH), 7.14 (m, 2H, *J*_XA_ = *J*_X′A′_*=* 8.4 Hz, *J*_XX′_*=* 2.5 Hz, *J*_XA′_ = *J*_X′A_*=* 0.5 Hz, ArH), 4.11 (t, *J* = 6.5 Hz, 2H, OCH_2_CH_2_CH_2_CH_3_), 1.86 (m, 2H, OCH_2_CH_2_CH_2_CH_3_), 1.56 (m, 2H, OCH_2_CH_2_CH_2_CH_3_), 1.04 (t, *J* = 7.4 Hz, 3H, OCH_2_CH_2_CH_2_CH_3_). ^19^F NMR (470 MHz, Cl_2_CDCDCl_2_): δ −136.70 (dd, *J*_1_ = 22 Hz, *J*_2_ = 6 Hz, 2F), -146.17 (t, *J* = 21 Hz, 1F), −154.97 (m, 2F). ^13^C{^1^H}NMR (151 MHz, Cl_2_CDCDCl_2_) δ 160.7, 159.5, 157.6, 145.1 (d, *J*_CF_ = 254.1 Hz), 145.0, 144.6, 142.1 (m, ^1^*J*_CF_ = 256 Hz), 138.2 (dt, ^1^*J*_CF_ = 253 Hz, ^2^*J*_CF_ = 13.0 Hz), 136.0, 134.3, 133.6, 132.6, 132.0, 131.7, 126.9, 125.8, 125.7, 125.4, 125.1, 124.8, 124.6, 120.9, 118.0, 115.0, 112.9, 109.0 (m), 68.3, 31.6, 19.6, 14.33. IR (KBr) 3064, 2935, 2874, 1634, 1604, 1575, 1524, 1500, 1249, 1082, 990, 980, 793, 758 cm^–1^. UV–vis (CH_2_Cl_2_) λ_max_ (ε): 275 (4.6 × 10^4^), 358 (4.8 × 10^3^), 476 (1.1 × 10^4^), 510 (2.7 × 10^4^), 549 (3.8 × 10^4^) nm (M^–1^ cm^–1^). MS (APCI) [M + H]^+^*m*/*z* 625.2. HRMS (ESI) *m*/*z*: [M + H]^+^ calcd for C_36_H_22_O_3_N_2_F_5_ 625.1545; found 625.1550. Anal. for 3 × C_36_H_21_F_5_N_2_O_3_: 1 × H_2_O: calcd C, 68.57; H, 3.46; N, 4.44. Found: C, 68.57; H, 3.43; N, 4.55.

##### 7-(3,5-Di-*tert*-butylphenyl)-14-(pentafluorophenyl)diindolo[3,2,1-de:3′,2′,1′-ij][1,5]naphthyridine-6,13-dione (**7**)

***Caution: 1,1,2,2-tetrachloroethane is a very toxic solvent. Work with this chemical must be carried out under very strictly observed safety conditions.*** A solution of 3,5-di-*tert*-butylphenylacetyl chloride **10** (0.95 g, 3.6 mmol) in anhydrous 1,1,2,2-tetrachloroethane (5 mL) was added to a suspension of **12** (270 mg, 0.6 mmol) in anhydrous 1,1,2,2-tetrachloroethane (20 mL) and heated to 155 °C (oil bath) in a nitrogen atmosphere. The mixture was brought to reflux and left to stir in a nitrogen atmosphere for 64 h. The solvent was removed under reduced pressure, and the residue was triturated with hexane (2 × 40 mL). The isolated solid was purified by column chromatography on silica gel using CH_2_Cl_2_ as an eluent. The pure product was obtained after trituration in pentane (3 × 40 mL) as a purple powder (52 mg, 13%). Mp: >300 °C. ^1^H NMR (400 MHz, CD_2_Cl_2_): δ 6.41–6.50 (m, 2H, ArH), 7.53–7.69 (m, 6H, ArH), 7.30–7.40 (m, 2H, ArH), 7.20–7.27 (m, 1H, ArH), 1.41 (s. 18H, ^t^Bu). ^19^F NMR (376 MHz, CD_2_Cl_2_): δ −137.36 (dd, *J*_1_ = 21 Hz, *J*_2_ = 7 Hz, 2F), −153.45 (tt, 1F), −162.10 (m, 2F). ^13^C{^1^H}NMR (126 MHz, CD_2_Cl_2_): δ 159.7, 157.8, 151.5, 145.6, 145.5 (m, *J*_CF_ = 251.4 Hz), 145.1, 142.4 (m, *J*_CF_ = 256.1 Hz), 138.6 (m, *J*_CF_ = 249.9 Hz), 136.4, 135.7, 133.7, 133.0, 132.8, 132.6, 126.8, 126.7, 126.19, 126.16, 125.7, 125.2, 125.1, 124.9, 124.2, 121.5, 118.2, 118.1, 113.4, 109.6, 35.6, 31.8. IR (KBr): 3069, 3060, 2954, 2925, 2906, 2869, 1744, 1606, 1641, 1592, 1595, 1499, 1484, 1413, 1395, 1363, 989, 756, 749 cm^–1^. UV–vis (CH_2_Cl_2_) λ_max_ (ε): 276 (4.6 × 10^4^), 360 (5.9 × 10^3^), 472 (1.0 × 10^4^), 505 (2.6 × 10^4^), 541 (3.9 × 10^4^) nm (M^–1^ cm^–1^). MS (APCI) [M+H]^+^*m*/*z* 665.2. HRMS (APCI) *m*/*z*: [M + H]^+^ calcd for C_40_H_30_O_2_N_2_F_5_ 665.2222; found 665.2221. Anal. C_40_H_29_F_5_N_2_O_2_: calcd C, 72.28; H, 4.40; N, 4.21. Found: C, 72.18; H, 4.39; N, 3.91.

##### 7,14-Bis(phenyl-4-*d*)diindolo[3,2,1-de:3′,2′,1′-ij][1,5]naphthyridine-6,13-dione (**1-*****d***_**2**_)

A solution of (phenyl-4-*d*)acetyl chloride (**22**, 1.77 g, 11.1 mmol) in an anhydrous isomer mixture of xylenes (5 mL) was added to a suspension of **8** (428 mg, 1.6 mmol) in an anhydrous isomer mixture of xylenes (50 mL) and heated to reflux (oil bath) in a nitrogen atmosphere for 74 h. The solvent was removed under reduced pressure, and the residue was triturated with toluene (2 × 40 mL), ethyl acetate (1 × 40 mL), ethanol (96%, 1 × 40 mL), and diethyl ether (1 × 40 mL). ^1^H NMR (Cl_2_CDCDCl_2_) revealed the resulting solid (75 mg) to be a mixture of **1-*****d***_**2**_ and the “half-cibalackrot” **25** (7-(phenyl-4-*d*)-6*H*-pyrido[1,2-a:3,4-b′]diindole-6,13(12*H*)-dione) in ratio 1:3. A solution of (phenyl-4-*d*)acetyl chloride **22** (0.275 g, 1.72 mmol) in 1,1,2,2-tetrachloroethane (2 mL) was added to a suspension of the mixture (75 mg) in anhydrous 1,1,2,2-tetrachloroethane (5 mL) and heated to reflux in a nitrogen atmosphere for 19 h. The solvent was removed under reduced pressure, and the residue was triturated with toluene (2 × 40 mL), ethyl acetate (1 × 40 mL), ethanol (96%, 1 × 40 mL), and diethyl ether (1 × 40 mL). The solid was dried under reduced pressure (150°C, 0.5 Torr, 5 h) to yield the product (45 mg, 6%). The ^1^H NMR spectrum revealed 87.5% of deuterium at expected positions. ^1^H NMR (400 MHz, Cl_2_CDCDCl_2_): δ 8.47 (d, *J* = 8.1 Hz, 2H, ArH), 7.74 (d, *J* = 8.1 Hz, 4H, ArH) 7.65–7.54 (m, 8H, ArH), 7.25 (t, *J* = 7.6 Hz, 2H, ArH). ^2^H NMR (77 MHz, Cl_2_CHCHCl_2_): δ 7.62 (br s). ^13^C{^1^H}NMR (126 MHz, Cl_2_CDCDCl_2_): δ 159.7, 144.8, 133.7, 132.6, 132.5, 131.4, 130.4, 129.4, 128.9, 126.6, 129.9, 125.8, 122.4, 117.8. MS (APCI) [M + H]^+^*m*/*z* 465.2. HRMS (ESI) *m*/*z*: [M + H]^+^ calcd for C_32_H_17_D_2_O_2_N_2_ 465.1567; found 465.1564. IR (KBr): 3111, 3077, 3036, 2284, 2264, 1603, 1574, 1480, 1435, 1417, 1266, 1075, 1016, 763 cm^–1^.

##### 7,14-Bis(phenyl-3,5-*d*_*2*_)diindolo[3,2,1-de:3′,2′,1′-ij][1,5]naphthyridine-6,13-dione (**1-*****d***_**4**_)

***Caution: 1,1,2,2-tetrachloroethane is a very toxic solvent. Work with this chemical must be carried out under very strictly observed safety conditions.*** A solution of (phenyl-3,5-*d*_*2*_)acetyl chloride **23** (1.70 g, 10.9 mmol) in anhydrous 1,1,2,2-tetrachloroethane (10 mL) was added to a suspension of **8** (380 mg, 1.5 mmol) and anhydrous 1,1,2,2-tetrachloroethane (10 mL) and heated to reflux (oil bath) in a nitrogen atmosphere for 43 h. The solvent was removed under reduced pressure, and the residue was triturated with toluene (2 × 40 mL), ethyl acetate (1 × 40 mL), ethanol (96%, 1 × 40 mL), and diethyl ether (1 × 40 mL). ^1^H NMR (Cl_2_CDCDCl_2_) revealed the resulting solid (155 mg) to be a mixture of the product **1**-*d*_4_ and “half-cibalackrot” **26** (7-(phenyl-3,5-*d*_2_)-6*H*-pyrido[1,2-a:3,4-b′]diindole-6,13(12*H*)-dione) in a 1:4 ratio. A solution of (phenyl-3,5-*d*_2_)acetyl chloride (**23**, 480 mg, 3.1 mmol) in anhydrous 1,1,2,2-tetrachloroethane (5 mL) was added to a suspension of the resulting mixture (75 mg) in anhydrous 1,1,2,2-tetrachloroethane (5 mL) and heated to reflux in a nitrogen atmosphere for 43 h. The solvent was removed under reduced pressure, and the residue was triturated with toluene (2 × 40 mL), ethyl acetate (1 × 40 mL), ethanol (96%, 1 × 40 mL), and diethyl ether (1 × 40 mL). Traces of **26** were removed by column chromatography on silica gel using CH_2_Cl_2_—ethyl acetate 100:1 (v/v) as an eluent. The pure product was obtained as a purple powder (59 mg, 9%). The ^1^H NMR spectrum revealed 80% deuteriation at the right positions. ^1^H NMR (400 MHz, Cl_2_CDCDCl_2_): δ 8.48 (d, *J* = 7.1 Hz, 2H, ArH), 7.74 (m, 4H, ArH) 7.65–7.54 (m, 6H, ArH), 7.25 (t, *J* = 7.5 Hz, 2H, ArH). ^2^H NMR (77 MHz, Cl_2_CHCHCl_2_): δ 7.68 (brs). ^13^C{^1^H}NMR (126 MHz, Cl_2_CDCDCl_2_): δ 159.6, 144.8, 133.6, 132.6, 132.5, 131.4, 130.3, 129.4, 128.7, 126.6, 125.9, 125.8, 122.5, 117.8. MS (APCI) [M + H]^+^*m*/*z* 467.2. HRMS (APCI) *m*/*z*: [M + H]^+^ calcd for C_32_H_15_D_4_O_2_N_2_ 467.1692; found 467.1688. IR (KBr): 3109, 3073, 3036, 2336, 2261, 1630, 1605, 1588, 1574, 1564, 1480, 1444, 1433, 1418, 1405, 1207, 1255, 1071, 1016, 763 cm^–1^.

##### 7,14-Bis(phenyl-*d*_5_)diindolo[3,2,1-de:3′,2′,1′-ij][1,5]naphthyridine-6,13-dione (**1-*****d***_**10**_)

***Caution: 1,1,2,2-tetrachloroethane is a very toxic solvent. Work with this chemical must be carried out under very strictly observed safety conditions.*** A solution of (phenyl-*d*_5_)acetyl chloride (**24**, 2.53 g, 15.7 mmol) in anhydrous 1,1,2,2-tetrachloroethane (15 mL) was added to a suspension of **8** (380 mg, 1.45 mmol) in anhydrous 1,1,2,2-tetrachloroethane (25 mL) and heated to reflux (oil bath) in a nitrogen atmosphere for 48 h. The solvent was removed under reduced pressure, and the residue was triturated with toluene (2 × 40 mL), ethyl acetate (1 × 40 mL), ethanol (96%, 1 × 40 mL), and diethyl ether (1 × 40 mL). The resulting solid was purified by column chromatography on silica gel using CH_2_Cl_2_–ethyl acetate 100:1 (v/v) as an eluent. The pure product was obtained as a purple powder (54 mg, 8%). The ^1^H NMR spectrum showed 98.8% of deuterium at specified positions. ^1^H NMR (400 MHz, Cl_2_CDCDCl_2_): δ 8.47 (d, *J* = 8 Hz, 2H, ArH), 7.64–7.54 (m, 4H, ArH), 7.25 (t, *J* = 7.7 Hz, 2H, ArH). ^2^H NMR (77 MHz, Cl_2_CHCHCl_2_): δ 7.66 (brs). ^13^C{^1^H}NMR (126 MHz, Cl_2_CDCDCl_2_): δ 159.6, 144.8, 133.5, 132.6, 132.4, 131.4, 130.0, 129.1, 128.4, 126.5, 125.84, 125.83, 122.4, 117.8. MS (ESI) [M + H]^+^*m*/*z* 473.2. HRMS (ESI) *m*/*z*: [M + H]^+^ calcd for C_32_H_9_D_10_O_2_N_2_ 473.2069; found 473.2067. IR (KBr): 3110, 3073, 3036, 2270, 1630, 1605, 1574, 1530, 1480, 1436, 1415, 1385, 1282, 1276, 1218, 1073, 976, 762 cm^–1^.

#### Preprint

This manuscript was posted on a preprint server.^51^

## Data Availability

The data underlying this study are available in the published article and its online Supporting Material.

## Supporting Information Available

^1^H, ^13^C, ^1^H–^1^H COSY, HSQC, and HMBC NMR spectra for all new compounds; nanosecond transient absorption spectroscopy (TAS) measurements; and crystallography information (PDF)
