# Supplementary material for: Phenyl-Substituted Cibalackrot Derivatives: Synthesis, Structure, and Solution Photophysics
Source: J Org Chem. 2023 May 23;88(11):6573–87. doi: 10.1021/acs.joc.2c02706 (PMC10242757; doi:10.1021/acs.joc.2c02706)
Supplement: Supplementary file 1 — jo2c02706_si_001.pdf [file jo2c02706_si_001.pdf]

Supporting information

Phenyl-Substituted Cibalackrot Derivatives: Synthesis, Structure, and Solution Photophysics

*Jiří Kaleta, Miroslav Dudič, Lucie Ludvíková, Alan Liška, Alexandr Zaykov, Igor Rončević, Milan Mašát, Lucie Bednárová, Paul I. Dron, Simon J. Teat, and Josef Michl**

Corresponding Author:

Josef Michl - *Institute of Organic Chemistry and Biochemistry of the Czech Academy of Sciences, Flemingovo nám. 542/2, 160 00 Prague, Czech Republic; Department of Chemistry, University of Colorado, Boulder, Colorado 80309-0215, United States;* *E-mail: michlj@colorado.edu

Authors:

Jiří Kaleta - *Institute of Organic Chemistry and Biochemistry of the Czech Academy of Sciences, Flemingovo nám. 542/2, 160 00 Prague, Czech Republic*

Miroslav Dudič - *Institute of Organic Chemistry and Biochemistry of the Czech Academy of Sciences, Flemingovo nám. 542/2, 160 00 Prague, Czech Republic*

Lucie Ludvíková - *Institute of Organic Chemistry and Biochemistry of the Czech Academy of Sciences, Flemingovo nám. 542/2, 160 00 Prague, Czech Republic*

Alan Liška - *J. Heyrovsky Institute of Physical Chemistry, Academy of Sciences of the Czech Republic, Dolejškova 3, 182 23 Prague 8, Czech Republic*

Alexander Zaykov - *Institute of Organic Chemistry and Biochemistry of the Czech Academy of Sciences, Flemingovo nám. 542/2, 160 00 Prague, Czech Republic*

Igor Rončevič - *Institute of Organic Chemistry and Biochemistry of the Czech Academy of Sciences, Flemingovo nám. 542/2, 160 00 Prague, Czech Republic*.

Milan Mašát - *Institute of Organic Chemistry and Biochemistry of the Czech Academy of Sciences, Flemingovo nám. 542/2, 160 00 Prague, Czech Republic*

Lucie Bednárová - *Institute of Organic Chemistry and Biochemistry of the Czech Academy of Sciences, Flemingovo nám. 542/2, 160 00 Prague, Czech Republic*

Paul I. Dron - *Institute of Organic Chemistry and Biochemistry of the Czech Academy of Sciences, Flemingovo nám. 542/2, 160 00 Prague, Czech Republic*

Simon J. Teat - *Advanced Light Source, Lawrence Berkeley National Laboratory, Berkeley, CA 94720-1460, USA*

Table of Contents

^{1}H NMR (400 MHz, $CDCl_3$) – Derivative **9** S1

^{1}H NMR (400 MHz, $CDCl_3$) – Derivative **10** S2

^{1}H NMR (400 MHz, $CDCl_3$) – Derivative **11** S3

^{19}F NMR (376 MHz, $CDCl_3$) – Derivative **11** S4

^{1}H NMR (500 MHz, DMSO-d_6) – Derivative **12** S5

^{13}C{^{1}H} NMR APT NMR (125 MHz, DMSO-d_6) – Derivative **12** S6

^{19}F NMR (376 MHz, DMSO-d_6) – Derivative **12** S7

^{1}H – ^{1}H COSY (DMSO-d_6) – Derivative **12** S8

HSQC (DMSO-d_6) – Derivative **12** S9
HMBC (DMSO-d_6) – Derivative **12** S10
^{1}H NMR (500 MHz, THF-d_8) – Derivative **13** S11
^{13}C{^{1}H} NMR (125 MHz, THF-d_8) – Derivative **13** S12
^{13}C{^{1}H} APT NMR (125 MHz, THF-d_8) – Derivative **13** S13
^{1}H – ^{1}H COSY (THF-d_8) – Derivative **13** S14
HSQC (THF-d_8) – Derivative **13** S15
HMBC (THF-d_8) – Derivative **13** S16
^{1}H NMR (400 MHz, $Cl_2CDCDCl_2$) – Derivative **2** S17
^{1}H NMR (400 MHz, $Cl_2CDCDCl_2$) – Derivative **2** – AA'XX' splitting patterns S18
^{13}C{^{1}H} APT NMR (125 MHz, $Cl_2CDCDCl_2$) – Derivative **2** S19
^{1}H – ^{1}H COSY ($Cl_2CDCDCl_2$) – Derivative **2** S20
HSQC ($Cl_2CDCDCl_2$) – Derivative **2** S21
HMBC ($Cl_2CDCDCl_2$) – Derivative **2** S22
^{1}H NMR (500 MHz, $CDCl_3$) – Derivative **3** S23
^{13}C{^{1}H} NMR (100 MHz, $CDCl_3$) – Derivative **3** S24
^{13}C{^{1}H} APT NMR (100 MHz, $CDCl_3$) – Derivative **3** S25
^{1}H – ^{1}H COSY ($CDCl_3$) – Derivative **3** S26
HSQC ($CDCl_3$) – Derivative **3** S27
HMBC ($CDCl_3$) – Derivative **3** S28
^{1}H NMR (400 MHz, $Cl_2CDCDCl_2$) – Derivative **4** S29
^{13}C{^{1}H} APT (125 MHz, $Cl_2CDCDCl_2$) – Derivative **4** S30
^{19}F (376 MHz, $Cl_2CDCDCl_2$) – Derivative **4** S31
^{1}H – ^{1}H COSY ($Cl_2CDCDCl_2$) – Derivative **4** S32
HSQC ($Cl_2CDCDCl_2$) – Derivative **4** S33
HMBC ($Cl_2CDCDCl_2$) – Derivative **4** S34
^{1}H NMR (500 MHz, $CDCl_3$) – Derivative **5** S35
^{13}C{^{1}H} NMR (100 MHz, $CDCl_3$) – Derivative **5** S36
^{13}C{^{1}H} APT NMR (100 MHz, $CDCl_3$) – Derivative **5** S37
^{1}H – ^{1}H COSY ($CDCl_3$) – Derivative **5** S38
HSQC ($CDCl_3$) – Derivative **5** S39

HMBC ($CDCl_3$) – Derivative **5** S40
^{1}H NMR (600 MHz, $Cl_2CDCDCl_2$) – Derivative **6** S41
$^{13}C\{^{1}H\}$ APT (150 MHz, $Cl_2CDCDCl_2$) – Derivative **6** S42
^{19}F (470 MHz, $Cl_2CDCDCl_2$) – Derivative **6** S43
^{1}H – ^{1}H COSY ($Cl_2CDCDCl_2$) – Derivative **6** S44
HSQC ($Cl_2CDCDCl_2$) – Derivative **6** S45
HMBC ($Cl_2CDCDCl_2$) – Derivative **6** S46
^{1}H NMR (400 MHz, CD_2Cl_2) – Derivative **7** S47
$^{13}C\{^{1}H\}$ APT (125 MHz, CD_2Cl_2) – Derivative **7** S48
^{19}F (376 MHz, CD_2Cl_2) – Derivative **7** S49
^{1}H – ^{1}H COSY (CD_2Cl_2) – Derivative **7** S50
HSQC (CD_2Cl_2) – Derivative **7** S51
HMBC (CD_2Cl_2) – Derivative **7** S52
^{1}H NMR (400 MHz, $Cl_2CDCDCl_2$) – Derivative **1-d_2** S53
^{2}H NMR (77 MHz, $Cl_2CDCDCl_2$) – Derivative **1-d_2** S54
$^{13}C\{^{1}H\}$ APT (125 MHz, $Cl_2CDCDCl_2$) – Derivative **1-d_2** S55
^{1}H – ^{1}H COSY ($Cl_2CDCDCl_2$) – Derivative **1-d_2** S56
HSQC ($Cl_2CDCDCl_2$) – Derivative **1-d_2** S57
HMBC ($Cl_2CDCDCl_2$) – Derivative **1-d_2** S58
^{1}H NMR (400 MHz, $Cl_2CDCDCl_2$) – Derivative **1-d_4** S59
^{2}H NMR (77 MHz, $Cl_2CDCDCl_2$) – Derivative **1-d_4** S60
$^{13}C\{^{1}H\}$ APT (125 MHz, $Cl_2CDCDCl_2$) – Derivative **1-d_4** S61
HSQC ($Cl_2CDCDCl_2$) – Derivative **1-d_4** S62
HMBC ($Cl_2CDCDCl_2$) – Derivative **1-d_4** S63
^{1}H NMR (400 MHz, $Cl_2CDCDCl_2$) – Derivative **1-d_{10}** S64
^{2}H NMR (77 MHz, $Cl_2CDCDCl_2$) – Derivative **1-d_{10}** S65
$^{13}C\{^{1}H\}$ APT (125 MHz, $Cl_2CDCDCl_2$) – Derivative **1-d_{10}** S66
HSQC ($Cl_2CDCDCl_2$) – Derivative **1-d_{10}** S67
HMBC ($Cl_2CDCDCl_2$) – Derivative **1-d_{10}** S68
Nanosecond Transient Absorption Spectroscopy (TAS) Measurements S69-74
Table S1. Observed (obsd., in dichloromethane) and Calculated (con, dis) Properties of Singlet States of **1** - **7** (energies in $10^{3}cm^{-1}$). S69

Estimation of Triplets' Energies of **1-7** S70-71

Figure S1. Plot of Logarithms of the Rate Constant for Energy Transfer (k_q) to **1**-**7** S70

Table S2. Singlet State Energies of **1**-**7** and Measured Rate Constants for Triplet State Energy Transfer to **1**-**7** S71

Determination of Pure Triplet-Triplet Excited State Absorption Spectra of **1-7** S71-74

Figure S2: Estimation of Triplet-Triplet Absorption Spectra of **1-7** by Linear Analysis Using the Singlet Depletion Method. S71-74

X-Ray Structure, Crystal Packing and X-Ray Data S75-80

Figure S3. Cibalackrot **2**. a) X-Ray Structure. b) Crystal Packing. S75

Figure S4. Cibalackrot **3**. a) X-Ray Structure. b) Crystal Packing. S76

Figure S5. Cibalackrot **4α**. a) X-Ray Structure. b) Crystal Packing. S77

Figure S6. Cibalackrot **4β**. a) X-Ray Structure. b) Crystal Packing. S78

Figure S7. Cibalackrot **6**. a) X-Ray Structure. b) Crystal Packing. S79

Figure S8. Cibalackrot **7**. a) X-Ray Structure. b) Crystal Packing. S80

Table S3. Parameters of Single Crystals of **2**, **3**, **4α**, **4β**, **6** and **7**. S81-82

References S83

^{1}H NMR (400 MHz, $CDCl_3$) – Derivative 9

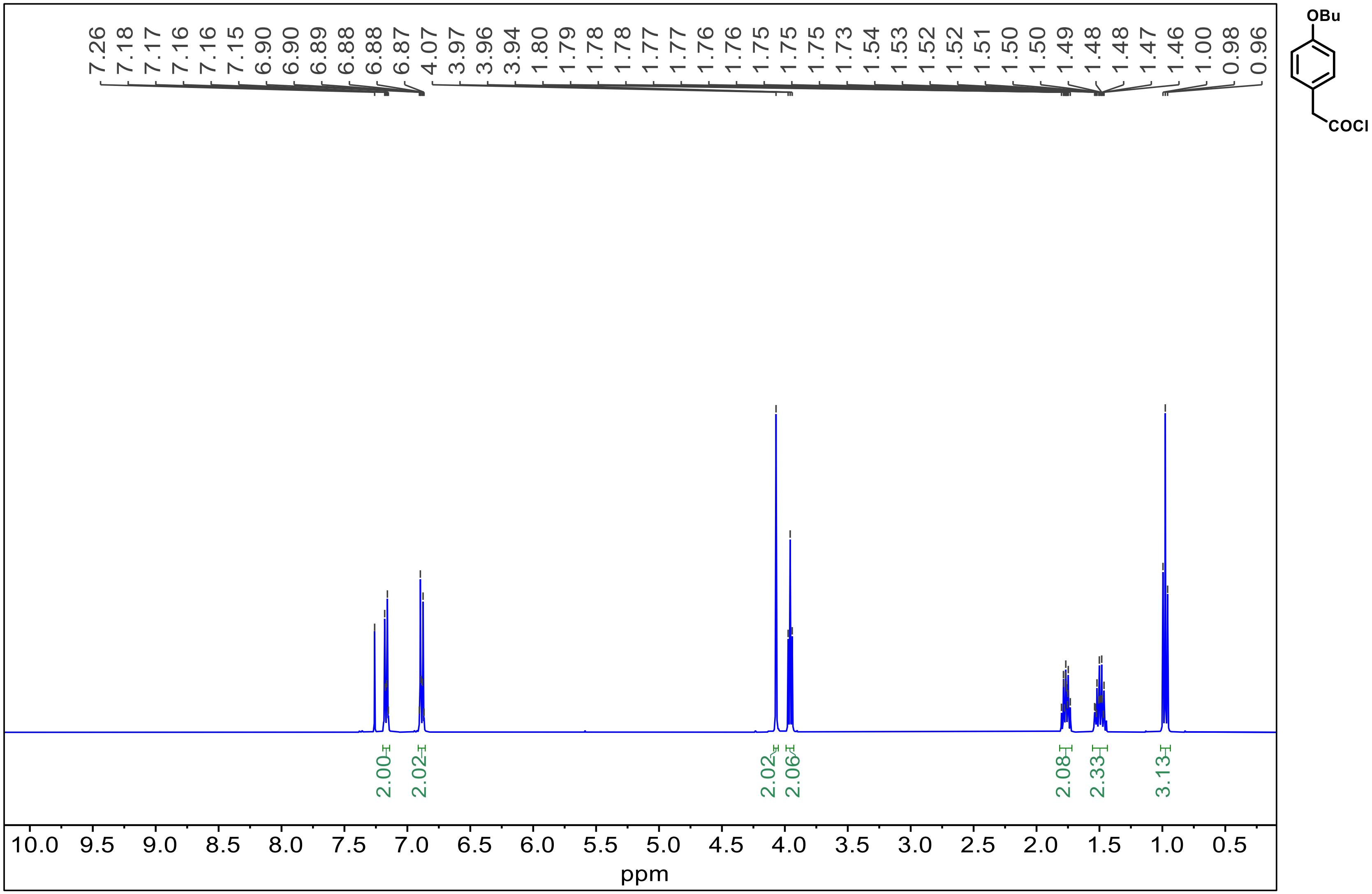

^{1}H NMR (400 MHz, CDCl$_3$) – Derivative 10

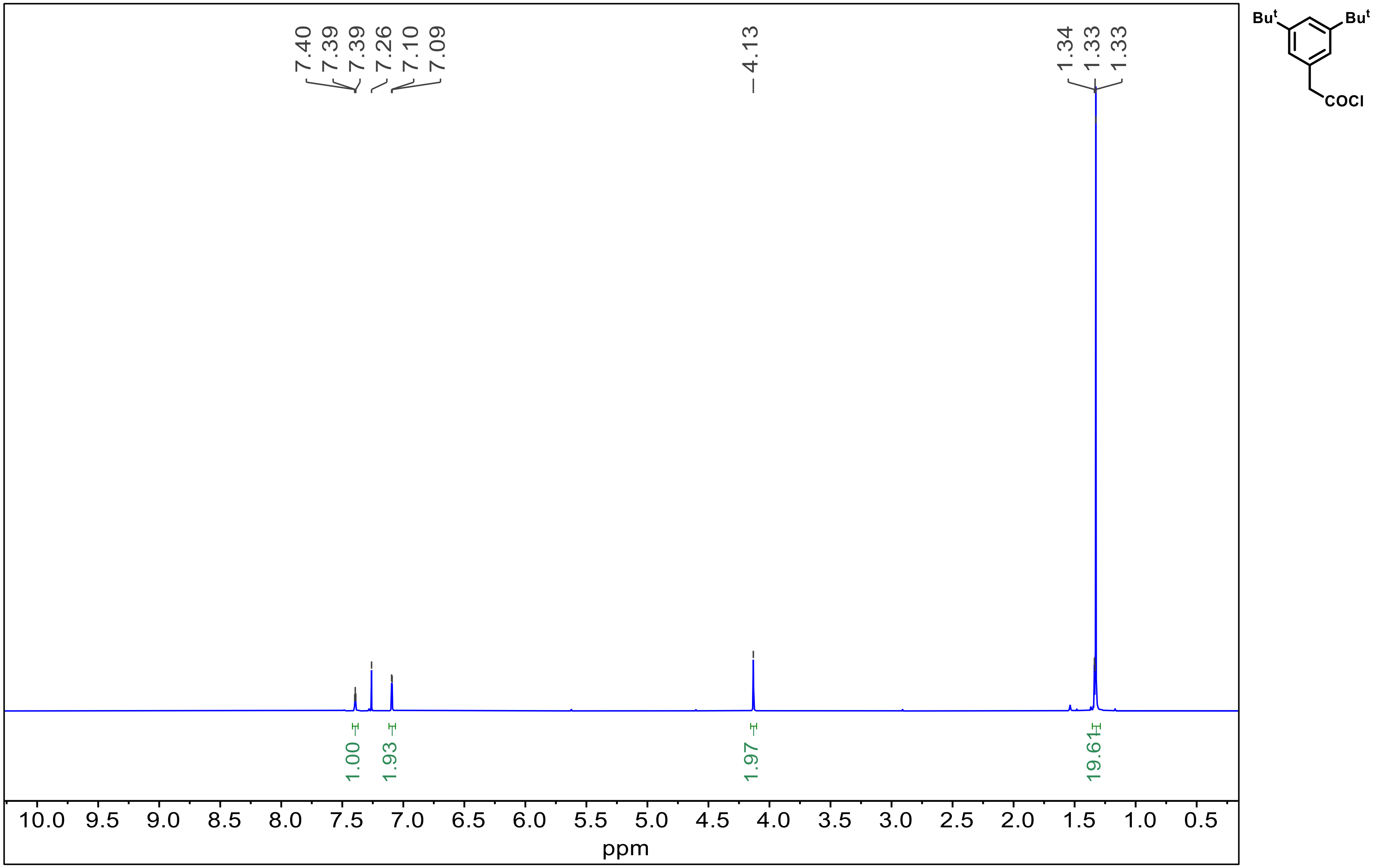

^{1}H NMR (400 MHz, $CDCl_3$) – Derivative 11

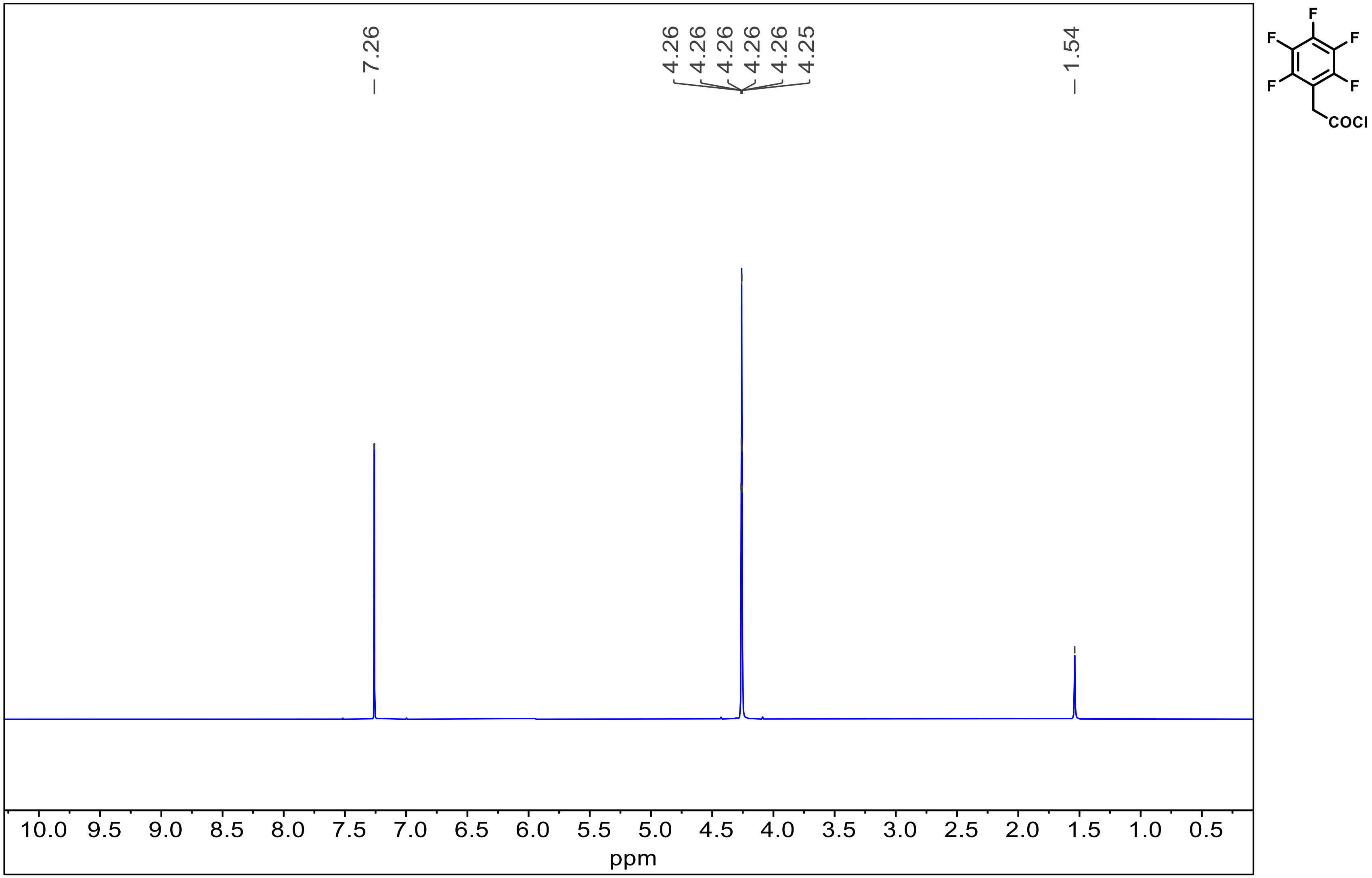

^{19}F NMR (376 MHz, CDCl$_3$) – Derivative 11

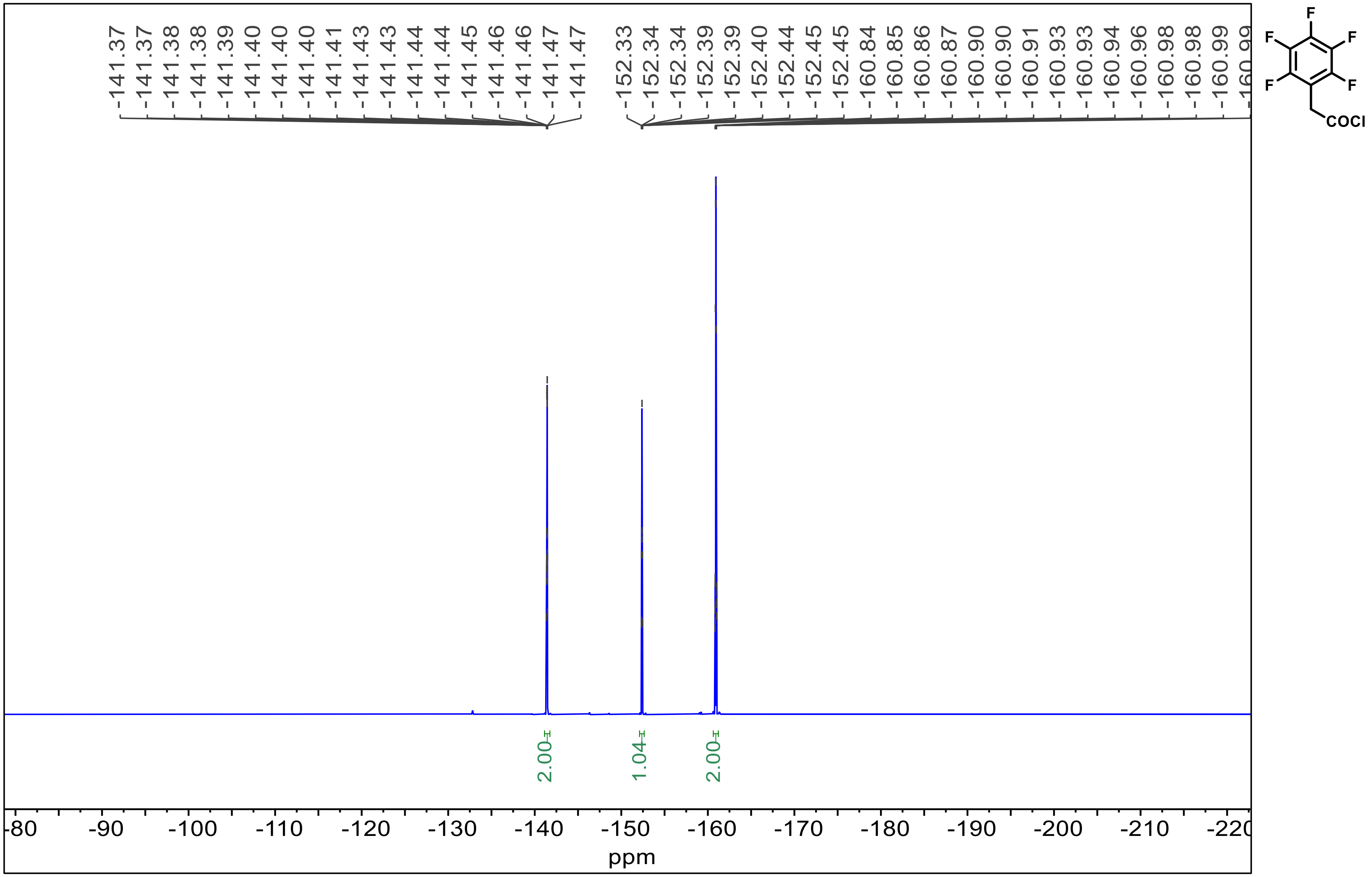

^{1}H NMR (500 MHz, DMSO-d_6) – Derivative 12

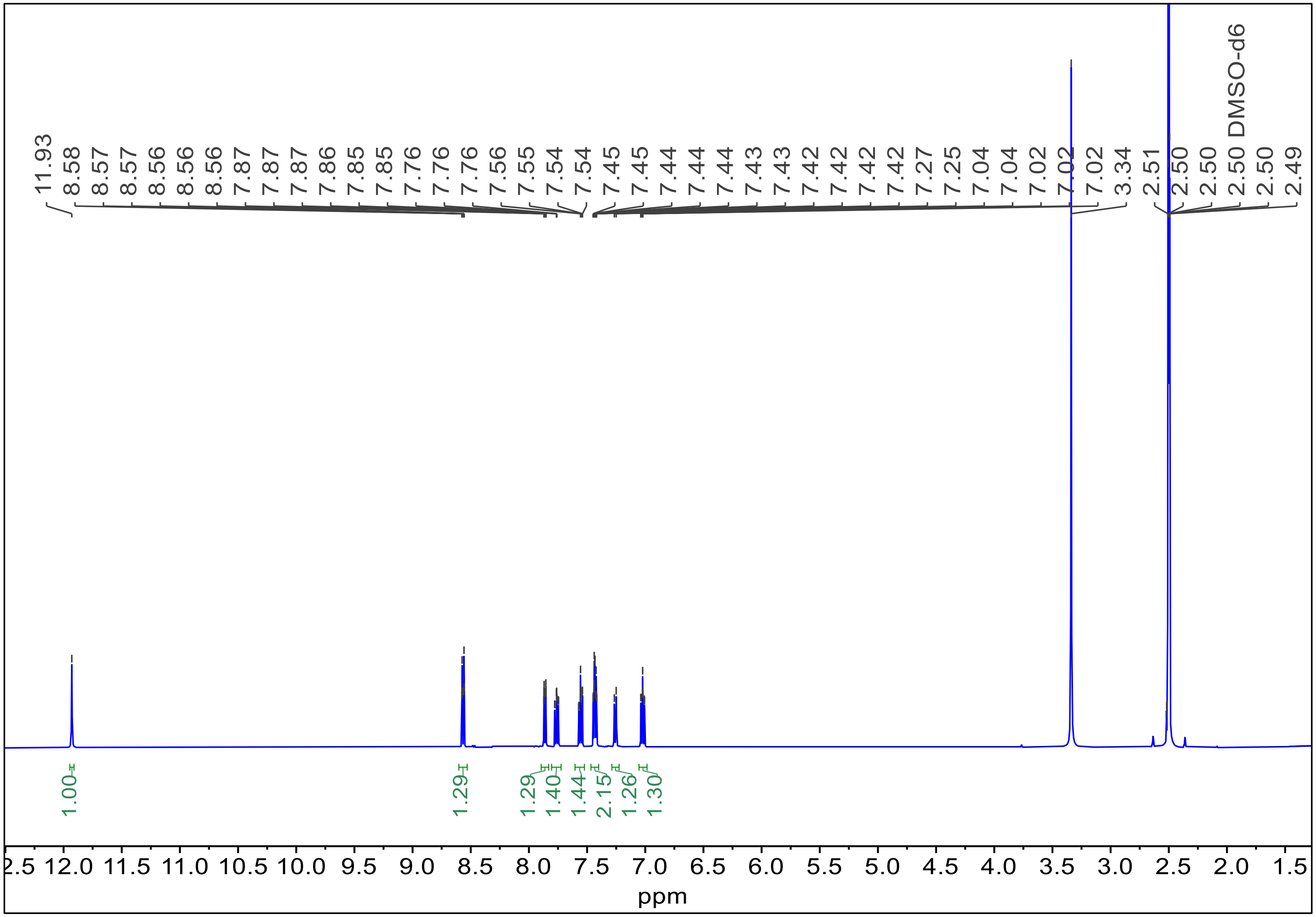

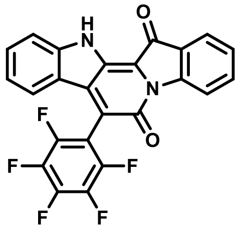

$^{13}C\{^{1}H\}$ APT NMR (125 MHz, DMSO-d_6) – Derivative 12

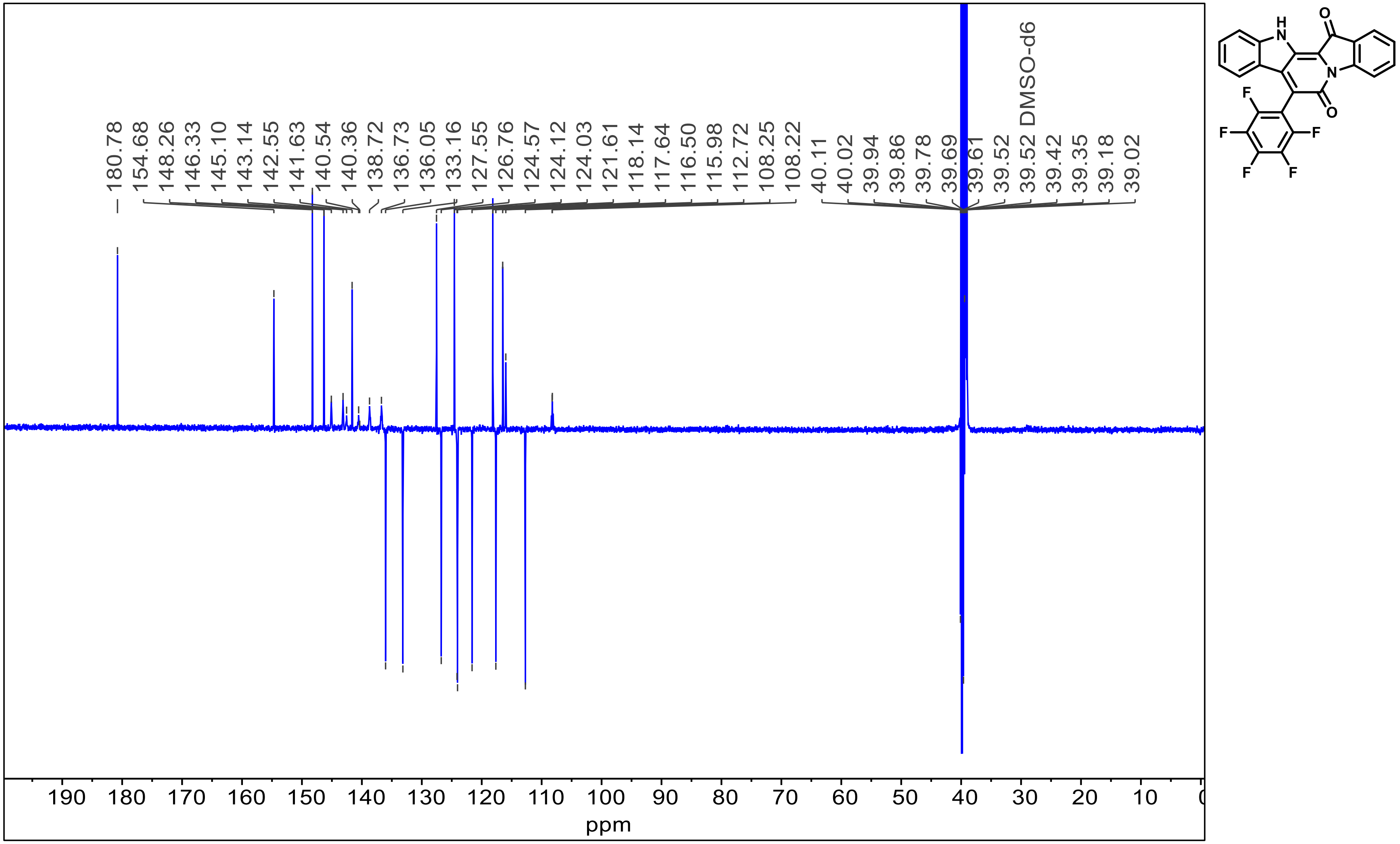

^{19}F NMR (376 MHz, DMSO-d_6) – Derivative 12

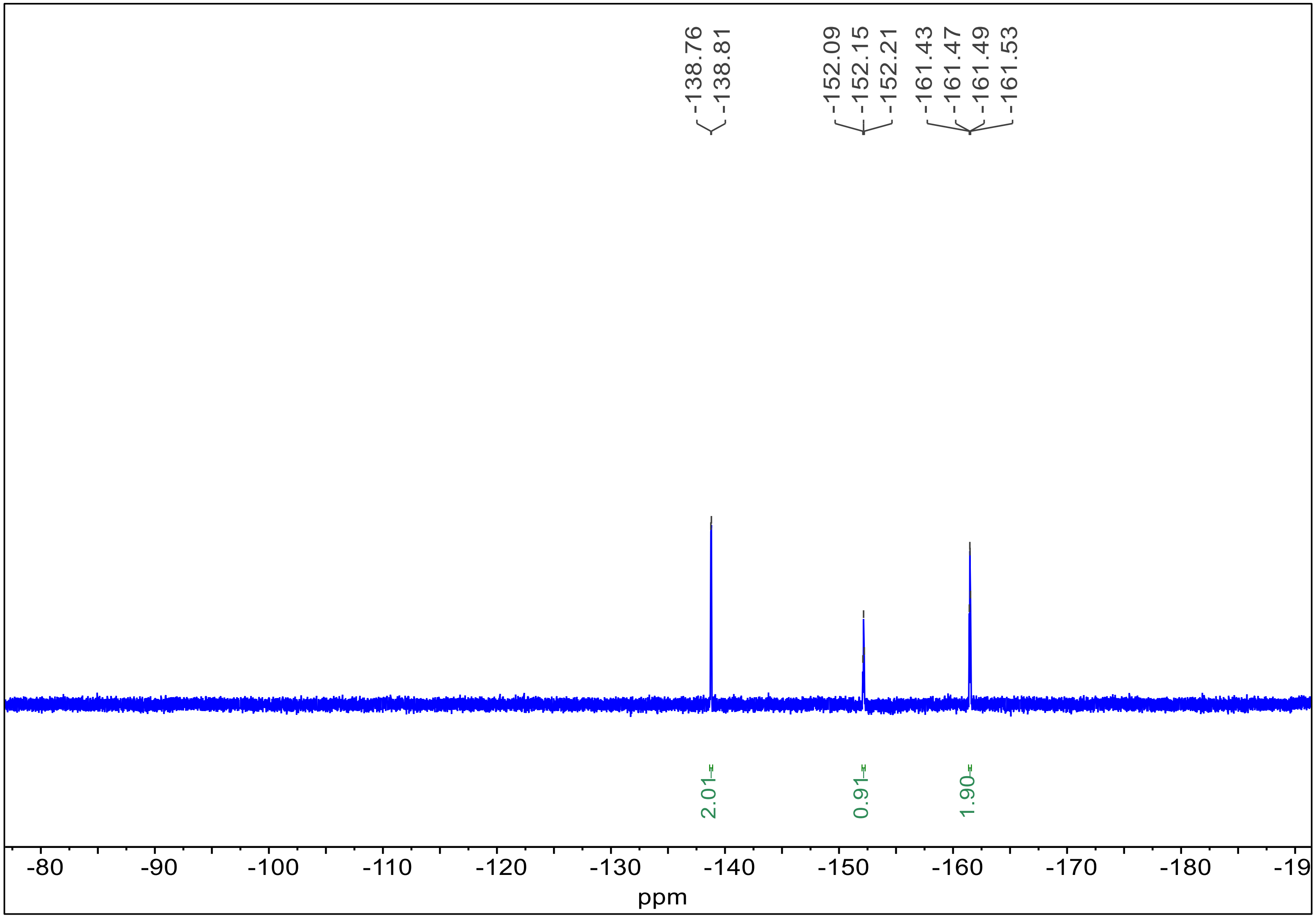

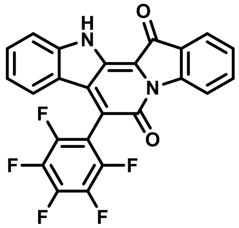

^{1}H – ^{1}H COSY (DMSO-d_6) – Derivative 12

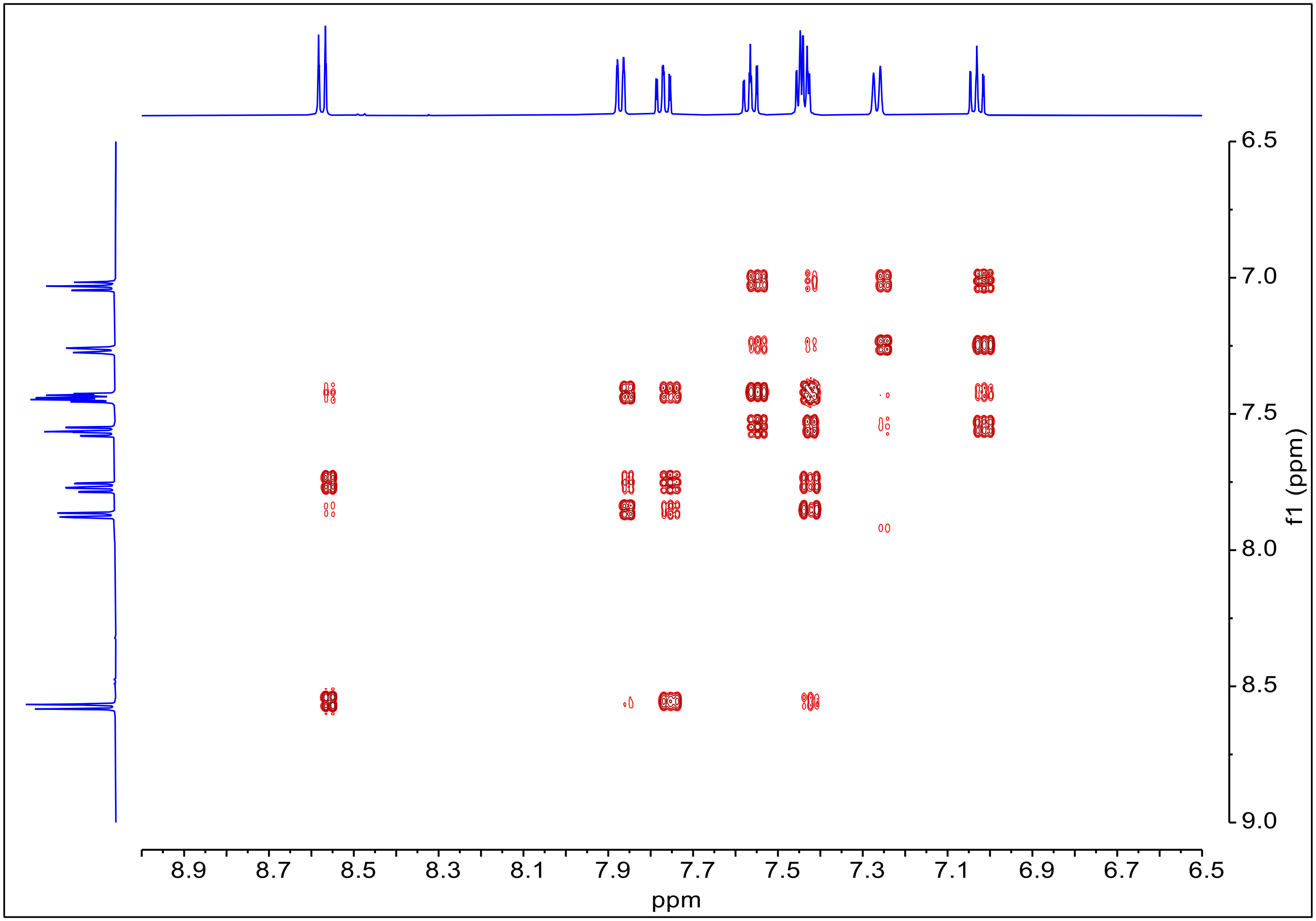

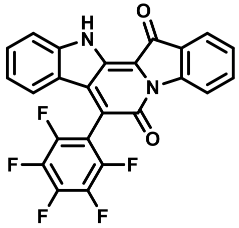

HSQC (DMSO-d_6) – Derivative 12

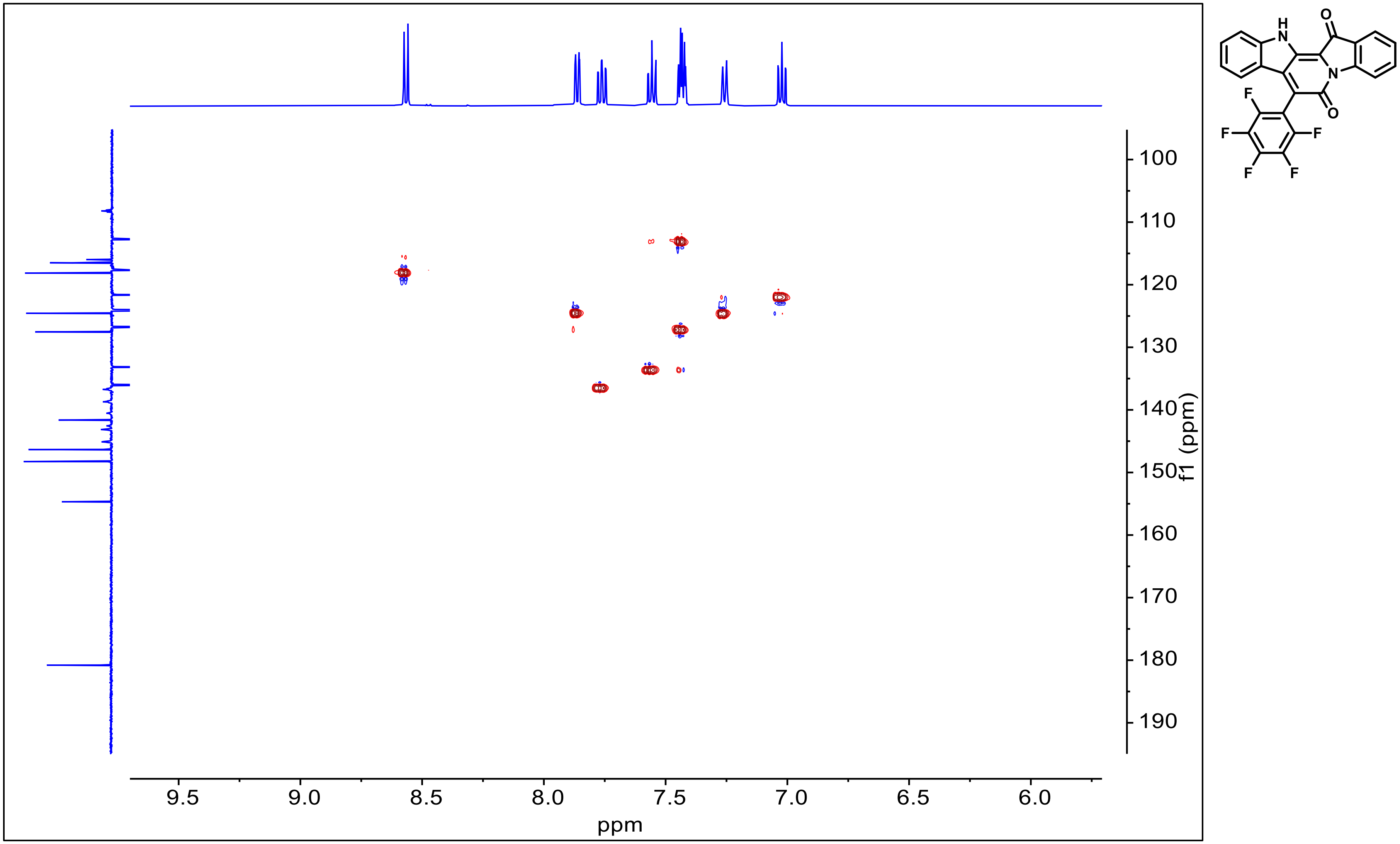

HMBC (DMSO-d_6) – Derivative 12

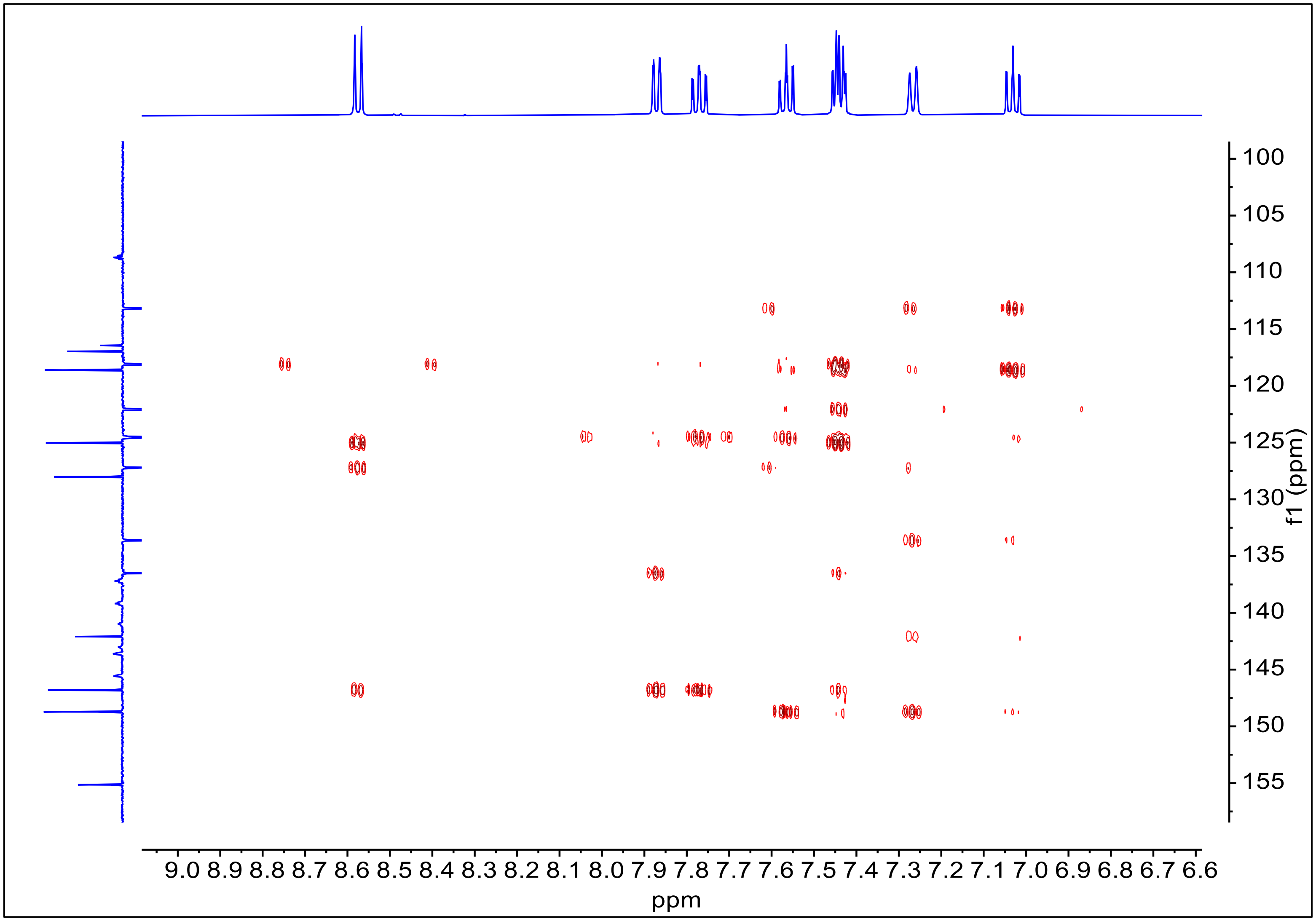

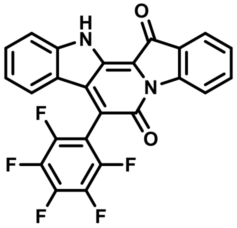

^{1}H NMR (500 MHz, THF-d_8) – Derivative 13

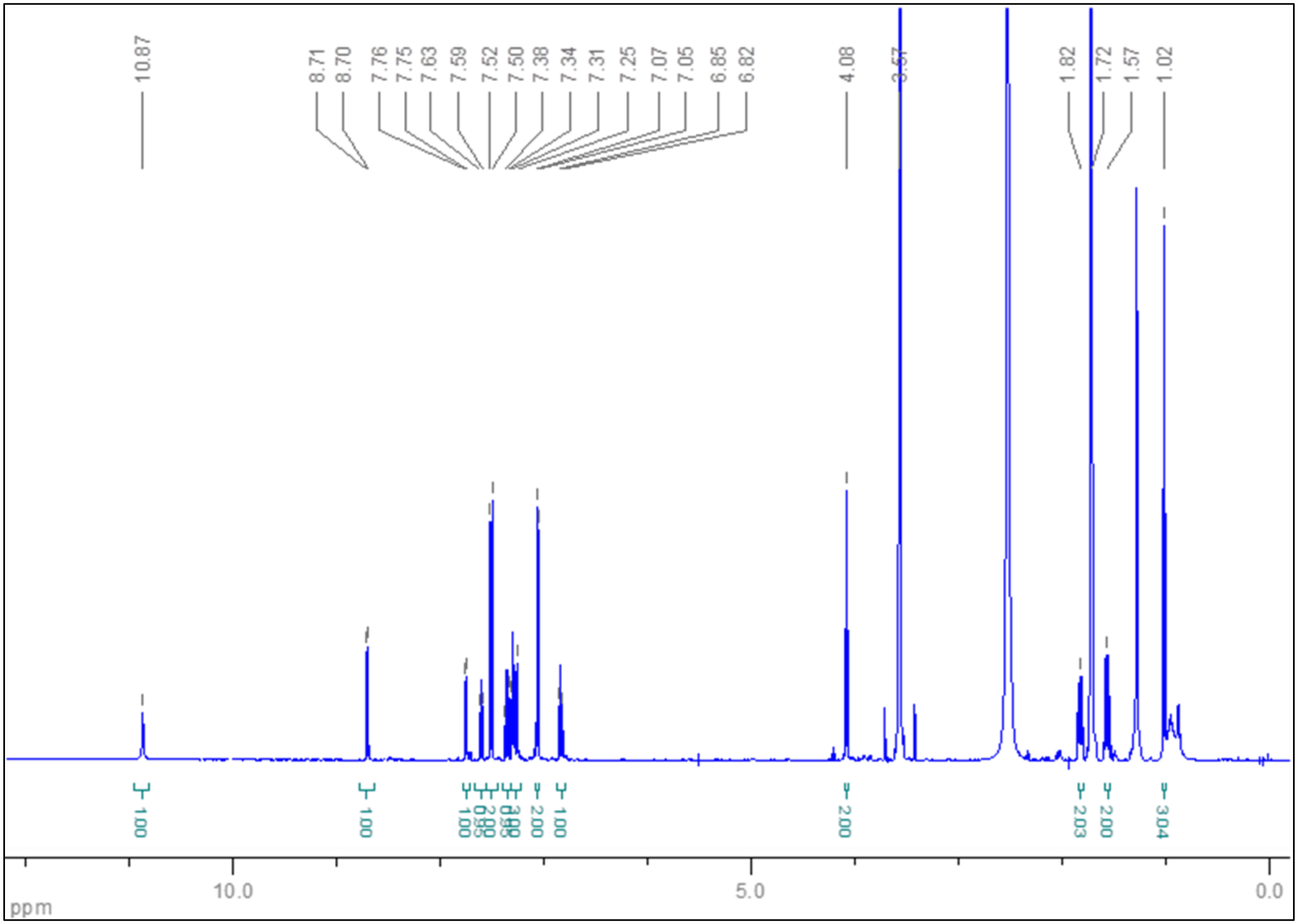

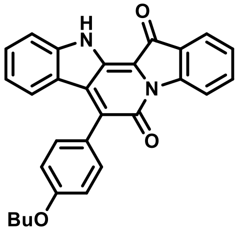

..

$^{13}C\{^{1}H\}$ NMR (125 MHz, THF-d_8) – Derivative 13

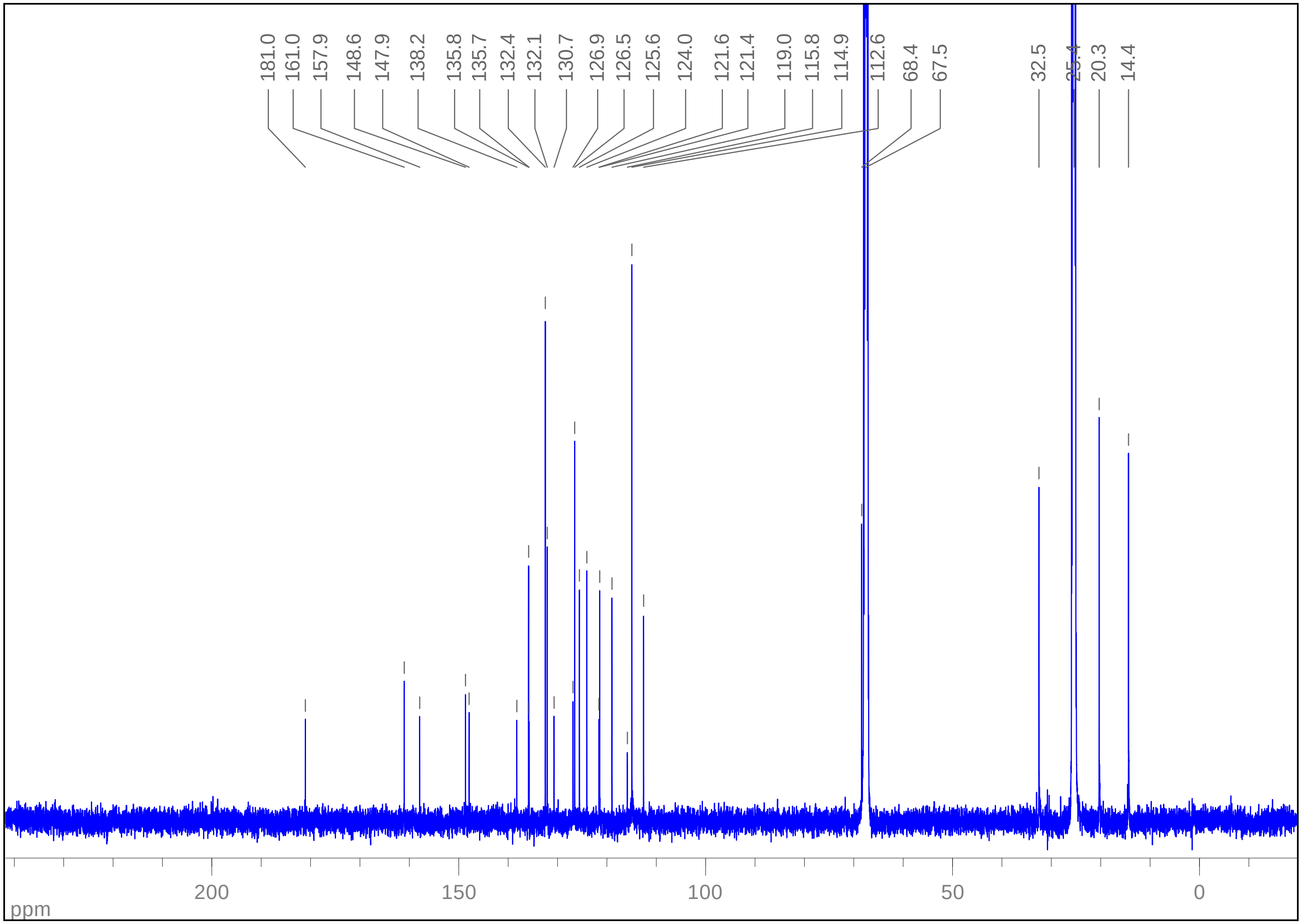

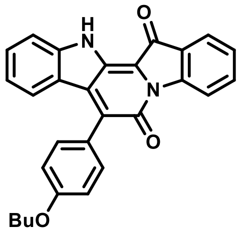

$^{13}C\{^{1}H\}$ APT NMR (125 MHz, THF-d_8) – Derivative 13

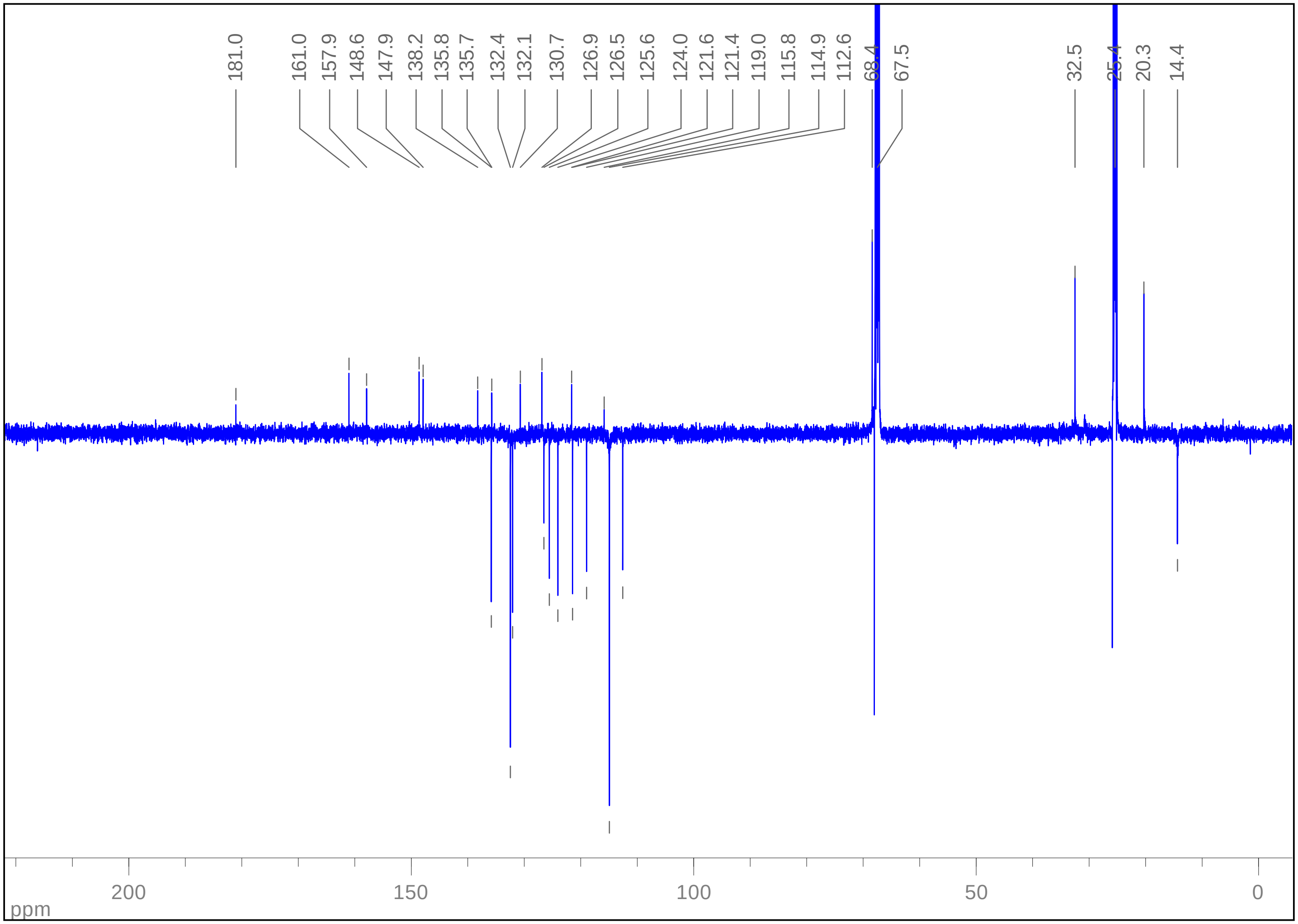

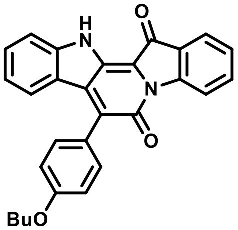

^{1}H – ^{1}H COSY (THF-d_8) – Derivative 13

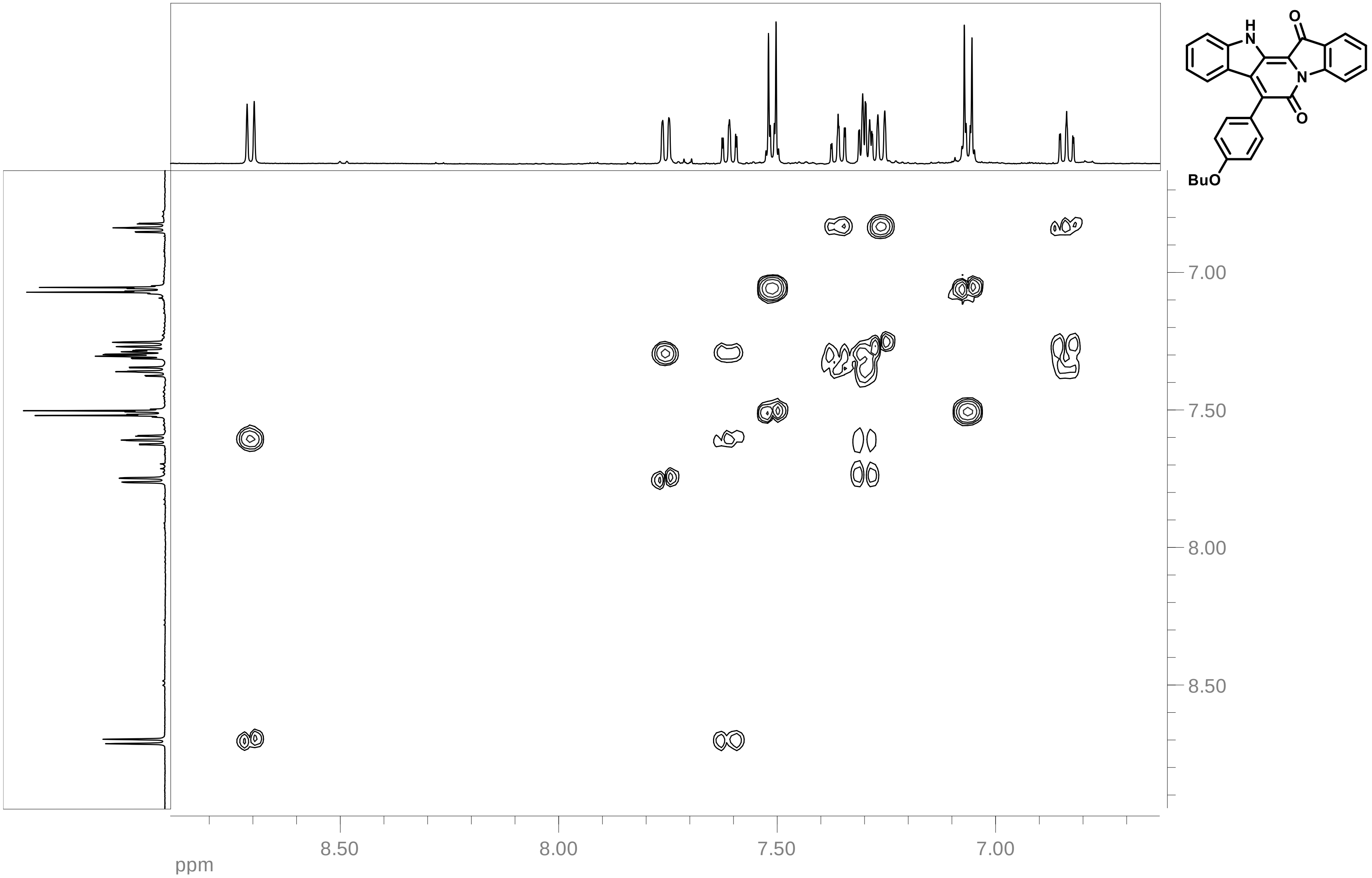

HSQC (THF-d_8) – Derivative 13

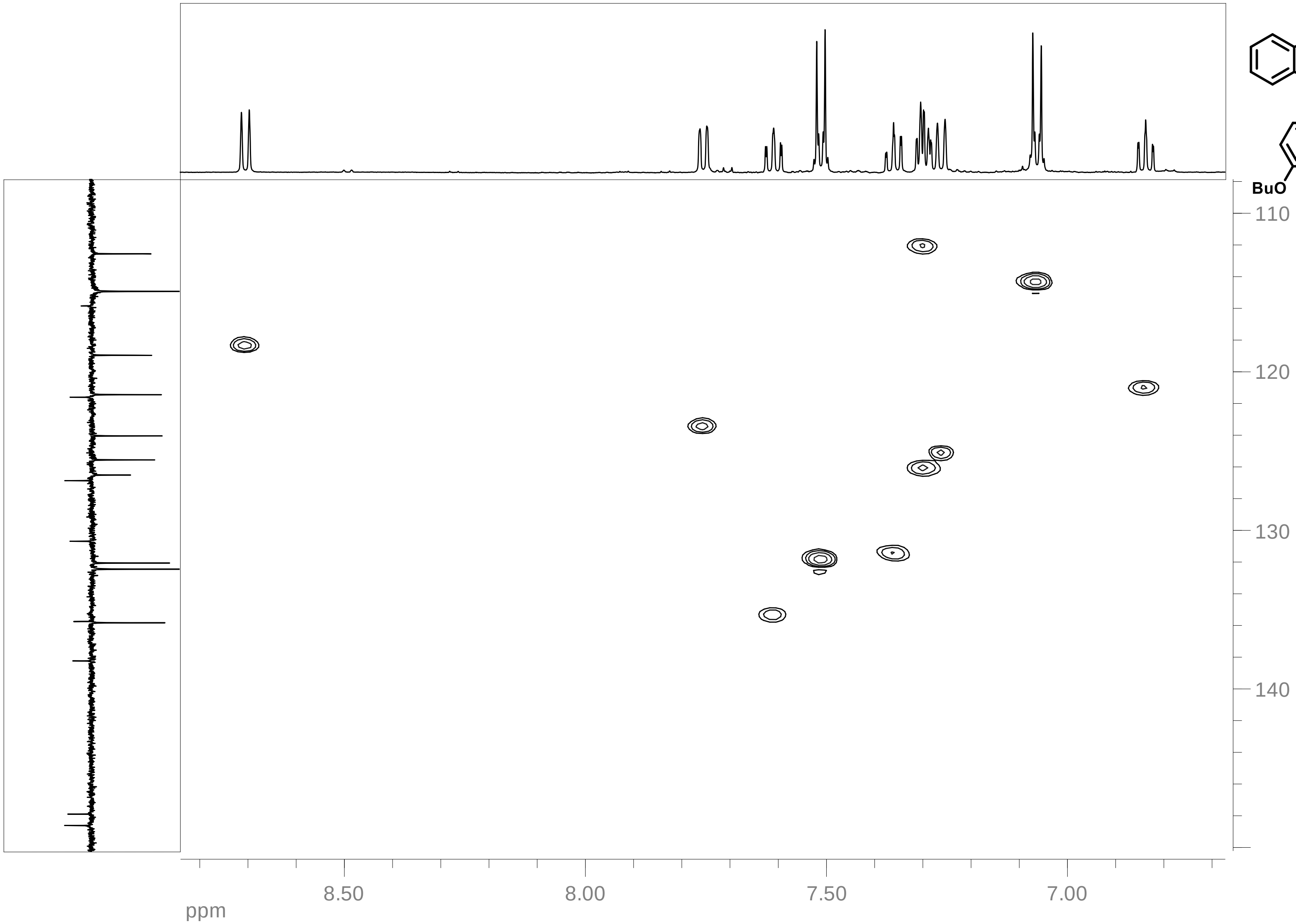

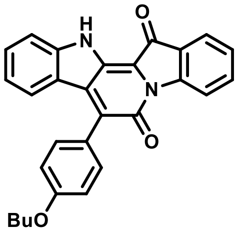

HMBC (THF-d_8) – Derivative 13

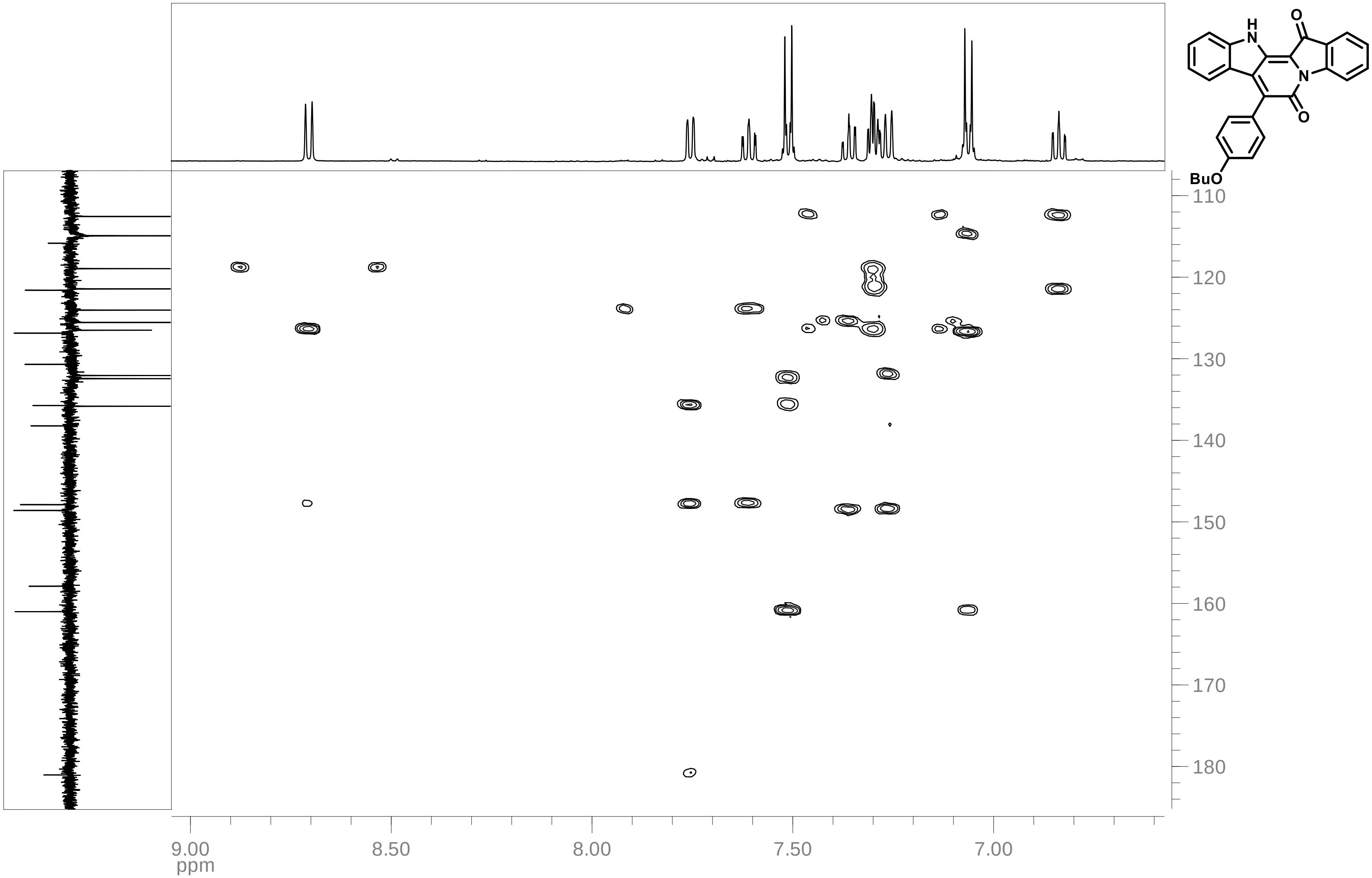

^{1}H NMR (400 MHz, $Cl_2CDCDCl_2$) – Derivative 2

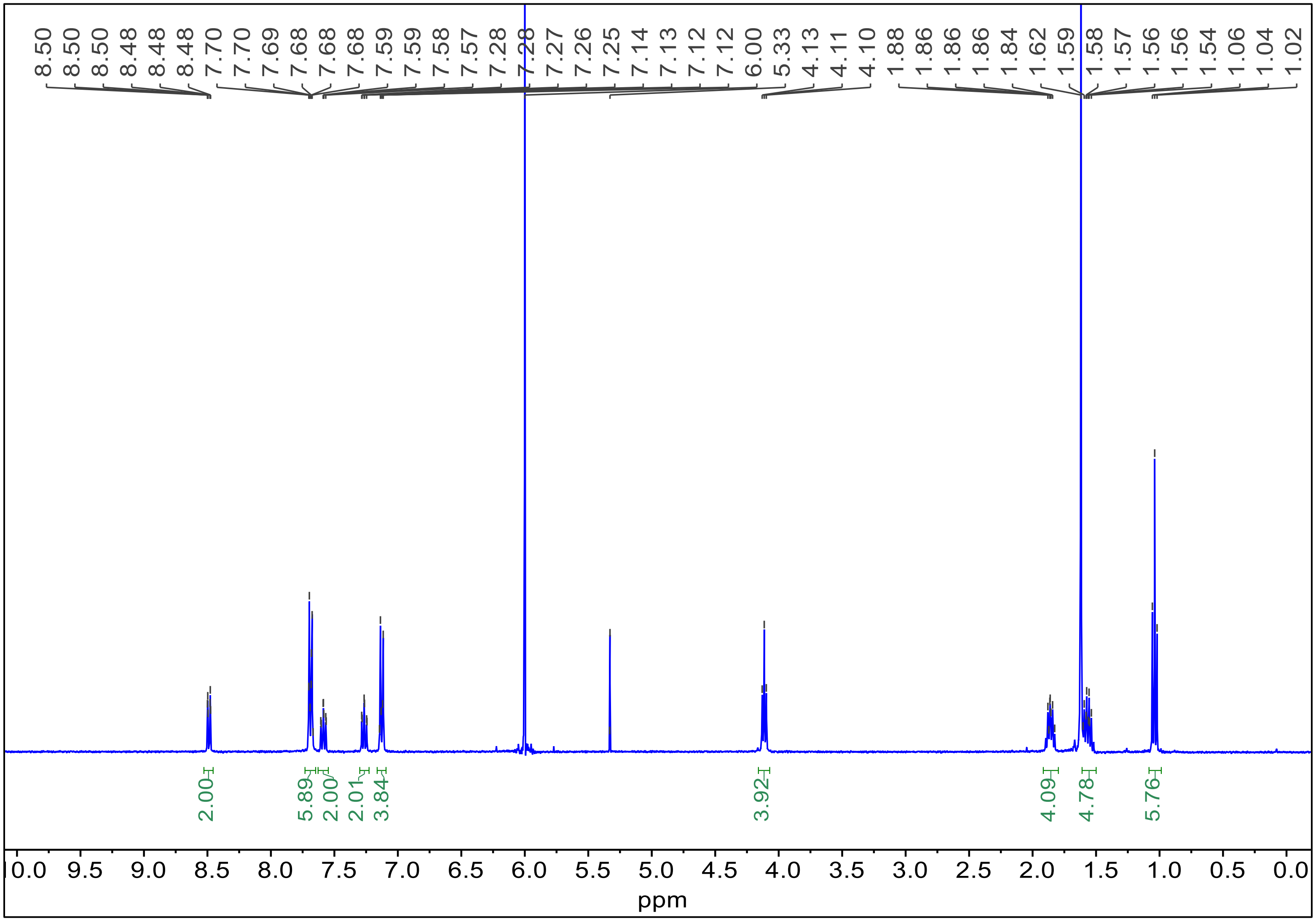

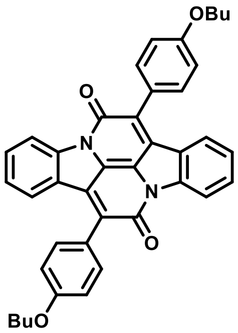

^{1}H NMR (400 MHz, $Cl_2CDCDCl_2$) – Derivative 2 – AA'XX' splitting patterns

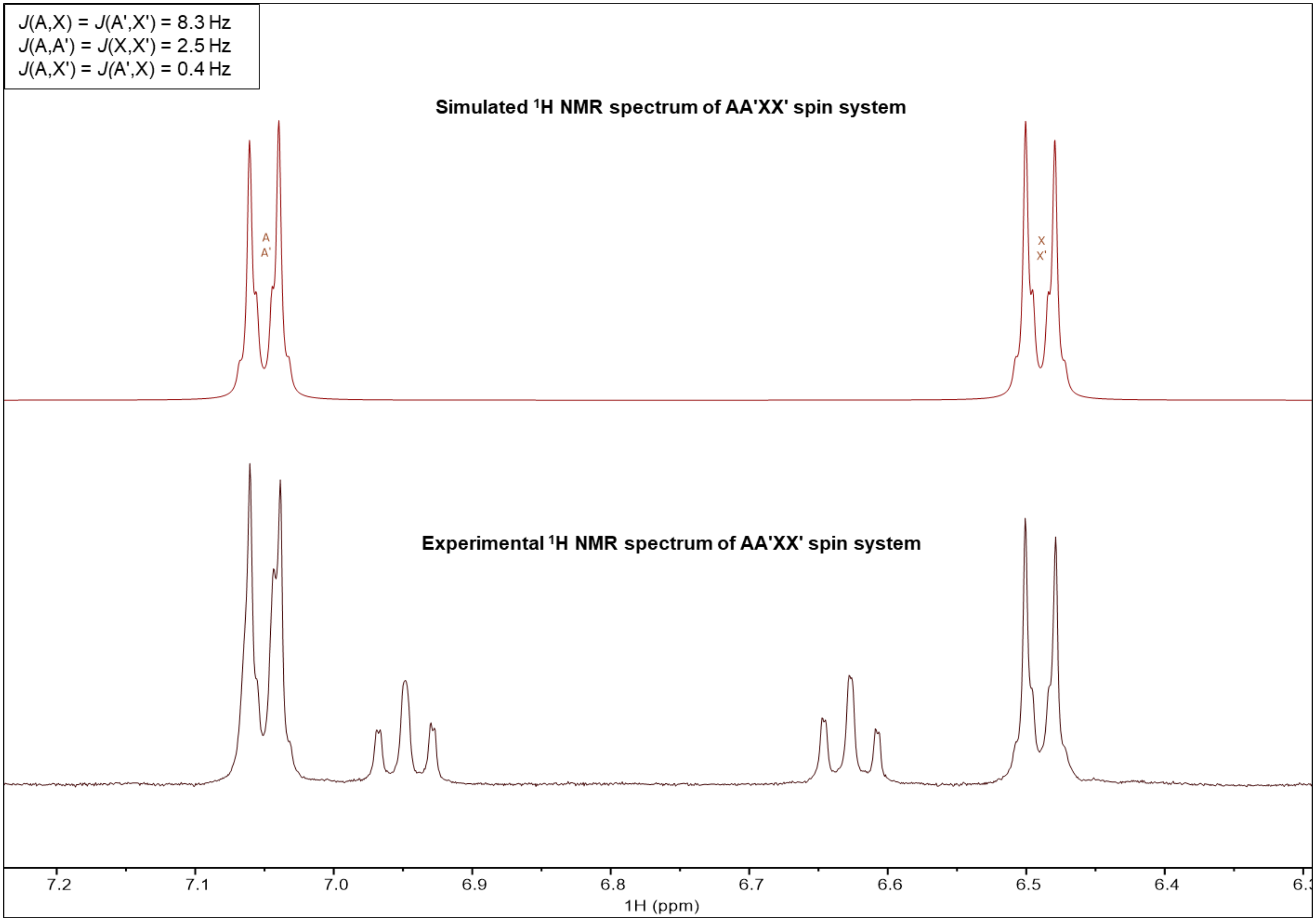

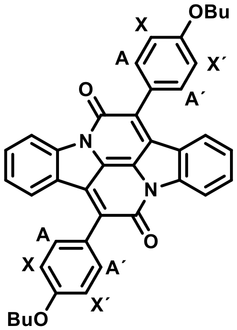

$^{13}C\{^{1}H\}$ APT NMR (125 MHz, $Cl_2CDCDCl_2$) – Derivative 2

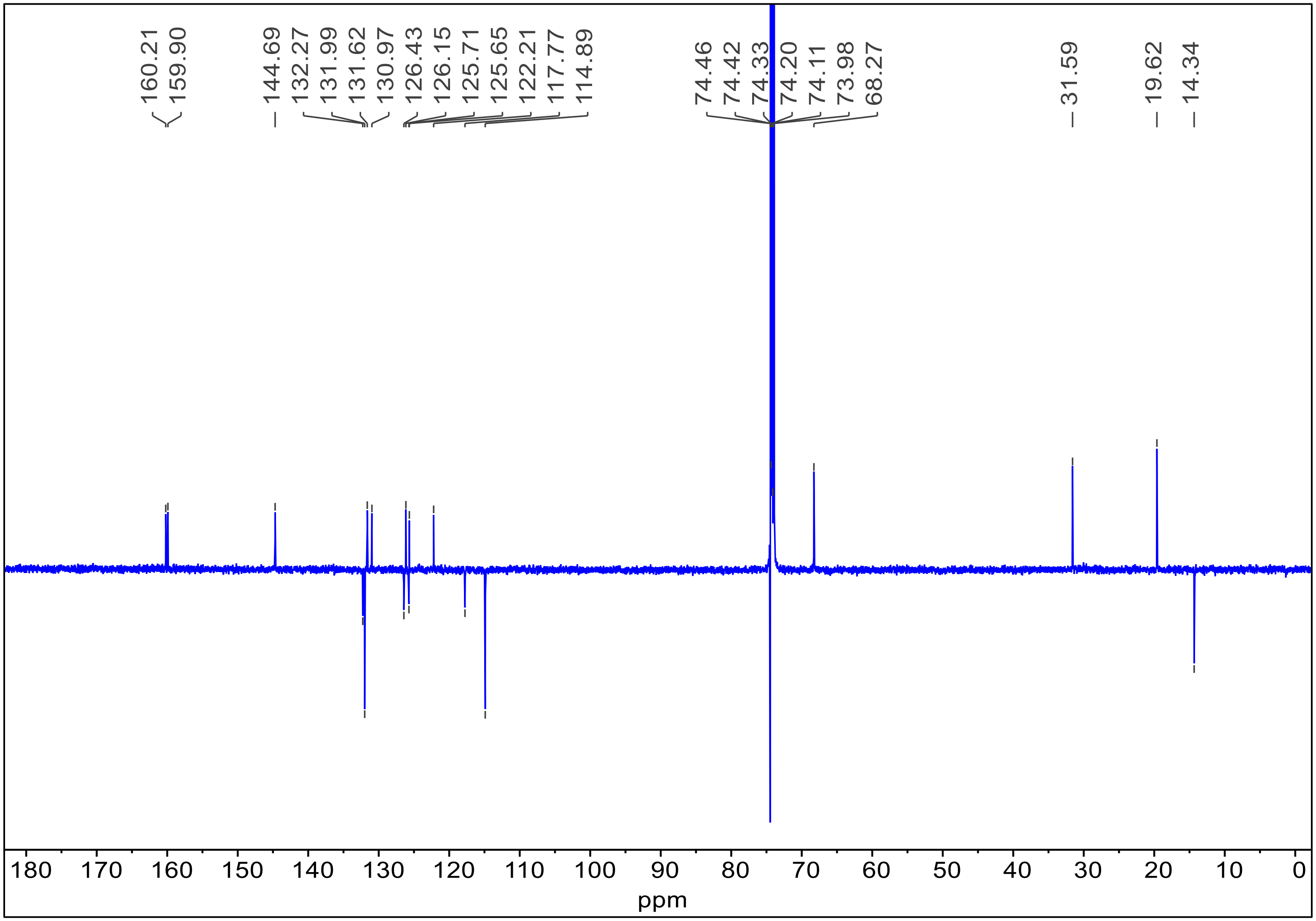

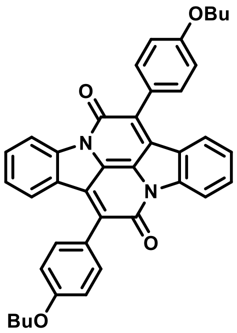

^{1}H – ^{1}H COSY ($Cl_2CDCDCl_2$) – Derivative 2

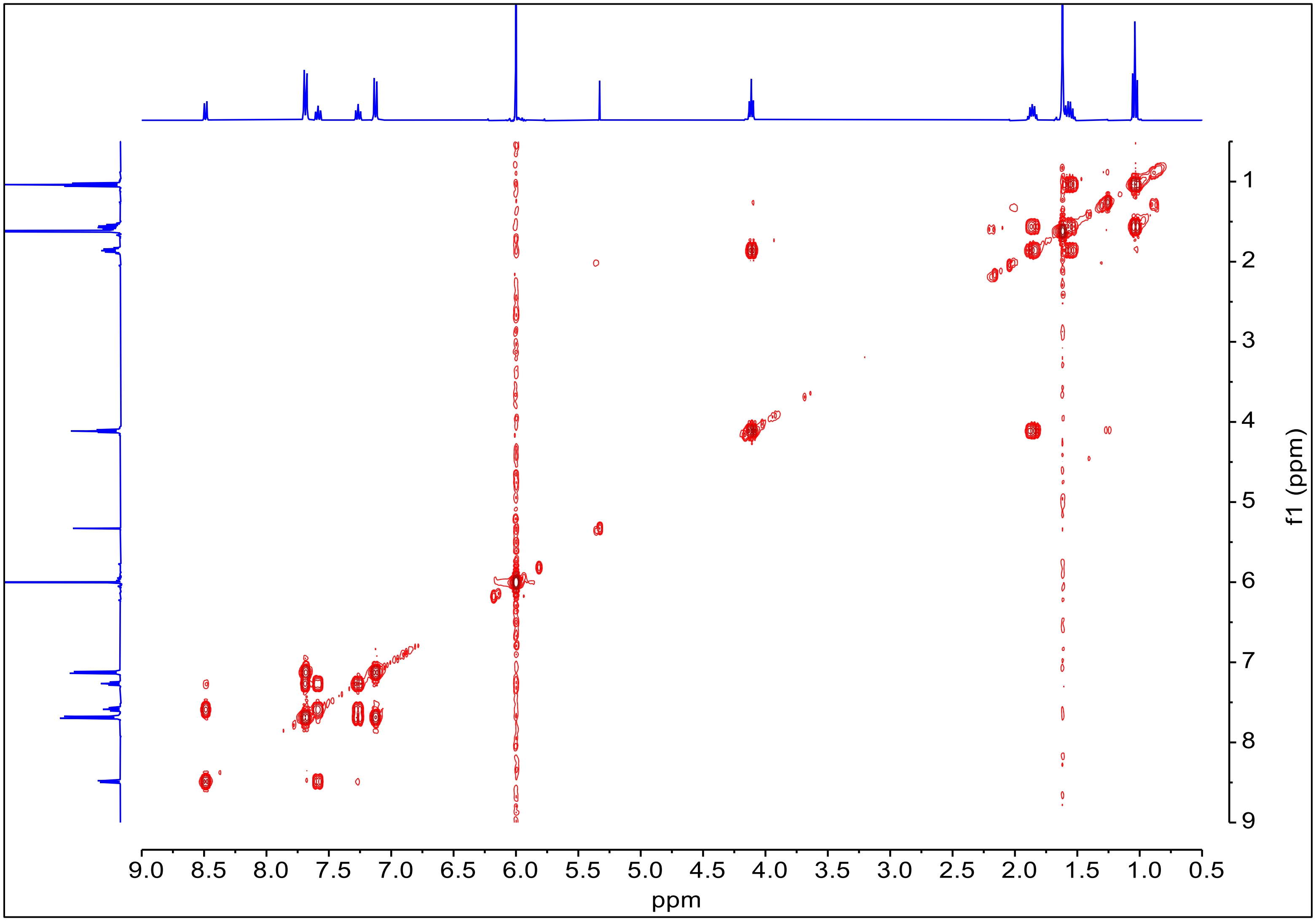

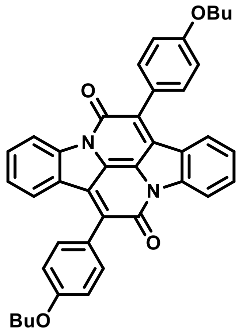

HSQC ($Cl_2CDCDCl_2$) – Derivative 2

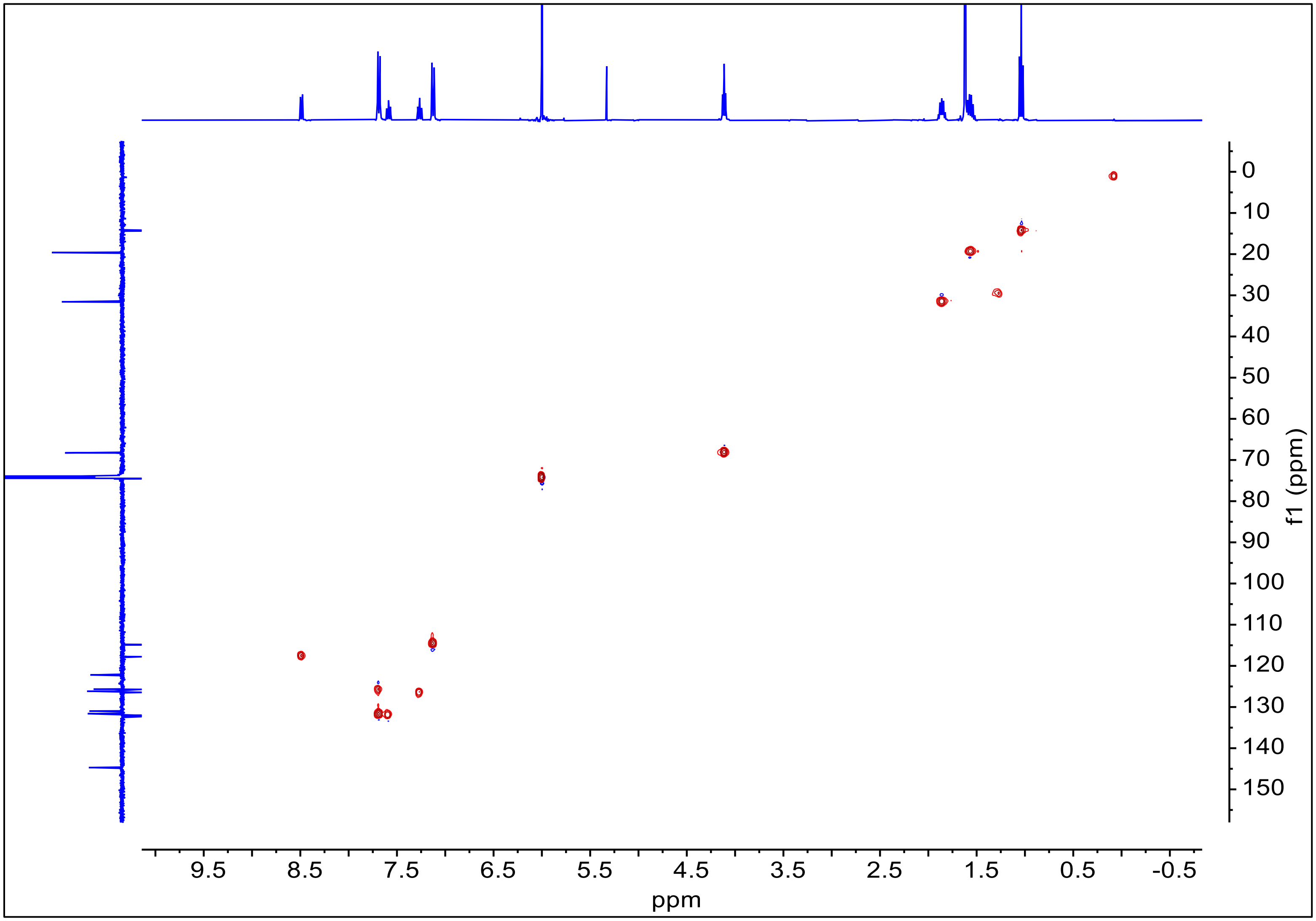

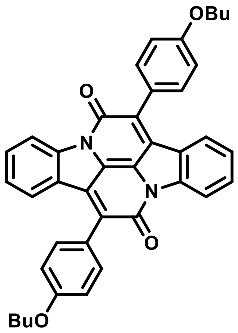

HMBC ($Cl_2CDCDCl_2$) – Derivative 2

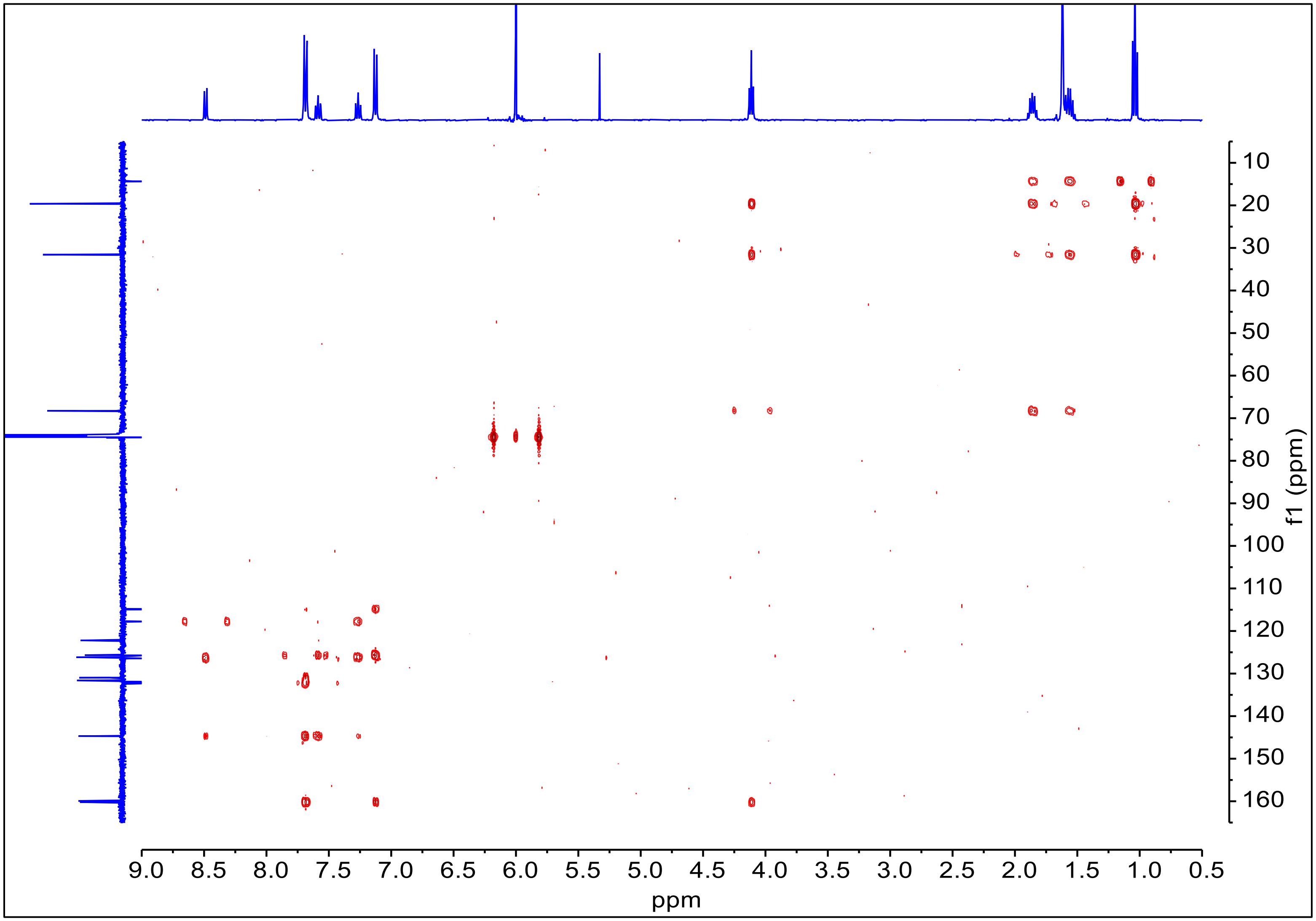

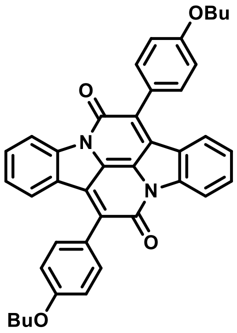

^{1}H NMR (500 MHz, $CDCl_3$) – Derivative 3

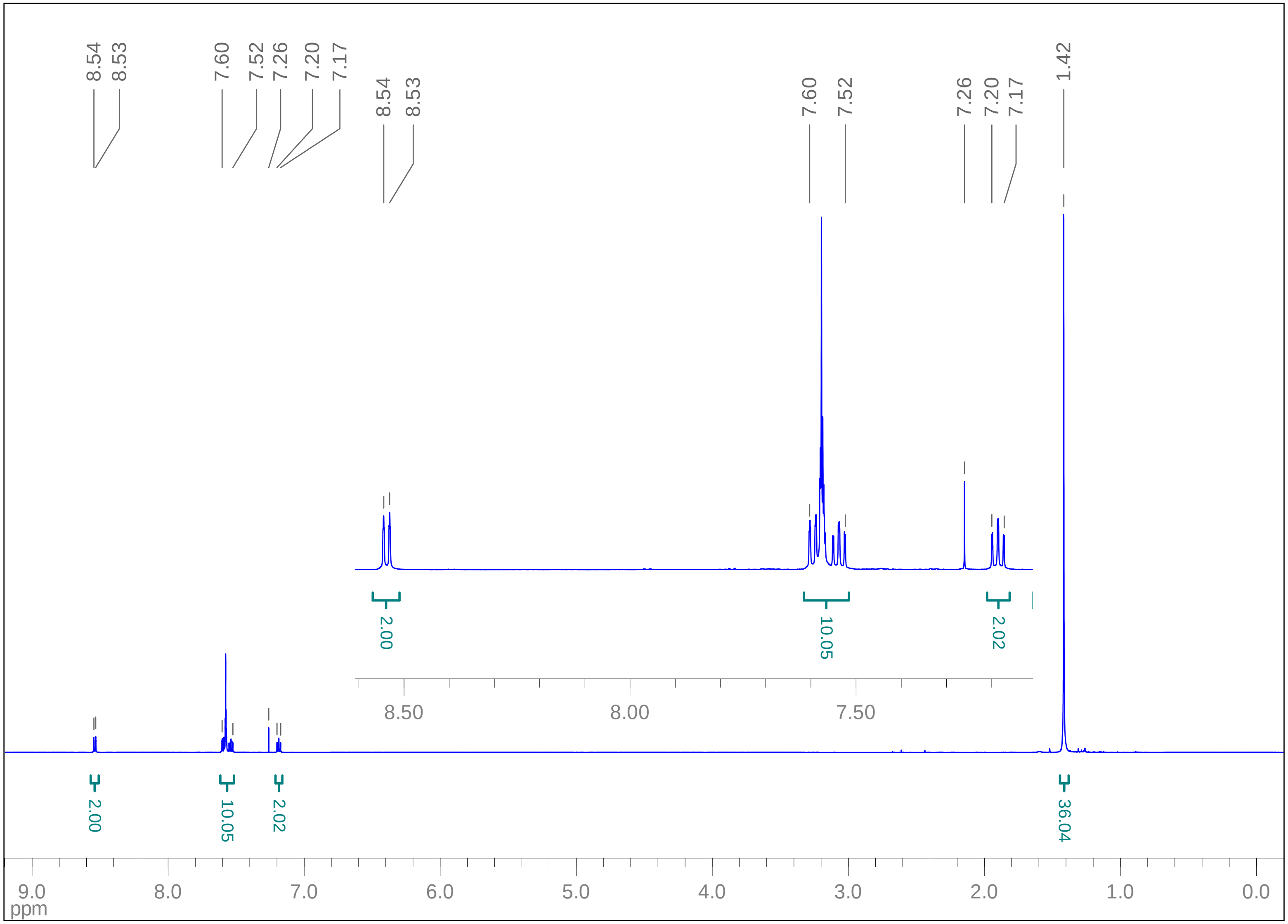

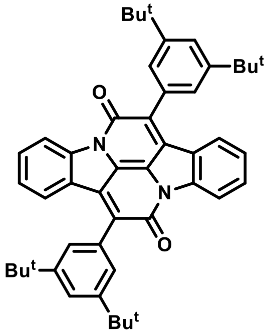

$^{13}C\{^1H\}$ NMR (100 MHz, $CDCl_3$) – Derivative 3

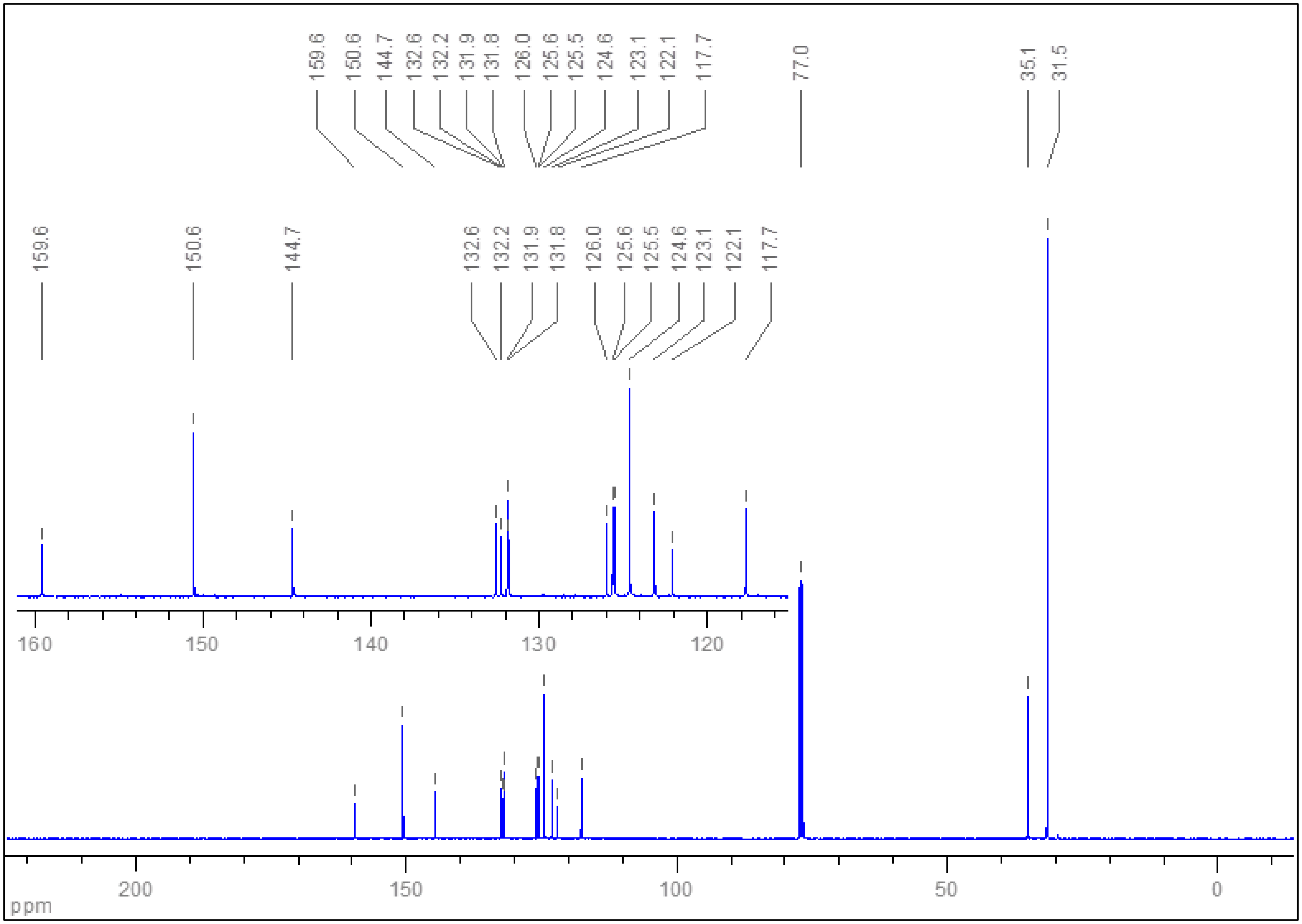

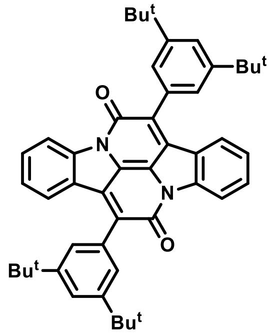

$^{13}C\{^{1}H\}$ NMR APT (100 MHz, $CDCl_3$) – Derivative 3

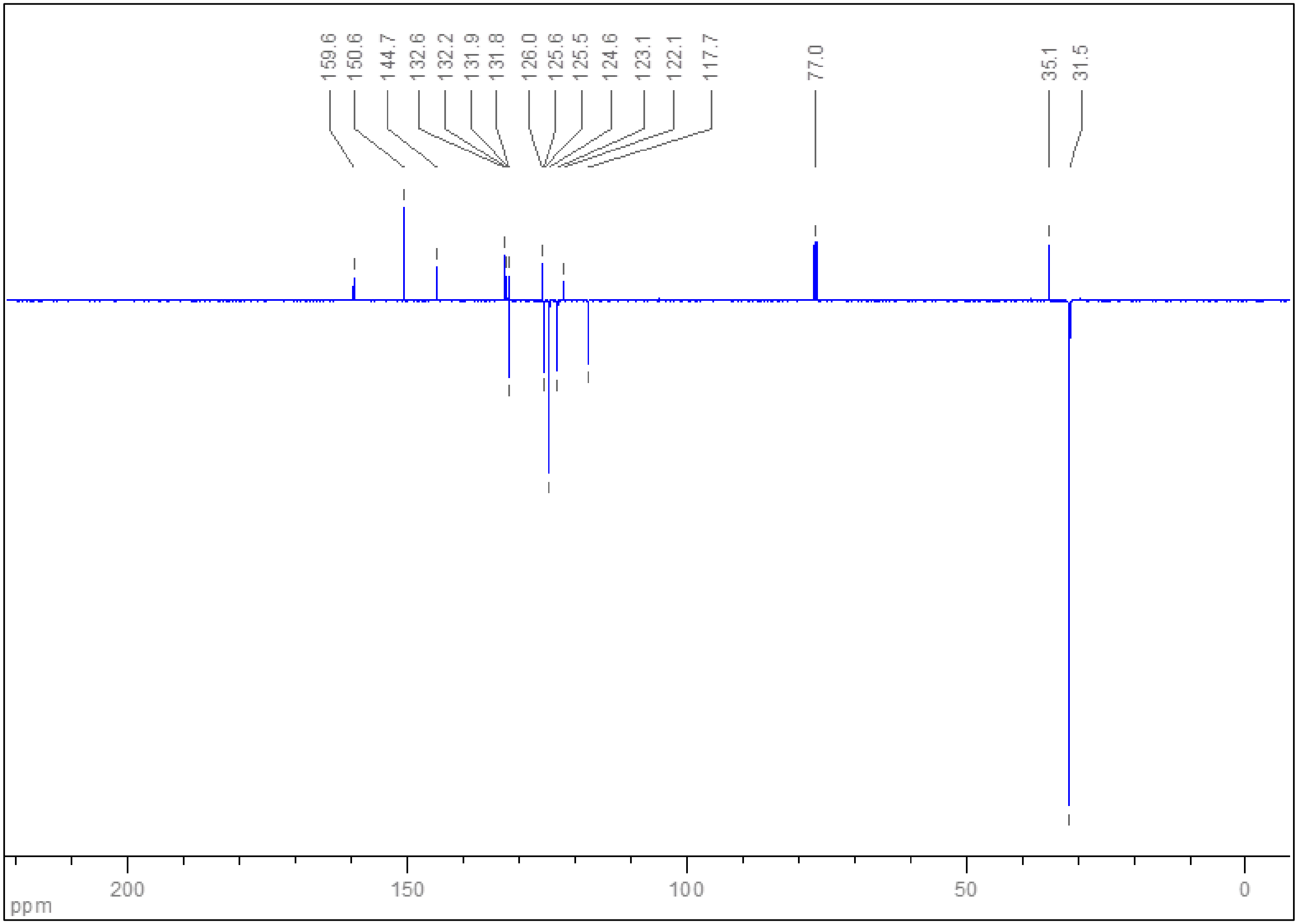

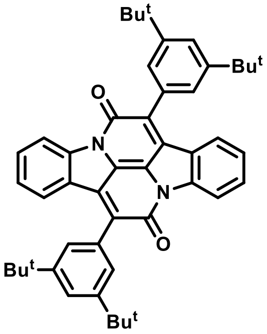

^{1}H – ^{1}H COSY ($CDCl_3$) – Derivative 3

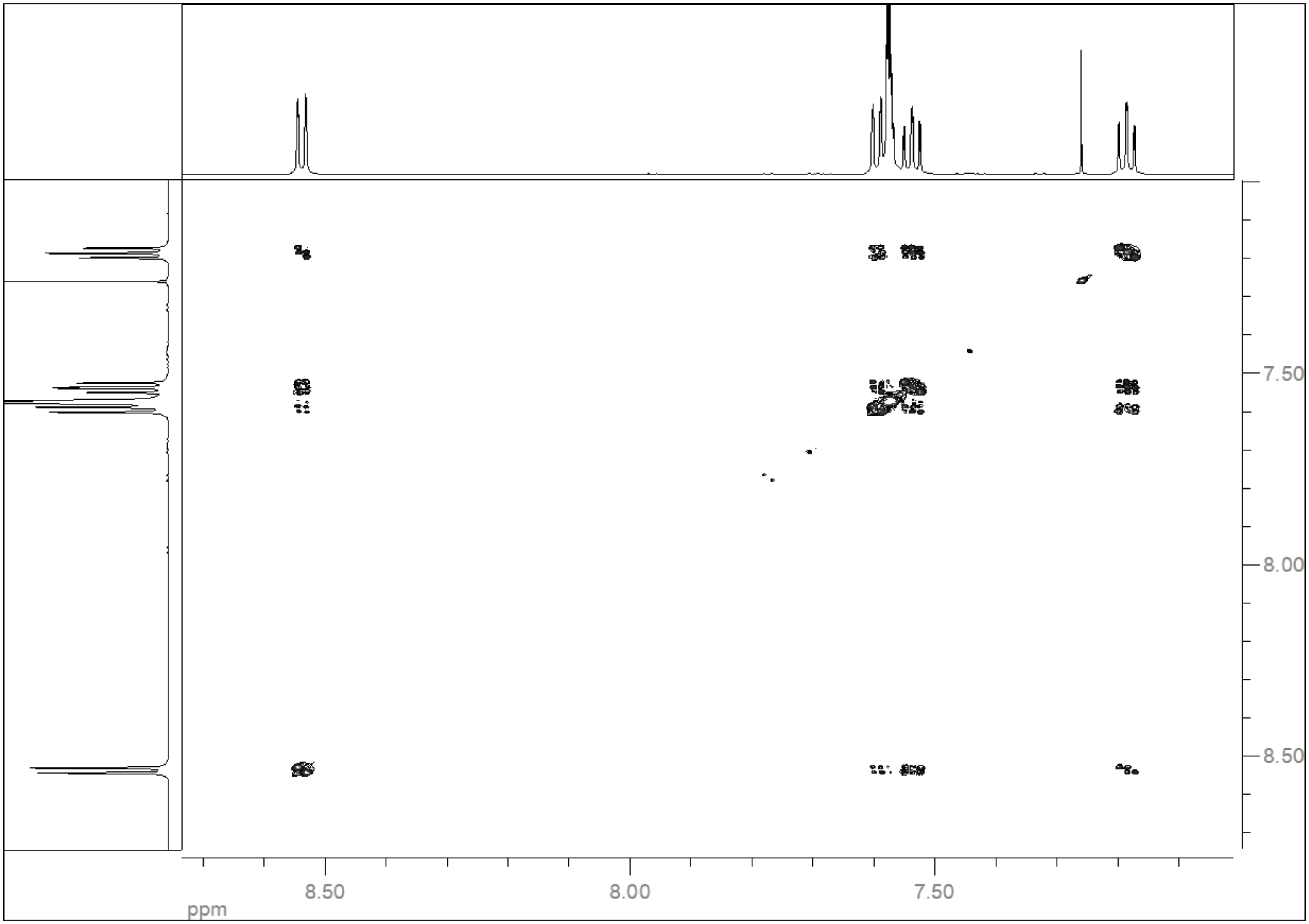

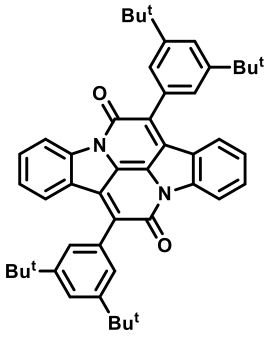

HSQC ($CDCl_3$) – Derivative 3

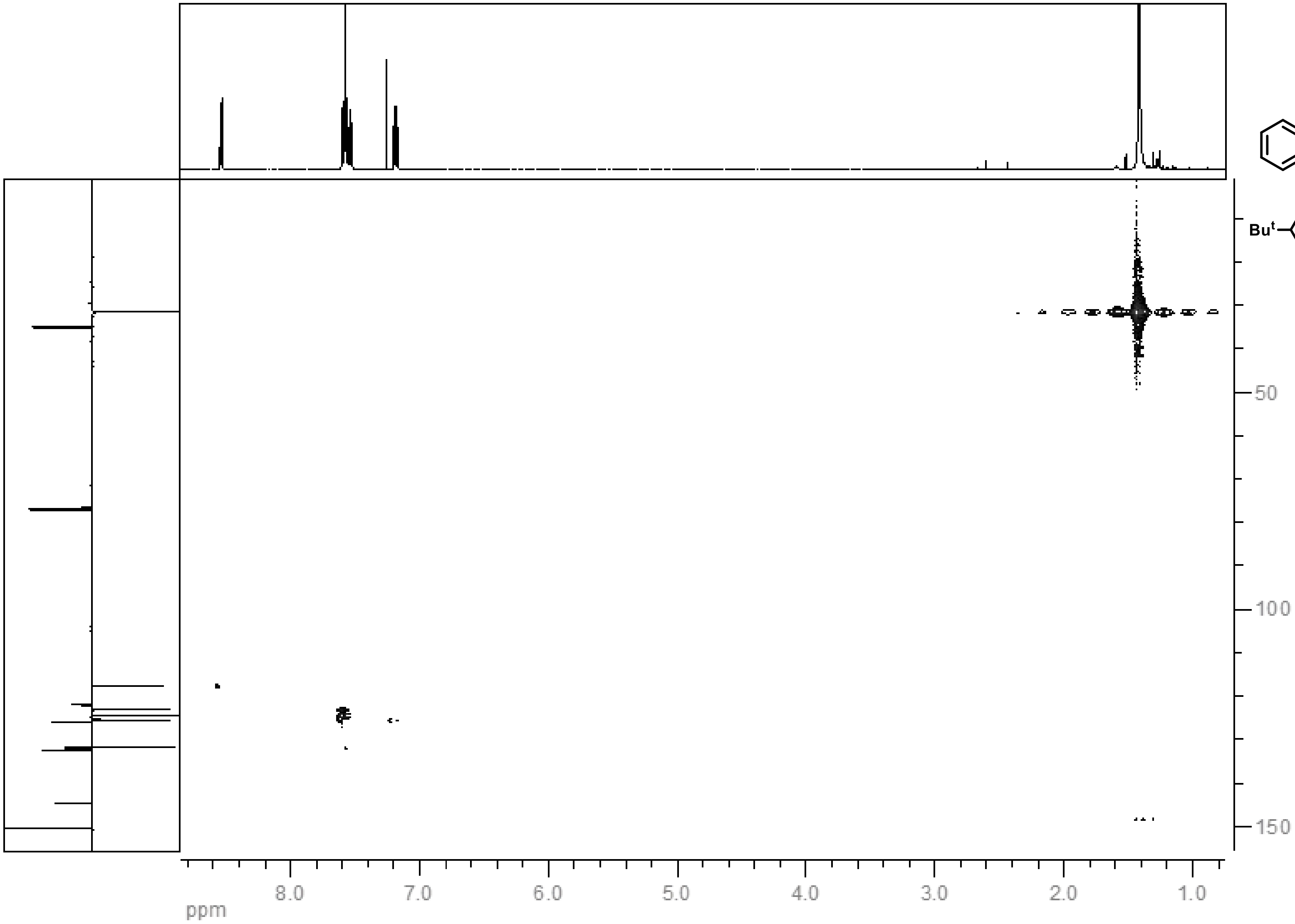

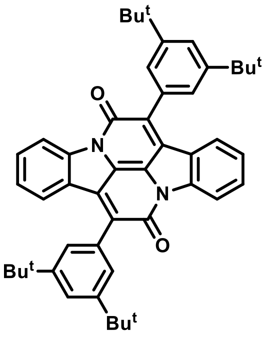

HMBC ($CDCl_3$) – Derivative 3

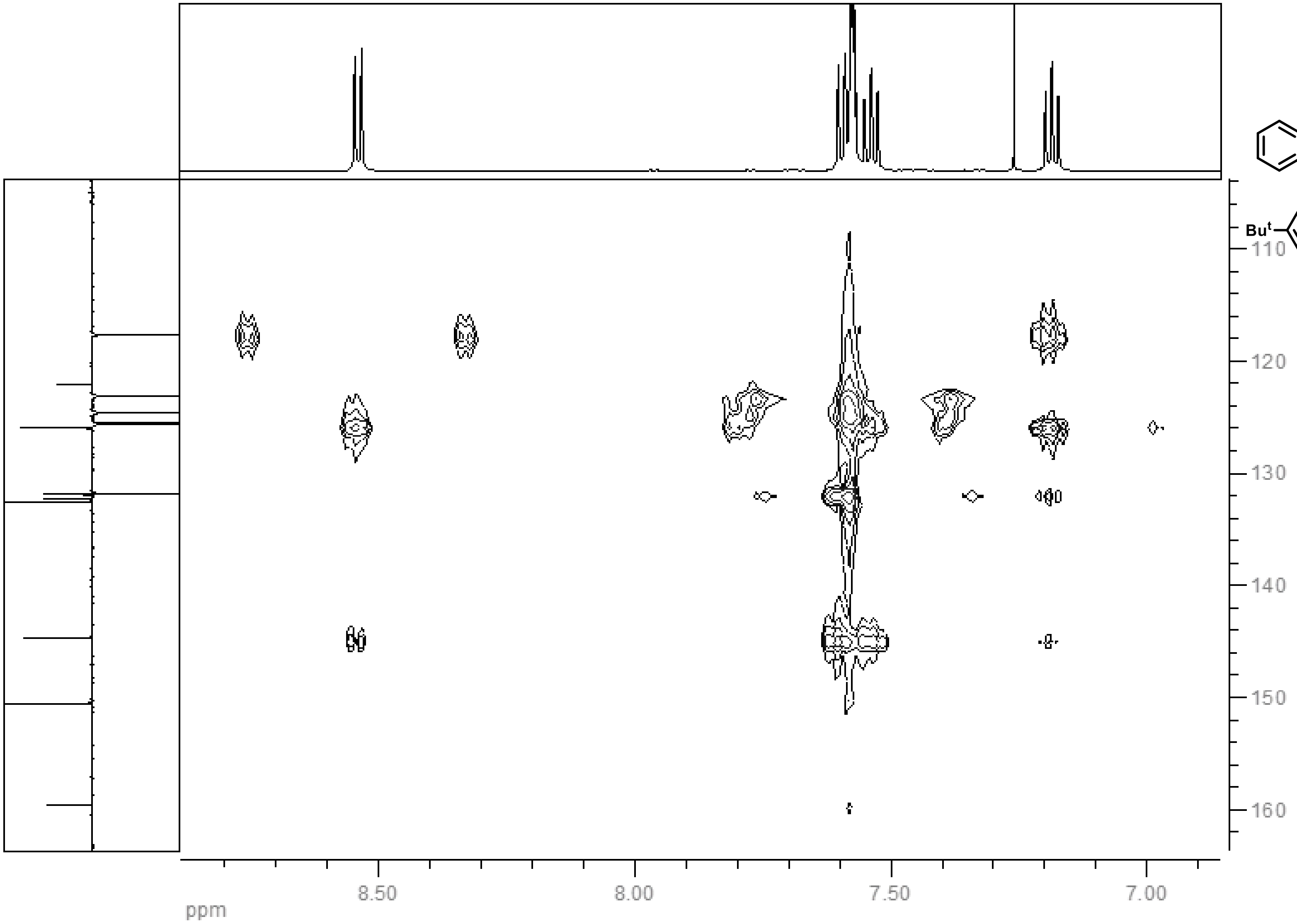

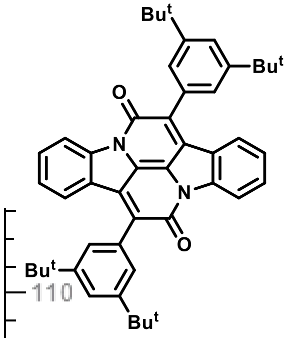

^{1}H NMR (400 MHz, $Cl_2CDCDCl_2$) – Derivative 4

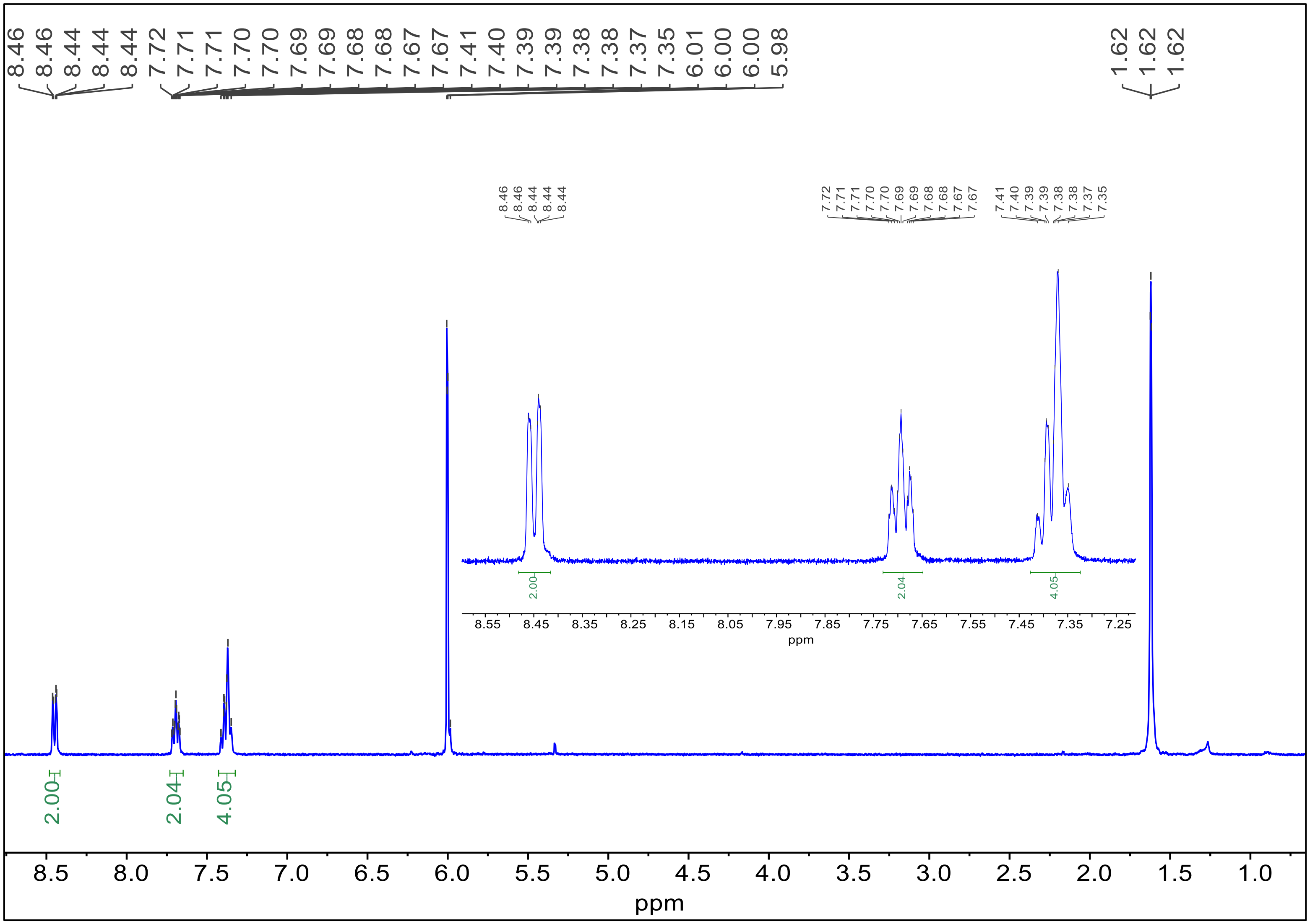

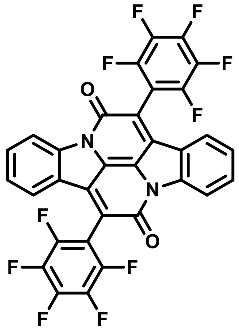

$^{13}C\{^1H\}$ APT NMR (125 MHz, $Cl_2CDCDCl_2$) – Derivative 4

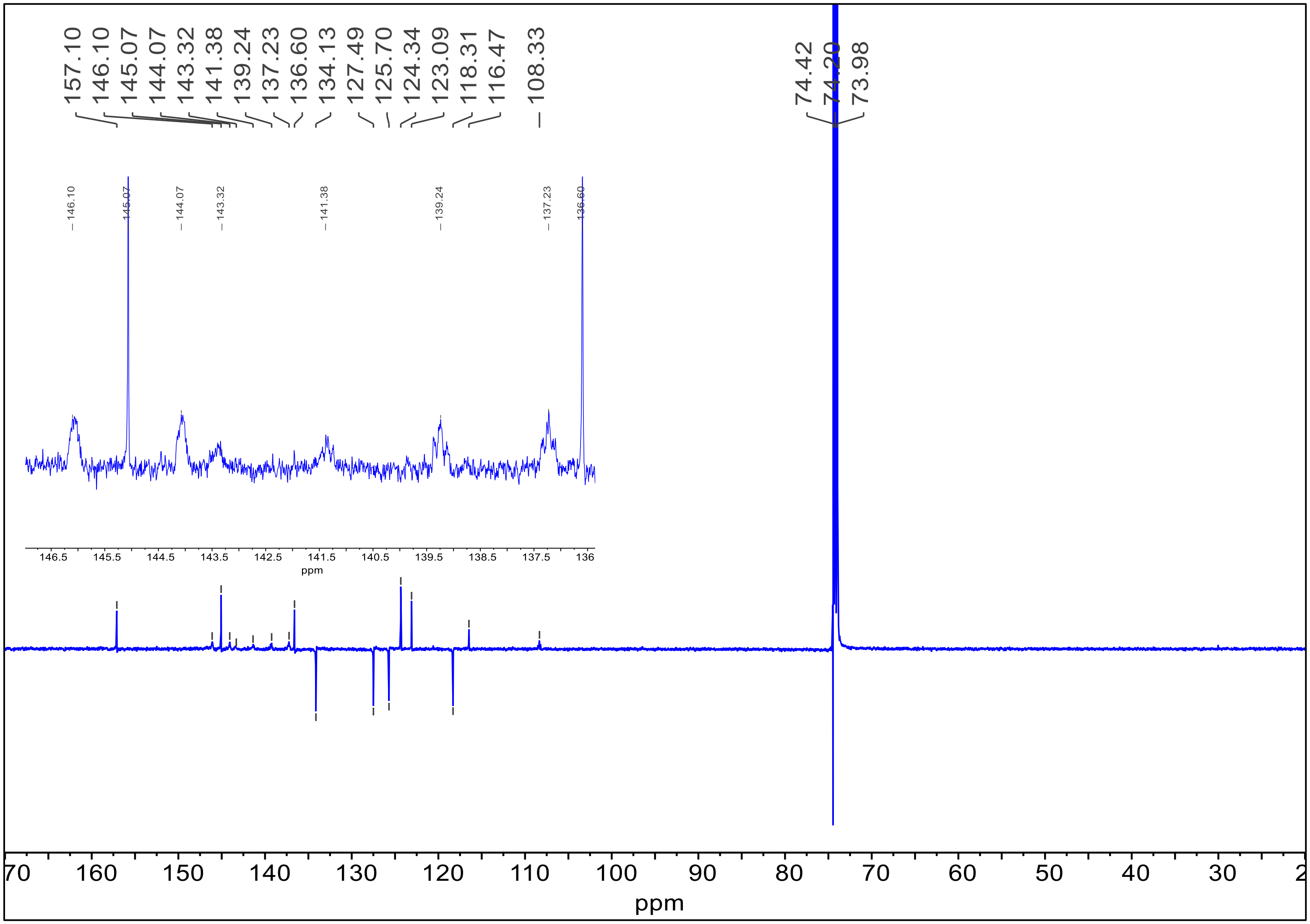

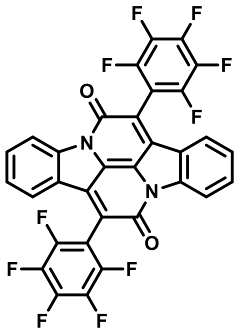

^{19}F NMR (376 MHz, $Cl_2CDCDCl_2$) – Derivative 4

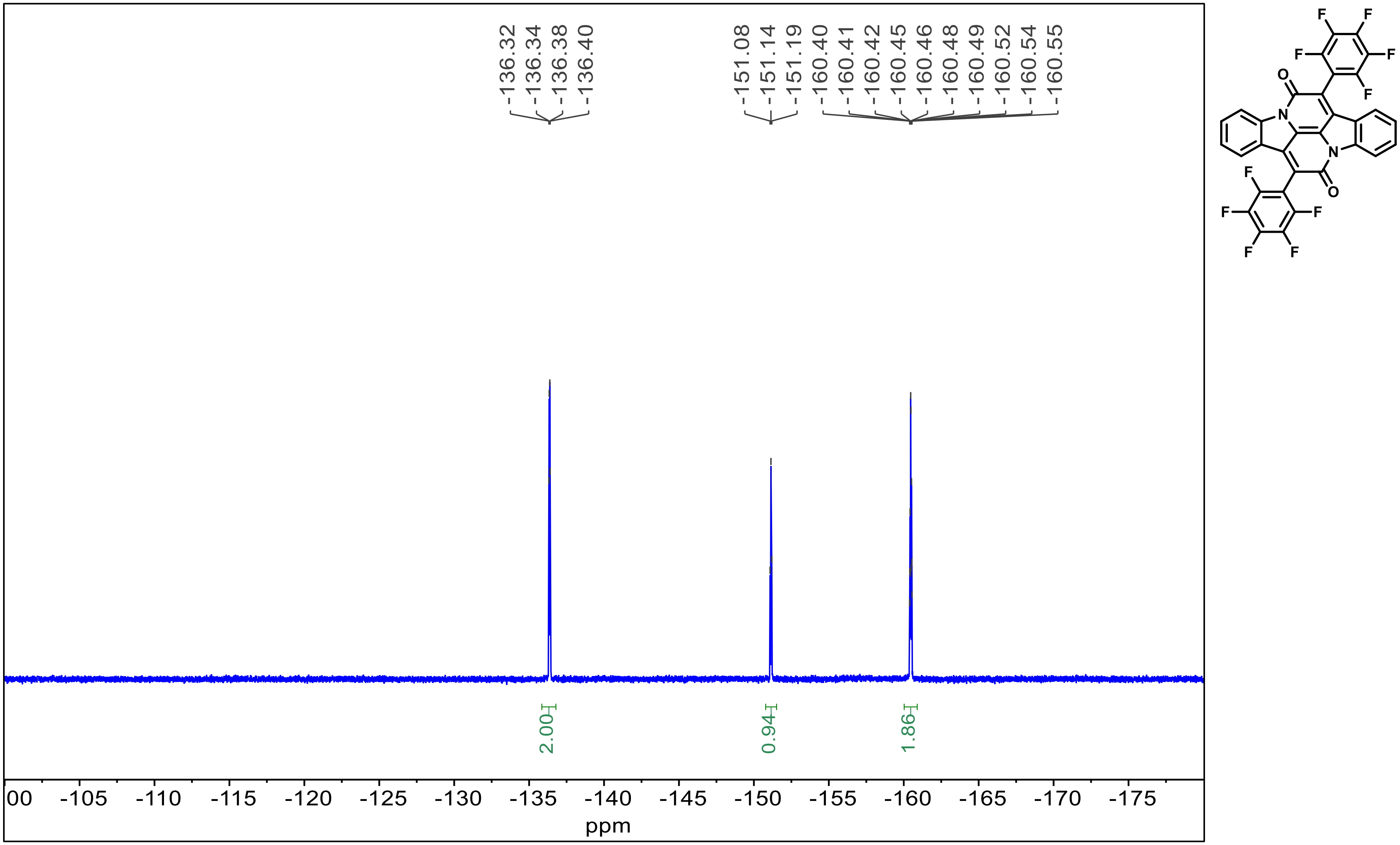

^{1}H – ^{1}H COSY ($Cl_2CDCDCl_2$) – Derivative 4

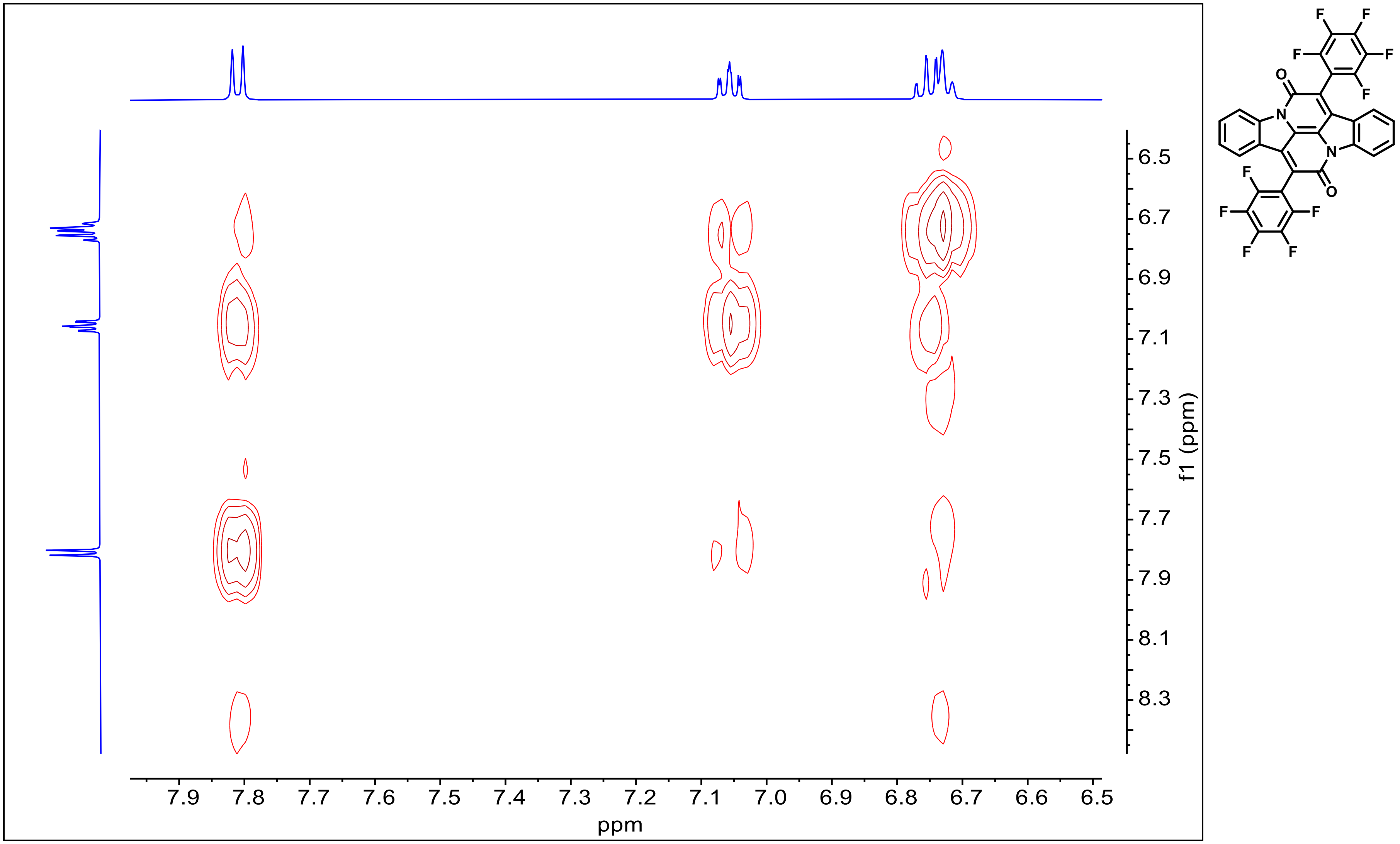

HSQC ($Cl_2CDCDCl_2$) – Derivative 4

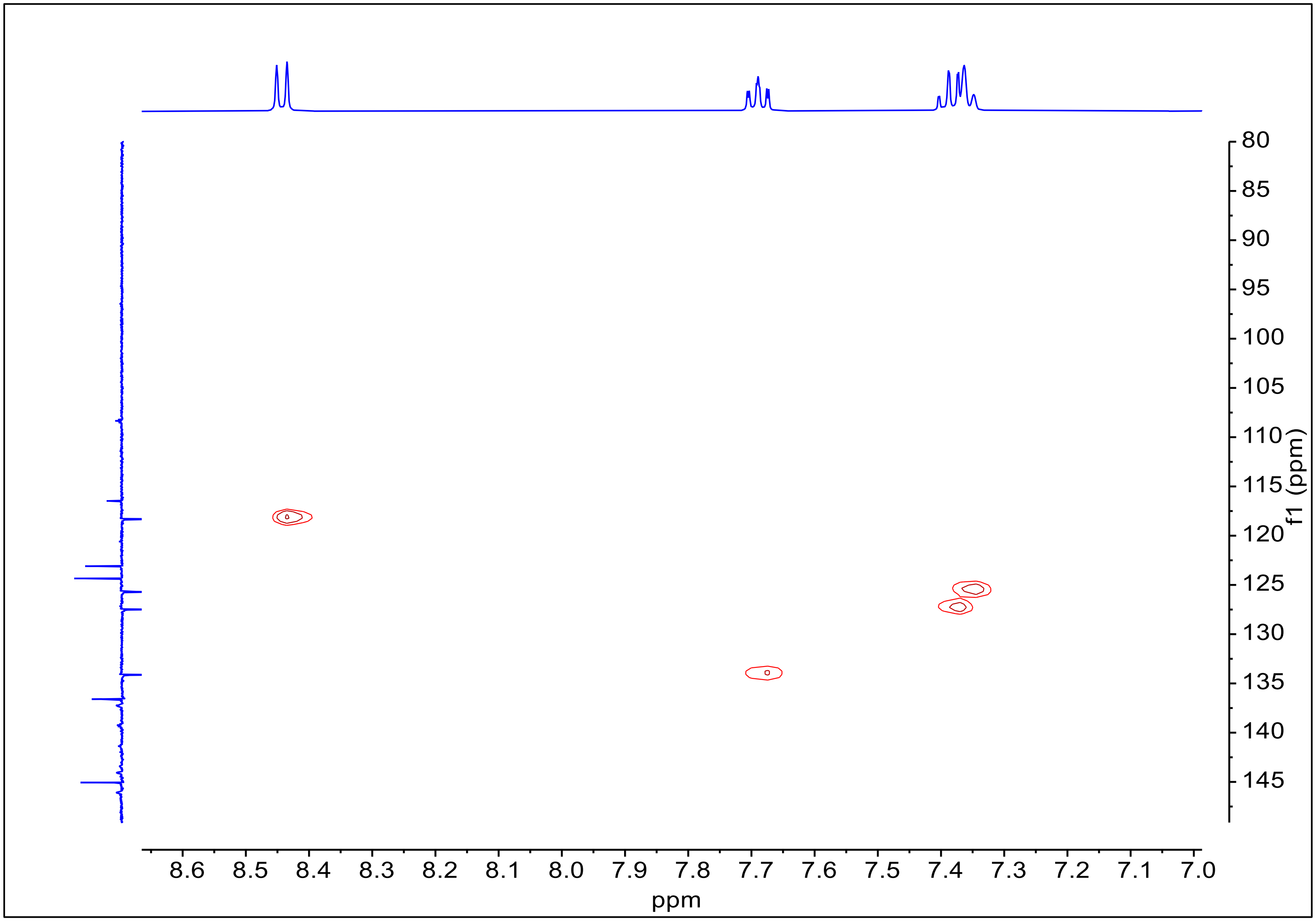

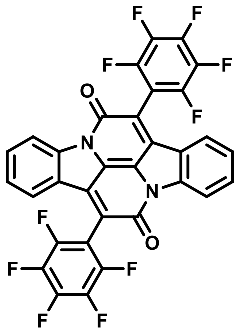

HMBC ($Cl_2CDCDCl_2$) – Derivative 4

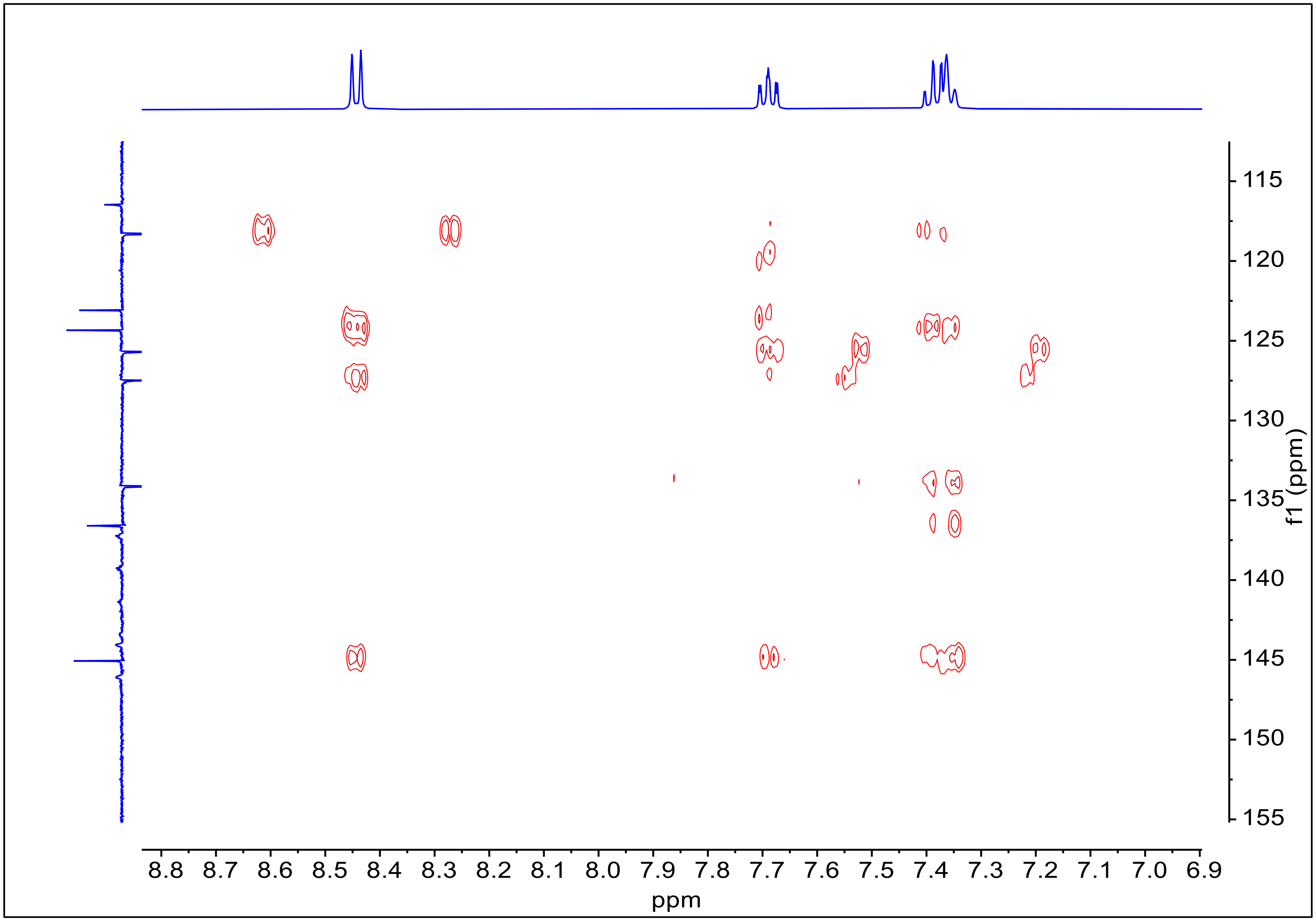

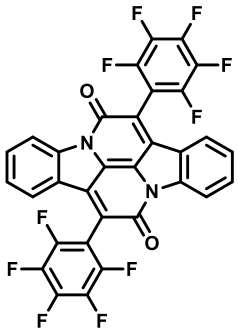

^{1}H NMR (500 MHz, CDCl$_3$) – Derivative 5

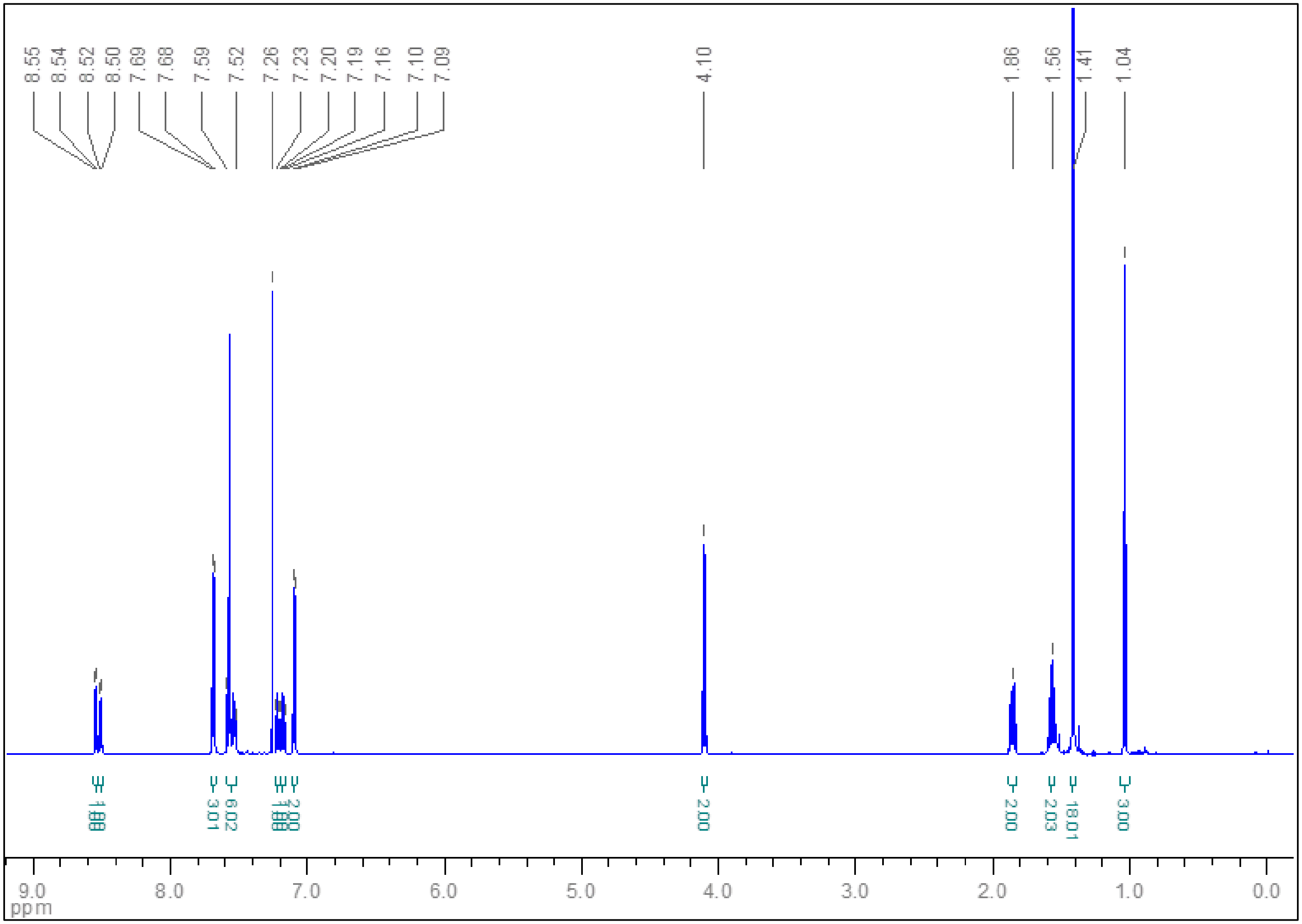

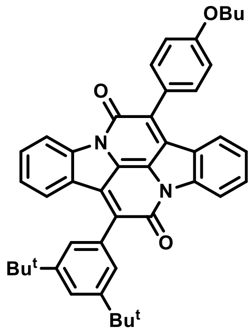

$^{13}C\{^1H\}$ NMR (100 MHz, $CDCl_3$) – Derivative 5

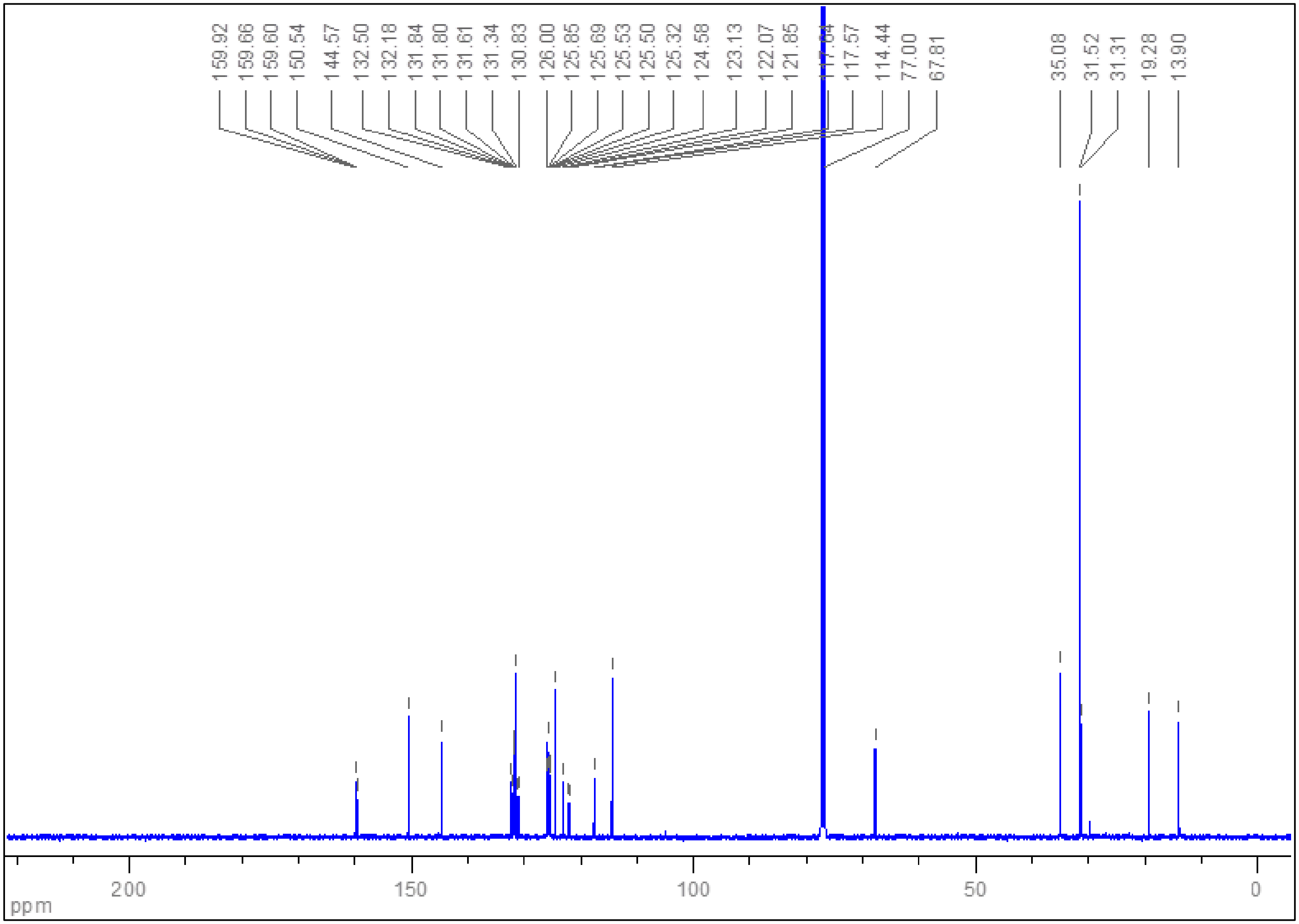

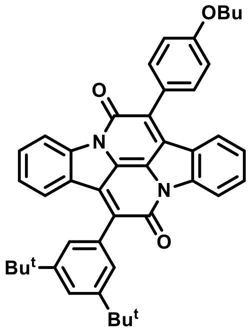

$^{13}C\{^1H\}$ NMR ATP (100 MHz, $CDCl_3$) – Derivative 5

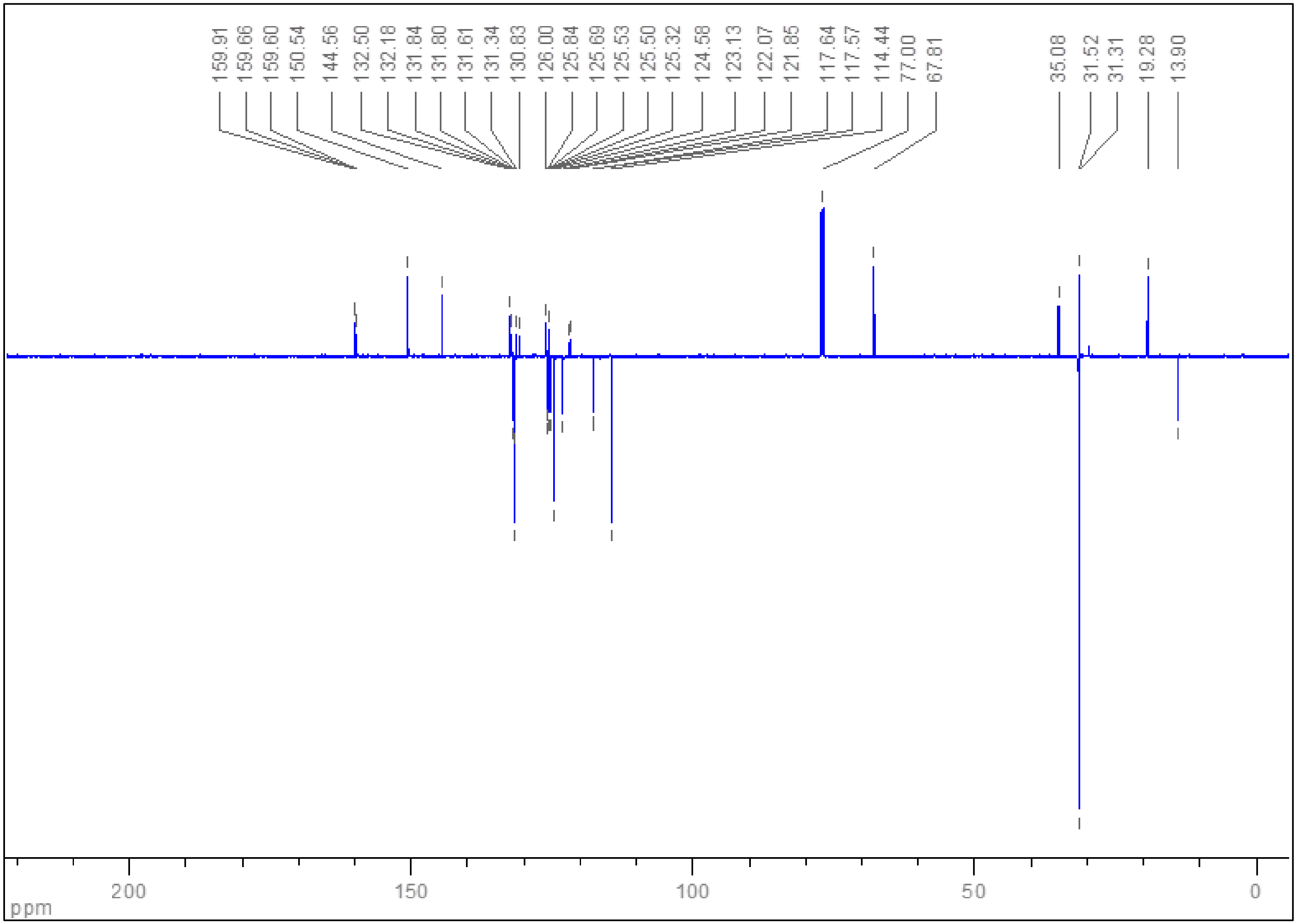

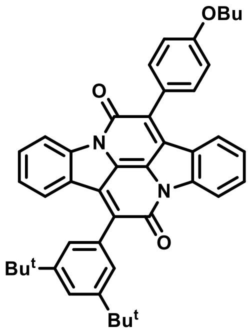

^{1}H – ^{1}H COSY ($CDCl_3$) – Derivative 5

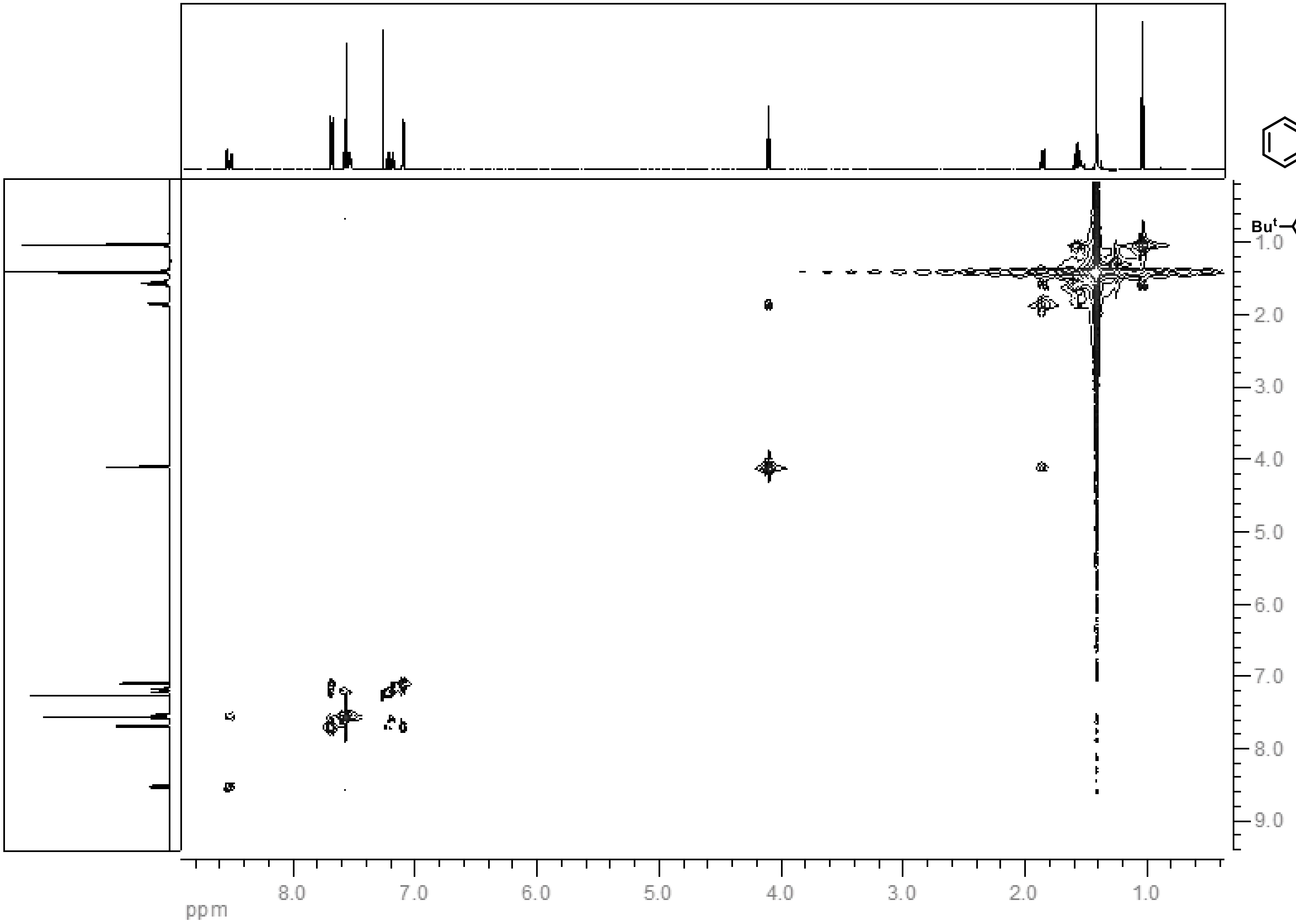

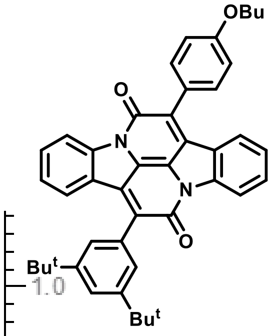

HSQC ($CDCl_3$) – Derivative 5

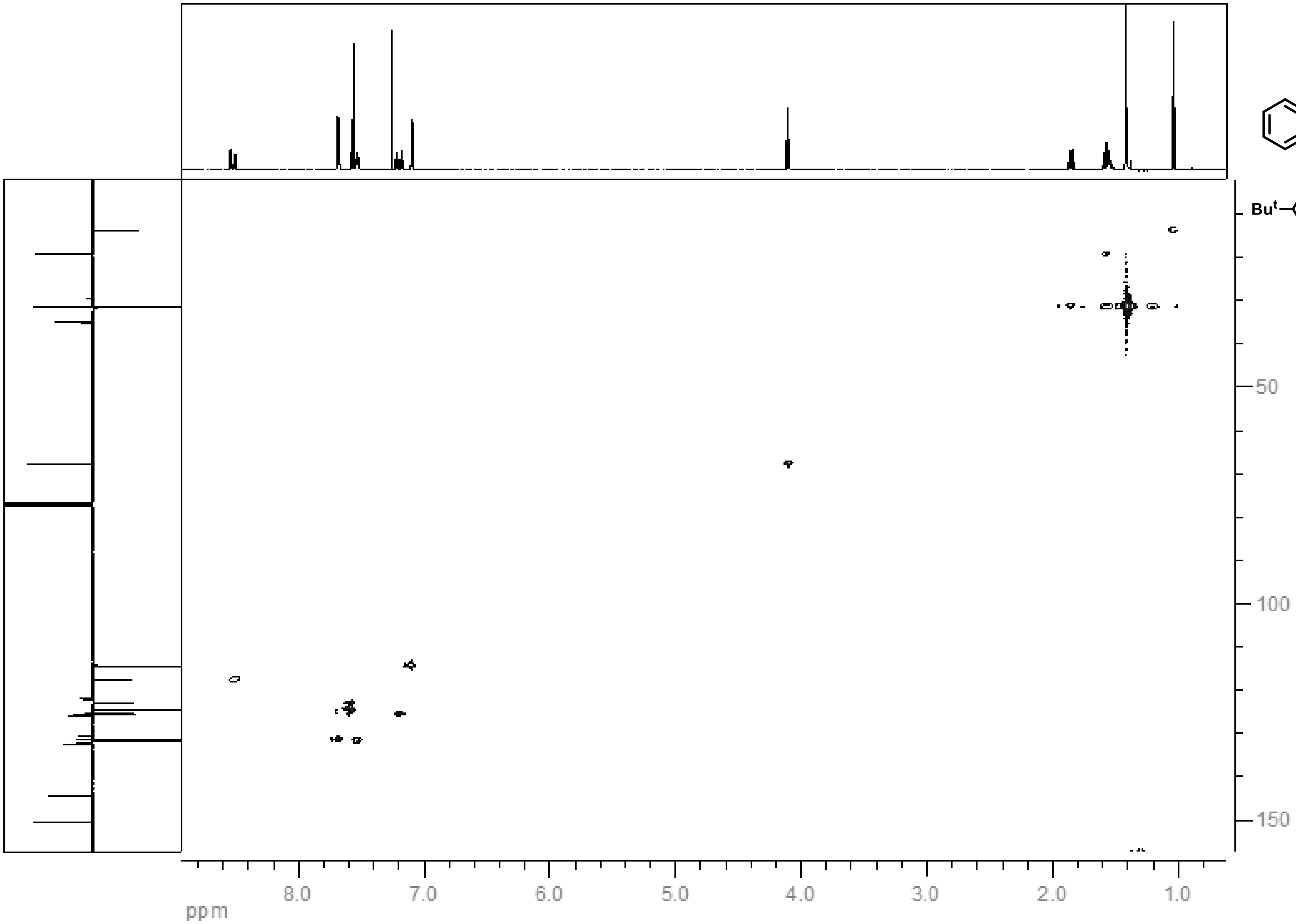

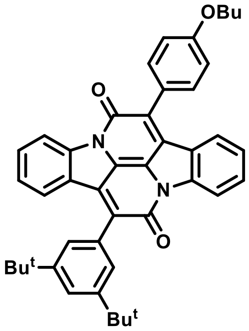

HMBC ($CDCl_3$) – Derivative 5

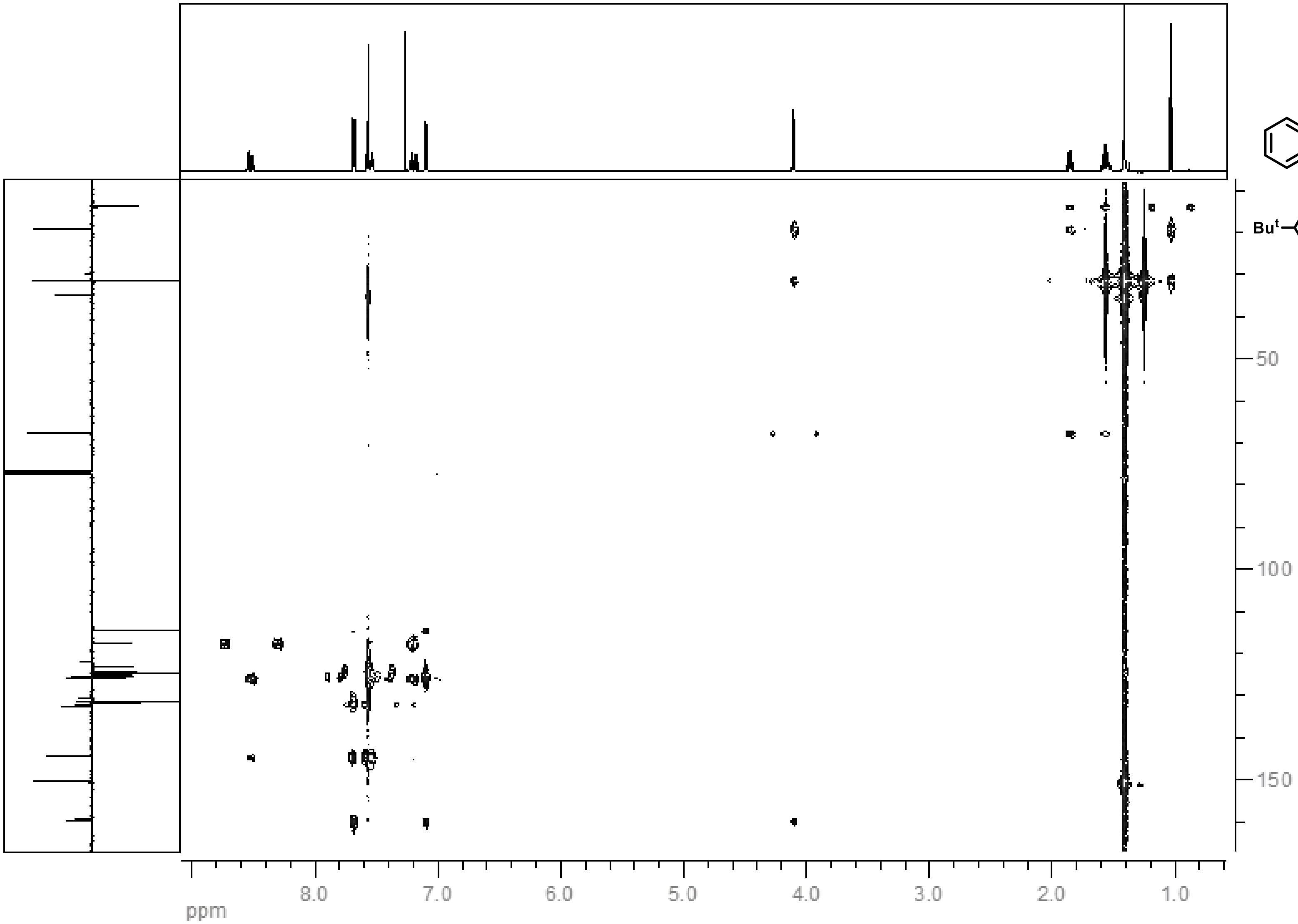

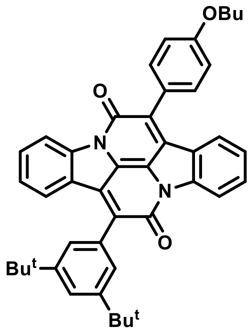

^{1}H NMR (600 MHz, $Cl_2CDCDCl_2$) – Derivative 6

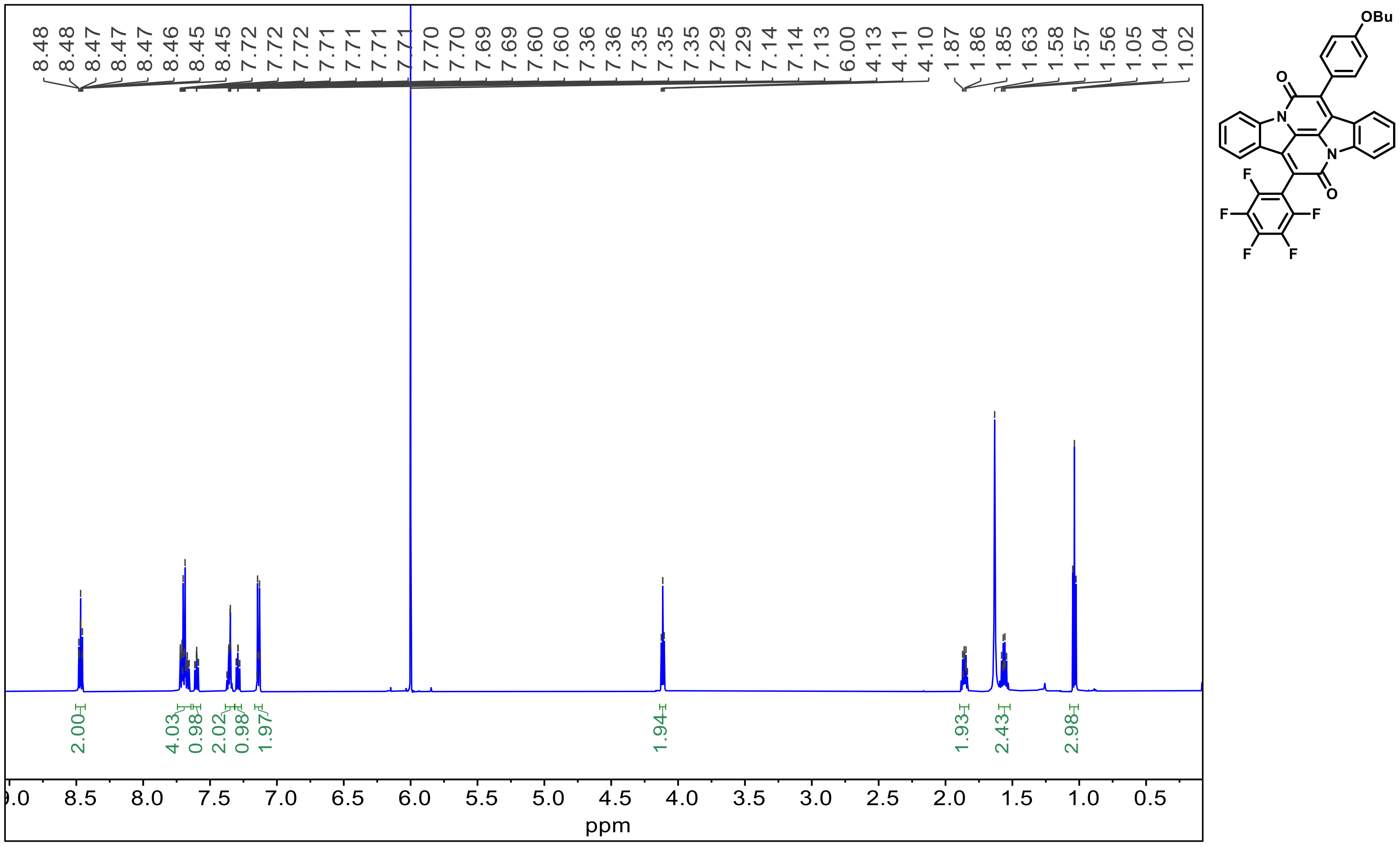

$^{13}C\{^{1}H\}$ NMR APT (150 MHz, $Cl_2CDCDCl_2$) – Derivative 6

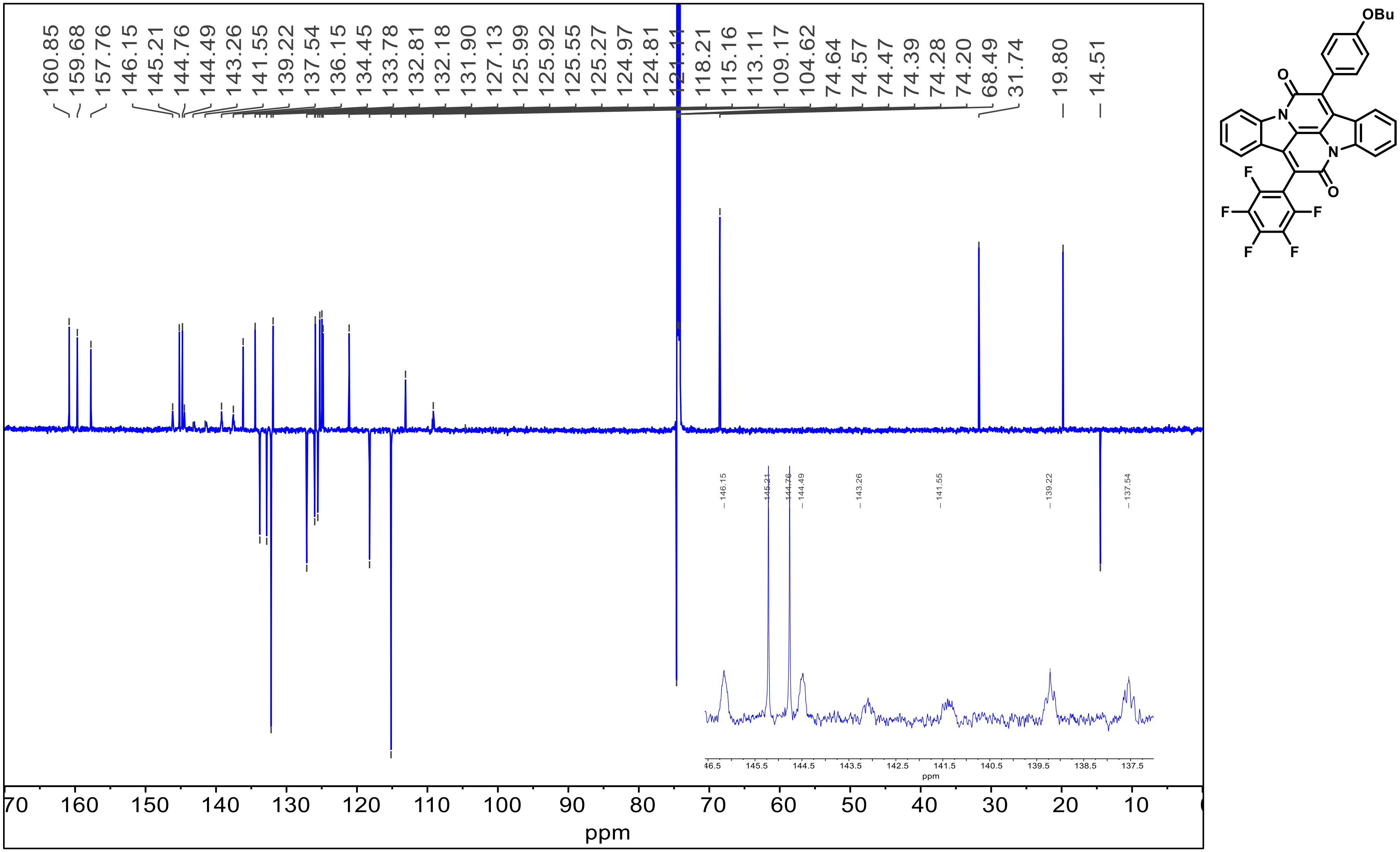

^{19}F NMR (470 MHz, $Cl_2CDCDCl_2$) – Derivative 6

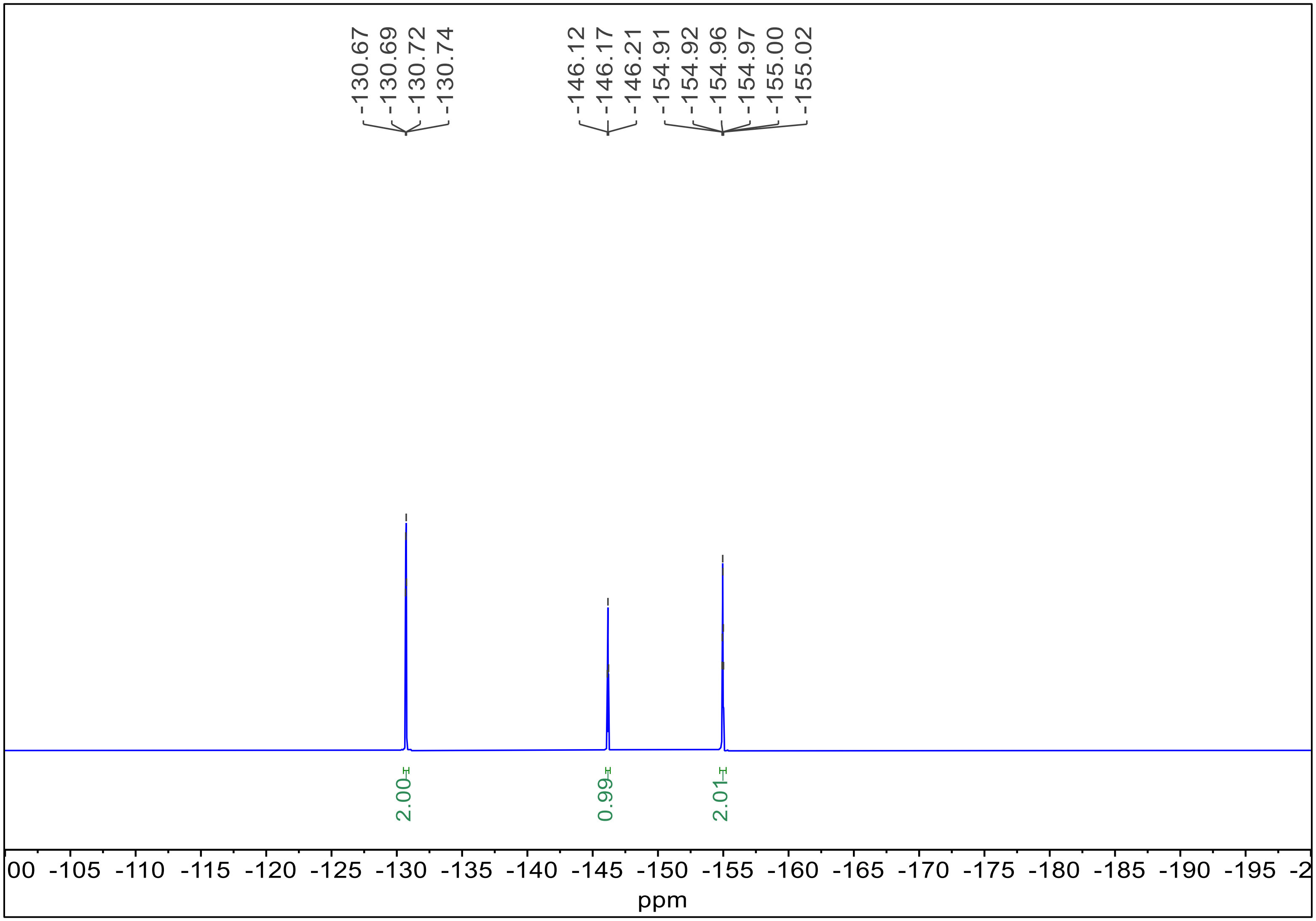

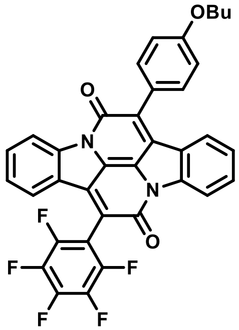

^{1}H – ^{1}H COSY ($Cl_2CDCDCl_2$) – Derivative 6

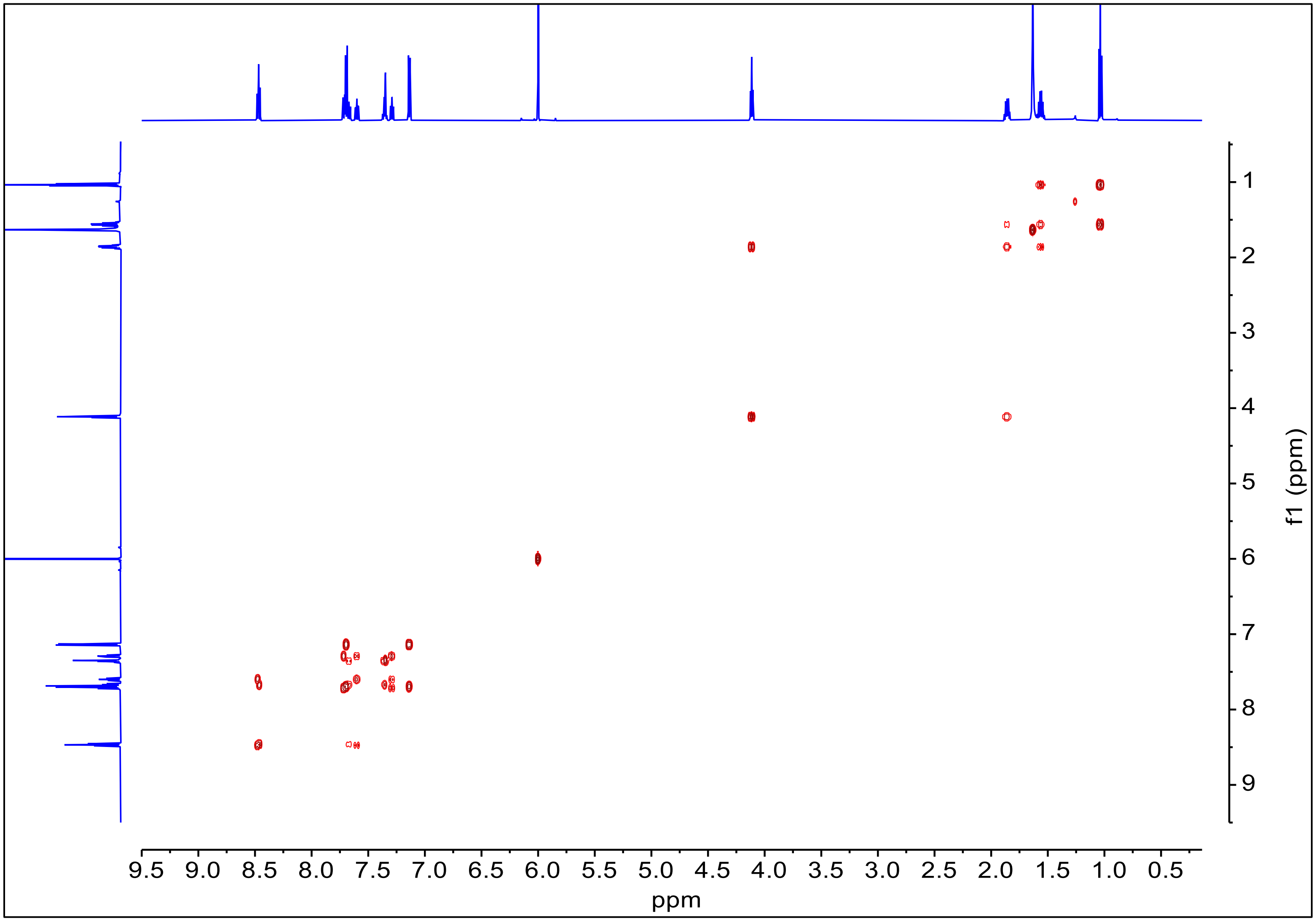

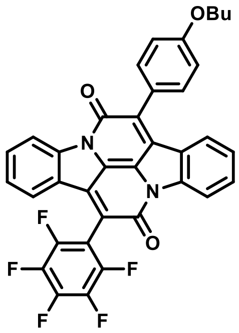

HSQC ($Cl_2CDCDCl_2$) – Derivative 6

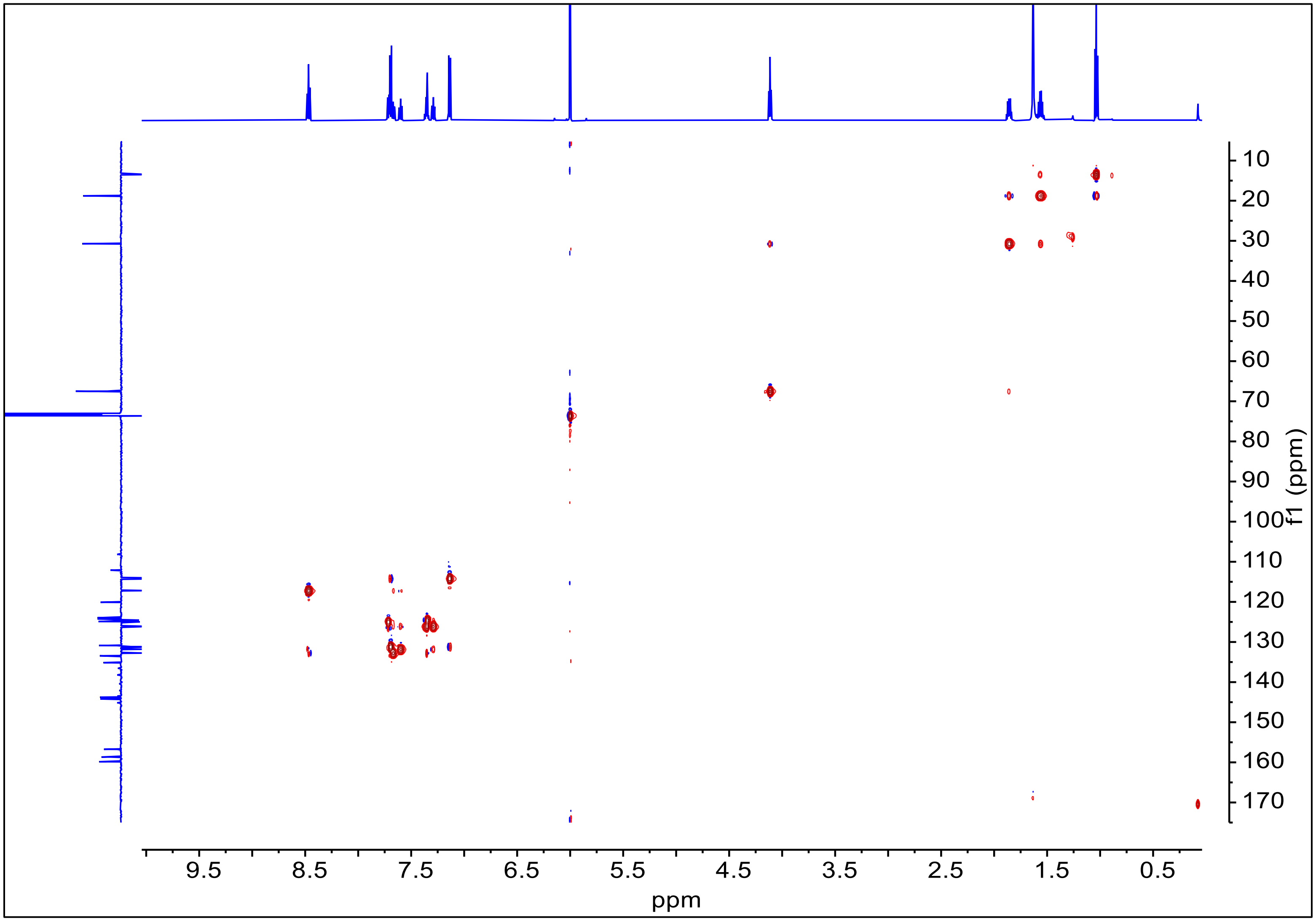

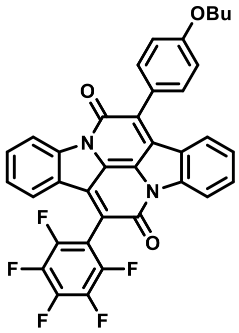

HMBC ($Cl_2CDCDCl_2$) – Derivative 6

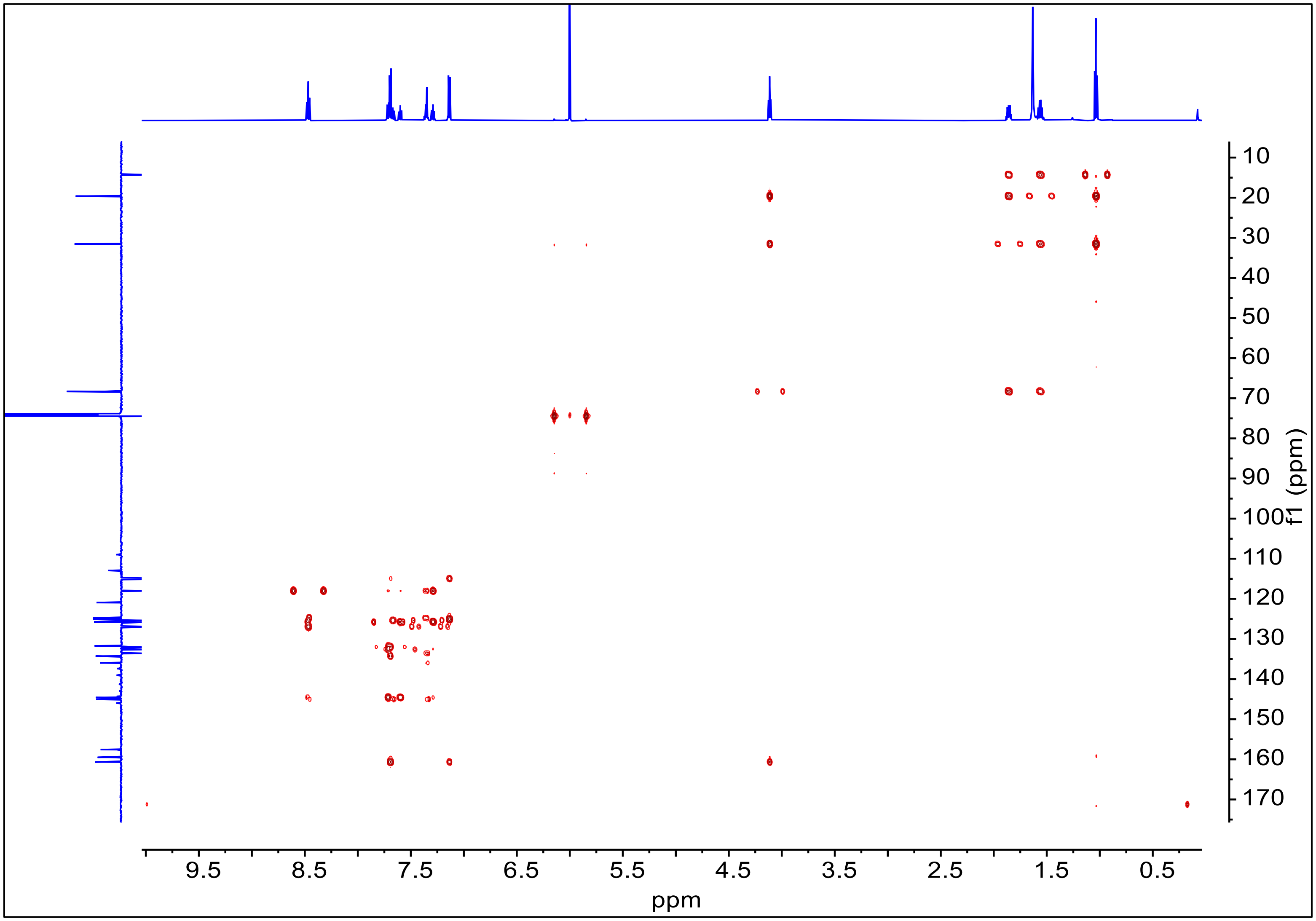

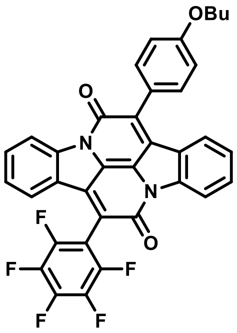

^{1}H NMR (400 MHz, CD_2Cl_2) – Derivative 7

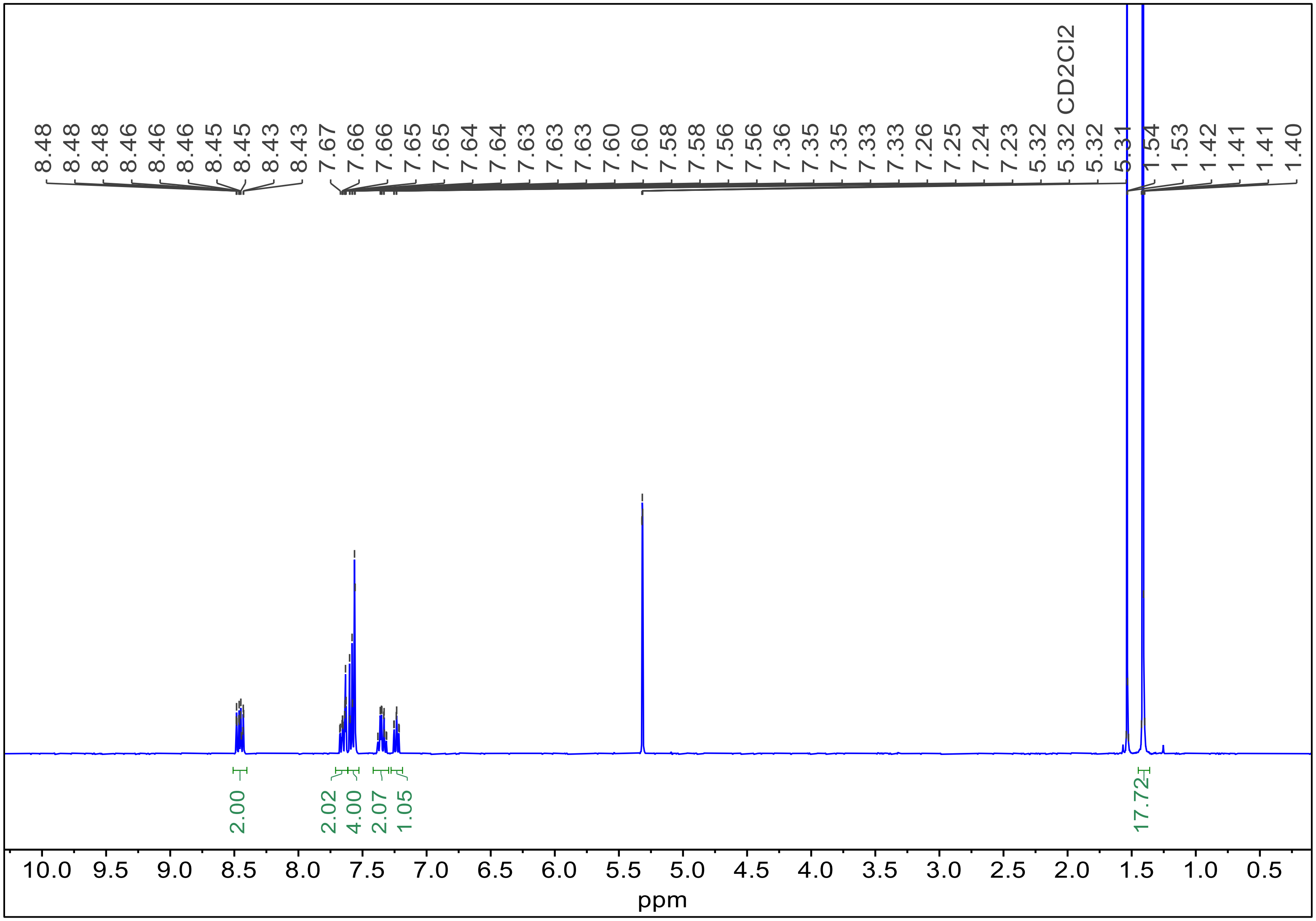

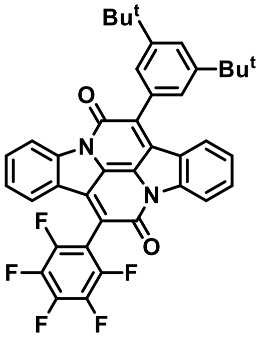

$^{13}C\{^1H\}$ APT NMR (125 MHz, CD_2Cl_2) – Derivative 7

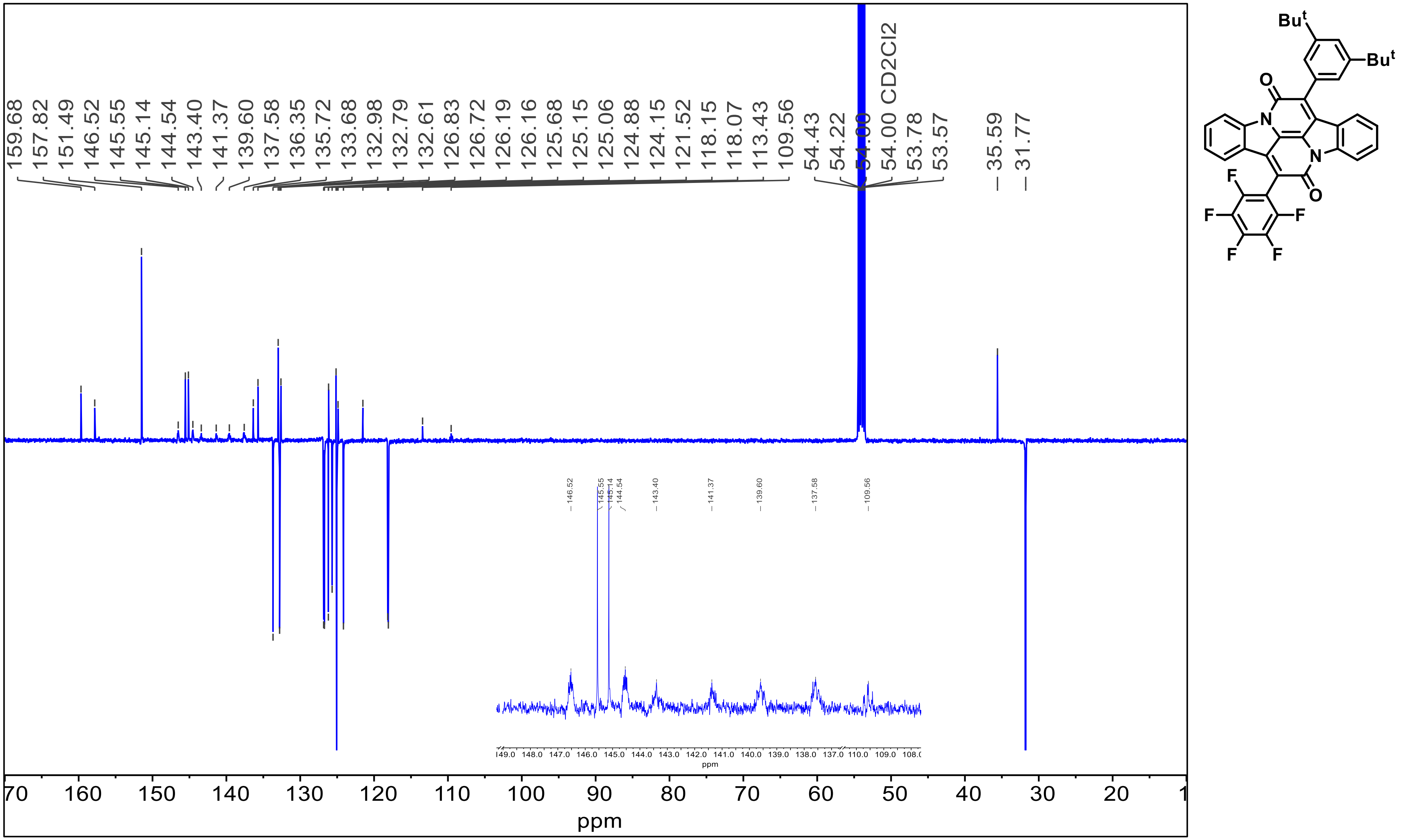

^{19}F NMR (376 MHz, CD_2Cl_2) – Derivative 7

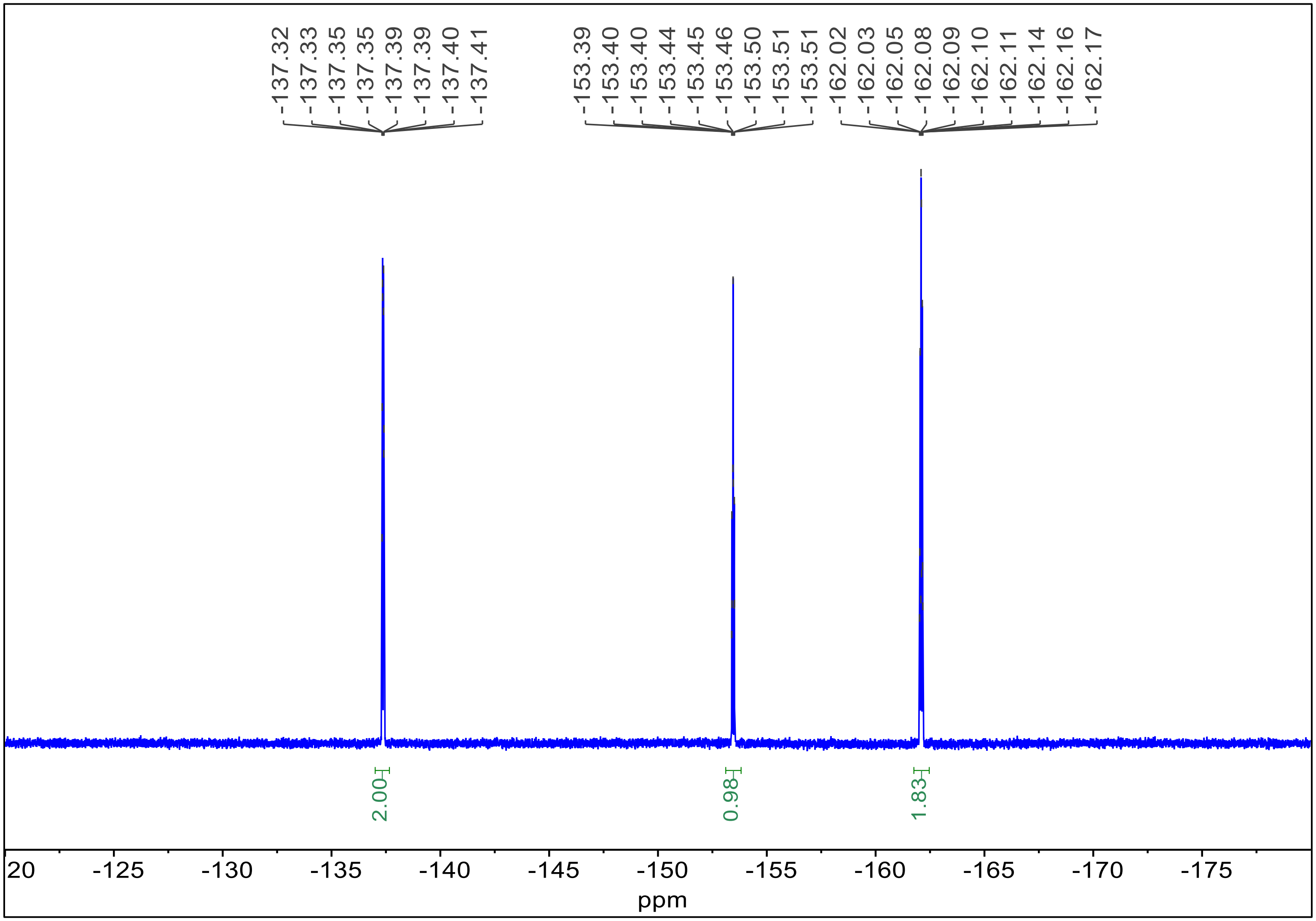

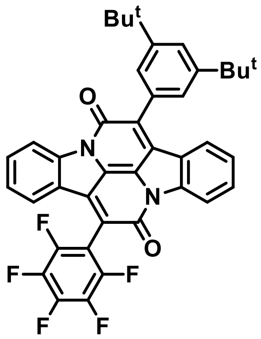

^{1}H – ^{1}H COSY (CD_2Cl_2) – Derivative 7

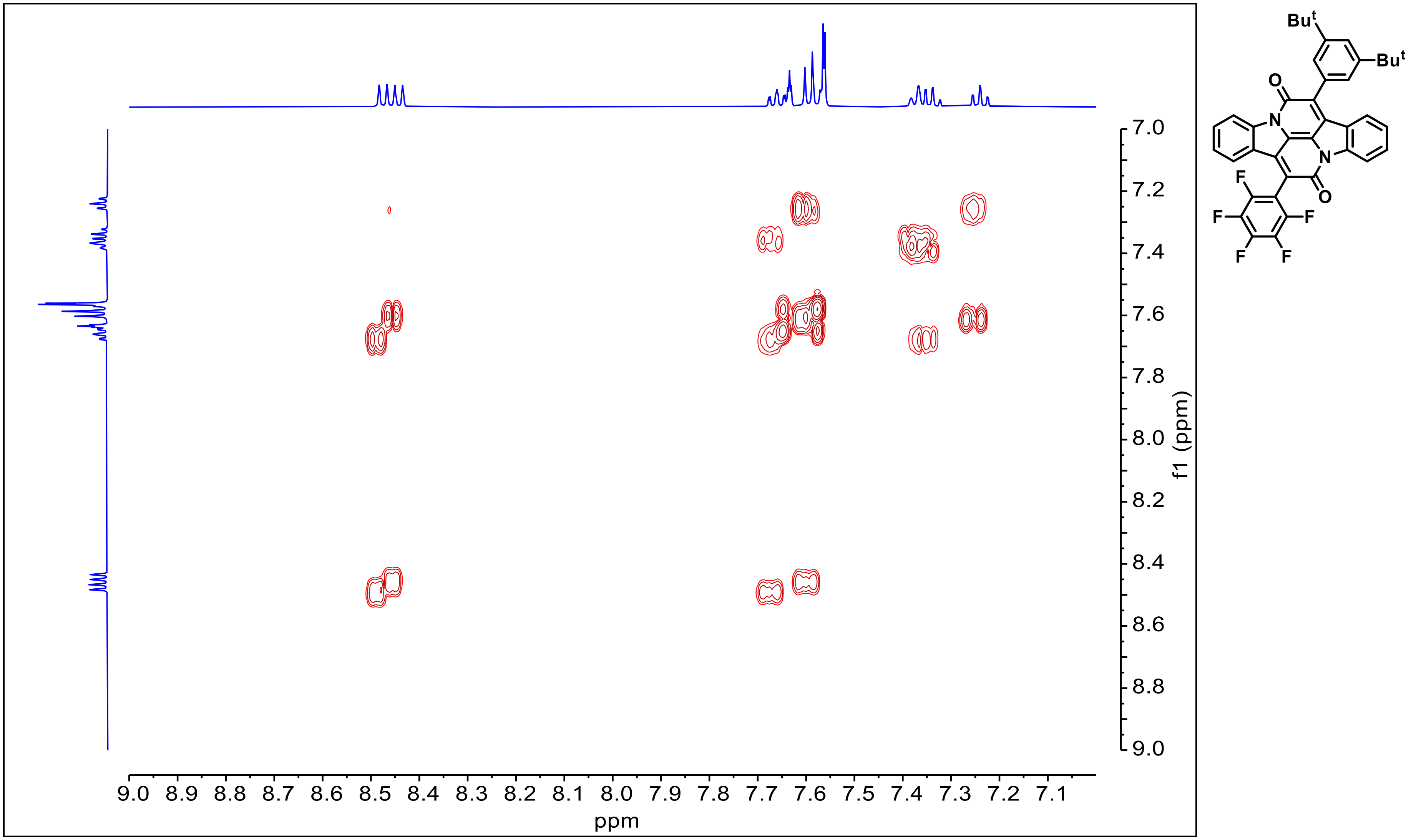

HSQC (CD_2Cl_2) – Derivative 7

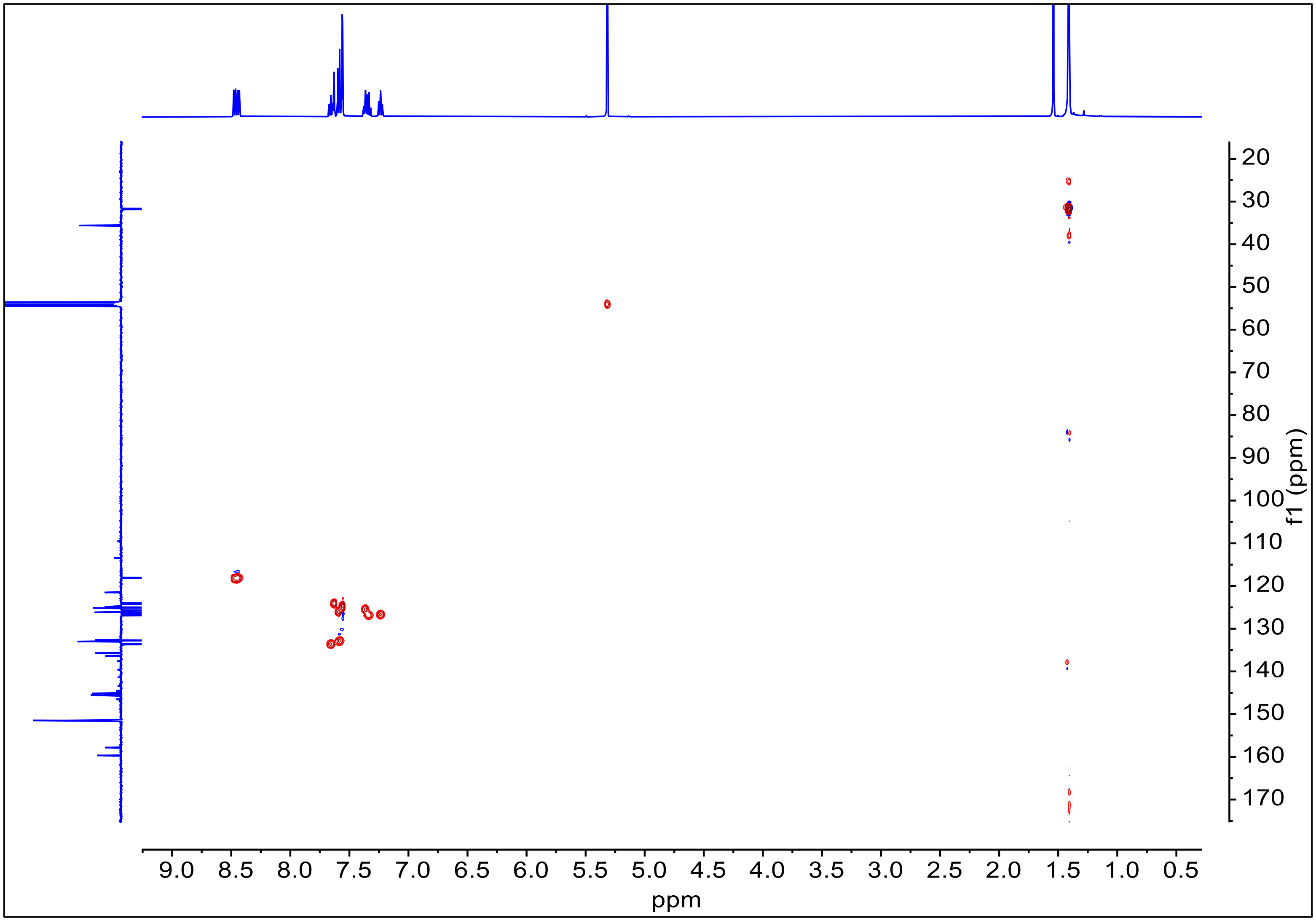

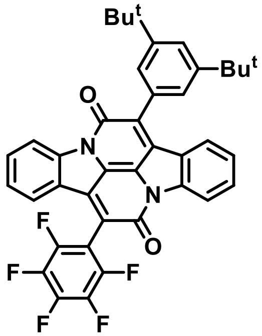

HMBC (CD_2Cl_2) – Derivative 7

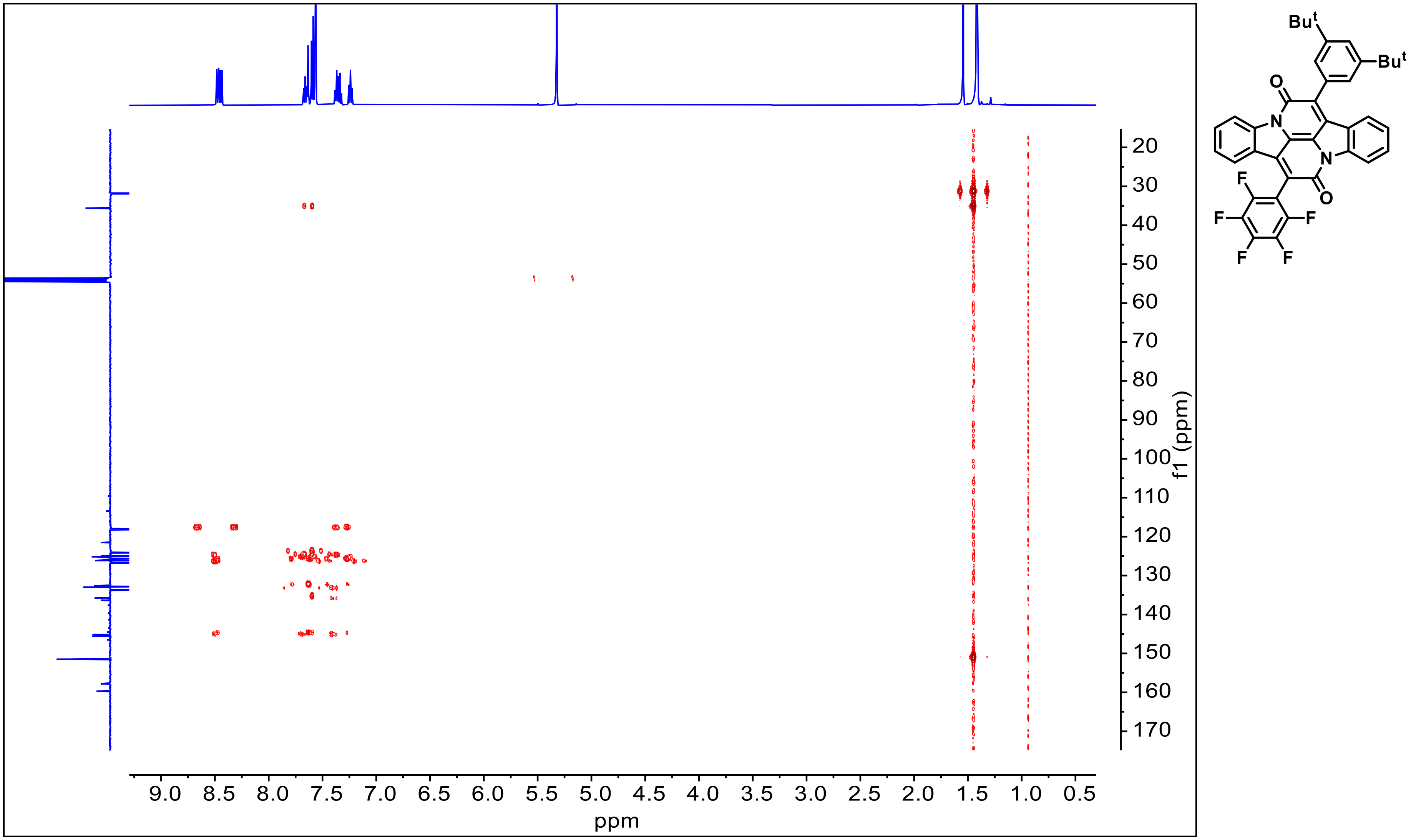

^{1}H NMR (400 MHz, $Cl_2CDCDCl_2$) – Derivative 1-d_2

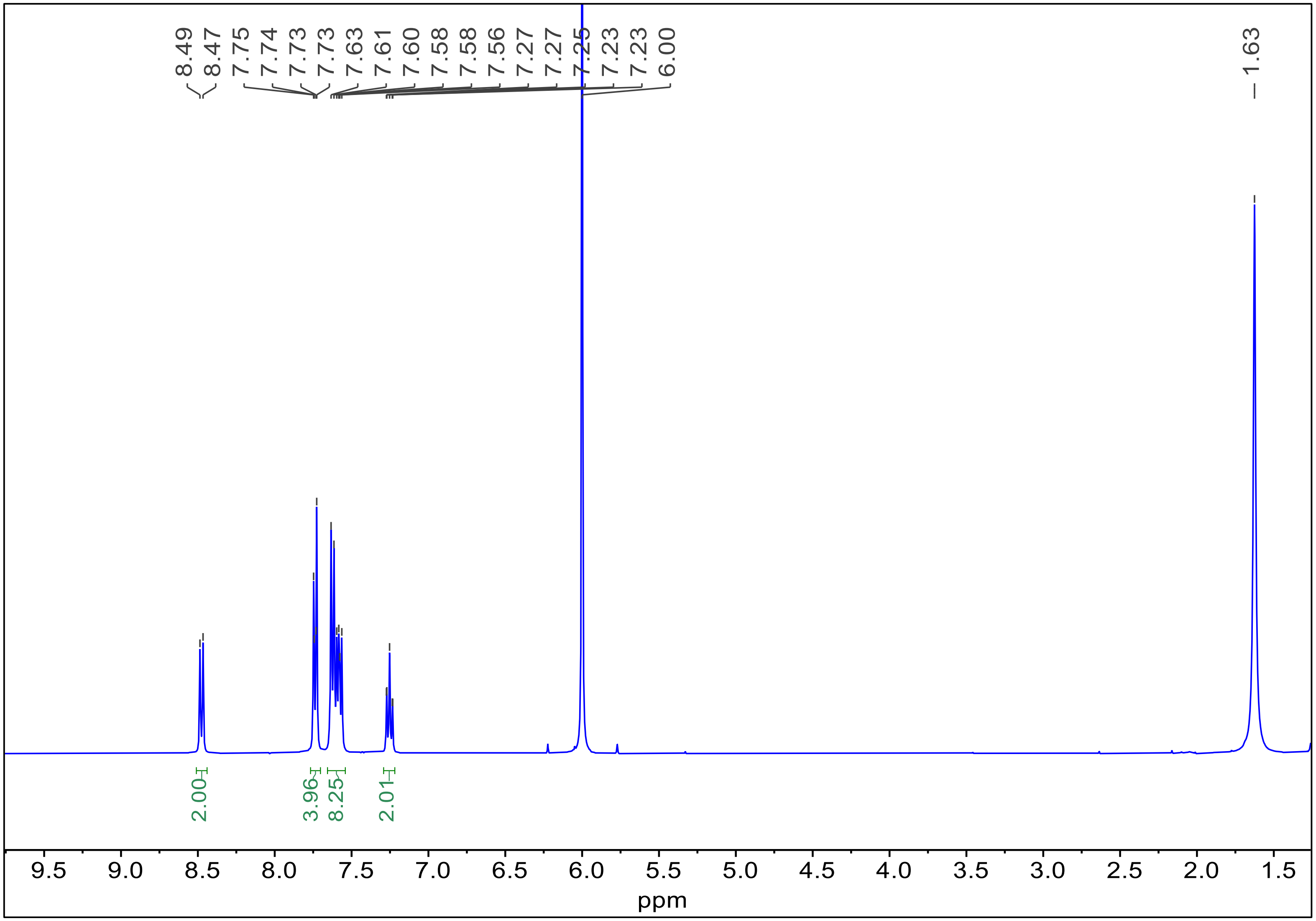

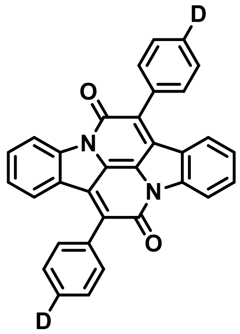

^{2}H NMR (77 MHz, $Cl_2CHCHCl_2$) – Derivative 1-d_2

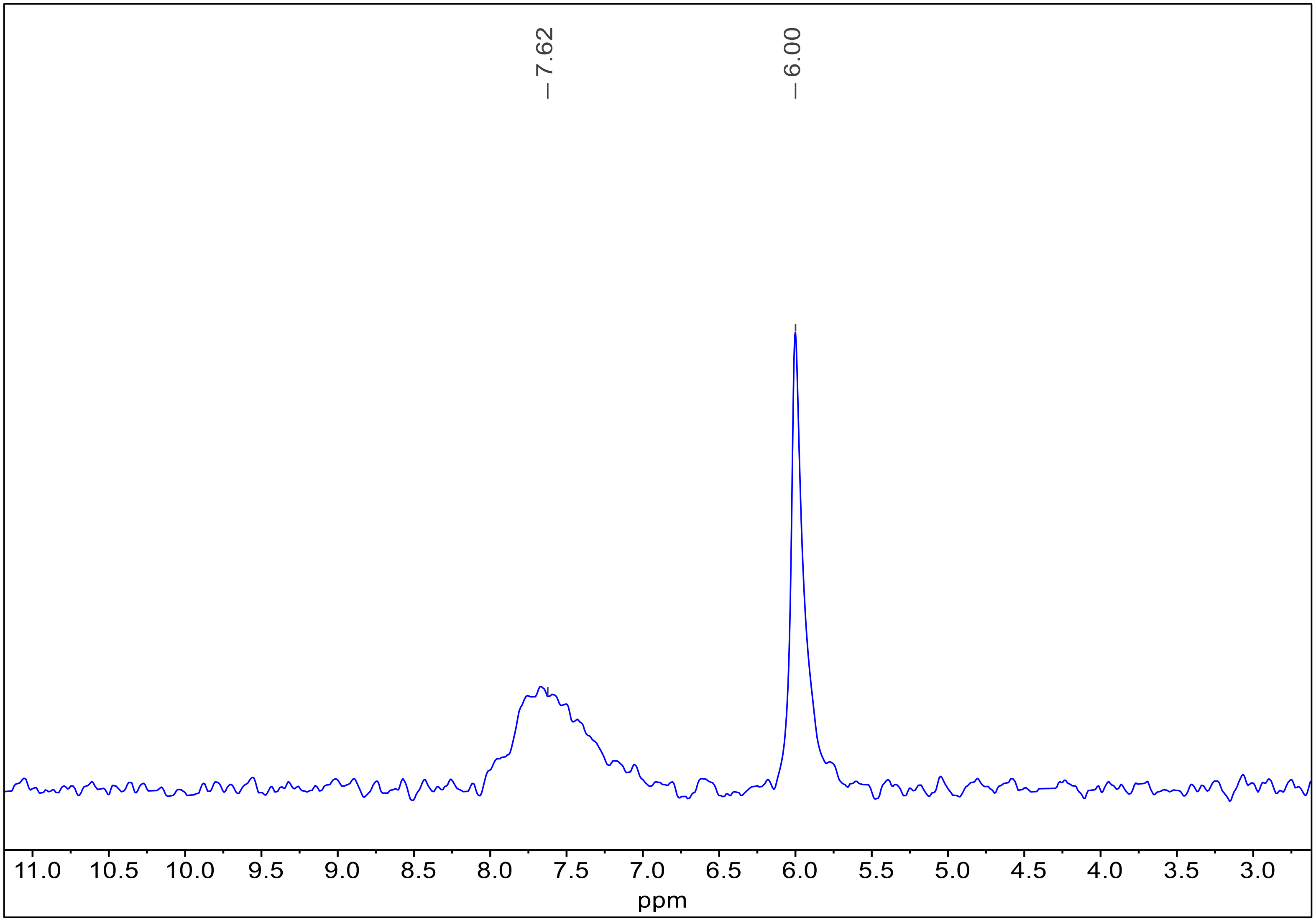

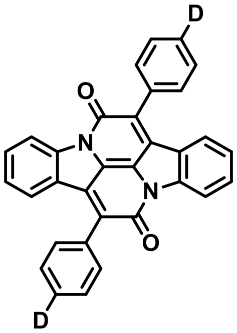

$^{13}C\{^1H\}$ NMR APT (125 MHz, $Cl_2CDCDCl_2$) – Derivative 1-d_2

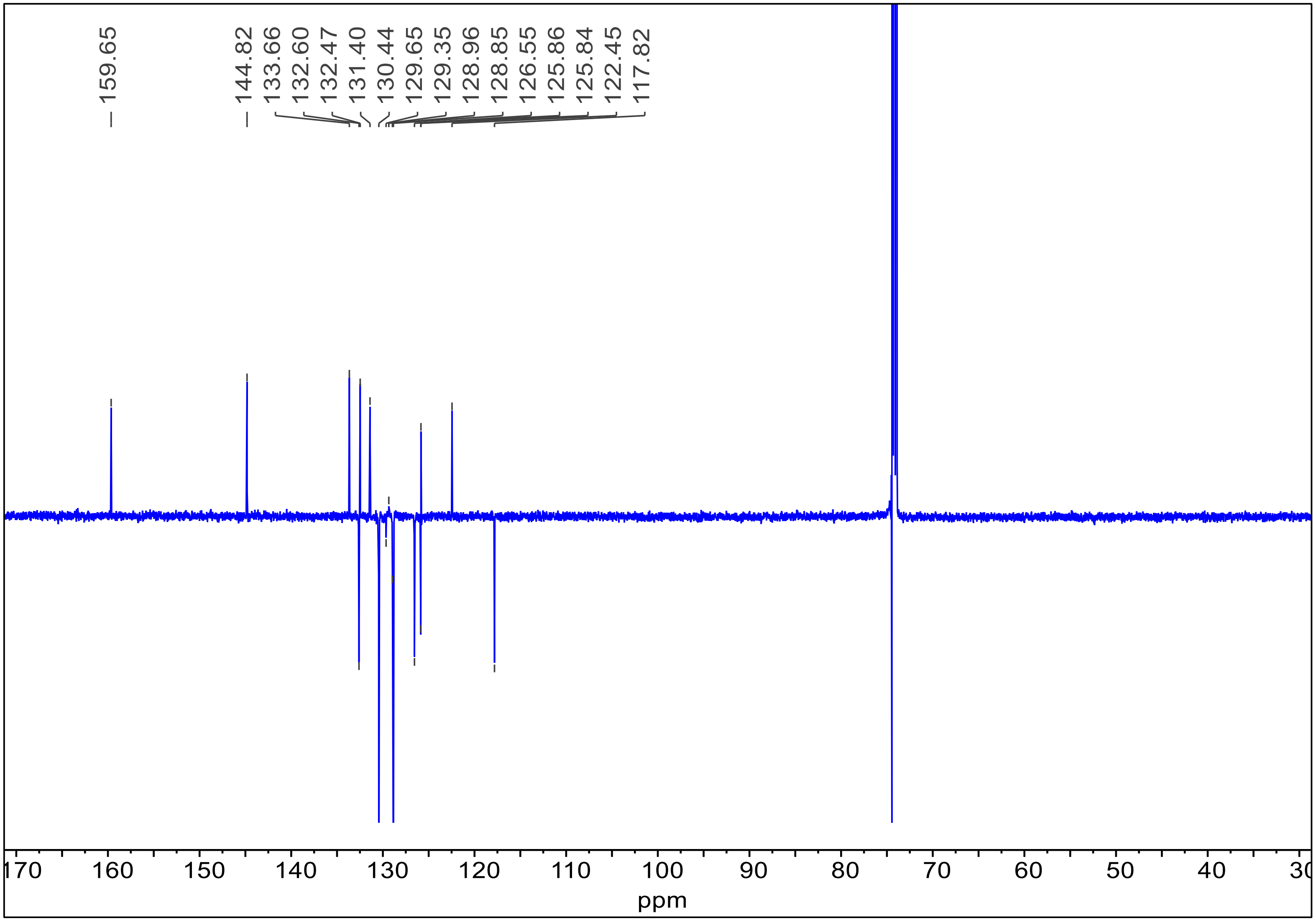

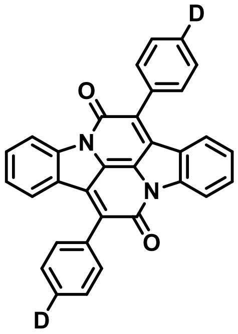

$^{1}H – ^{1}H$ COSY ($Cl_2CDCDCl_2$) – Derivative 1-d_2

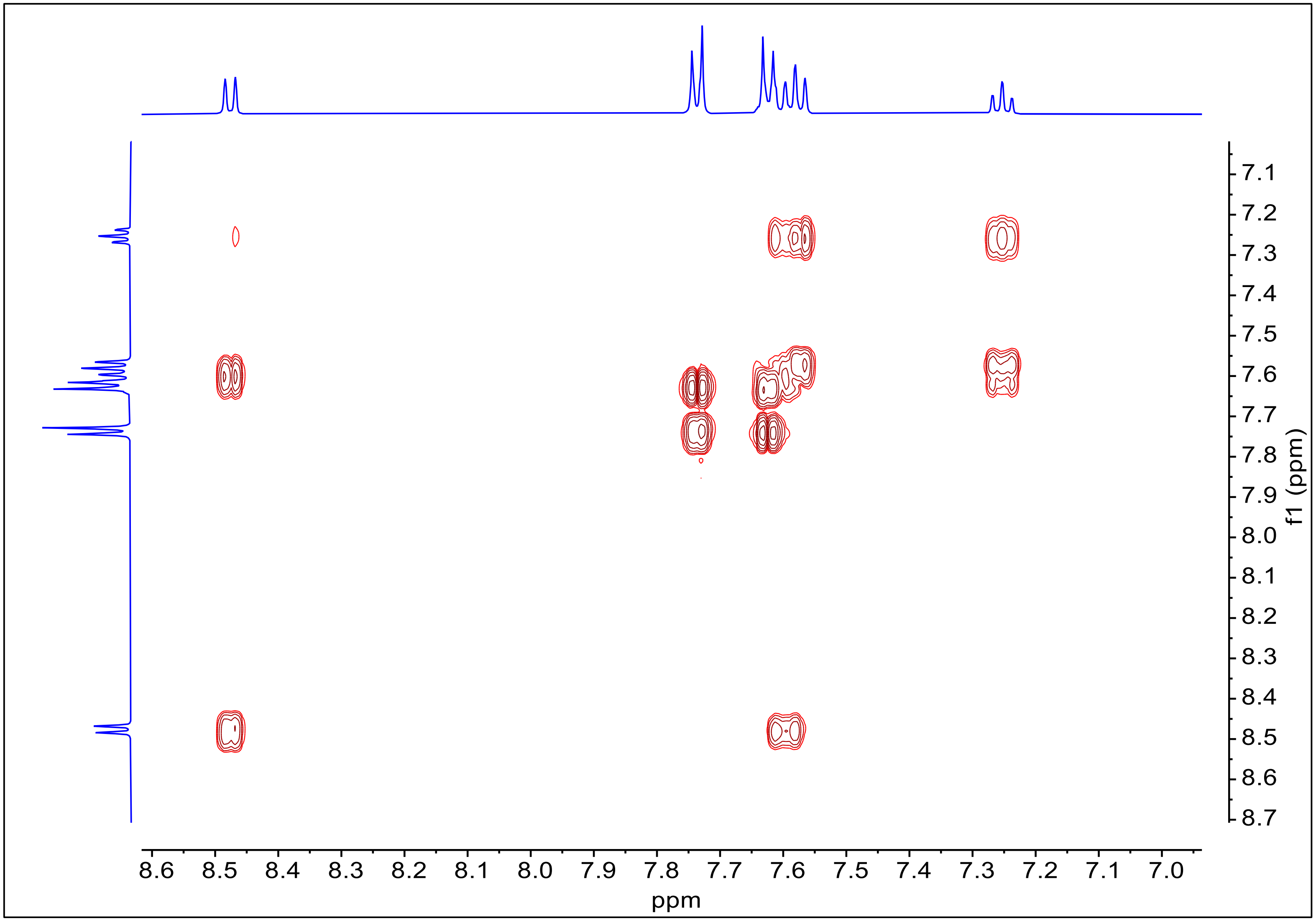

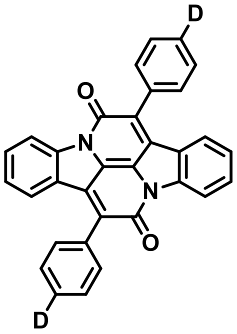

HSQC ($Cl_2CDCDCl_2$) – Derivative 1-d_2

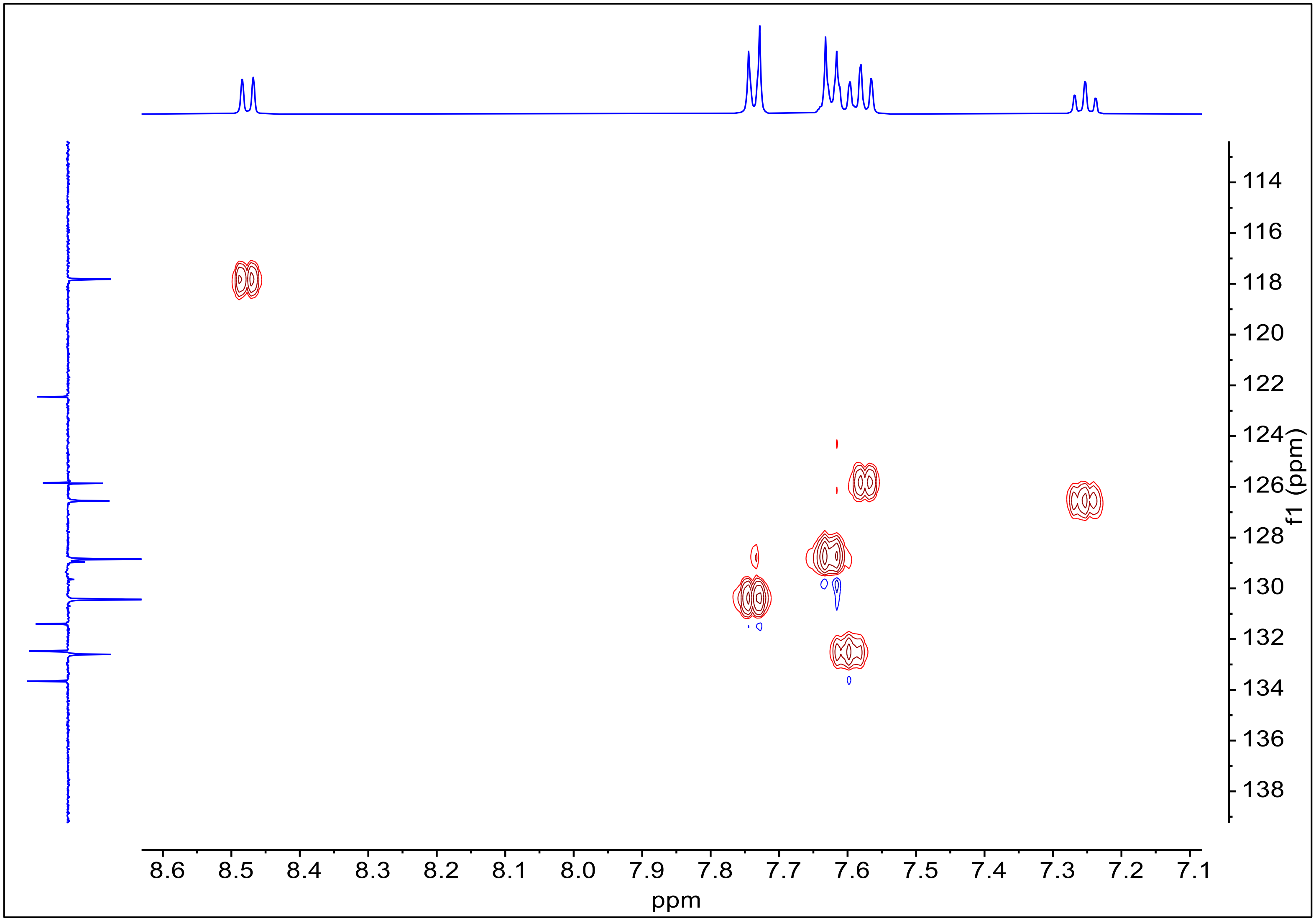

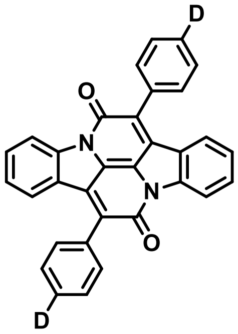

HMBC ($Cl_2CDCDCl_2$) – Derivative 1-d_2

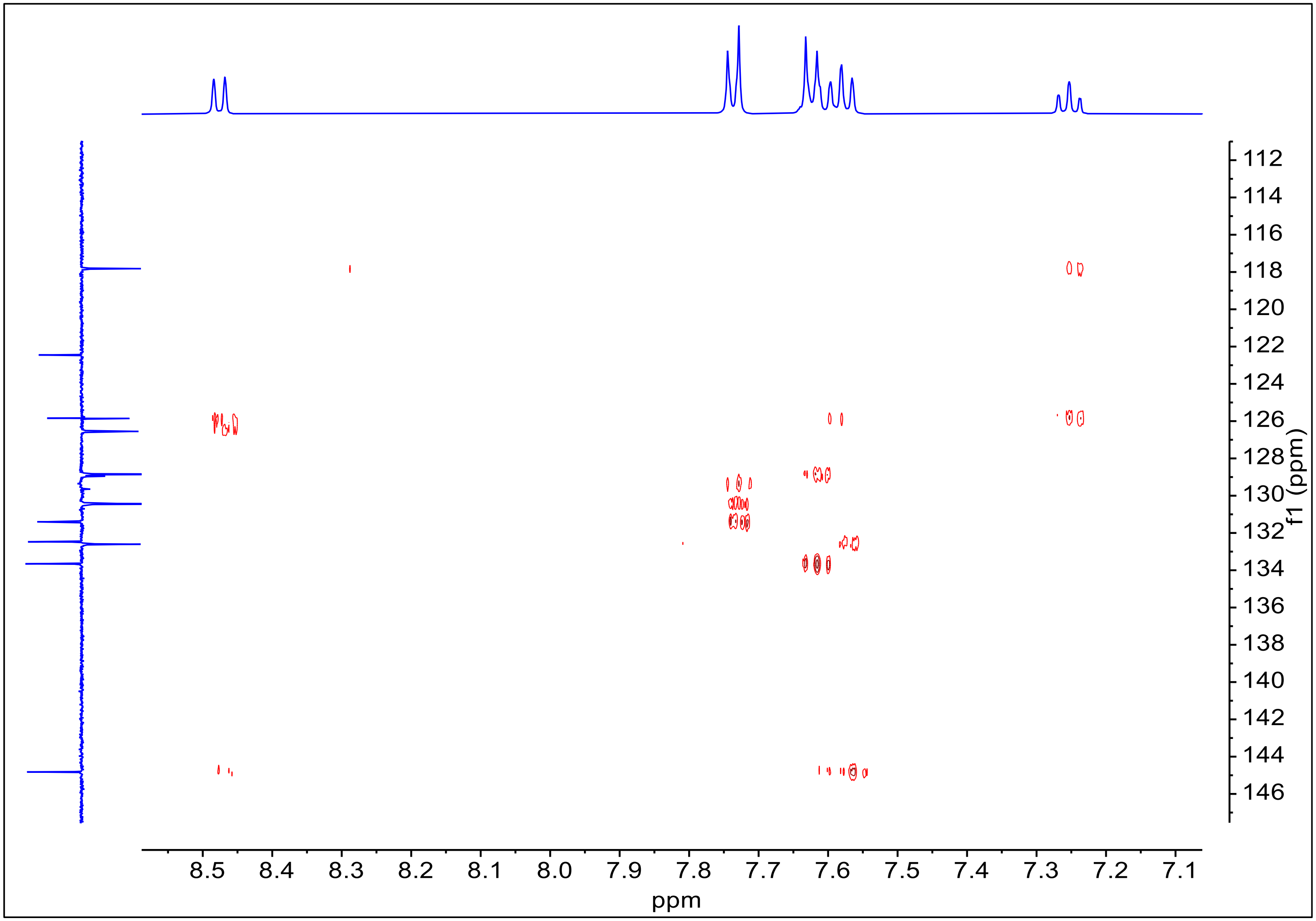

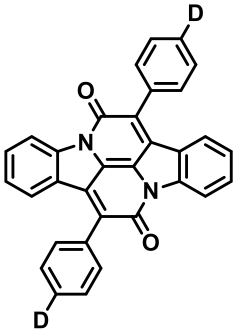

^{1}H NMR (400 MHz, $Cl_2CDCDCl_2$) – Derivative 1-d_4

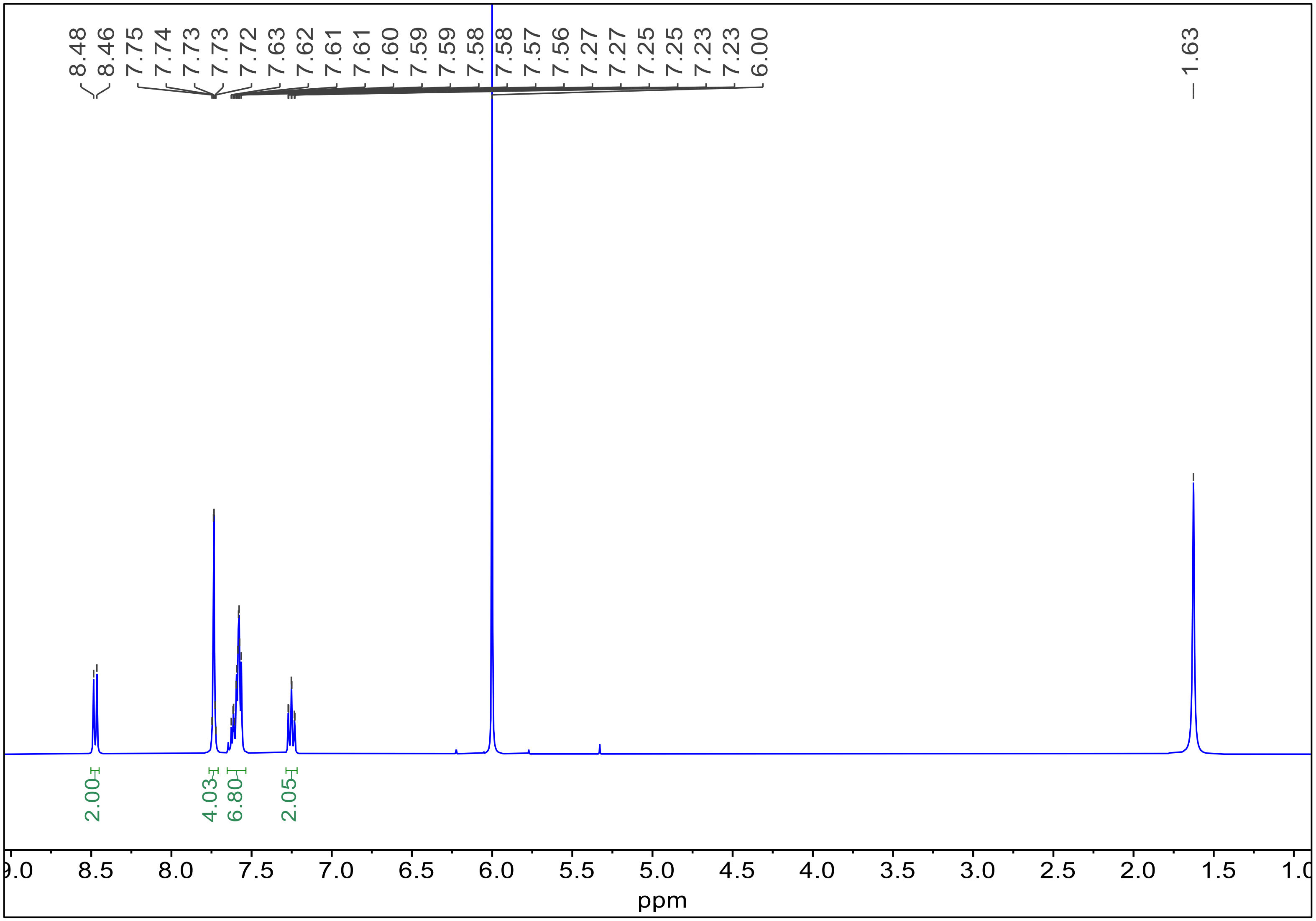

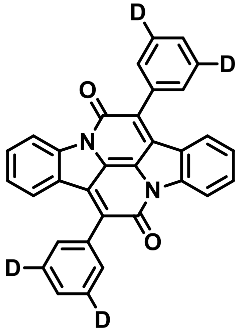

^{2}H NMR (77 MHz, $Cl_2CHCHCl_2$) – Derivative 1-*d*$_4$

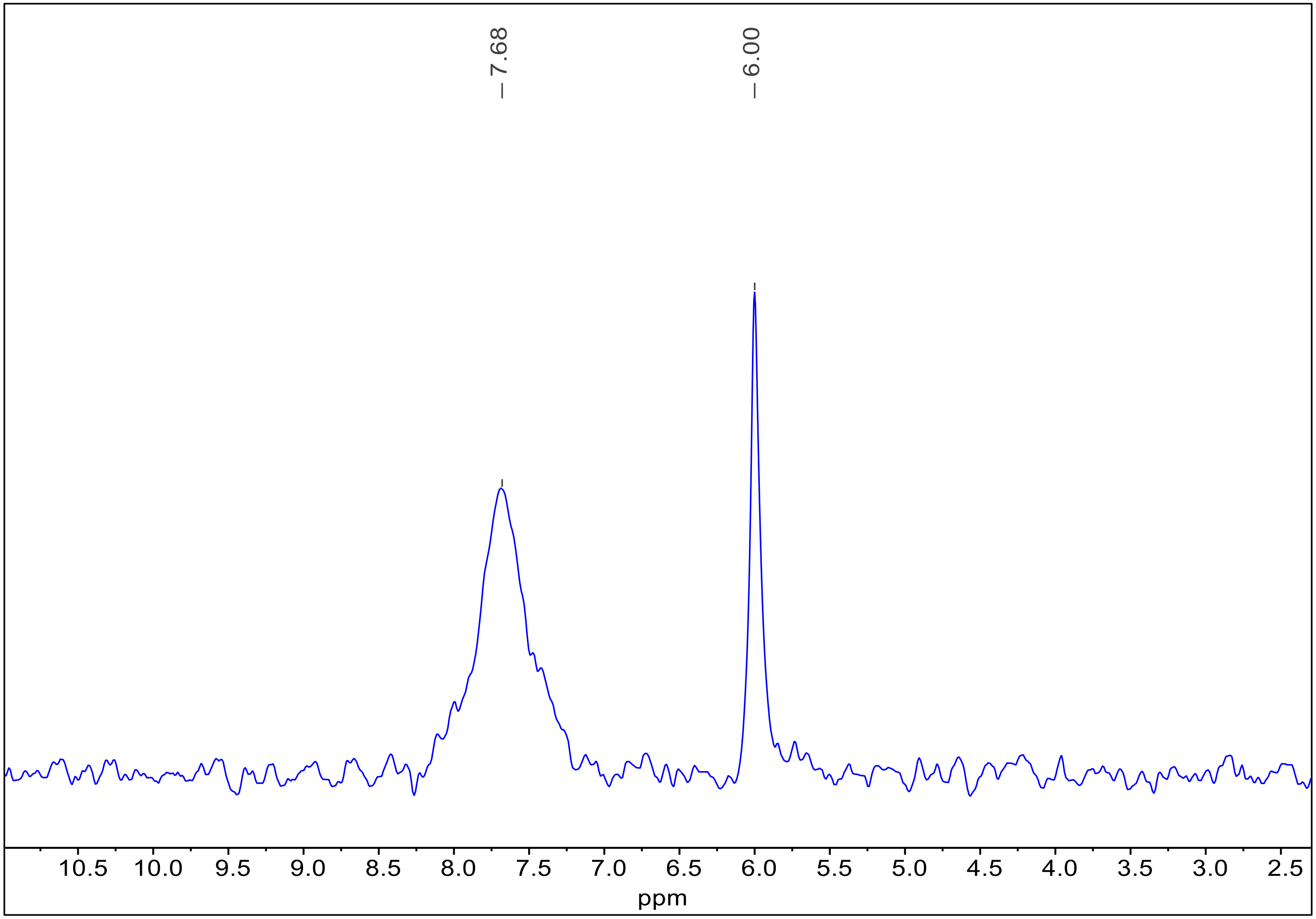

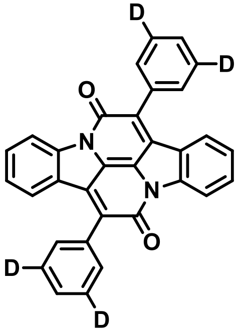

$^{13}C\{^{1}H\}$ NMR APT (125 MHz, $Cl_2CDCDCl_2$) – Derivative 1-*d*$_4$

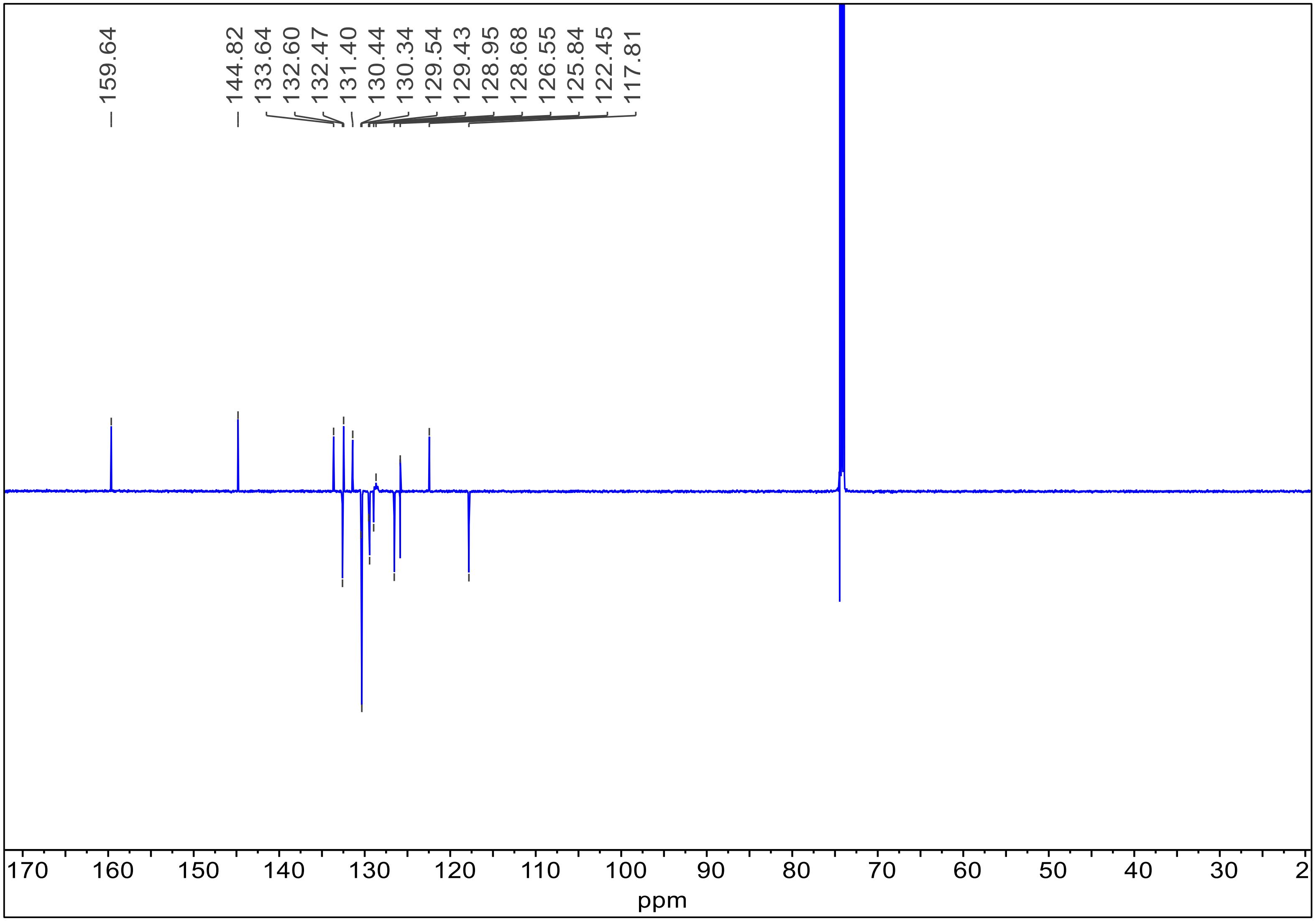

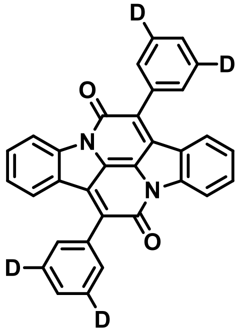

HSQC ($Cl_2CDCDCl_2$) – Derivative 1-*d*$_4$

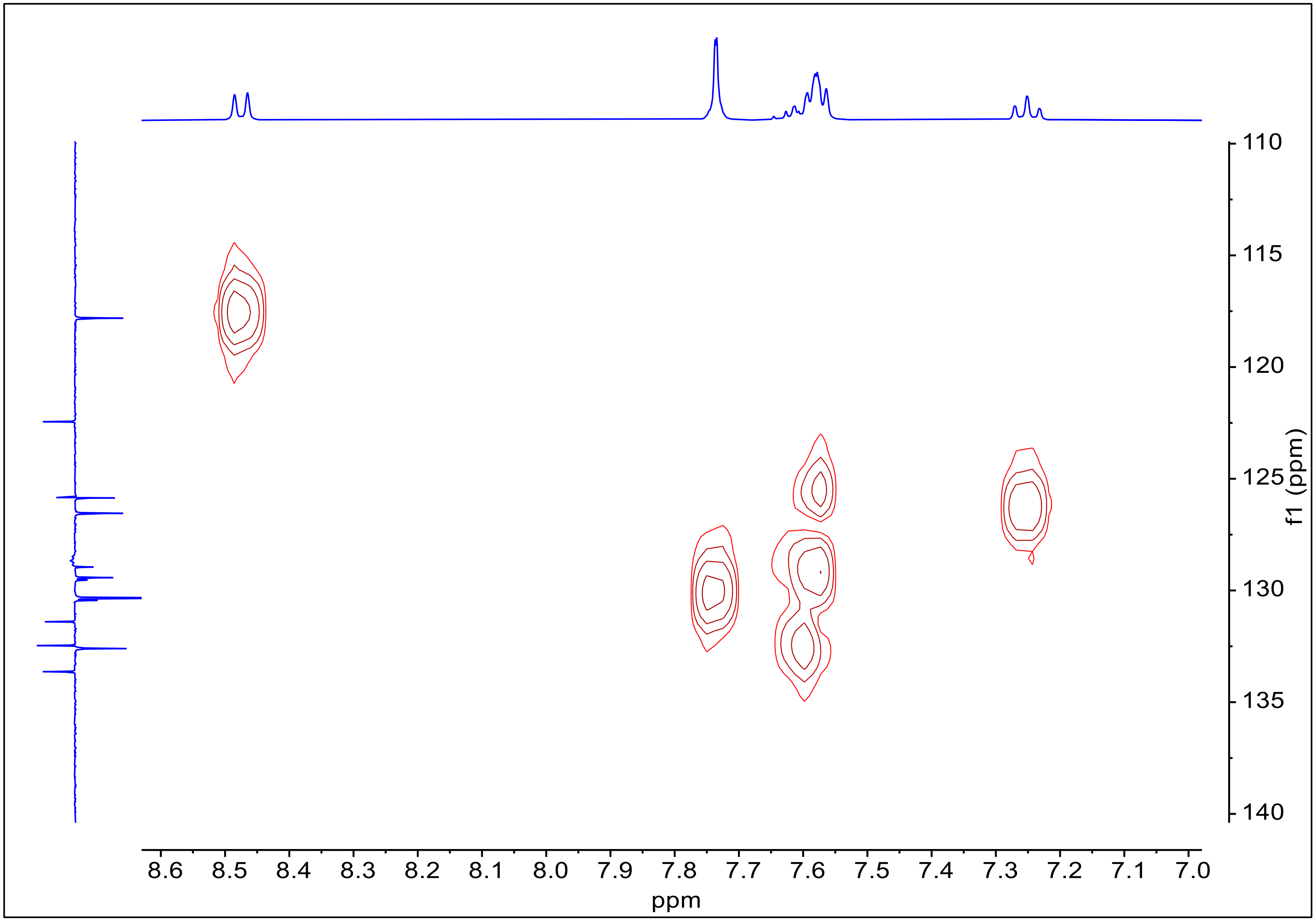

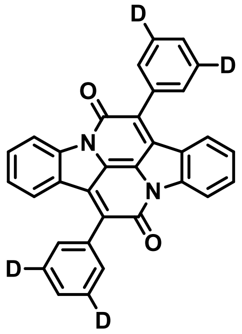

HMBC ($Cl_2CDCDCl_2$) – Derivative 1-*d*$_4$

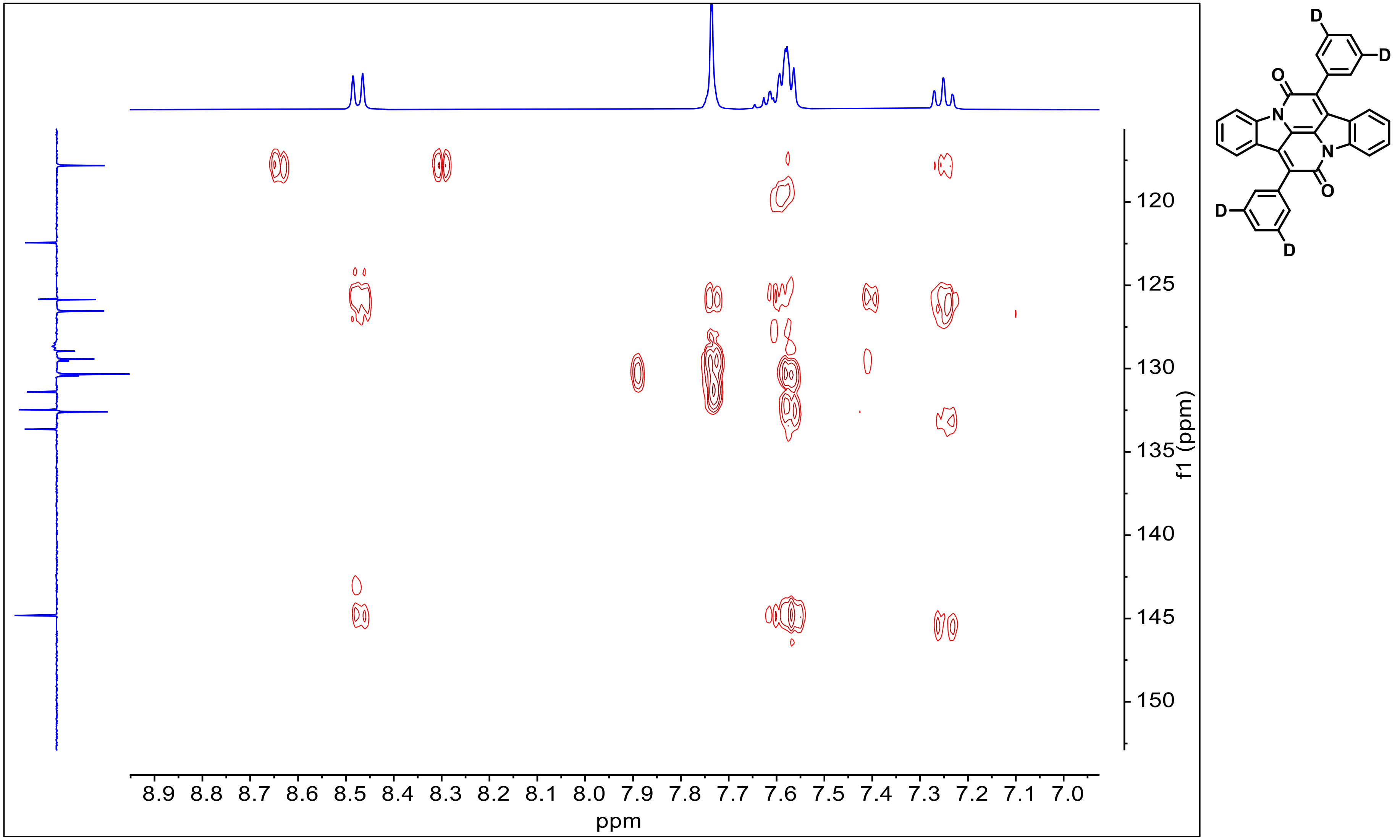

^{1}H NMR (400 MHz, $Cl_2CDCDCl_2$) – Derivative 1-d_{10}

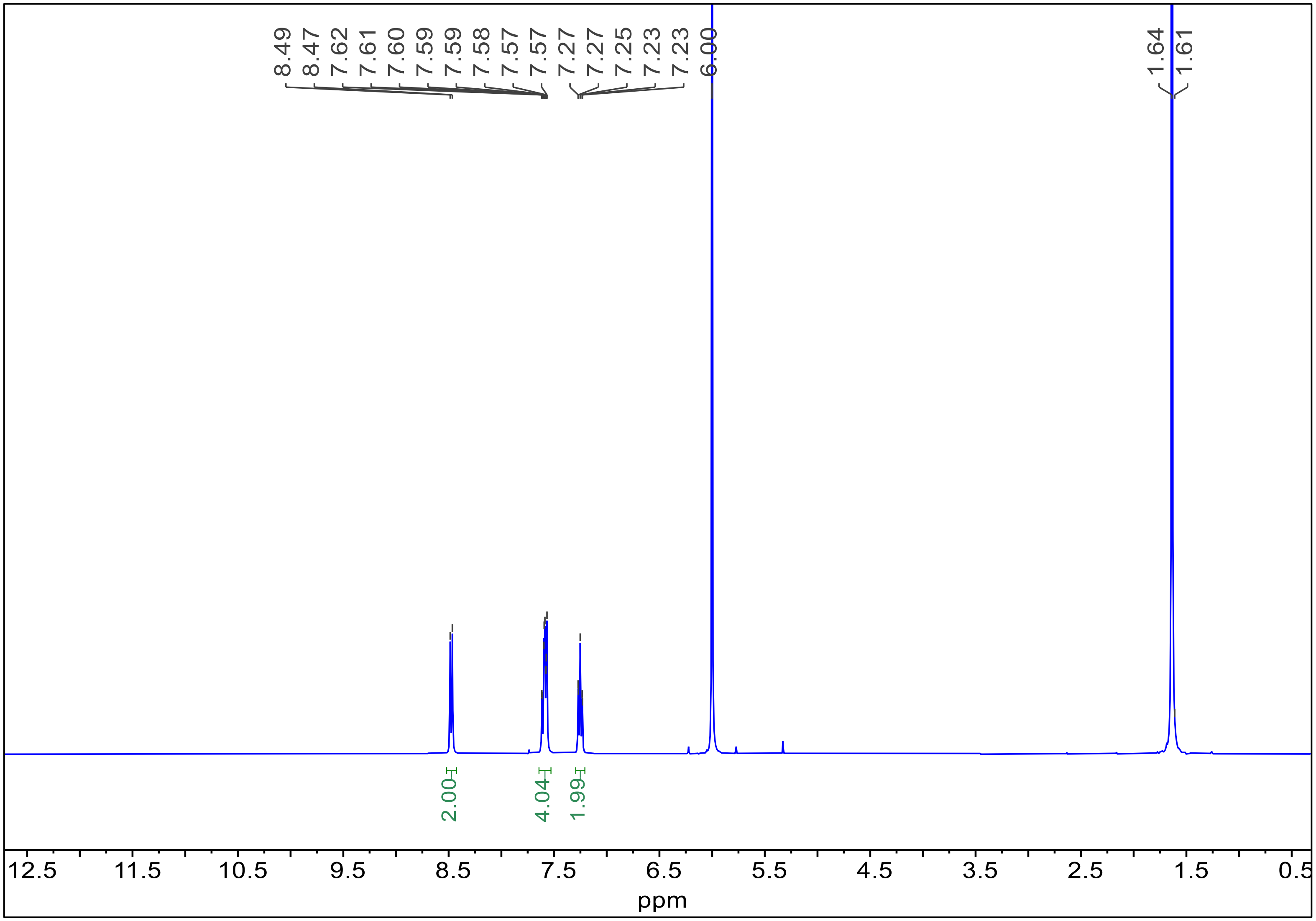

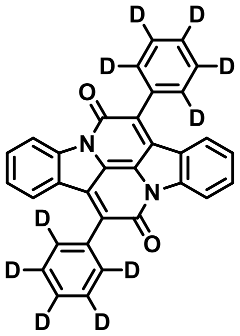

^{2}H NMR (77 MHz, $Cl_2CHCHCl_2$) – Derivative 1-d_{10}

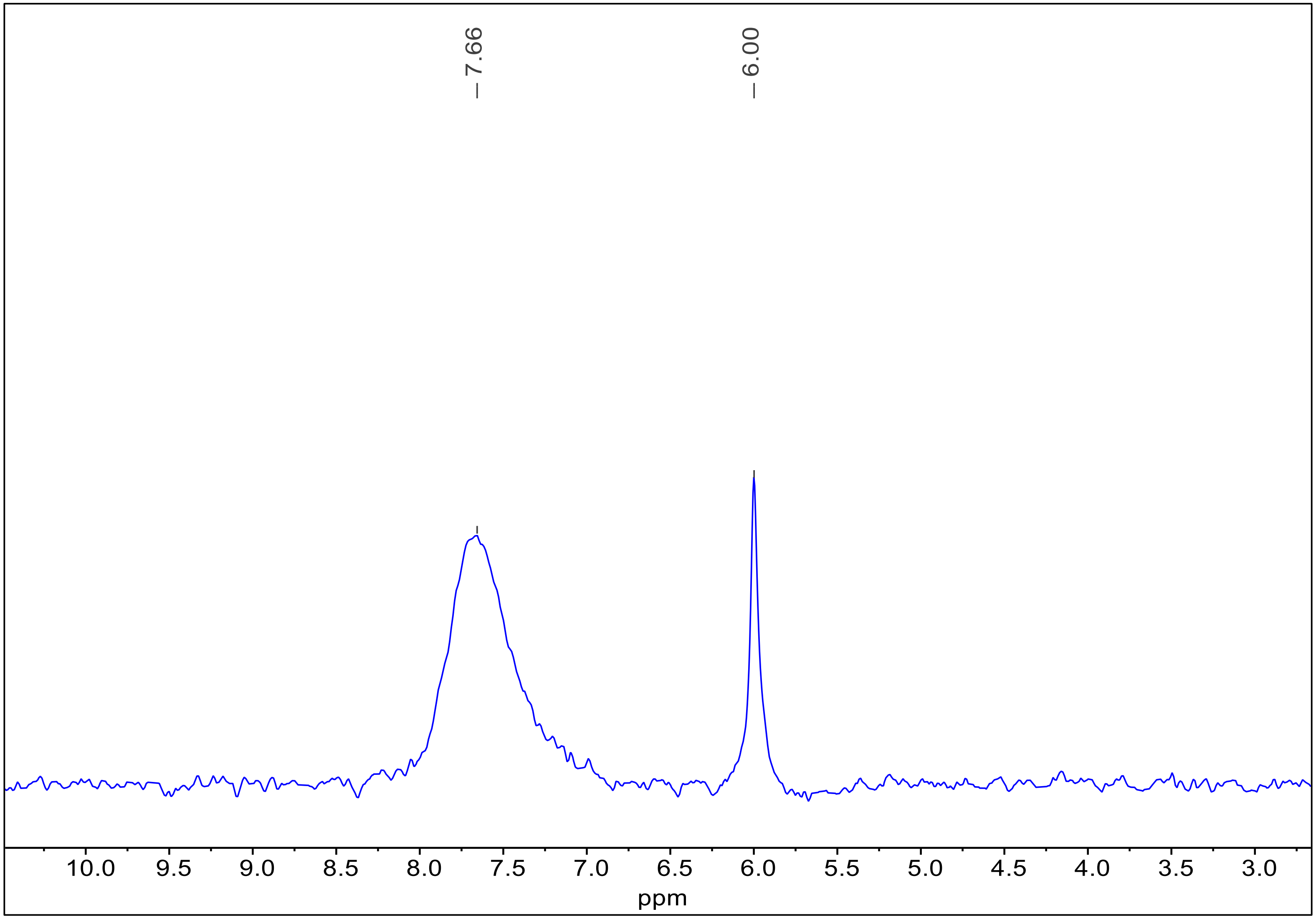

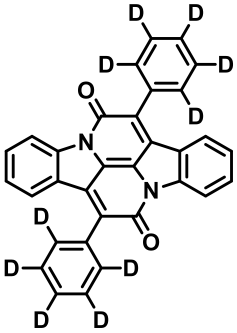

$^{13}C\{^{1}H\}$ NMR APT (125 MHz, $Cl_2CDCDCl_2$) – Derivative 1-d_{10}

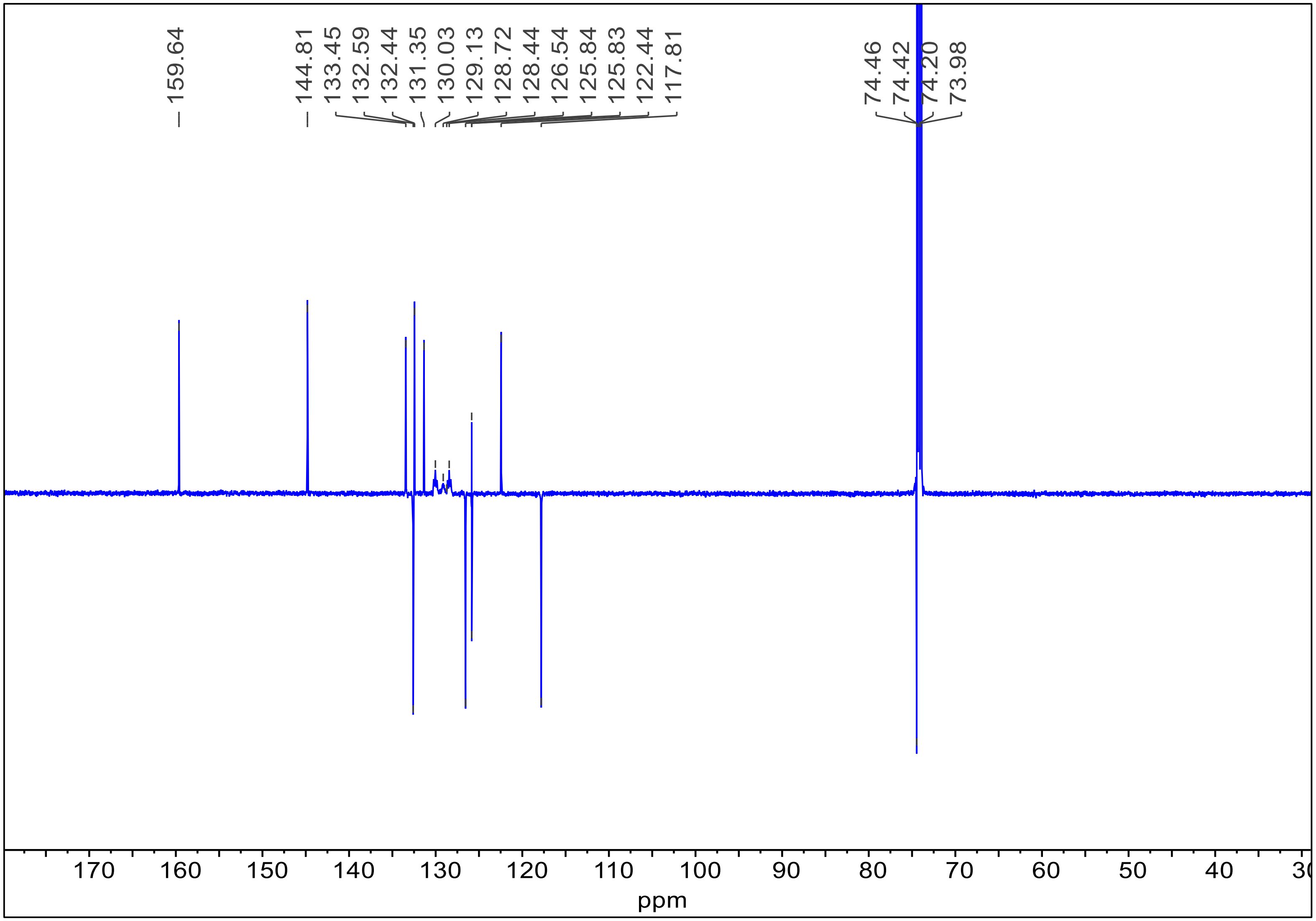

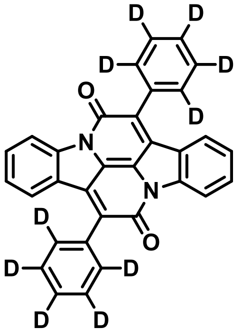

HSQC ($Cl_2CDCDCl_2$) – Derivative 1-*d*$_{10}$

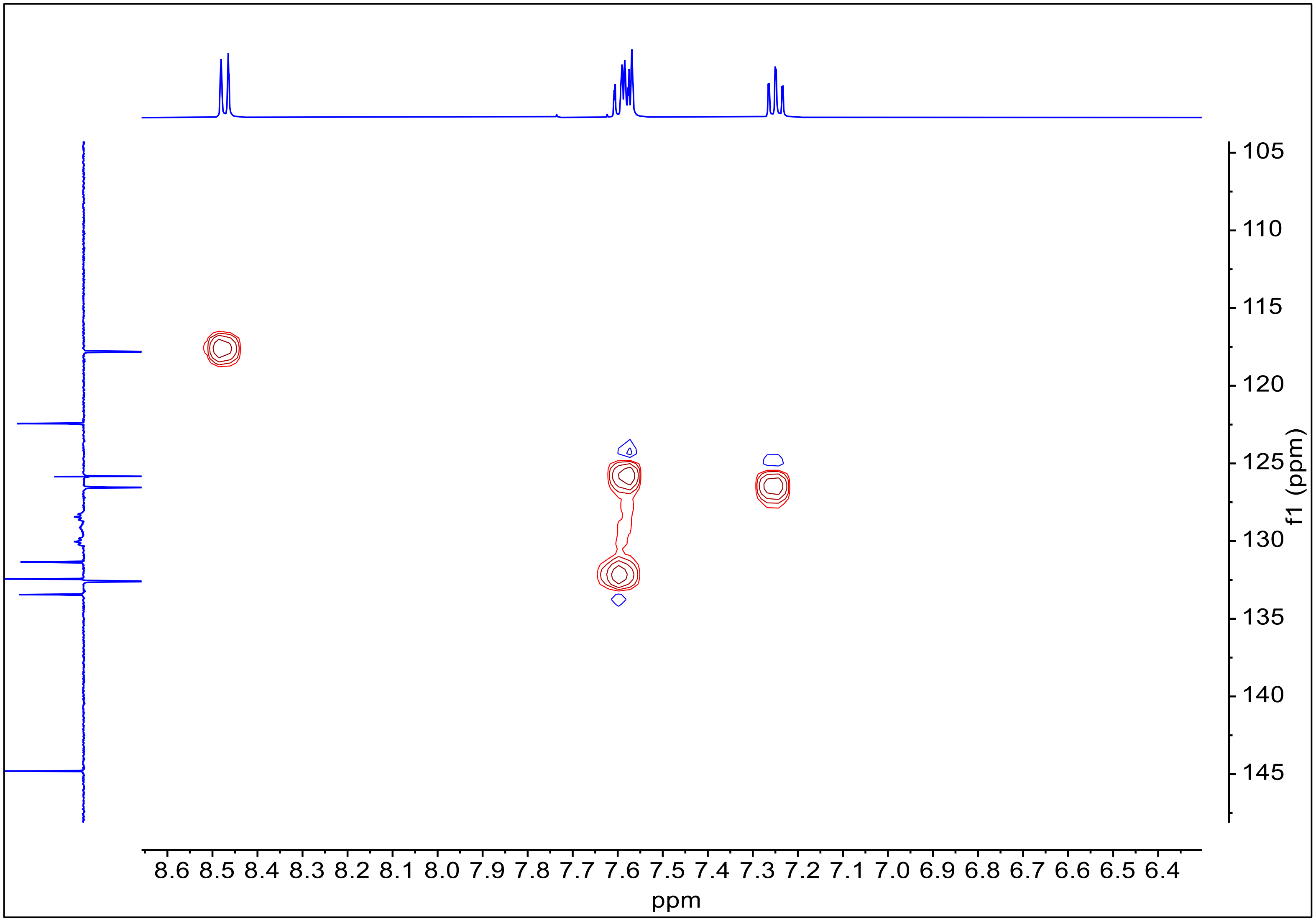

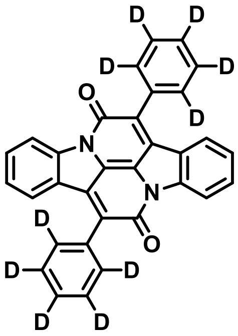

HMBC ($Cl_2CDCDCl_2$) – Derivative 1-*d*$_{10}$

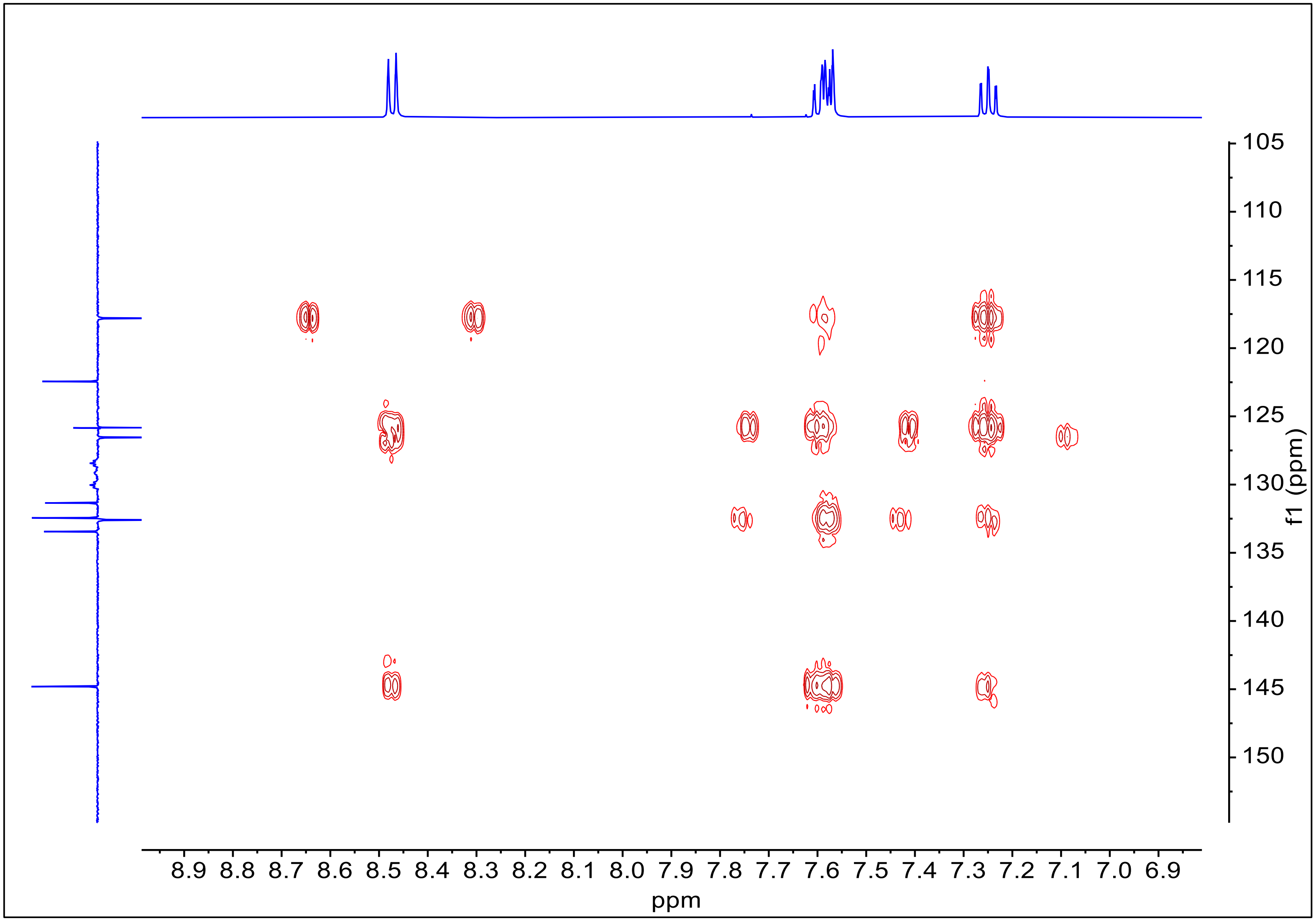

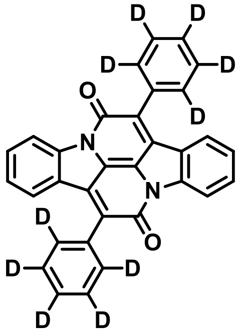

Nanosecond transient absorption spectroscopy (TAS) measurements

Table S1. Observed (obsd., in dichloromethane) and Calculated (con, dis) Properties of Singlet States of **1** - **7** (energies in $10^3 cm^{-1}$).

trans.	**1**			**2**			**3**			**4**			**5**			**6**			**7**		
$S_0 \rightarrow$	con	dis	obsd.	con	dis	obsd.	con	dis	obsd.	con	dis	obsd.	con	dis	obsd.	con	dis	obsd.	con	dis	obsd.
S_1 (f)^a	19.6 (0.57)	19.7 (0.56)	18.6 (0.22)	18.6 (0.77)	18.7 (0.75)	18.1 (0.28)	19.3 (0.68)	19.3 (0.67)	18.4 (0.27)	19.7 (0.52)	19.7 (0.52)	18.6 (0.23)	18.9 (0.71)	19.0 (0.71)	18.2 (0.30)	18.9 (0.64)	18.9 (0.64)	18.2 (0.28)	19.4 (0.61)	19.4 (0.61)	18.5 (0.26)
S_2 (f)^a	24.0 (0)	24.0 (0)		22.6 (0)	22.5 (0)		23.8 (0)	23.8 (0)		23.2 (0)	23.2 (0)		23.1 (0.01)	23.1 (0.01)		22.7 (0.02)	22.7 (0.02)	26.2 (0.03)	23.4 (0)	23.4 (0)	
S_3 (f)^a	27.0 (0.16)	27.0 (0.16)	27.5 (0.07)	24.7 (0.06)	24.6 (0.06)	26.2 (0.05)	25.7 (0.01)	25.7 (0.01)		27.0 (0.19)	27.0 (0.20)	27.6 (0.07)	25.2 (0.05)	25.2 (0.04)	26.6 (0.07)	24.7 (0.03)	24.7 (0.04)	27.6 (0.04)	25.1 (0)	25.1 (0)	
S_4 (f)^a	28.3 (0)	28.3 (0)		26.0 (0)	26.0 (0)	29.5 (0.01)	26.1 (0.01)	26.1 (0.02)	27.2 (0.08)	27.8 (0)	27.8 (0)		25.9 (0)	25.9 (0)		28.3 (0.04)	28.3 (0.05)	30.3 (0.02)	25.8 (0.09)	25.8 (0.09)	27.8 (0.08)
$S_1 \rightarrow S_0$			17.7			17.3			17.7			18.1			17.4			17.4			17.8
ave ^{b}			18.1			17.6			18.0			18.4			17.8			17.8			18.1
Φ_F ^{c}			0.46			0.42			0.59			0.32			0.57			0.54			0.46
τ_F/ns ^{d}			5.0			5.0			5.2			3.1			5.1			5.2			4.3

[a] Oscillator strength., $f = (4.3910^{-9}/n)\int_{band} e(\tilde{v})\, d\tilde{v}$; n = 1.49693. [b] Obsd. crossing of normalized absorption and emission spectra. [c] Fluorescence quantum yield. Standard: Rhodamine 6G in ethanol; corrected for different index of refraction. Error, ± 0.05. [d] Fluorescene lifetime. Error, ± 0.1 ns.

*Estimation of Triplets' Energies of **1-7***

To estimate triplet state energies of **1-7**, triplet sensitization experiments were performed with several sensitizers of different triplet state energy. The rate constant of triplet energy transfer (k_q) from sensitizer to **1-7** was measured in a freeze−pump−thaw degassed toluene solution. The following sensitizers and their known triplet energies were used: anthracene (42.5 kcal/mol, 14 900 cm^{-1}),[1] anthanthrene (33.8 kcal/mol, 11 800 cm^{-1}),[2] tetraphenylporphyrin (TPP, 33 kcal/mol, 11 500 cm^{-1}),[3] chlorophyll b (31.1-32.5 kcal/mol, 10 900-11 400 cm^{-1}),[3] Pt(II) phthalocyanine (30.2 kcal/mol, 10 600 cm^{-1}),[4] and tetracene (29.3 kcal/mol, 10 200 cm^{-1}).[5] For Pt(II) phthalocyanine, the solvent used was chlorobenzene. The concentration of **1-7** was kept near 25 µM (only for **2**, 8 µM was used due to lower solubility), and the concentration of the sensitizer was adjusted to an absorbance of 0.3 over 2 mm at the excitation wavelength.

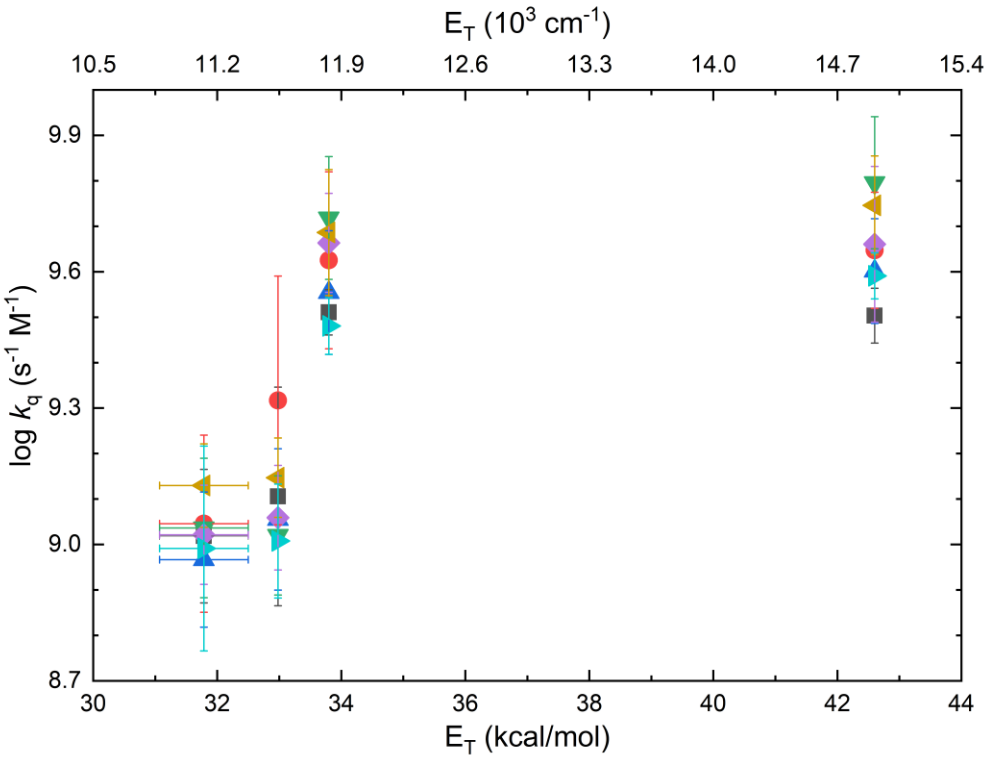

Figure S1. Plot of logarithms of the rate constant for energy transfer (k_q) to **1-7** (**1** black, **2** red, **3** blue, **4** green, **5** violet, **6** dark yellow, **7** turquoise) against triplet energies of the sensitizers (the mean of published values is used for chlorophyll b).

The measured rate constants of triplet state energy transfer are summarized in Table S2. Energy transfer remains diffusion controlled until the sensitizer has less than 3 kcal/mol excess energy over that required to excite the quencher from the ground to the triplet state.[2,6] In case of

1-**7** the transfer rate drops under the diffusion limit when the sensitizer triplet energy is reduced to less than 33.8 kcal/mol (11 800 cm^{-1}). Therefore, their triplet excitation energies are all within the 30.0 - 30.8 kcal/mol range. No transfer from tetracene and Pt(II) phthalocyanine to **1** was observed. A plot of the logarithm of the energy transfer rate constant against triplet state energy of the sensitizer is shown in Figure S1.

Table S2. Singlet State Energies of **1-7** and Measured Rate Constants for Triplet State Energy Transfer to **1**-**7** in Toluene at 21-22 °C. Energies are in kcal/mol (10^3 cm^{-1}).

compd.	E_{S1}	Sensitizer triplet energy					
		**42.5 (14.9)**[a]	**33.8 (11.8)**[b]	**33.0 (11.5)**[c]	**31.3-32.5 (10.9-11.4)**[d]	**30.2 (10.6)**[e]	**29.3 (10.2)**[f]
		k_q / 10^9 $s^{-1}M^{-1}$					
**1**	49.8 (17.4)	3.2 ± 0.2	3.2 ± 0.2	1.3 ± 0.3	1.0 ± 0.2	0	0
**2**	48.6 (17.0)	4.4 ± 0.6	4.2 ± 0.8	2.1 ± 0.6	1.1 ± 0.2	-	0
**3**	49.5 (17.3)	4.0 ± 0.5	3.6 ± 0.5	1.1 ± 0.2	0.92 ± 0.1	-	0
**4**	50.6 (17.4)	6.3 ± 0.9	5.2 ± 0.7	1.1 ± 0.1	1.1 ± 0.2	-	0
**5**	48.9 (17.1)	4.6 ± 0.8	4.6 ± 0.5	1.2 ± 0.1	1.1 ± 0.1	-	0
**6**	49.5 (17.3)	5.6 ± 0.6	4.9 ± 0.7	1.4 ± 0.1	1.4 ± 0.1	-	0
**7**	50.0 (17.5)	3.9 ± 0.2	3.0 ± 0.2	1.0 ± 0.1	0.98 ± 0.2	-	0

Values with recalculated standard deviations obtained from the fit of the data. 0: neither energy transfer nor sensitizer triplet quenching were observed, -: not measured, [a] anthracene, [b] anthanthrene, [c] TPP, [d] chlorophyll b, [e] Pt(II) phtalocyanine, [f] tetracene.

Determination of Pure Triplet-triplet Excited State Absorption Spectra of ***1-7***

Triplet-triplet excited state absorption spectra of **1**-**7** were obtained by linear analysis based on singlet depletion method used where the triplet and ground state absorption spectra overlap.[7,8] The method relies on the following assumptions: (i) The triplet and the ground state do not absorb in exactly the same region (their peaks may lie close to each other but are not identical). (ii) All photoexcited molecules either return to the ground state or form the triplet state.

Then, we can obtain the triplet-triplet absorption spectrum and the ratio of triplet to ground state concentrations by adding a multiple of the known ground state absorption spectrum to the triplet difference absorption spectrum in the spectral region close to the negative peak of the ground-state bleach (GSB), where we expect a linear profile for the triplet-triplet excited state absorption. From the fraction of the ground state absorption in the triplet difference absorption

spectrum and known molar absorption coefficient of the ground state absorption, the concentration of triplet excited molecules is obtained and the molar absorption coefficient of the triplet state is calculated.

Adapting the singlet depletion method, we take a region of the triplet difference absorption spectrum obtained from the global fit of the nanosecond TAS spectrotemporal evolution in the sample (triplet sensitization with anthracene) centered around a peak of the ground state absorption spectrum. A "baseline subtraction", defined by the endpoints of the specified region, allows a comparison of the linearity of each residual spectrum. Where the sum of the residuals of the resulting linearized curves (i.e., integration under the linearized curves) equals zero, we obtain the fraction of ground state spectrum *f*.

In Figure S2, we show the residual spectra with varying value of *f* for **1**-**7** in the left column. Spectra of the scaled ground-state bleach, triplet difference spectra, and the resulting triplet-triplet absorption spectra are shown in the right column.

1:

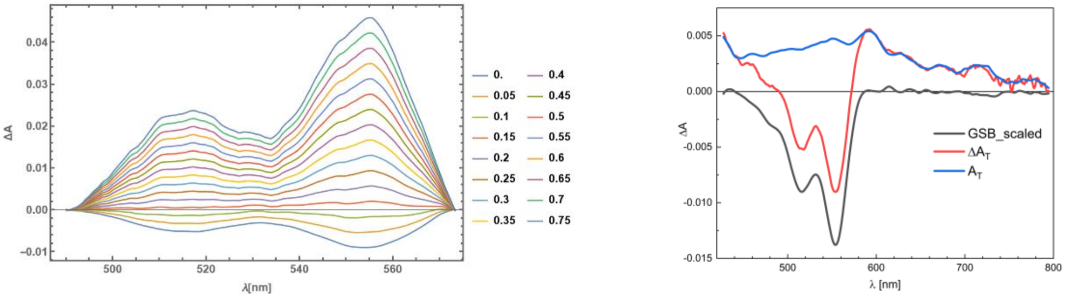

2:

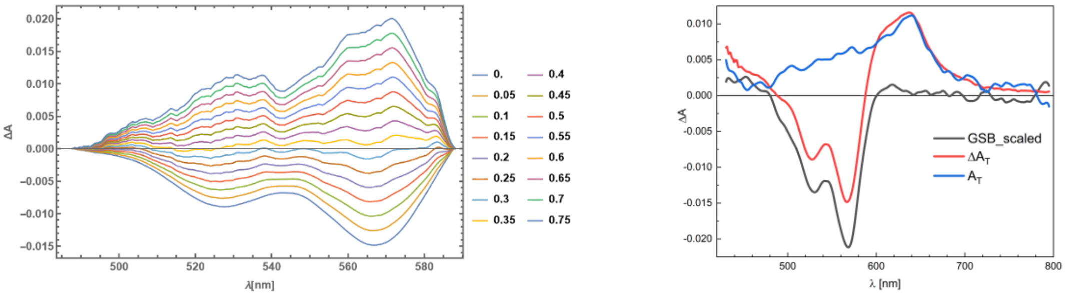

3:

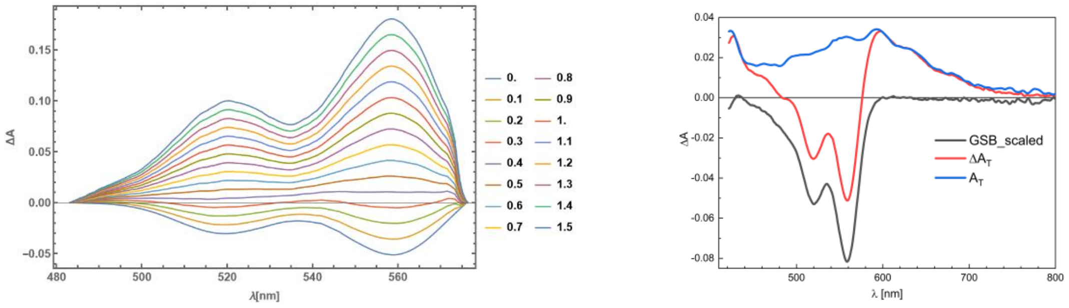
ΔA
λ[nm]
0.
0.1
0.2
0.3
0.4
0.5
0.6
0.7
0.8
0.9
1.
1.1
1.2
1.3
1.4
1.5
GSB_scaled
ΔA_T
A_T
λ [nm]

4:

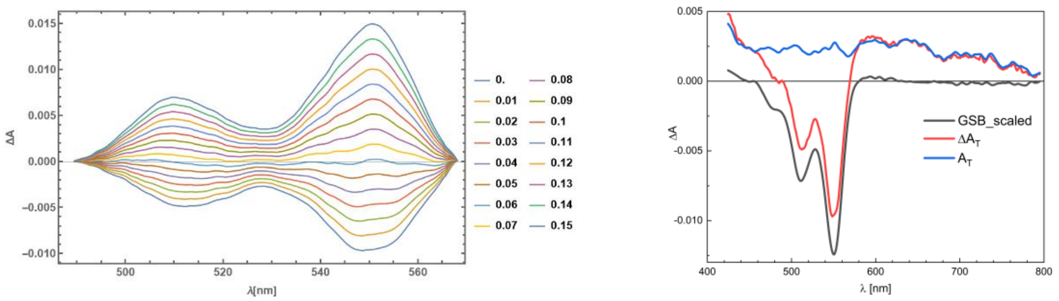
ΔA
λ[nm]
0.
0.01
0.02
0.03
0.04
0.05
0.06
0.07
0.08
0.09
0.1
0.11
0.12
0.13
0.14
0.15
GSB_scaled
ΔA_T
A_T
λ [nm]

5:

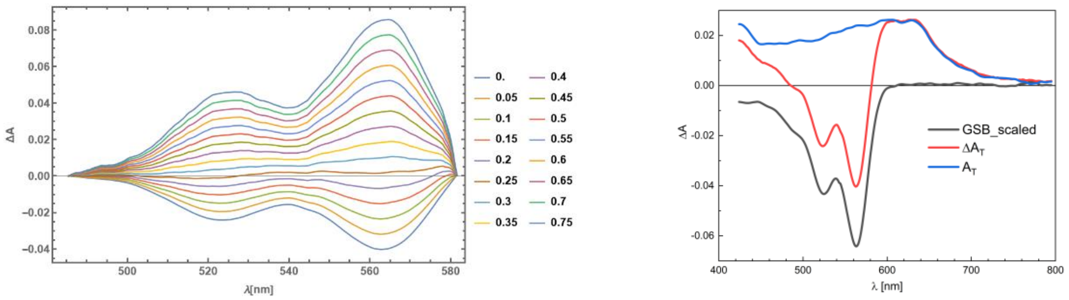
ΔA
λ[nm]
0.
0.05
0.1
0.15
0.2
0.25
0.3
0.35
0.4
0.45
0.5
0.55
0.6
0.65
0.7
0.75
GSB_scaled
ΔA_T
A_T
λ [nm]

6:

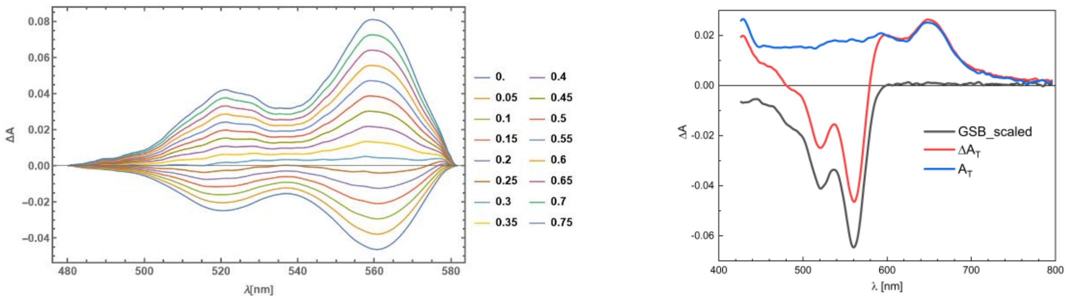
ΔA
λ[nm]
0.
0.05
0.1
0.15
0.2
0.25
0.3
0.35
0.4
0.45
0.5
0.55
0.6
0.65
0.7
0.75
GSB_scaled
ΔA_T
A_T
λ [nm]

7:

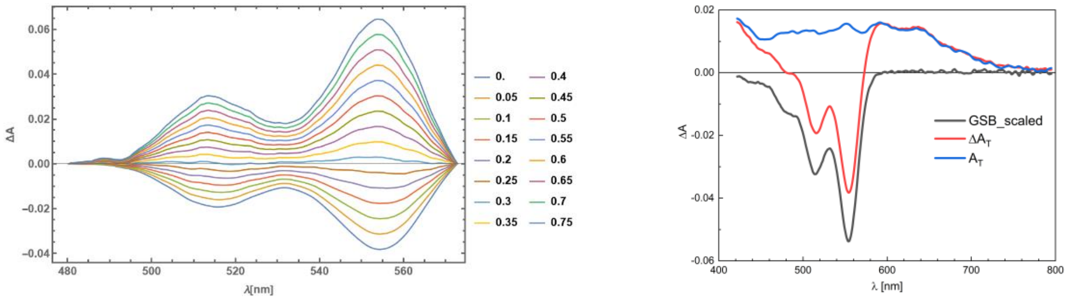

Figure S2: Triplet-triplet absorption spectra of **1**-**7** obtained by linear analysis using the singlet depletion method. Left column: residuals of linear analysis applied to triplet difference absorption spectra in the ground-state bleach region. Right column: scaled ground state absorption spectrum (black), triplet difference absorption spectrum obtained from global analysis (red), and triplet-triplet absorption spectrum (blue). Rows: **1**-**7**. Note: the ground state spectra are recorded with the same detector as the transient spectra.

X-Ray Structure, Crystal Packing and X-Ray Data

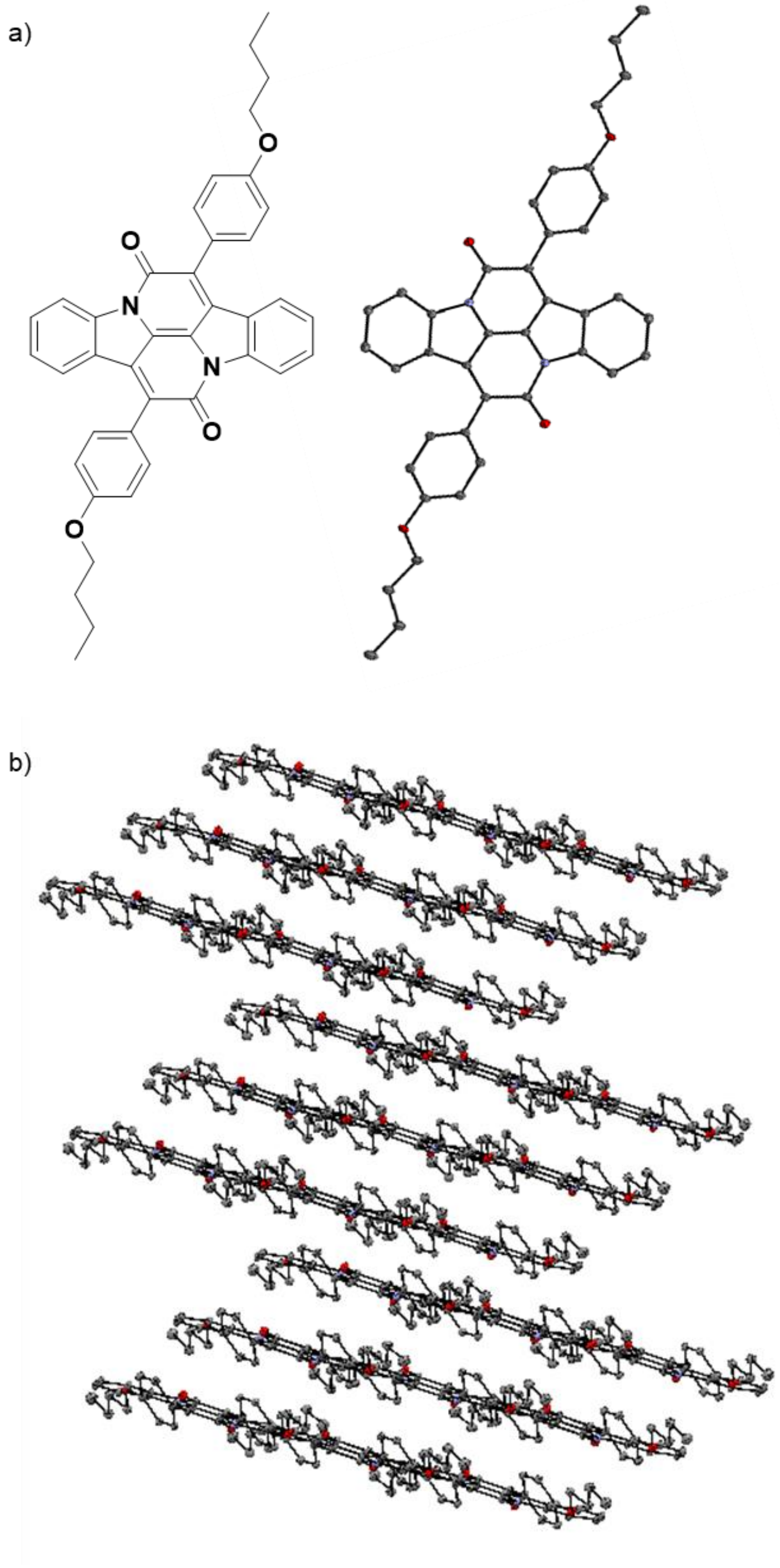

Figure S3. Cibalackrot **2**. a) X-Ray Structure (50% probability ellipsoids). b) Crystal Packing.

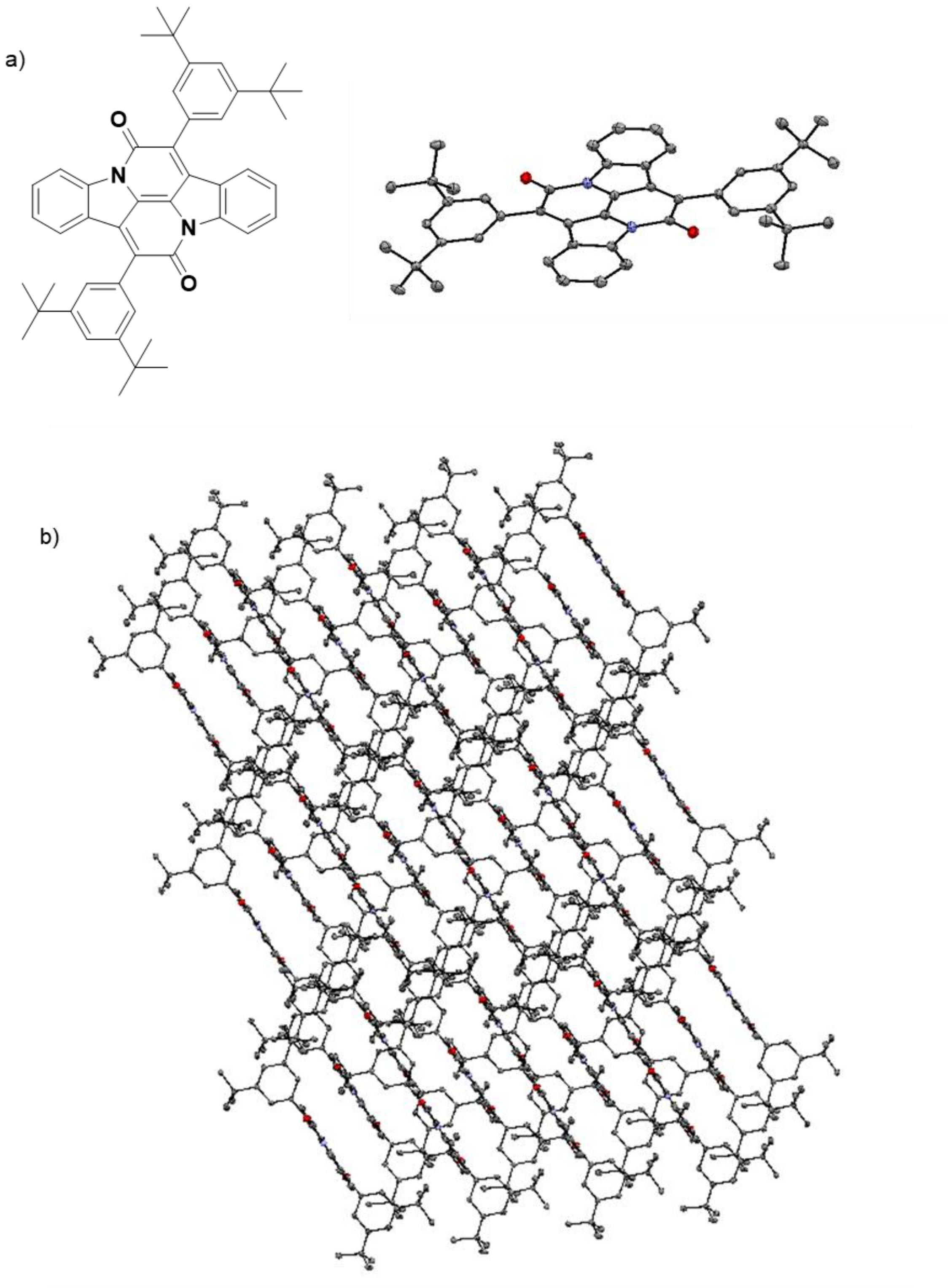

Figure S4. Cibalackrot **3**. a) X-Ray Structure (50% probability ellipsoids). b) Crystal Packing.

a)

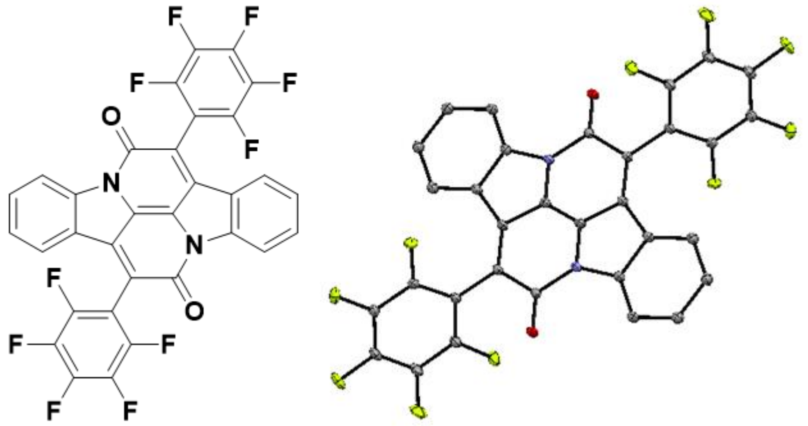

b)

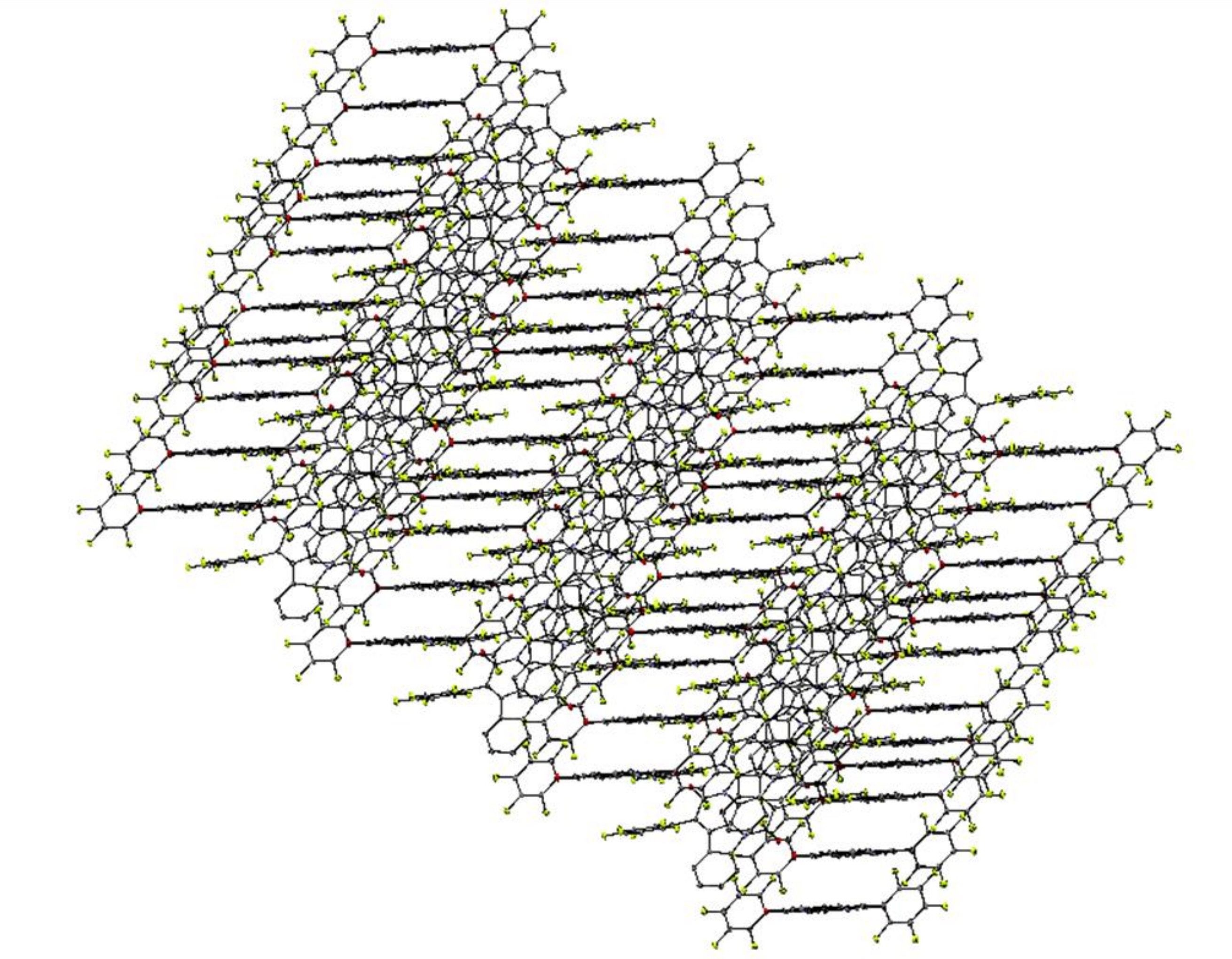

Figure **S5**. Cibalackrot **4**α. a) X-Ray structure (50% probability ellipsoids). b) Crystal Packing.

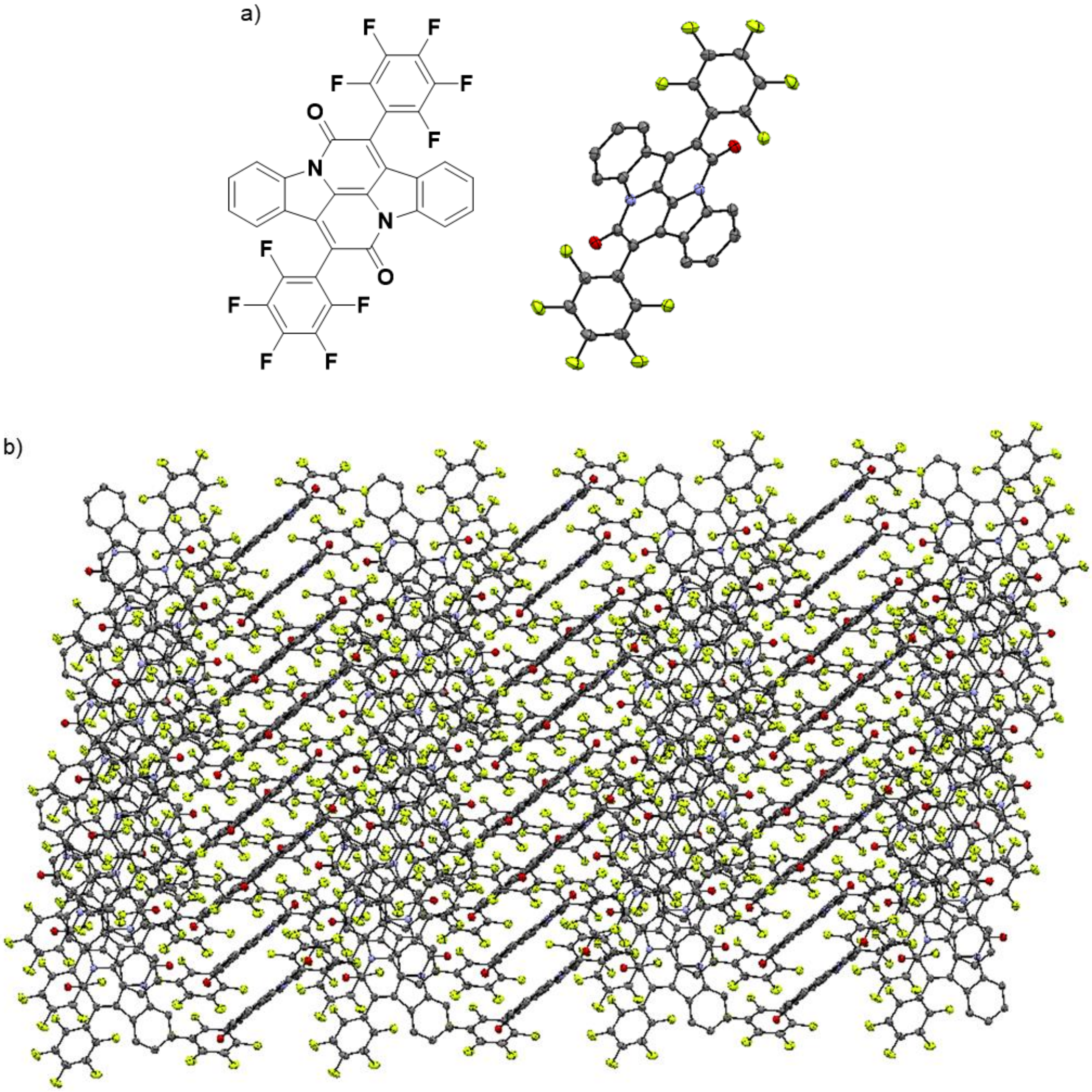

Figure S6. Cibalackrot **4β**. a) X-Ray Structure (50% probability ellipsoids). b) Crystal Packing.

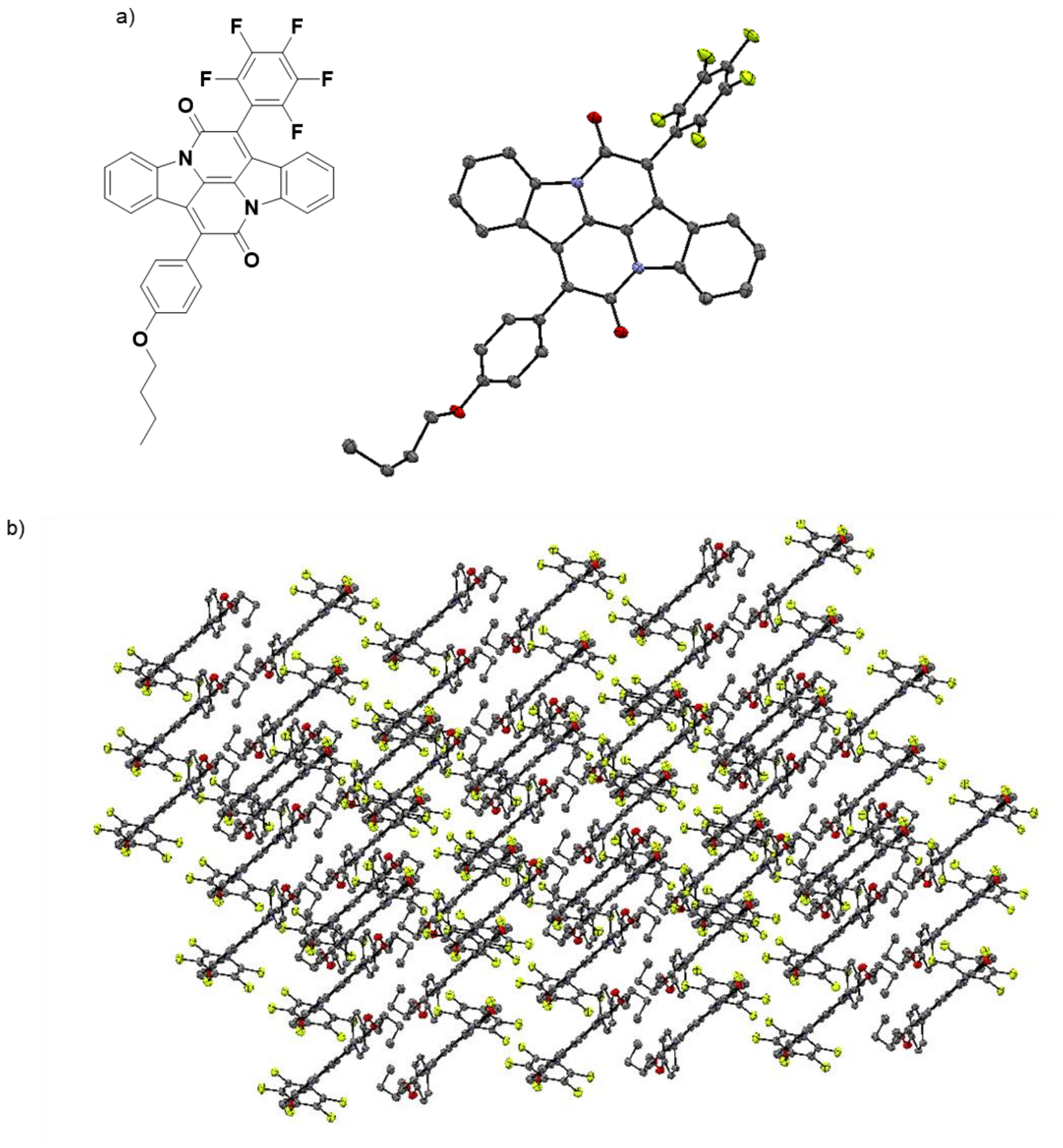

Figure S7. Cibalackrot **6** a) X-Ray Structure (50% probability ellipsoids). b) Crystal Packing.

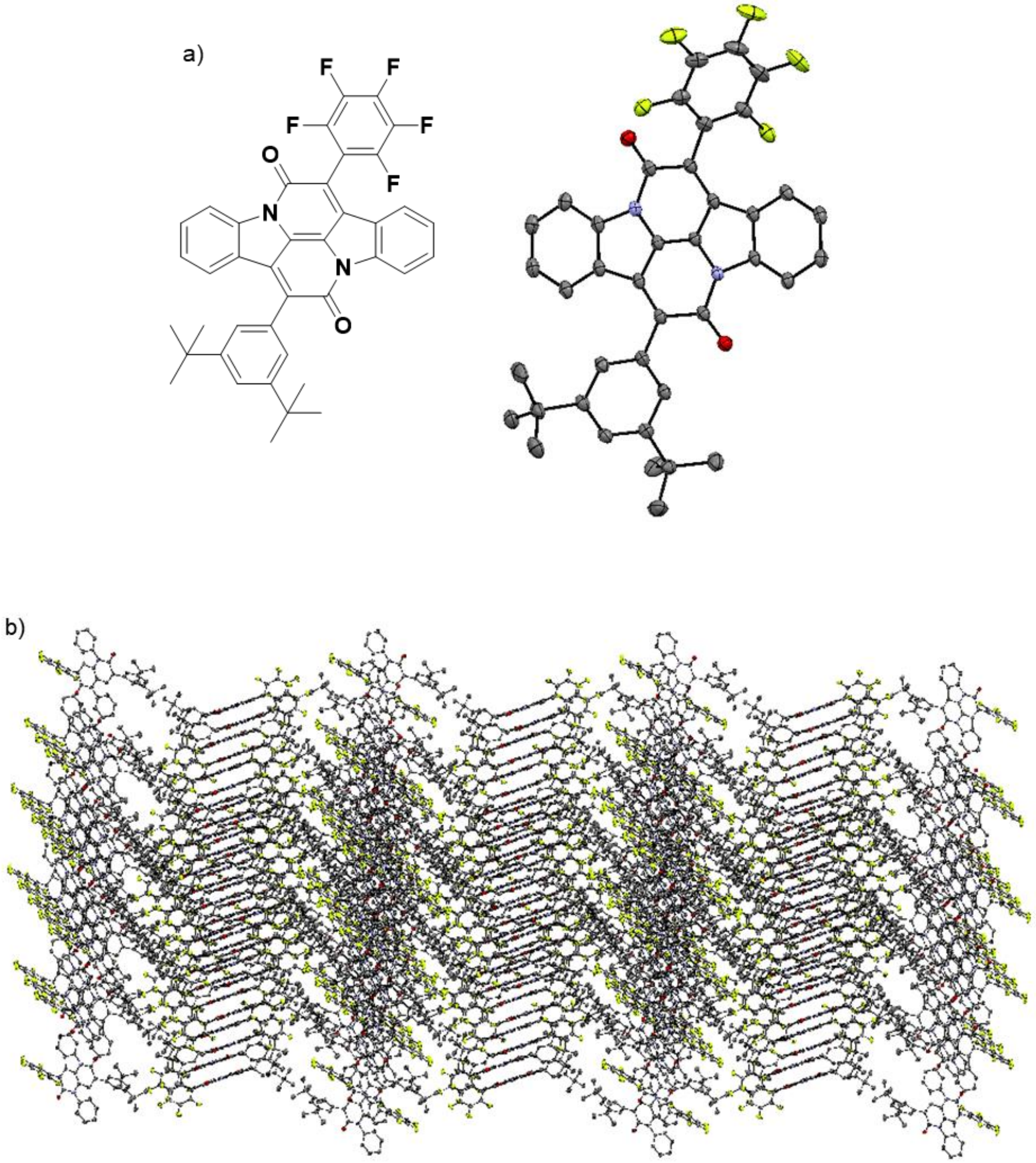

Figure S8. Cibalackrot **7** a) X-Ray Structure (50% probability ellipsoids). b) Crystal Packing.

Table S3. Parameters of Single Crystals of **2**, **3**, **4α**, **4β**, **6** and **7**.

	2	3	4α	4β	5	6	7
Empirical formula	$C_{40}H_{34}N_2O_4$	$C_{48}H_{50}N_2O_2$	$C_{32}H_8F_{10}N_2O_2$	$C_{32}H_8F_{10}N_2O_2$	$C_{44}H_{42}N_2O_3$	$C_{36}H_{21}F_5N_2O_3$	$C_{40}H_{29}F_5N_2O_2$
Formula weight	606.69	686.90	642.40	642.40	646.79	624.55	664.65
Temperature	100(2) K	100(2) K	100(2) K	100(2) K		100(2) K	100(2) K
Wavelength	0.7288 Å	0.7749 Å	0.7288 Å	0.8856 Å		0.7288 Å	0.7288 Å
Crystal system	Triclinic	Triclinic	Monoclinic	Monoclinic		Triclinic	Monoclinic
Space group	P-1	P-1	P2₁/c	P2₁/c		P-1	C2/c
Unit cell dimensions	a = 5.2469(5)Å<br>α= 75.262(4)°<br>b = 10.3256(10)Å<br>β= 81.752(4)°<br>c = 14.0369(13)Å<br>γ = 89.731(4)°	a = 5.6979(3) Å<br>α= 78.274(3)°<br>b = 8.9989(5) Å<br>β= 87.753(3)°<br>c = 18.2466(8) Å<br>γ = 88.618(4)°	a = 11.6402(4) Å<br>α= 90°<br>b = 6.9910(2) Å<br>β= 96.7850(10)°<br>c = 14.9437(5) Å<br>γ = 90°	a = 12.7732(8) Å<br>α= 90°<br>b = 5.3396(3) Å<br>β= 91.298(2)°<br>c = 17.8237(10) Å<br>γ = 90°		a = 6.1897(3) Å<br>α= 88.318(2)°<br>b = 12.8505(7) Å<br>β= 86.104(2)°<br>c = 16.9344(9) Å<br>γ = 85.364(2)°	a = 19.1223(12) Å<br>α= 90°<br>b = 9.4917(6) Å<br>β= 100.613(2)°<br>c = 35.098(2) Å<br>γ = 90°
Volume	727.45(12) $Å^3$	915.24(8) $Å^3$	1207.55(7) $Å^3$	1215.33(12) $Å^3$		1339.08(12) $Å^3$	6261.4(7) $Å^3$
Z	1	1	2	2		2	8
Density (calculated)	1.385 Mg/m^3	1.246 Mg/m^3	1.767 Mg/m^3	1.755 Mg/m^3		1.549 Mg/m^3	1.410 Mg/m^3
Absorption coefficient	0.093 mm^{-1}	0.089 mm^{-1}	0.172 mm^{-1}	0.283 mm^{-1}		0.128 mm^{-1}	0.112 mm^{-1}
F(000)	320	368	640	640		640	2752
Crystal size	0.080 x 0.010 x 0.010 mm^3	0.300 x 0.050 x 0.010 mm^3	0.170 x 0.140 x 0.010 mm^3	0.140 x 0.020 x 0.005 mm^3		0.350 x 0.020 x 0.005 mm^3	0.300 x 0.020 x 0.005 mm^3
Theta range for data collection	1.555 to 31.433°	2.487 to 26.538°	1.807 to 34.118°	1.987 to 35.147°		1.236 to 31.436°	2.327 to 28.346°
Reflections collected	26713	9589	46223	12532		44981	65766
Independent reflections	4462 [R(int) = 0.0450]	2801 [R(int) = 0.0381]	4612 [R(int) = 0.0521]	2768 [R(int) = 0.0580]		8208 [R(int) = 0.0509]	7230 [R(int) = 0.0528]
Max. and min. transmission	0.999 and 0.910	0.999 and 0.844	0.998 and 0.929	0.999 and 0.820		0.999 and 0.856	0.999 and 0.876

Data / restraints / parameters	4462 / 0 / 209	2801 / 0 / 242	4612 / 0 / 224	2768 / 0 / 224		8208 / 0 / 416	7230 / 0 / 448
Goodness-of-fit on F^2	1.077	1.066	1.067	1.106		1.048	1.024
Final R indices [I>2sigma(I)]	R1 = 0.0452, wR2 = 0.1212	R1 = 0.0404, wR2 = 0.0891	R1 = 0.0394, wR2 = 0.1065	R1 = 0.0517, wR2 = 0.1232		R1 = 0.0462, wR2 = 0.1253	R1 = 0.0449, wR2 = 0.1155
R indices (all data)	R1 = 0.0511, wR2 = 0.1266	R1 = 0.0522, wR2 = 0.0940	R1 = 0.0453, wR2 = 0.1112	R1 = 0.0659, wR2 = 0.1315		R1 = 0.0624, wR2 = 0.1363	R1 = 0.0574, wR2 = 0.1244
Largest diff. peak and hole	0.502 and -0.282 $eÅ^{-3}$	0.166 and -0.219 $eÅ^{-3}$	0.559 and -0.274 $eÅ^{-3}$	0.259 and -0.333 $eÅ^{-3}$		0.524 and -0.321 $eÅ^{-3}$	0.280 and -0.331 $eÅ^{-3}$

References

1. Padhye, M. R.; McGlynn, S. P.; Kasha, M., Lowest Triplet State of Anthracene. *J. Chem. Phys.* **1956,** *24* (3), 588-594.
2. Herkstroeter, W. G., Triplet energies of azulene, .beta.-carotene, and ferrocene. *J. Am. Chem. Soc.* **1975,** *97* (15), 4161-4167.
3. Montalti, M.; Credi, A.; Prodi, L.; Gandolfi, M. T., *Handbook of Photochemistry*. CRC Press: 2006.
4. Vincett, P. S.; Voigt, E. M.; Rieckhoff, K. E., Phosphorescence and Fluorescence of Phthalocyanines. *J. Chem. Phys.* **1971,** *55* (8), 4131-4140.
5. McGlynn, S. P.; Padhye, M. R.; Kasha, M., Lowest Triplet Levels of the Polyacenes. *J. Chem. Phys.* **1955,** *23* (3), 593-594.
6. Sandros, K., Transfer of triplet state energy in fluid solutions. *Acta Chem. Scand* **1964,** *18*, 2355-2374.
7. Carmichael, I.; Hug, G. L., Triplet–Triplet Absorption Spectra of Organic Molecules in Condensed Phases. *J. Phys. Chem. Ref. Data* **1986,** *15* (1), 1-250.
8. Mauck, C. M.; Hartnett, P. E.; Margulies, E. A.; Ma, L.; Miller, C. E.; Schatz, G. C.; Marks, T. J.; Wasielewski, M. R., Singlet Fission via an Excimer-Like Intermediate in 3,6-Bis(thiophen-2-yl)diketopyrrolopyrrole Derivatives. *J. Am. Chem. Soc.* **2016,** *138* (36), 11749-11761.
